# Human haematopoietic stem cells remember inflammatory stress

**DOI:** 10.1038/s41586-026-10522-7

**Published:** 2026-05-27

**Authors:** Andy G. X. Zeng, Murtaza S. Nagree, Niels Asger Jakobsen, Sayyam Shah, Angelica Varesi, Jasmine Ryu Won Kang, Alex Murison, Jin-Gyu Cheong, Sven Turkalj, Xuan Zhang, Felix A. Radtke, Tsega-Ab Abera, Isabel N. X. Lim, Liqing Jin, Joana Araújo, Alicia G. Aguilar-Navarro, Darrien Parris, Jessica McLeod, Hyerin Kim, Ho Seok Lee, Lin Zhang, Mason Boulanger, Elyssa Bader, Elias Gbeha, Christopher N. Parkhurst, Elvin Wagenblast, Eugenia Flores-Figueroa, Bo Wang, Gregory W. Schwartz, Leonard D. Shultz, Anna S. Nam, H. Leighton Grimes, Steven Z. Josefowicz, Philip Awadalla, Paresh Vyas, John E. Dick, Stephanie Z. Xie

**Affiliations:** 1 Princess Margaret Cancer Centre, University Health Network, Toronto, Ontario Canada; 2 Department of Molecular Genetics, University of Toronto, Toronto, Ontario Canada; 3 MRC Molecular Haematology Unit, MRC Weatherall Institute of Molecular Medicine, Radcliffe Department of Medicine, University of Oxford, Oxford, UK; 4 NIHR Oxford Biomedical Research Centre, Oxford, UK; 5 Temerty Faculty of Medicine, University of Toronto, Toronto, Ontario Canada; 6 Ontario Institute for Cancer Research, Toronto, Ontario Canada; 7 Department of Pathology and Laboratory Medicine, Weill Cornell Medicine, New York, NY USA; 8 Immunology and Microbial Pathogenesis Program, Weill Cornell Graduate School, NewYork, NY USA; 9 Department of Pediatric Oncology, Dana-Farber Cancer Institute and Boston Children’s Hospital, Boston, MA USA; 10 Division of Immunobiology, Cincinnati Children’s Hospital Medical Center, Cincinnati, OH USA; 11 Department of Hematology, Centro Hospitalar Universitário de São João, Porto, Portugal; 12 Faculty of Medicine, University of Porto, Porto, Portugal; 13 Instituto de Investigação e Inovação em Saúde, University of Porto, Porto, Portugal; 14 Instituto Nacional de Investigação Biomédica, University of Porto, Porto, Portugal; 15 Peter Munk Cardiac Centre, University Health Network, Toronto, Ontario Canada; 16 Department of Statistics and Actuarial Science, Simon Fraser University, Burnaby, British Columbia Canada; 17 Division of Pulmonary and Critical Care Medicine, Weill Cornell Graduate School, NewYork, NY USA; 18 Icahn School of Medicine at Mount Sinai, New York, NY USA; 19 Department of Computer Science, University of Toronto, Toronto, Ontario Canada; 20 Vector Institute, Toronto, Ontario Canada; 21 Department of Laboratory Medicine and Pathobiology, University of Toronto, Toronto, Ontario Canada; 22 Department of Medical Biophysics, University of Toronto, Toronto, Ontario Canada; 23 The Jackson Laboratory, Bar Harbor, ME USA; 24 Division of Immunobiology, Cincinnati Children’s Hospital Medical Center, Department of Pediatrics, University of Cincinnati, Cincinnati, OH USA; 25 Nuffield Department of Population Health, Big Data Institute, University of Oxford, Oxford, UK; 26 Department of Clinical Haematology, Oxford University Hospitals NHS Foundation Trust, Oxford, UK

**Keywords:** Haematopoietic stem cells, Inflammation, Innate immunity, Ageing

## Abstract

Inflammation activates blood cells, contributing to ageing and malignancy^1–3^. Haematopoietic stem cells (HSCs) survive a lifetime of infection to sustain life-long haematopoiesis^1–9^, but how human HSCs respond and adapt to inflammatory stress is largely unknown. Here, to empirically understand this adaptation, we developed xenograft inflammation–recovery models and performed single-cell multiomics on xenografted human HSCs. Two transcriptionally and epigenetically distinct HSC subsets were identified with one, termed HSC inflammatory memory (HSC-iM), retaining a molecular memory of previous inflammatory treatments. The HSC-iM subset exhibited quiescence and restrained haematopoietic output. Molecularly, the HSC-iM program was enriched in HSCs from adult and paediatric samples across conditions ranging from COVID-19 recovery, sickle cell disease, ageing and clonal haematopoiesis, establishing both the validity of our xenograft models and the physiological relevance of HSC-iM. Clonal haematopoiesis mutations in HSC-iM attenuated the effects of inflammatory stress by promoting HSC activation and differentiation. Moreover, transmission of the pro-inflammatory HSC-iM transcriptional program to differentiated immune progeny was demonstrated in xenograft and physiological settings. Finally, HSC-iM program enrichment in circulating blood cells was associated with a heightened risk score for all-cause mortality in population cohort analyses, underscoring the clinical relevance of this newly identified HSC subset in characterizing heterogeneous health outcomes across a lifetime.

## Abstract (web summary)

Using xenograft inflammation–recovery models and single-cell multiomics, a haematopoietic stem cell population after inflammatory stress is identified.

## Main

Humans have enormous demand for haematopoietic output that is met by a complex cellular hierarchy with HSCs at its apex^10^. Mature blood cells with a finite lifespan are continuously replenished by bone marrow haematopoietic stem and progenitor cells (HSPCs) producing 3 million cells per second in human adults, via a tightly controlled process^11^. Human HSCs are both genetically and functionally diverse with 50,000–200,000 HSCs contributing to haematopoiesis at any one time^12^. With age, HSCs show functional decline^1,9^, have a marked decrease in clonal diversity^7^, a consequent increase in incidence of clonal haematopoiesis (CH), and an increased risk for blood malignancy^13^. How daily blood production is coordinated with HSC pool maintenance over a lifespan is unclear, especially when exposed to inflammatory stressors, for example, recurrent infections, which can induce HSC activation^3,4,14,15^. In addition, HSC fate transitions from quiescence towards activation are essential for HSC function, but can be dysregulated with ageing and inflammatory stress^16–20^. Finally, HSCs are not homogeneous; HSCs exhibit transcriptional, epigenetic and functional heterogeneity, where inflammatory pathways are a major source of variation^14,18–22^. Together, this indicates that heterogeneous cellular responses to inflammation within the human HSC pool might drive ageing phenotypes in response to life-long inflammatory insults^9,23,24^.

Human lineage tracking shows that pre-leukaemic CH mutations arise decades before disease detection^25–28^. CH mutation-bearing clones occur ubiquitously in adults older than 60 years of age, although only a fraction expand to reach detectable clone size^13^. Crucially, mechanisms regulating CH clone size are poorly understood. Mouse models have shown that inflammation activates HSCs, consequently impairing differentiation and self-renewal^1–5,15,29,30^, affecting clonal selection in CH^6,29,31^. Some inflammatory responses include activation of target genes downstream of tumour necrosis factor (TNF) via NF-κB, known to regulate HSC survival in both mouse and human settings^3,14^. Repeated inflammatory challenges have also been associated with sustained epigenetic changes^30^ and accelerated ageing of mouse HSCs^1,4^, as well as expansion of clones bearing CH-associated mutations^5,6,29,31^. However, given the heterogeneity of the HSC compartment, it is unclear whether all HSCs, especially in humans, respond equally to inflammation. If HSCs respond heterogeneously, it is also unclear whether this would affect clonal selection such as in CH.

## Inflammatory response priming in LT-HSC

**Fig. 1:**
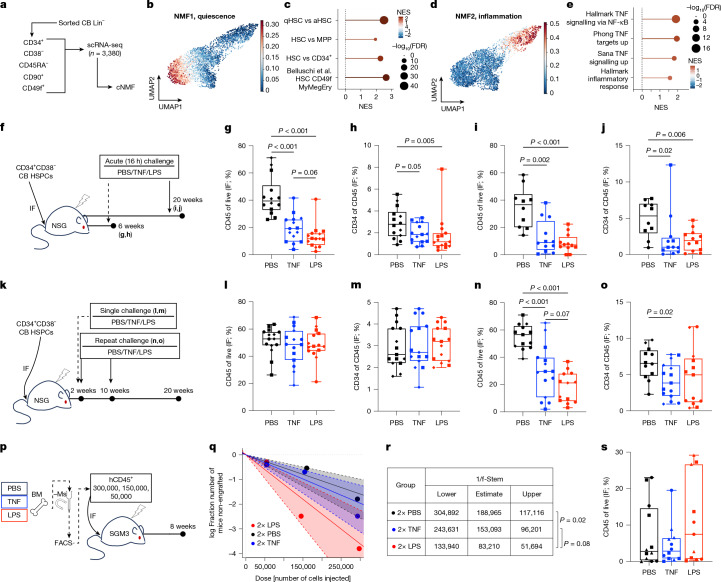
Modelling inflammatory response and recovery in human LT-HSC. **a**, CB LT-HSCs were subjected to scRNA-seq. Consensus NMF (cNMF) was performed on 3,381 cells. **b**–**e**, Projection of a cNMF transcriptional program of quiescence (NMF1; **b**) or inflammation (NMF2; **d**), as defined by GSEA enrichment (**c**,**e**, respectively). aHSC, activated HSC; FDR, false discovery rate; MyMegEry, myeloid-megakaryocyte-erythroid lineage potential; NES, normalized enrichment score; qHSC, quiescent HSC; UMAP, uniform manifold approximation and projection. **f**–**j**, NSG mice xenografted with CB-derived CD34^+^CD38^−^ cells were challenged with TNF or LPS at either 6 weeks (**g**,**h**; *n* = 14 for the PBS and *n* = 15 for the TNF and LPS groups across 3 independent pools) or 20 weeks (**i**,**j**; *n* = 10 for the PBS and *n* = 12 for the TNF and LPS groups across 2 independent pools) post-transplant. Total human engraftment (**g**,**i**) and progenitors (**h**,**j**) 16 h after inflammatory challenge or vehicle were measured. IF, injected femur. **k**, Schematic of the inflammation–recovery xenograft models. **l**,**m**, Human engraftment (**l**) and progenitor composition (**m**) 18 weeks after a single challenge (*n* = 15 mice per group across 3 independent CB pools). **n**,**o**, Human engraftment (**n**) and progenitor composition (**o**) 10 weeks after repeated challenge (*n* = 13 mice in PBS and LPS groups, and *n* = 15 in the TNF group across 3 independent CB pools). **p**, Schematic of the secondary transplantation with limiting dilution of human leukocytes from repeat-challenged xenografts. BM, bone marrow; FACS, fluorescence-activated cell sorting; −Ms, mouse depletion. **q**,**r**, Stem cell frequency (f-Stem) estimates for **p**. Dashed lines and shaded regions show 95% confidence intervals for each group. **s**, Human engraftment from **p** transplanted with 300,000 hCD45^+^ cells (*n* = 12 for PBS and TNF groups and *n* = 11 for the LPS group across 3 independent pools). Engraftment data are presented as boxplots: quartiles indicate a line at the median; the whiskers show the range; individual points are per mouse; and the symbols reflect independent CB pools. Engraftment data were compared with pairwise two-sided Mann–Whitney tests, whereas chi-squared tests were used for stem cell frequencies; *P* < 0.1 is shown numerically, whereas *P* > 0.1 is not shown. Source data

**Extended Data Fig. 1:**
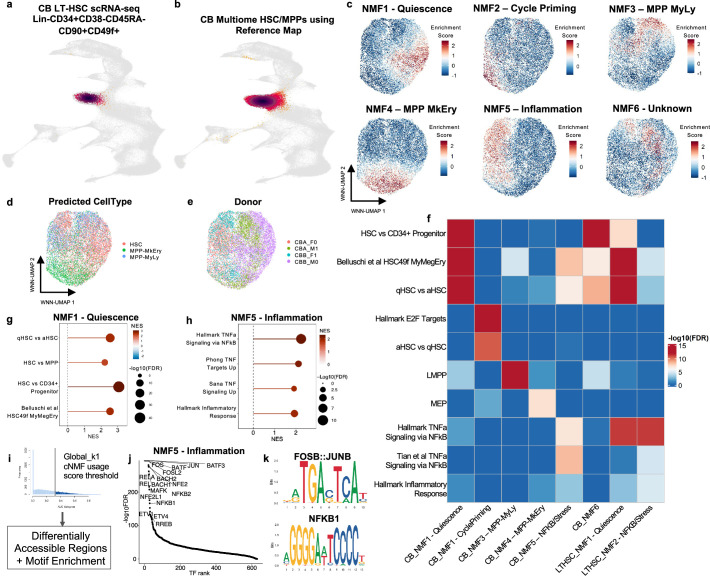
Validation and characterization of inflammatory programs from CB HSPCs. **a**,**b**, Density plot of cells mapped onto BoneMarrowMap. **c**, Enrichment of cNMF programs on a UMAP embedding of CB HSC/MPPs, spanning NMF1: Quiescence, NMF2: Cycle Priming, NMF3: MPP-MyLy, NMF4: MPP-MkEry, NMF5: Inflammation, and NMF6: Unknown. **d**,**e**, CB HSC/MPP annotations pertaining to cell type (d) and donor (e). **f**, Key pathways significant in cNMF programs defined on the CB scMultiome HSC/MPPs and LT- HSC scRNA-seq. **g**,**h**, GSEA pathway enrichment for CB scMultiome NMF signatures NMF1: Quiescence (g) and NMF5: Inflammation (h). **i**, Schematic outlining TF motif enrichment analysis among differentially accessible regions specific to cells that pass a threshold for the CB scMultiome NMF5: Inflammation program. **j**, Top motifs in cells enriched with the NMF5: Inflammation program in the CB scMultiome. **k**, Two candidate motifs from the AP-1 and NFkB family. Source data

Human HSC can be deeply quiescent or cell-cycle primed; states tied to variations in regeneration kinetics, 3D chromatin architecture and endolysosomal activity^14,19–21^. We performed single-cell transcriptional profiling of cord blood (CB) Lin^−^CD34^+^CD38^−^CD45RA^−^CD90^+^CD49f^+^ long-term HSCs (LT-HSCs) and used non-negative matrix factorization (NMF) to explore transcriptional heterogeneity among 3,381 LT-HSCs at homeostasis (Fig. 1a). Programs of quiescence (Fig. 1b,c) and inflammatory signalling (Fig. 1d,e) were identified (Supplementary Tables 1 and 2). These were independently validated in single-nucleus gene expression and chromatin accessibility profiles (hereafter, scMultiome) of 15,590 CB HSCs and multipotent progenitors (HSC–MPPs) as identified using BoneMarrowMap^32^ (Extended Data Fig. 1a–h and Supplementary Table 3). The inflammatory program was associated with chromatin accessibility of AP-1 and NF-κB transcription factor-binding motifs (Extended Data Fig. 1i–k). To jointly evaluate the relevance of the distinct inflammation and quiescence programs identified by NMF within these datasets, ‘meta-programs’ were defined by consensus; these also showed stronger enrichment for inflammation-specific and quiescence-specific programs, respectively (Supplementary Table 4 and Supplementary Note 1). These data corroborate previous findings^19–22^ of variable levels of transcriptional inflammatory priming within individual CB HSC-MPPs.

## Modelling human HSC inflammation in vivo

**Extended Data Fig. 2:**
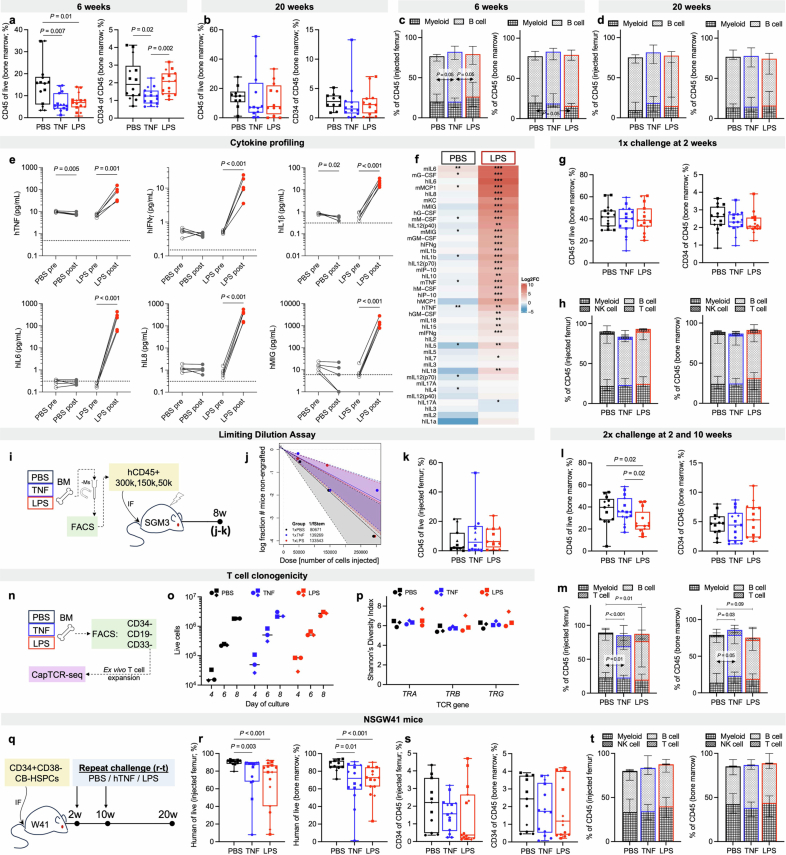
Recovery and lineage distribution in human HSC grafts after acute and repeated inflammatory challenges. **a**, Effect of acute inflammatory challenge in xenografts 6 weeks post-transplant on human CD45+ cells and CD34+ progenitors in non-injected bone marrow (BM) 16 h after administration (n = 14 for the PBS group and n = 15 for the hTNF and LPS groups across 3 independent CB pools). **b**, Effect of acute inflammatory challenge of xenografts 20 weeks post-transplant on human cells and progenitors in non-injected BM 16 h after administration (n = 10 for the PBS group and n = 12 for the hTNF and LPS groups across 2 independent CB pools). **c**, Proportion of human B cell (CD19+) versus myeloid (CD33+) composition in injected or non-injected BM of mice in (a). **d**, Proportion of human B cell versus myeloid composition in injected or non-injected BM of mice in (b). **e**,**f**, Blood plasma from xenografted mice (n = 5 mice per group transplanted with 1 CB pool) was sampled 6 weeks post-transplant before and after an acute challenge with LPS or PBS control, and a panel of human (23) and mouse (18) cytokines was measured using a multiplex assay. Data for a subset of human cytokines in individual mice is shown as individual dots with lines connecting levels from matching mice in (e); the dashed horizontal line shows the lower detection limit. Data for the full panel is shown as a heatmap in (f). **g**, Human engraftment and progenitor composition in non-injected BM 18 weeks after a single inflammatory challenge (n = 15 mice per group across 3 independent CB pools). **h**, Proportion of human B cell versus myeloid versus T/NK cell (CD3+ and CD56+, respectively) composition in injected or non-injected BM of mice from (g). **i**, Experimental schematic for secondary transplantation of single-challenged xenografts with limiting dilution. **j**, Stem cell frequency estimates calculated for secondary transplants in (i). No significant differences were found by chi-squared tests. **k**, Proportion of engrafted human cells in cohorts from (i) transplanted with 300,000 hCD45+ cells (n = 11 for the PBS group and n = 12 for the TNF and LPS groups across 3 independent CB pools). **l**, Human engraftment and progenitor composition in non-injected BM 10 weeks after repeated inflammatory challenge (n = 13 mice in PBS and LPS groups, and n = 15 in the TNF group across 3 independent CB pools). **m**, Proportion of human B cell versus myeloid versus T cell composition in injected or non-injected BM of mice from (l). **n**, T cells from repeated challenge and 10w recovery xenografts were expanded in culture and T cell receptor (TCR) repertoire was evaluated by CapTCR-seq. **o**,**p**, Live cell counts (o) were determined via trypan blue exclusion at the indicated time points. Shannon’s diversity index (p) was calculated for the unique sequences obtained for the three indicated TCR genes. Each point/symbol reflects data obtained from pooled T cells (n = 3 pools) from a group of mice engrafted with different CB pools. **q**, Schematic for xenograft model of recovery from repeated (2 weeks and 10 weeks) acute inflammatory challenge in NSGW41 mice. **r**, Engraftment of total human cells - calculated by combining CD45+ and GlyA+ proportions - in injected and non-injected BM from repeat-challenged NSGW41 xenografts (n = 11, n = 14 and n = 15 mice in the PBS, TNF and LPS groups, respectively, across 3 independent CB pools). **s**,**t**, Proportion of CD34 + CD19 − CD33− progenitors (s), or lineage composition by myeloid (CD33+), B (CD19+), NK (CD56+) and T (CD3+) cells (t), in injected and non-injected BM from xenografts in (r). All engraftment data is presented as box plots with a line at the median, boxes showing quartiles and error bars showing range. Individual points show data for each animal used in the study, and different symbols reflect different CB pools. Stacked bar plots for B/T/NK/Myeloid proportions represent median composition from the same experiments, and error bars reflect interquartile range. All xenograft data were compared using pairwise two-sided Mann-Whitney tests. Data for cytokines pre- and post-challenge with LPS were compared using two-sided paired-ratio t tests; *** *p* < 0.001, ** *p* < 0.01, * *p* < 0.05 and *p* > 0.05 is not shown. Source data

To investigate how CB HSCs would functionally respond to inflammatory stress, we developed a xenotransplantation model of inflammation and focused on the impact of lipopolysaccharide (LPS), which mimics sepsis^33^, and TNF, which has been associated with deleterious effects on human health in the context of advanced age, COVID-19 and sepsis^34–36^. To examine acute inflammatory responses (Fig. 1f), xenotransplanted mice were treated with phosphate-buffered saline (PBS), TNF or LPS and examined 16 h later at 6 weeks, when the engrafted human HSCs are mostly cycling (Fig. 1g,h and Extended Data Fig. 2a), or 20 weeks when engrafted HSC are mainly quiescent^22^ (Fig. 1i,j and Extended Data Fig. 2b). Human engraftment within the injected femur derives from a combination of HSCs and progenitors, whereas the non-injected bone marrow compartment arises from self-renewing HSCs that migrated from the injected femur^20,37^. Both injected femur hCD45^+^ cells and hCD34^+^ progenitors were reduced after TNF or LPS treatment at 6 weeks (Fig. 1g,h) and 20 weeks (Fig. 1i,j) compared with PBS controls. Variable responses were observed in the non-injected bone marrow (Extended Data Fig. 2a,b). B lymphoid and myeloid lineage proportions were unchanged (Extended Data Fig. 2c,d). A broad human inflammatory cytokine response to LPS was measured (Extended Data Fig. 2e,f and Supplementary Table 5). Engraftment analysis at 20 weeks showed recovery following a single challenge of either treatment at 2 weeks post-transplant (Fig. 1k–m and Extended Data Fig. 2g). No significant differences in lineage distribution were observed (Extended Data Fig. 2h). Limiting dilution assays into secondary NSG-SGM3 mice showed that stem cell frequencies remained unchanged between conditions (Extended Data Fig. 2i–k and Supplementary Table 6). Overall, these findings demonstrate a negative effect of acute inflammatory stimuli on human grafts in a xenograft model, consistent with previous mouse studies^1^, but there was full long-term recovery.

We then developed a model to investigate the effect of repeated inflammatory insults on human HSC function: xenografted NSG mice were treated with PBS, TNF or LPS at 2 weeks and 10 weeks post-transplant followed by a 10-week recovery period (Fig. 1k). In contrast to the single-challenge model, the hCD45^+^ engraftment in the injected femur at 20 weeks remained significantly lower in LPS-treated or TNF-treated groups than PBS, despite the recovery period (Fig. 1n). The proportion of hCD34^+^ cells was also reduced with TNF challenge (Fig. 1o). Only LPS challenge affected engraftment in the non-injected bone marrow, possibly reflective of a role for the magnitude of inflammatory insult (Extended Data Fig. 2l). Lineage distribution after recovery of the human grafts was also variable (Extended Data Fig. 2m). Of note, CD3^+^ T cells were significantly increased upon recovery from LPS or TNF at the expense of CD19^+^ B cells (Extended Data Fig. 2m); this was not due to clonal T cell expansion (Extended Data Fig. 2n–p). Human engraftment was also significantly reduced after repeated challenge in xenograft recipients (NSGW41) that were not irradiated (Extended Data Fig. 2q,r), although CD34^+^ HSPCs and mature lineages were unchanged (Extended Data Fig. 2s,t). Secondary limiting dilution assay transplants (Fig. 1p) demonstrated that HSCs with repopulating capacity were still present at similar frequency in the PBS and TNF groups, and with modest increase in the LPS group (Fig. 1q–s and Supplementary Table 6). This contrasts with mouse models of chronic inflammation where HSCs failed to recover functional potency up to 1 year after challenge^1,4^. Overall, our model reveals lasting functional changes in xenografted human HSCs after recovery from repeated inflammatory stress.

## Identification of two human HSC subsets

**Fig. 2:**
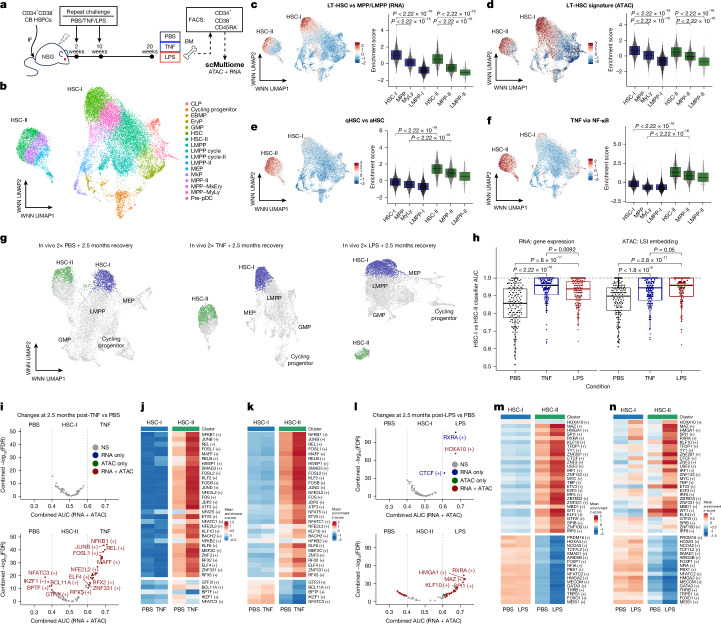
Identification of a human HSC subset with durable memory of inflammation after recovery in a xenograft model. **a**, Schematic outlining scMultiome profiling of HSPCs from inflammatory–recovery xenografts. **b**, UMAP of 27,492 HSPCs based on integrated RNA + ATAC embeddings from weighted nearest-neighbour (WNN) analysis. CLP, common lymphoid progenitor; EBMP, erythroid-basophil-megakaryocyte-biased progenitor; EryP, erythroid progenitor; MEP, megakaryocyte–erythroid progenitor; MkP, megakaryocyte progenitor; MPP-MyLy, myeloid-lymphoid-primed multipotent progenitors; pDC, plasmacytoid dendritic cells. **c**–**f**, Normalized enrichment (AUCell) scores of LT-HSC-specific gene (**c**) or chromatin (**d**) signatures, quiescent versus activated HSC signature (**e**) and a hallmark TNF-via-NF-κB signalling gene set (**f**) overlaid on the WNN UMAP and also depicted as boxplots (quartiles with median are indicated by a line, and whiskers show the range) for the indicated populations (*n* = 3,830 for HSC-I; *n* = 2,146 for HSC-II; *n* = 5,148 for LMPP-I; *n* = 539 for LMPP-II; *n* = 2,779 for MPP MyLy; and *n* = 2,317 for MPP-II). Statistical comparisons were made with two-sided Wilcoxon rank-sum tests. **g**, WNN UMAPs of HSPCs separated by treatment condition: PBS (*n* = 9,756), TNF (*n* = 8,785) or LPS (*n* = 8,951). **h**, Separability of HSC-I and HSC-II within each experimental condition using Augur. Each point represents the area under curve (AUC) of a random forest classifier trained to predict HSC-I versus HSC-II status from 150 subsamples of the data, summarized as a boxplot (the interquartile range with a line at the median and whiskers showing the range). A maximum AUC of 1, shown with a dashed horizontal line, indicates near-perfect separability. Distributions were compared using two-sided Wilcoxon rank-sum tests. LSI, latent semantic indexing. **i**–**k**, Differential transcription factor activity (SCENIC+) between TNF challenge versus PBS control, stratified by HSC subset. The coloured points in the volcano plots (**i**) have significance at FDR < 0.05 and 0.4 > AUC > 0.6. Transcription factors that met this criteria by both RNA and ATAC are coloured in red; the ‘(+)’ notation indicates positive correlation between chromatin accessibility and target expression. The mean scores for differentially enriched transcription factors by chromatin accessibility (**j**) or gene expression (**k**) are depicted for each condition and each HSC subset. NS, not significant. **l**–**n**, Differential transcription factor activity between LPS challenge versus PBS control, presented as in **i**–**k**. Source data

**Extended Data Fig. 3:**
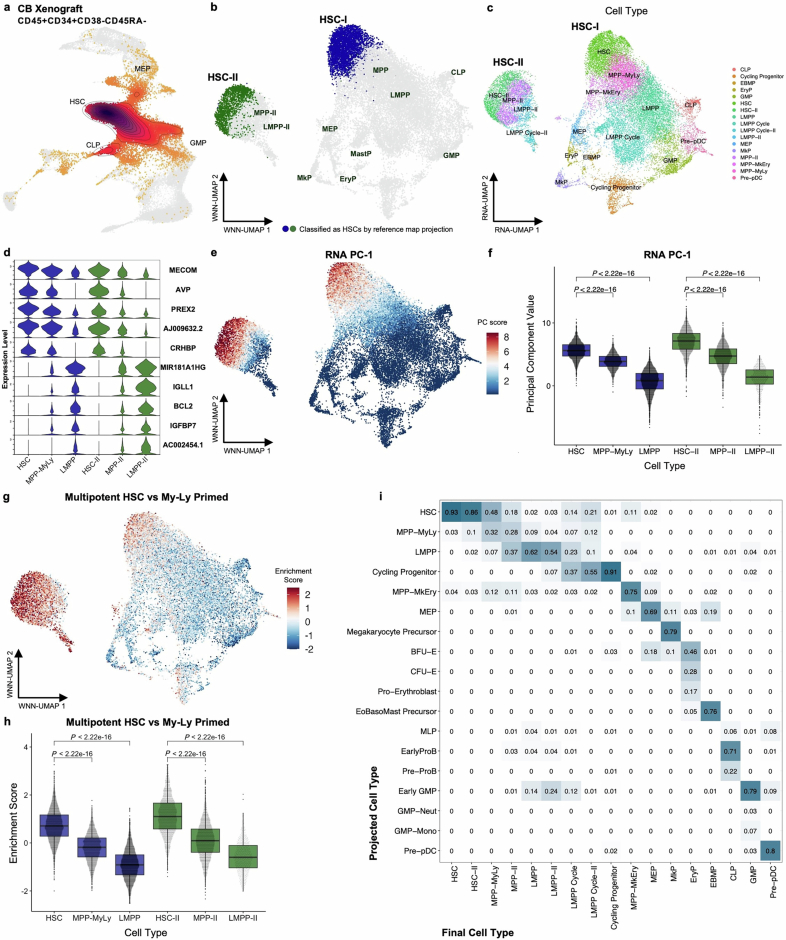
Cell type characterization of repeated challenge CB xenograft scMultiome profiles. **a**, Projection results of snRNA-seq from CD45 + CD34 + CD38 − CD45RA− cells profiled by scMultiome, sorted from 20-week cord blood xenografts in the setting of repeated inflammatory challenge and recovery. **b**, 7,507 CB xenograft cells identified as HSCs by reference map projection (a) of xenografted cells. **c**, UMAP embedding for xenografted HSPCs based on gene expression. **d**, Normalized expression of marker genes specific to HSC or LMPP. **e**,**f**, Value of gene expression principal component 1 (PC1) overlaid on the WNN UMAP embedding (e) and depicted as boxplots (interquartile range with a line at the median and whiskers show minimum and maximum values) for HSC (n = 3,830), MPP (n = 2,779), LMPP (n = 5,148), and HSC-II (n = 2,146), MPP-II (n = 2,317), LMPP-II (n = 539) populations (f). Statistical comparisons are made with two-sided Wilcoxon rank sum tests. **g**,**h**, Normalized enrichment score (AUCell) of a signature of multipotent human HSC compared to myelo-lymphoid lineage restricted human HSC. Enrichment is shown on the WNN UMAP embedding (g) and depicted as boxplots (h), displayed and compared as in (f). **i**, Confusion matrix depicting the final cell types assigned by focused RNA + ATAC-based clustering (columns) with projected cell types based on reference gene expression profiles from BoneMarrowMap (rows); proportions are normalized by column. Source data

To understand the molecular basis for the decreased human graft size observed after repeated inflammatory stress, CD45^+^CD34^+^CD38^−^CD45RA^−^ human HSPCs were isolated from NSG inflammation–recovery xenografts at 20 weeks and subjected to scMultiome (Fig. 2a). Cell-type assignment of 27,492 transcriptomes using BoneMarrowMap (Supplementary Table 7) identified expected primitive HSC–MPPs as well as granulocyte–monocyte progenitors (GMPs), megakaryocyte–erythrocyte progenitors and common lymphoid progenitors, which are typically observed only in the CD38^+^ compartment at homeostasis (Extended Data Fig. 3a). This suggests that immunophenotypic early human progenitors exhibit some transcriptional plasticity probably induced by xenotransplantation.

Two populations were observed among cells classified as HSCs across the three conditions, denoted HSC-I and HSC-II (Fig. 2b and Extended Data Fig. 3b,c). Separate MPP and lymphomyeloid primed progenitor (LMPP) populations contiguous with HSC-II were identified, designated as MPP-II and LMPP-II, respectively. The HSC identity of both HSC-I and HSC-II was independently confirmed by: expression of stem cell-specific markers^14,19,38^, LT-HSC-specific transcriptional features relative to downstream MPP and LMPP populations^14,19,38^ (Fig. 2c, Extended Data Fig. 3d–h and Supplementary Table 7), chromatin accessibility^19^ (Fig. 2d and Supplementary Table 1) and with transcriptomes matching an HSC reference^32^ (Extended Data Fig. 3i).

**Extended Data Fig. 4:**
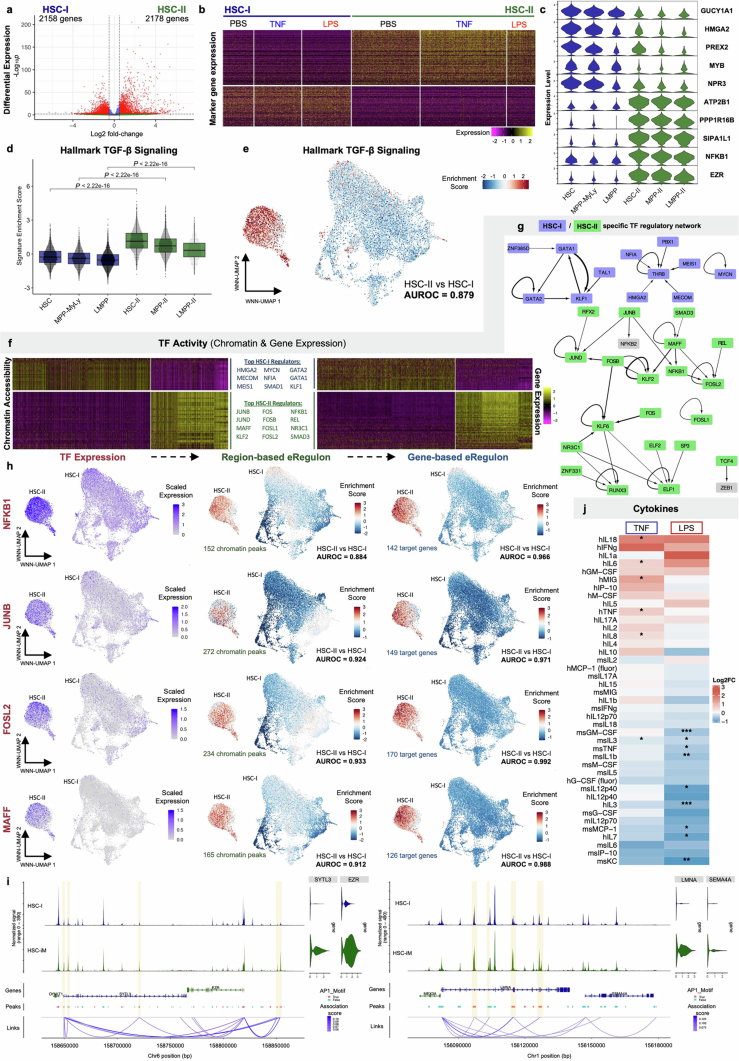
Epigenetic and transcriptional characterization of HSC-I and HSC-II. **a**,**b**, Differentially expressed (DE) genes between HSC-II and HSC-I, identified through pseudo-bulk analysis. **c**, Normalized expression of top HSC-I and HSC-II markers within HSC, MPP, LMPP subsets. **d**,**e**, Normalized enrichment score of the TGF-β signaling pathway across HSC (HSC-I n = 3,830; HSC-II n = 2,146), MPP (MPP-MyLy n = 2,779; MPP-II n = 2,317), and LMPP (LMPP-I n = 5,148; LMPP-II n = 539) subsets (d) and on the WNN UMAP embedding (e). Boxplots show interquartile range with a line at the median and whiskers show minimum and maximum values; statistical comparisons were made with two-sided Wilcoxon rank sum tests. **f-i**, Enrichment of transcription factor (TF) activity in HSC-I or HSC-II based on target chromatin accessibility and gene expression inferred using SCENIC+. TFs in rows are matched in both heatmaps (f). Top regulators for each HSC state are also indicated. TF regulatory networks (g) specific to either HSC-I or HSC-II are shown; colored boxes indicate TFs with significant enrichment by both target gene expression and target chromatin accessibility. Regulation strength between TFs is represented by edge width. TF activity, namely TF RNA expression and chromatin- and gene-based eRegulon scores for NFKB1, JUNB, FOSL2 and MAFF (h) are overlaid on xenograft WNN UMAPs. AUROC scores for discriminatory power between HSC-I and HSC-II are also shown for each TF activity. Examples of chromatin regions along select HSC-iM marker genes for which *cis*-regulatory relationships could be inferred are shown (i). The expression of these genes in HSC-I and HSC-iM are shown as violin plots on the right-hand side. Chromatin accessibility in the vicinity of each gene is visualized for the HSC-I and HSC-iM populations. Differentially accessible peaks at FDR < 0.01 are highlighted with yellow bands. Peaks containing AP1 motifs are also indicated in each plot. Finally, Peak-to-Gene linkages from Signac are shown, which infer *cis*-regulatory relationships between chromatin peaks and target gene expression. **j**, Human and mouse cytokines were measured using a multiplex assay in blood plasma from a subset (n = 9 mice in PBS and LPS groups, and n = 10 in the TNF group across 2 independent CB pools) of the xenografted mice that recovered from TNF, LPS or PBS control repeat challenge. Results are summarized as a heatmap showing fold-changes compared to the PBS group. Data were compared using pairwise two-sided Mann-Whitney tests, *** *p* < 0.001, ** *p* < 0.01, * *p* < 0.05 and *p* > 0.05 is not marked. Source data

By contrast, comparison of HSC-II against HSC-I revealed 3,663 differentially accessible chromatin regions and 2,178 differentially expressed genes (DEGs) upregulated in HSC-II (Extended Data Fig. 4a–c and Supplementary Tables 8 and 9). Transcriptionally, HSC-II was specifically enriched for LT-HSC quiescence (Fig. 2e), TNF via NF-κB signalling (Fig. 2f) and TGFβ signalling (Extended Data Fig. 4d,e) programs. Next, SCENIC+^39^ was used to integrate gene expression and chromatin accessibility data into transcription factor ‘eRegulons’, enabling the identification of key transcription factors and gene regulatory networks (GRNs; Extended Data Fig. 4f,g and Supplementary Table 10). HSC-I GRNs included well-known stemness regulators and regulators of megakaryocyte–erythroid fate^40^, whereas HSC-II GRNs were governed by the inflammation-related transcription factors *NFKB1* and *REL*, AP-1 family members such as *JUNB* and *FOS*, and a TGFβ signalling mediator *SMAD3* (Extended Data Fig. 4f–h). As one HSC-II-specific example, inferred *cis*-regulatory activity of AP-1 family transcription factors is shown (Extended Data Fig. 4i). Multiple complementary analyses showed that HSC-I and HSC-II represent distinct transcriptional and epigenetic states (Supplementary Note 2). Collectively, these data provide strong evidence that the two human HSC populations within the inflammation–recovery model represent transcriptionally and epigenetically distinct states, with HSC-II distinguished primarily by enrichment of inflammatory stress programs.

## HSC-II retain memory of inflammation

Mouse epithelial stem cells and HSCs retain molecular changes after inflammatory treatments^41,42^. We therefore asked whether inflammatory memory was imparted onto human HSCs in our experimental model. Plasma profiling revealed significant differences in several human pro-inflammatory cytokines in xenografts after recovery from challenge with TNF or LPS relative to PBS controls (Extended Data Fig. 4j and Supplementary Table 11), suggesting sustained changes in human HSPCs and their progeny. Indeed, we observed a more pronounced transcriptional and epigenetic distinction between HSC-I and HSC-II following TNF or LPS treatments compared with PBS-treated controls (Fig. 2g,h). Specifically, the activity of 30 transcription factors was increased in HSC-II after recovery from TNF compared with controls, including *NFKB1* and *JUNB* (Fig. 2i–k and Supplementary Table 12); no transcriptional activity was meaningfully altered in HSC-I (Fig. 2i). Similarly, the activity of 28 transcription factors was increased in HSC-II after LPS recovery, including *HMGA1* and *SPI1* (Fig. 2l–n and Supplementary Table 12); changes in HSC-I were modest but included upregulated *HOXA10*, *RXRA* and *CTCF* activity (Fig. 2l–n and Supplementary Table 12). Distinct transcription factors were implicated after TNF and LPS challenge, suggesting that HSC-II show stimulus-dependent epigenetic and transcriptional changes. In summary, durable molecular changes in HSCs following recovery from inflammation occurred predominantly within the HSC-II compartment, suggesting that HSC-II are preferentially impacted by, and retain memory of, previous inflammatory stress. Given these findings, we hereafter refer to HSC-II as HSC-inflammatory memory (HSC-iM)^41,43^.

**Extended Data Fig. 5:**
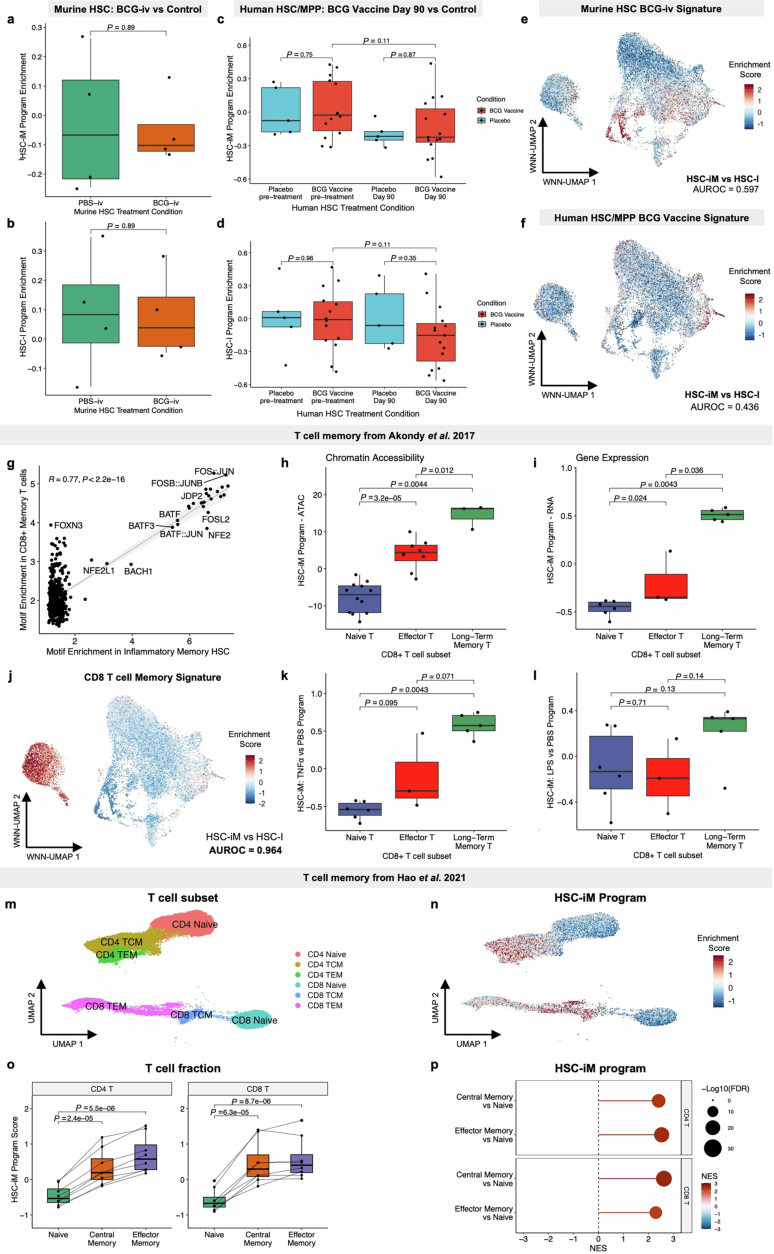
Shared features between inflammatory memory in HSC-iM and immune memory in T-cells. **a**,**b**, Enrichment of HSC-iM (a) vs HSC-I (b) programs in mouse HSCs (LSK + CD150 +) treated with intravenous BCG vaccine or PBS (n = 4 mice/group). Enrichment scores were calculated in bulk RNA-seq data by GSVA. Human marker gene names were converted to mouse orthologs using the babelgene R package. Boxplots show interquartile range with a line at the median and whiskers showing range. Statistical comparisons were made with two-sided Wilcoxon rank sum tests. **c**,**d**, Enrichment of HSC-iM (c) vs HSC-I (d) programs within HSC/MPPs (CD34 + CD38 − CD45RA−) from human volunteers receiving either a BCG vaccine (n = 15 participants) or placebo (n = 5 participants). Samples were collected pre-vaccination and day 90 post-vaccination. Enrichment scores were calculated in bulk RNA-seq data by GSVA. Data are shown and compared as in (a,b). **e**,**f**, Enrichment of a mouse HSC BCG signature consisting of 490 genes, converted to human orthologs using the babelgene R package (e), and of a human HSC/MPP BCG vaccination signature consisting of 206 genes (f) is projected onto the xenograft scMultiome dataset. Enrichment scores were calculated in scRNA-seq data by AUCell. AUROC for separability between HSC-I and HSC-iM is shown. **g**, Pearson correlation of TF motif enrichment among differentially accessible regions (DARs) specific to memory T cells from Akondy et al.^47^ vs in HSC-iM; p-value is derived from a two-sided t-distribution. **h**,**i**, Enrichment of chromatin accessibility-based (h) or transcript-based (i) programs specific to HSC-iM (vs HSC-I) was evaluated within human T cell subsets; n = 12, n = 8 and n = 3 donors were subjected to ATACseq, shown in (h), and n = 6, n = 3 and n = 5 to RNAseq, shown in (i) for Naïve, Effector and memory T, respectively. Data are shown and compared as in (a,b). **j**, Scoring of a CD8 long-term T cell memory signature in xenograft scMultiome data. AUROC score for discriminatory power between HSC-I and HSC-iM is also shown. **k**,**l**, Enrichment scores of a TNF-specific (vs PBS) program (23 genes) (k) or LPS-specific program (177 genes) (l) from HSC-iM in functionally-defined CD8 T cell subsets, quantified by GSVA. Data are presented and compared as in (a,b), and number of donors are as in (i). **m**, Integrated UMAP embedding of human PBMC CITE-seq from Hao et al.^49^, incorporating gene expression and immunophenotype. Only Naïve, Central Memory T (TCM), and Effector Memory T (TEM) CD4 and CD8 T cells are shown. **n**, HSC-iM program enrichment in human T cell data, represented by the scaled AUCell enrichment score. **o**, HSC-iM program enrichment in human T cell subsets at the level of individual donors (n = 8). Scaled AUCell enrichment scores were averaged across cells in each subset within each human donor; subsets from each donor are connected by a line. Boxplots are as in (a,b); data were compared using two-sided paired Student’s t-tests. **p**, GSEA results depicting HSC program enrichment in differential expression results between memory and Naïve T cells, utilizing pseudo-bulk profiles and adjusting for donor ID as a covariate. Positive scores indicate enrichment in memory T cell subsets compared to Naïve. Source data

To compare HSC-iM to immunological memory in other contexts, HSC-iM-specific programs composed of 3,663 differentially accessible chromatin regions (Supplementary Table 8) or the top 200 marker genes (Supplementary Tables 9 and 12) were defined. HSC-iM is distinct from ‘trained’ immunity programs associated with BCG vaccination in mouse^42^ or human^44^ settings (Extended Data Fig. 5a–f and Supplementary Table 13). AP-1 family transcription factors, which are associated with the HSC-iM state, are known to have a key role in T cell memory^45,46^. Data from naive (naive T), effector (T_eff_) and memory (T_M_) CD8 T cells from volunteers after vaccination^47^ revealed shared chromatin motif enrichment patterns between T_M_ cells and HSC-iM (Extended Data Fig. 5g). Specifically, AP-1 family transcription factor-binding sites defined by a core 7-bp motif — 5′-TGAG/CTCA-3′ — were highly enriched in accessible chromatin regions specific to both HSC-iM and T_M_ cells, whereas motifs for T cell-specific regulators^48^ were unique to T_M_ cells. There was greater enrichment of HSC-iM chromatin and transcriptional programs in T_M_ cells than in T_eff_ and naive T cells (Extended Data Fig. 5h,i). Reciprocally, a T_M_ transcriptional program, composed of 257 genes upregulated in T_M_ cells (versus naive T and T_eff_ cells; Supplementary Table 13), was specifically enriched in HSC-iM with high discriminatory power (Extended Data Fig. 5j). Of note, treatment-specific memory programs retained by HSC-iM were also enriched in T_M_ cells (Extended Data Fig. 5k,l). The HSC-iM program was also enriched in CD4 and CD8 T_M_ cells from another dataset^49^ (Extended Data Fig. 5m–p). Together, HSC-iM harbours inflammatory memory programs convergent with those in functionally defined human T cell memory.

## HSC-iM in pathophysiological contexts

**Fig. 3:**
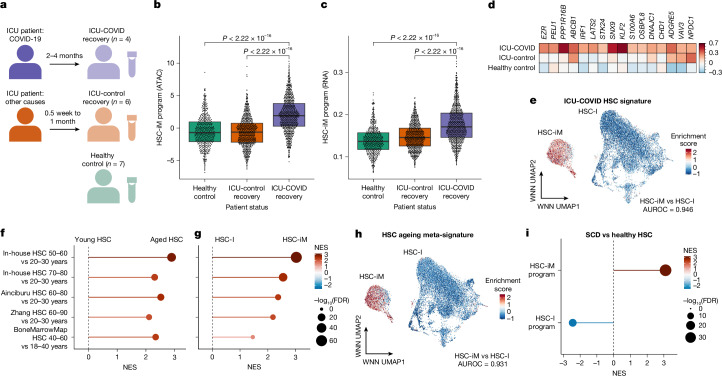
HSC inflammatory memory is induced by severe infection and is upregulated with age and SCD. **a**, Experimental setup of scMultiome profiling of HSPCs from donors 2–4 months after severe COVID-19 infection, as well as ICU recovery controls and healthy donors^50^. **b**,**c**, Enrichment of the HSC-iM program within human HSCs (*n* = 490, *n* = 747 and *n* = 812 for healthy, ICU-control and ICU-COVID groups, respectively) from donors in **a**, scored on the basis of chromatin accessibility (**b**) and gene expression (**c**). The boxplots depict the interquartile range with a line at the median, and the whiskers show the range; data are compared using two-sided Wilcoxon rank-sum tests. **d**, Mean normalized expression of 20 DEGs comprising a post-COVID HSC signature upregulated in HSCs from ICU-COVID donors compared with healthy controls and ICU-controls at FDR < 0.05. **e**, Projection of the post-COVID HSC signature onto the xenograft scMultiome. The area under the receiver-operator curve (AUROC) score for discriminatory power between HSC-I and HSC-iM is also shown. **f**, GSEA results depicting HSC-iM gene expression program enrichment in differential expression results between five separate comparisons of older-age (60–90 years) or middle-aged (40–60 years) versus young adult (aged 18–40 years) human HSCs. **g**, GSEA results depicting enrichment of individual aged HSC signatures from datasets in **f** in xenograft HSC-iM versus HSC-I. The *y* axis is as in **f**. **h**, Human HSC ageing datasets from **f** were integrated to derive a 37-gene aged HSC-specific meta-signature, which was scored in the xenograft scMultiome. The AUROC score for discriminatory power between HSC-I and HSC-iM is indicated. **i**, GSEA results depicting enrichment of HSC-I and HSC-iM signatures in patients with SCD versus healthy donors. Source data

**Extended Data Fig. 6:**
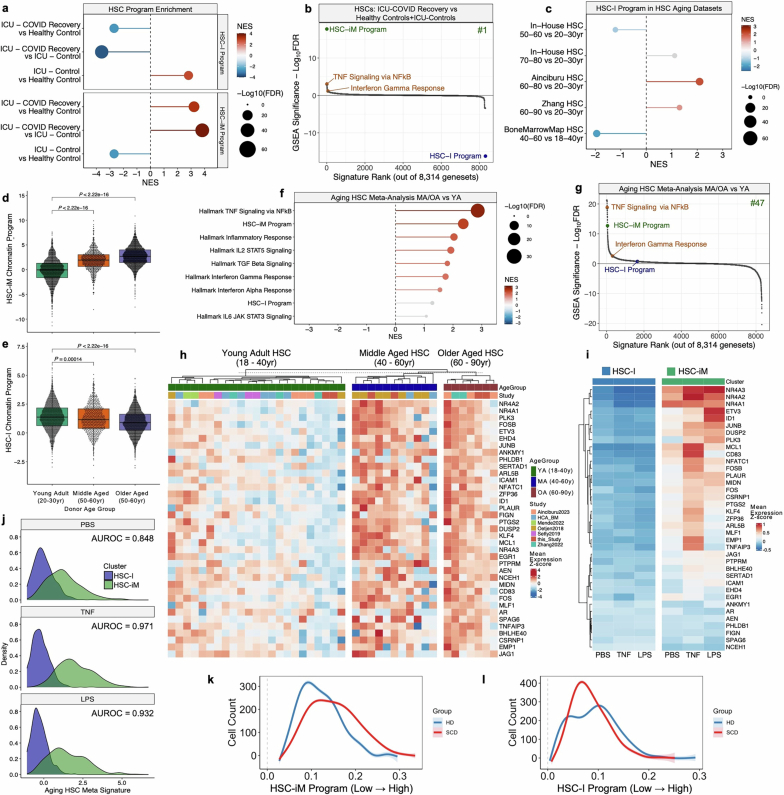
Identification of HSC-iM within HSCs following severe COVID-19 illness, across ageing, and in sickle cell patients. **a**, GSEA results depicting enrichment of HSC-I- and HSC-iM-specific gene expression programs in HSCs from Healthy Controls, ICU Controls, and Severe COVID-19 Recovery. **b**, Benchmarking of GSEA results from HSC-iM and HSC-I programs against signatures from MSigDB, totaling 8,314 gene sets. The enrichment rank of the HSC-iM program is depicted in the top right corner of each comparison. **c**, GSEA results depicting HSC-I gene expression program enrichment in differential expression results between five separate comparisons of older aged (OA) and middle aged (MA) vs young adult (YA) human HSC. **d**,**e**, ChromVAR enrichment scores of accessible chromatin peaks specific to HSC-iM (d) and HSC-I (e) subsets in scATAC-seq from YA (n = 2,531 cells), MA (n = 1,273 cells), and OA (n = 2,828 cells) HSCs within the in-house BM scMultiome dataset. Boxplots depict interquartile range with a line at the median and whiskers show range; data are compared using two-sided Wilcoxon rank-sum tests. **f**, GSEA enrichment of HSC programs and hallmark pathways in ageing HSCs compared to HSCs from young adults. **g**, Benchmarking of GSEA results comparing HSC-iM and HSC-I program enrichment against signatures from MSigDB, totaling 8,314 gene sets. The enrichment rank of the HSC-iM program is depicted in the top right corner of each comparison. **h**, Expression profile of 37 shared genes commonly upregulated in aged HSCs through meta-analysis of independent HSC ageing datasets. Each column is a pseudo-bulk profile of HSCs within an individual donor from each ageing study. **i**, Expression of 37 shared HSC ageing genes within HSC-I and HSC-iM in each treatment condition. **j**, Enrichment scores of the 37-gene HSC ageing meta-signature between HSC-I and HSC-iM, stratified by treatment condition. **k**,**l**, Density plots depicting HSC-iM (k) or HSC-I (l) program enrichment scored by AUCell in healthy donors and patients with sickle cell disease. Source data

We assessed the relevance of the xenograft-derived HSC-iM program to physiological health and disease scenarios. First, there was stronger enrichment of the HSC-iM program in 3,759 peripheral blood HSCs collected from patients 2–4 months following intensive care unit (ICU) admission for severe COVID-19 (ICU-COVID) versus ICU admission for other reasons (ICU-control) or healthy donors (healthy control) by both chromatin accessibility and gene expression^50^ (Fig. 3a–c and Extended Data Fig. 6a). Gene set enrichment analysis (GSEA) was then used to benchmark the HSC-iM program against 8,312 pathways or gene sets; enrichment of the HSC-iM program in ICU-COVID HSC surpassed all others by statistical significance (Extended Data Fig. 6b). Moreover, a post-COVID HSC transcriptional program comprising 20 DEGs unique to ICU-COVID HSCs (Fig. 3d and Supplementary Table 13) was specifically enriched within the HSC-iM population (Fig. 3e). Thus, the HSC-iM program is enriched in HSCs sampled after recovery from severe inflammation in patients with COVID-19 infection.

Infections occur throughout the human lifespan, and physiological ageing is associated with chronic, low-grade inflammation (‘inflammageing’)^51^. We therefore curated 23,048 HSC transcriptomes from 4 distinct bone marrow cohorts (*n* = 41 donors; 19–87 years of age)^32,52–54^. The HSC-iM program was significantly enriched in HSCs from older-aged (60–90 years of age) or middle-aged (40–60 years of age) donors compared with young adult (18–40 years of age) donors (Fig. 3f) unlike HSC-I (Extended Data Fig. 6c). DEGs upregulated in aged HSCs (Supplementary Table 14) were also significantly enriched within HSC-iM (Fig. 3g). Similarly, HSCs from middle-aged and older-aged donors had stronger enrichment for the HSC-iM chromatin program than young adult donors (Extended Data Fig. 6d,e). GSEA benchmarking as above revealed enrichment of the HSC-iM program surpassing 99.4% of other tested gene sets in HSCs from middle-aged and older-aged donors versus young adult donors (Extended Data Fig. 6f,g). Next, we defined a human HSC ageing-related meta-signature of 37 genes (see Methods), including *NR4A1*, which links to inflammation resistance in CH^55^ (Extended Data Fig. 6h and Supplementary Table 14). This meta-signature was enriched within HSC-iM (Fig. 3h); greater enrichment was observed in theTNF and LPS groups (Extended Data Fig. 6i,j). Finally, the HSC-iM program was significantly enriched in HSCs from paediatric patients with sickle cell disease (SCD)^56^ (Fig. 3i), indicating that HSC-iM could be associated with inflammation-related premature HSC ageing phenotypes as opposed to ‘physiological’ ageing alone. Furthermore, there was variable expression of HSC-iM within SCD HSCs (Extended Data Fig. 6k,l), concordant with SCD heterogeneity^57^. Together, the HSC-iM transcriptional signature accumulates after inflammatory stress following infection, ageing and SCD.

## HSC-iM in CH

**Fig. 4:**
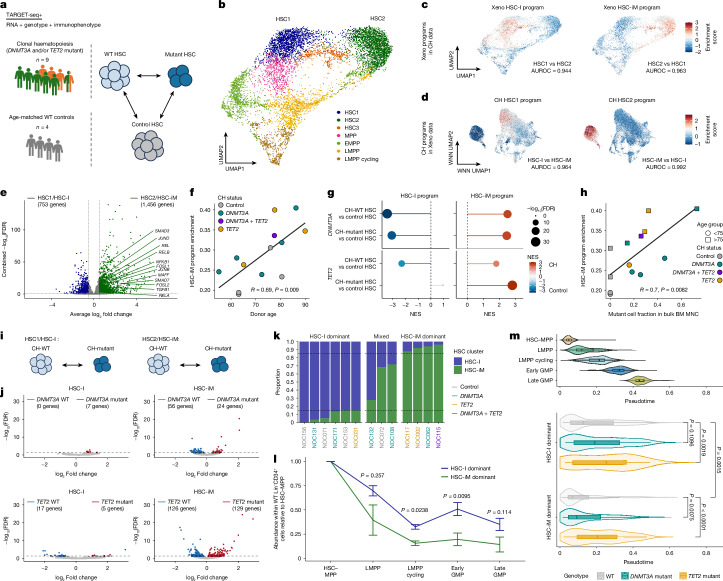
CH mutations preferentially affect HSC-iM to promote differentiation. **a**, TARGET-seq+ dataset of bone marrow HSPCs. **b**, UMAP of 8,059 HSPCs from donors in **a**. **c**,**d**, Xenograft HSC-I and HSC-iM program enrichment in bone marrow HSPCs (**c**) and vice versa (**d**). The AUROC score between populations is shown. **e**, Volcano plot of common DEGs between xenograft (HSC-iM versus HSC-I) and bone marrow (HSC2 versus HSC1) datasets. For each gene, the average log_2_-transformed fold change and the least significant FDR-corrected *P* value was used. Discordant DEGs were not plotted. **f**, Pearson correlation between HSC-iM program enrichment within HSCs and age (*n* = 13 donors); *P* values were derived from a two-sided *t*-distribution. **g**, HSC-I and HSC-iM program enrichment by GSEA between control, CH-WT and CH-mutant HSC–MPPs, after adjusting for multiple covariates. **h**, Pearson correlation between HSC-iM program enrichment and mutant cell fraction in mononuclear cells (MNCs); *P* values were derived from a two-sided *t*-distribution. **i**,**j** Differential expression analysis between CH-mutant and CH-WT cells within HSC1/HSC-I or HSC2/HSC-iM populations (**i**); significant (FDR < 0.05; the cut-off is shown as a horizontal dashed line) DEGs (**j**) are coloured. **k**, Proportion of HSC2/HSC-iM versus HSC1/HSC-I within each bone marrow sample. **l**, Relative abundance of HSPC populations among WT cells from HSC-I-dominant and HSC-iM-dominant hierarchies (*n* = 6 and *n* = 4 samples, respectively), normalized to HSC–MPP abundance. Data are presented as mean ± s.e.m. Data were compared using a two-sided Wilcoxon rank-sum test. **m**, Effect of *DNMT3A* and *TET2* mutations on lineage commitment within HSC-I-dominant and HSC-iM-dominant hierarchies. Pseudotime distributions are shown for each population along myeloid differentiation, and for CH-WT and CH-mutant cells from HSC-I-dominant (*n* = 1,576 WT, *n* = 125 *DNMT3A* mutant and *n* = 61 *TET2* mutant) and HSC-iM-dominant (*n* = 596 WT, *n* = 202 *DNMT3A* mutant and *n* = 290 *TET2* mutant) hierarchies. The boxplots display the median and interquartile range. Holm–Bonferroni-adjusted *P* values were calculated by likelihood ratio tests of a linear mixed model accounting for sample effects. Source data

**Extended Data Fig. 7:**
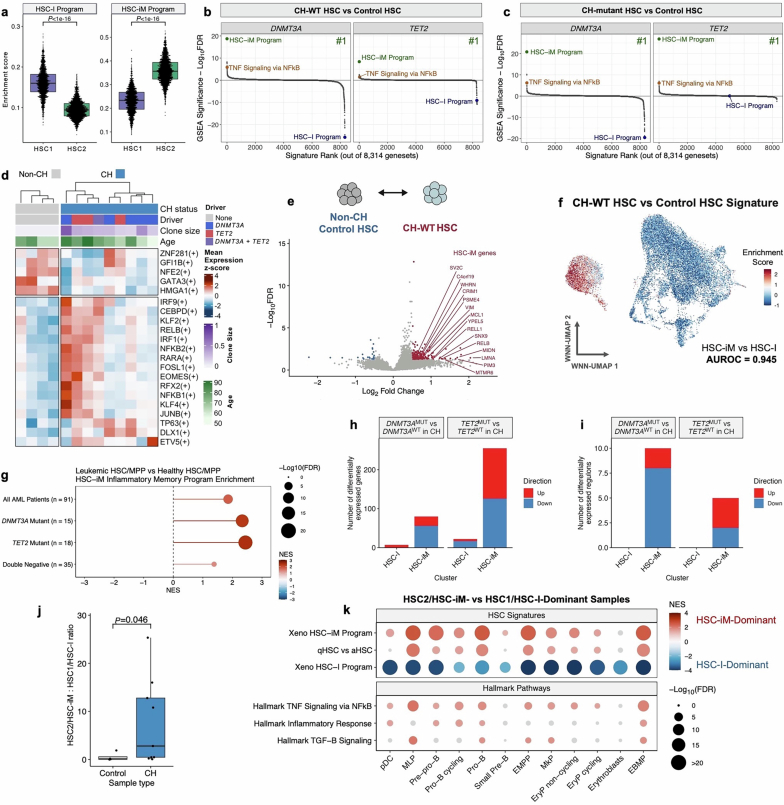
Identification of HSC-iM within human bone marrow samples with clonal hematopoiesis. **a**, Enrichment of the HSC-iM program within the HSC1 (n = 1,701 cells) and HSC2 (n = 2,150 cells) populations identified in the TARGET-seq+ dataset of CH and non-CH donors. Boxplots display the median and interquartile range. Statistical comparisons were made using two-sided Wilcoxon rank sum tests. **b**,**c** Benchmarking of GSEA results from differential expression comparisons of HSC/MPPs between CH-WT versus Control (b) and CH-mutant versus Control (c) after adjusting for multiple covariates. Enrichment scores for HSC-iM and HSC-I programs were compared against signatures from MSigDB, totaling 8,314 gene sets. The enrichment rank of the HSC-iM program is depicted in the top right corner of each comparison. Results are faceted by CH mutation type. **d**, Differential expression of regulons (with FDR < 0.1) between CH-WT HSCs and HSCs from non-CH donors, performed by linear mixed model adjusting for donor ID, donor age, donor sex, and FACS sort batch. Each column represents a pseudo-bulk from one donor. Donors are annotated with their CH status, driver mutation, the clone size in bulk BM sequencing and their age. **e**, Differentially expressed genes (DEGs) between CH-WT HSCs from 9 donors and control HSCs from 4 non-CH donors, performed by ‘dream’ linear mixed model adjusting for donor ID, donor age, donor sex, and FACS sort batch. Significant genes were called with FDR < 0.05 and log_2_FC > 0.5. DEGs in the HSC-iM program are labeled. **f**, Enrichment of the CH-WT HSC versus Control HSC signature from (e) within the xenograft scMultiome dataset. Enrichment scores were calculated in scRNA-seq data by AUCell. **g**, GSEA of HSC-iM in acute myeloid leukemia samples (n = 91) stratified by subtype compared to healthy controls (n = 16). **h**, Number of differentially expressed genes between CH-mutant and CH-WT cells within the HSC1 and HSC2 clusters. Significant genes were called with FDR < 0.05. **i**, Number of differentially expressed regulons between CH-mutant and CH-WT cells within the HSC1 and HSC2 clusters. TF activity was inferred by SCENIC. **j**, Ratio of HSC2/HSC-iM to HSC1/HSC-I cells in donors with (n = 9) or without (n = 4) CH. Boxplots display the median and interquartile range and whiskers show range. Statistical comparison was made using a two-sided Student’s *t-*test. **k**, GSEA results depicting enrichment of HSC programs and hallmark signatures in each cell type between HSC2/HSC-iM-dominant hierarchies vs HSC1/HSC-I-dominant hierarchies. Source data

Similar to xenograft-derived HSC-iM programs, CH is associated with ageing, inflammation^13^ and elevated inflammatory cytokines^26^. We utilized TARGET-seq+ data of bone marrow HSPCs^52^ (Fig. 4a) to investigate HSC-iM in natural cases of CH. Two main clusters of human HSC were previously identified: HSC1 and HSC2 (Fig. 4b); HSC2 were co-enriched in quiescence and TNF via NF-κB signalling programs^52^. The xenograft-derived HSC-I and HSC-iM programs enabled strong separation of bone marrow-derived HSC1 and HSC2 (Fig. 4c), and vice versa (Fig. 4d, Extended Data Fig. 7a and Supplementary Table 15). Of note, HSC2 and HSC-iM shared 1,456 significantly upregulated genes relative to HSC1 and HSC-I, respectively (Fig. 4e and Supplementary Table 16), including key AP-1 and NF-κB transcription factors and mediators of TGFβ signalling. These data provide crucial evidence that the human bone marrow counterparts for the xenograft-derived HSC-I and HSC-iM are HSC1 and HSC2, hereafter also referred to as HSC-I and HSC-iM.

Next, we compared mutant (CH-mutant) and wild-type (CH-WT) HSCs from individuals with *DNMT3A* or *TET2* mutations to HSCs from age-matched control non-CH individuals. The enrichment of the xenograft HSC-iM program in 4,651 HSC transcriptomes correlated well with donor age (Fig. 4f). Unexpectedly, both CH-WT and CH-mutant HSCs were significantly enriched for the HSC-iM program and depleted for HSC-I compared with control non-CH HSCs, even after adjusting for donor, age, sex and cell-sorting batch as covariates (Fig. 4g). GSEA benchmarking revealed that HSC-iM program enrichment surpassed all tested gene sets when comparing CH-WT or CH-mutant against non-CH HSCs (Extended Data Fig. 7b,c). Activity of HSC-iM-associated transcription factors were upregulated in CH-WT HSCs compared with control non-CH HSCs, including *NFKB1* and AP-1 family members (Extended Data Fig. 7d). Moreover, a signature of 219 genes upregulated in CH-WT HSCs (Extended Data Fig. 7e and Supplementary Table 15) was enriched within xenograft-derived HSC-iM (Extended Data Fig. 7f). Collectively, these data identify inflammatory memory as a transcriptional hallmark of both CH-WT and CH-mutant HSCs from *DNMT3A*-mutated and *TET2-*mutated CH donors.

HSC-iM program enrichment within donors also correlated with CH clone size in their bulk DNA sequencing data (Fig. 4h). An increased clone size in CH is predictive of acute myeloid leukaemia onset up to a decade later^23,24^. Accordingly, we found HSC-iM enrichment in acute myeloid leukaemia compared with healthy controls, which was most pronounced in samples with *TET2* or *DNMT3A* mutations (Extended Data Fig. 7g). These data suggest a mutation-dependent, complex relationship between inflammatory memory in HSCs, clonal expansion and evolution to malignancy.

## CH impacts HSC-iM differentiation

We next investigated CH-mutant and CH-WT HSC within each bone marrow-derived HSC subset (Fig. 4i) and found that mutation-associated gene expression changes occur predominantly within HSC-iM (Fig. 4j, Extended Data Fig. 7h and Supplementary Table 17). Dysregulated transcription factor activity in CH-mutant versus CH-WT was exclusively found in HSC-iM (Extended Data Fig. 7i). This indicates that, at a transcriptional level, CH mutations preferentially affect the HSC-iM transcriptional state.

Overall, we found that the ratio of HSC-iM to HSC-I cells to be higher among CH donors than non-CH controls (Extended Data Fig. 7j). There was also a polarization (more than 85% dominance) towards one HSC state in ten samples — six HSC-I dominant samples and four HSC-iM dominant samples — and three samples showed a mixed pattern (Fig. 4k). We reasoned that most progenitors and downstream myeloid progeny would originate from the dominant HSC subset in these samples. To interrogate how CH mutations might affect HSC-I and HSC-iM output, the myeloid differentiation patterns from each HSC subset were stratified by mutation status with pseudotime analysis. Within CH-WT hierarchies, HSC-iM-dominant samples had reduced frequencies of LMPP and GMP progenitors relative to HSC–MPP when compared with HSC-I-dominant samples (Fig. 4l,m). This suggests that HSC-iM may have reduced differentiation compared with HSC-I, in keeping with enrichment of quiescence programs. By contrast, the HSC-iM-dominant samples had significantly increased differentiation from HSC–MPP within CH-mutant cells compared with CH-WT cells (Fig. 4m). This finding is consistent with selective downregulation of quiescent HSC programs and upregulation of progenitor programs induced by CH mutations within HSC-iM^52^. Thus, transcriptional dysregulation associated with CH mutations occurs preferentially within the HSC-iM state and is characterized by attenuation of a baseline differentiation impairment, probably leading to increased myelopoiesis.

**Extended Data Fig. 8:**
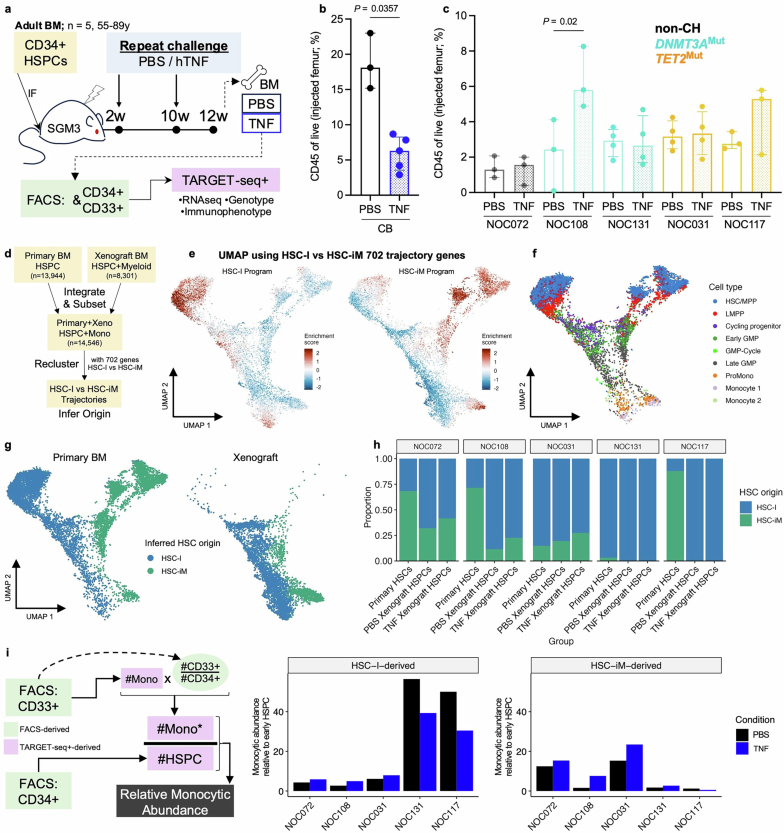
Hematopoietic output from human bone marrow HSPCs in an inflammation recovery model. **a**, Schematic for modeling inflammatory challenge with TNF in the NSG-SGM3 mouse model. **b**, Human engraftment from a single CB donor (80,000 cells injected/mouse) is decreased following repeated TNF challenge (n = 5 mice) compared to PBS controls (n = 3 mice) at 12w post-transplant. Bars depict median and error bars show interquartile range; data were compared with a two-sided Mann-Whitney test. **c**, Human engraftment after CD34+ HSPCs from bone marrow of 5 individuals with and without CH mutations at 12w post-transplant where the HSC-I and HSC-iM composition was known (NOC072: 77,500 cells/mouse, n = 3 mice/group; NOC108: 55,800 cells injected/mouse, n = 3 mice/group; NOC131: 90,600 cells/mouse, n = 4 mice/group; NOC031: 80,600 cells/mouse, n = 4 mice/group; NOC117: 55,800 cells/mouse, n = 3 mice/group). Bars depict median and error bars show interquartile range. Data were compared using a Kruskal-Wallis test with donor-wise treatment pairs preselected for posthoc comparison using an uncorrected Dunn’s test; p < 0.1 is shown. **d**, Strategy for integrating the 8,301 cells (CD34+ HSPCs and CD33+ myeloid cells) isolated from xenografts in (c) with 13,944 primary HSPCs from the original TARGET-seq+ dataset to allow inference of HSC-I vs HSC-iM origin (See Supplementary Note 3 for the complete methods and QC). **e**, UMAP plots generated using HSC-I vs HSC-iM trajectory genes showing enrichment of the HSC-I and HSC-iM gene programs. **f**, UMAP showing cell type annotations of HSPCs and monocytic lineage cells used for subclustering with HSC-I vs HSC-iM trajectory genes. **g**, UMAP showing annotation of the inferred HSC origin of cells from primary BM and xenograft BM. **h**, Proportion of HSC-I vs HSC-iM-derived cells within the HSCs at homeostasis and within the CD34+ HSPCs isolated from xenograft recipients treated with PBS or TNF. **i**, Abundance of monocytic cells (Late GMP, ProMono, and Monocytes) relative to early HSPC populations among HSC-I vs HSC-iM-derived cells, calculated as shown in the schematic, in the xenograft BM samples treated with either PBS or TNF. Source data

To experimentally validate the contribution that HSC-I and HSC-iM make to downstream progeny, we transplanted bone marrow samples with known HSC-I and HSC-iM composition into NSG-SGM3-immunodeficient mice, which allow for better myeloid differentiation than NSG (Extended Data Fig. 8a). Engraftment of a single CB donor enabled us to bridge previous findings from modelling in NSG and NSGW41 mice to NSG-SGM3, where TNF treatment still reduced graft size (Extended Data Fig. 8b). Conversely, TNF treatment significantly increased total graft size in NOC108, a *DNMT3A*-mutant sample that is mixed for HSC-I and HSC-iM, but not in any of the other samples (Extended Data Fig. 8c). TARGET-seq+ was performed on CD34^+^ HSPCs and CD33^+^ progeny isolated from the xenografts, and the data were integrated with that from the primary samples to infer the HSC of origin of xenograft-derived progeny (Extended Data Fig. 8d–g and Supplementary Note 3). Both HSC-I-derived and HSC-iM-derived cells were identified in xenografts with variable representation of HSC-I and HSC-iM frequencies compared with the primary sample (Extended Data Fig. 8h). Two samples (NOC131 and NOC117) showed dominance of HSC-I-derived HSPCs, and the output of monocytic progeny was greater in these samples than in the remaining three samples with balanced contribution from HSC-I and HSC-iM (Extended Data Fig. 8i). These data support our observation that HSC-iM generate fewer myeloid progeny than HSC-I and suggest that the two HSC subsets may respond differently to inflammation.

## Mature progeny retain features of HSC-iM

**Fig. 5:**
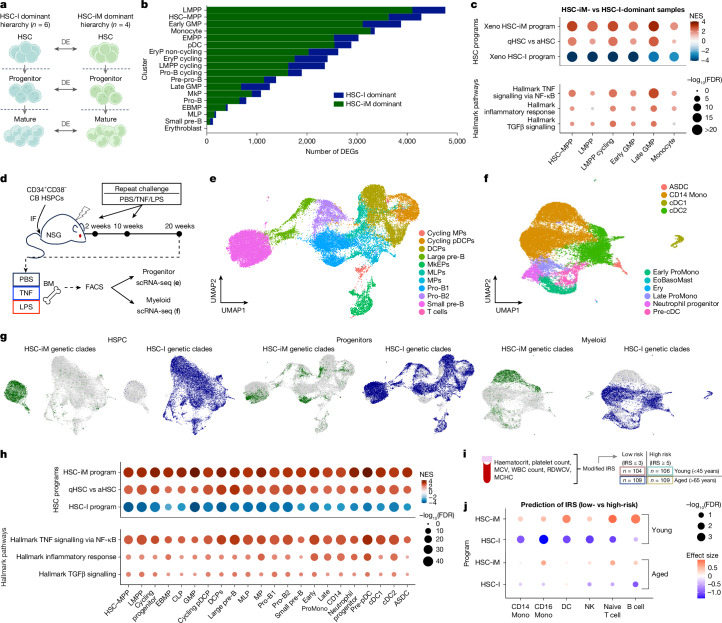
Transmission of inflammatory memory programs from HSC-iM to downstream progeny. **a**, Schematic outlining differential expression (DE) analysis between HSC-I-dominant and HSC-iM-dominant hierarchies within cell types at various stages of differentiation. Schematic created in BioRender; Zeng, A. https://biorender.com/rdr5wvf (2026). **b**, Number of DEGs between HSC-I-dominant and HSC-iM-dominant hierarchies within each cell type. **c**, GSEA results depicting enrichment of HSC and hallmark signatures in HSC-iM-dominant versus HSC-I-dominant hierarchies within each cell type along myeloid differentiation. Positive enrichment scores indicate enrichment in cells from HSC-iM-dominant hierarchies. **d**, Schematic depicting sorting of committed progenitor and myeloid compartments from the xenograft model for scRNA-seq. **e**,**f**, UMAPs of CD34^+^CD38^+^ progenitors (**e**) and CD33^+^ myeloid cells (**f**). ASDC, AXL^+^SIGLEC6^+^ dendritic cells; cDC, conventional dendritic cells; DCP, dendritic cell progenitors; EoBasoMast, eosinophils, basophils and/or mast cells; MkEP, megakaryocyte–erythrocyte progenitors; MkP, megakaryocyte progenitors; MLP, multi-lymphoid progenitors; MP, myeloid progenitors; Mono, monocytes; pDCP, plasmacytoid dendritic cell progenitors; ProMono, promonocytes. **g**, UMAPs of HSPC, progenitor and myeloid cells depicting distribution of HSC-iM-dominant and HSC-I-dominant genetic clades. **h**, GSEA results depicting enrichment of HSC and hallmark signatures in differential expression results between HSC-iM-dominant genetic clades versus HSC-I-dominant genetic clades within HSPCs, downstream progenitors and myeloid populations. Positive enrichment scores indicate enrichment in HSC-iM-dominant genetic clades. **i**, Schematic depicting the calculation of the modified IRS using six blood parameters and the stratification scheme of the Ontario Health Study scRNA-seq cohort by age and IRS. MCHC, mean corpuscular haemoglobin concentration; MCV, mean corpuscular volume; RDWCV, red cell distribution width-coefficient of variation; WBC, white blood cells. **j**, Prediction of low or high IRS using HSC-I and HSC-iM program scores in mature cell pseudobulks using generalized linear models, corrected for batch, sex and age. NK, natural killer. Source data

We next evaluated whether immune progeny derived from either HSC-I or HSC-iM retain any of the molecular features specific to each HSC subset. We performed differential expression analysis between progeny from bone marrow donor samples with HSC-I-dominant or HSC-iM-dominant hierarchies, at each stage of myeloid differentiation (Fig. 5a). The number of significant DEGs between HSC-I-derived versus HSC-iM-derived progeny varied by cell type, with thousands of DEGs observed within LMPPs, GMPs and monocytes (Fig. 5b and Supplementary Table 18). Critically, myeloid populations from HSC-iM-dominant hierarchies were enriched in the HSC-iM program and other inflammatory signalling pathways when compared with their counterparts from HSC-I-dominant hierarchies (Fig. 5c and Extended Data Fig. 7k). Two hypotheses may underlie this phenomenon: direct inheritance from upstream HSCs and/or extrinsic activation due to exposomic differences between donors.

**Extended Data Fig. 9:**
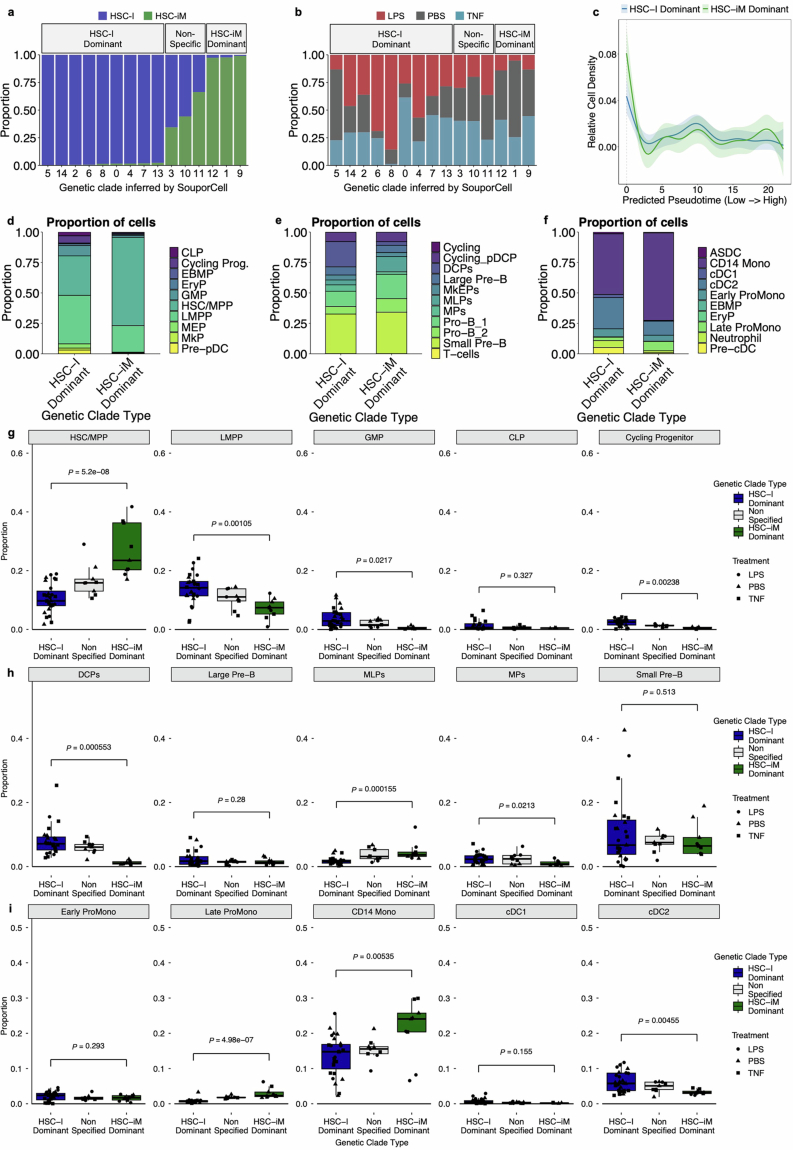
Identification of polymorphism-based genetic clades within human cells from xenografts. **a**,**b** Barplots depicting the proportion of cells derived from HSC-iM or HSC-I (a), or mice treated with PBS, TNF, and LPS (b) in each genetic clade. **c**, Density plot of predicted pseudotime within HSC-I and HSC-iM derived progeny. Shaded area indicates 95% confidence interval. **d-f**, Stacked barplots depicting the proportions of cell types in HSC-iM-dominant, HSC-I-dominant, and non-specific genetic clades within CD34 + CD38 − CD45RA − HSPC (d), CD34 + CD38+ Progenitors (e), and CD33+ (f) datasets. **g-i**, Proportion of each indicated cell type in HSC-I-dominant, non-specified and HSC-iM-dominant clades for HSPCs (g), Progenitors (h) and myeloid (i) subpopulations. Simple linear regression was used to evaluate differences in proportions within genetic clades, with p-values derived from a two-sided uncorrected Student’s t-test with unequal sample size. Boxes represent the interquartile range, center lines indicate median, whiskers extend to 1.5x interquartile range. Points denote proportions from individual clades (n = 9 HSC-I- and n = 3 HSC-iM-dominant, and n = 3 non-specified) within individual conditions (PBS, TNF, LPS) shown with different symbols; total points equally distributed across conditions are n = 27 for HSC-I-dominant, n = 9 for HSC-iM-dominant, and n = 9 for non-specified in HSC/MPP, MLP, MPs, Small Pre-B and cDC2. Some clades do not have enough cells in certain conditions, so proportions were computed with variable numbers of HSC-I-dominant clades (n = 27 for DCPs, n = 26 for LMPP, GMP, Cycling Progenitors, Early ProMono, n = 25 for Large Pre-B, Late ProMono, n = 24 for CD14 Mono, cDC1, and n = 21 for CLP), HSC-iM-dominant clades (n = 9 for LMPP, Large Pre-B, Early ProMono, Late ProMono, n = 7 for DCPs, n = 6 for GMP, n = 5 for Cycling Progenitors, n = 4 for CD14 Mono, cDC1, and n = 3 for CLP), and non-specified clades (n = 9 for all cell types). Source data

**Extended Data Fig. 10:**
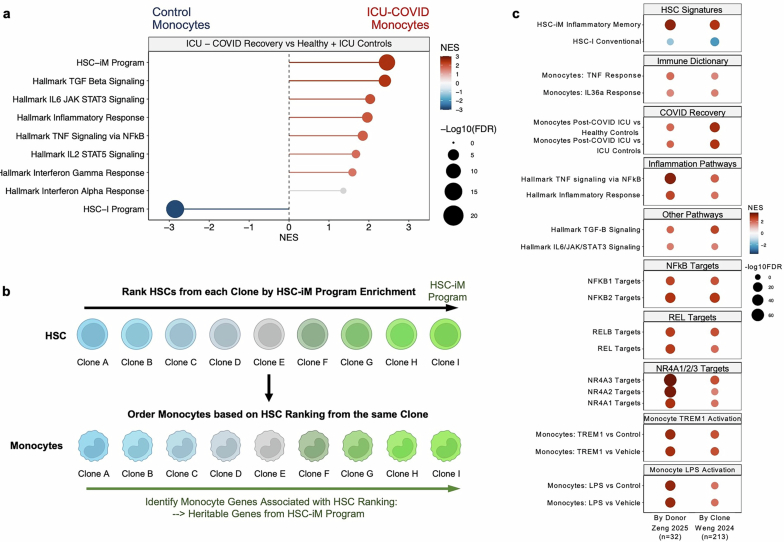
Transmission of HSC-iM programs to monocytes. **a**, GSEA of HSC-iM and HSC-I programs alongside other inflammatory signaling pathways in CD14+ monocytes following severe COVID infection (ICU-COVID) in comparison to healthy controls and ICU-recovery controls. **b**, Schematic for relating HSC-iM program strength in HSCs with transcriptional features of downstream monocytes. Genes were ranked by test statistic from differential expression using HSC-iM program enrichment among HSCs as a continuous variable. Schematic created in BioRender; Zeng, A. https://biorender.com/rdr5wvf (2026). **c**, GSEA of signatures and pathways along monocyte transcriptomes from the analysis of the association between HSC-iM program strength in HSCs with transcriptional features of downstream monocytes across donors from BoneMarrowMap (n = 32 donors) and mtDNA-defined clones (n = 213 clones from n = 13 donors). Source data

To test whether the HSC-iM program could be transmitted to downstream progeny independent of environmental context, we performed single-cell RNA sequencing (scRNA-seq) of CD34^+^CD38^+^-committed progenitors and CD33^+^ myeloid progeny (Fig. 5d–f) from xenografts to complement the HSPC scMultiome (Fig. 2a,b). We leveraged the inherent genetic polymorphisms within the CB donors (*n* = 62) to infer clonal relationships between 82,527 xenograft-derived human cells (Supplementary Note 4). ‘Genetic clades’ were defined and found to be either HSC-iM dominant (*n* = 3), HSC-I dominant (*n* = 9) or nonspecific (*n* = 3), enabling us to link groups of HSCs to their downstream progenitor, lymphoid and myeloid progeny (Extended Data Fig. 9a–c). Of note, HSC-iM-dominant clades exhibited limited contribution to the progenitor pool as compared with HSC-I-dominant clades (Extended Data Fig. 9d–g), which aligns with the findings from primary bone marrow samples (Fig. 4l). Although committed B cell lineages did not show significant bias (Extended Data Fig. 9h and Supplementary Table 19), CD14 monocytic output was proportionally higher in HSC-iM-dominant clades than HSC-I-dominant clades (Extended Data Fig. 9i). Differential expression between HSC-iM-derived versus HSC-I-derived progeny showed consistent enrichment of the HSC-iM program and other inflammatory signalling pathways alongside depletion of the HSC-I program (Fig. 5g,h and Supplementary Table 20). We obtained further evidence for transmission of pro-inflammatory signalling programs from HSC-iM to mature monocytes in the ICU-COVID dataset (Extended Data Fig. 10a), in 32 healthy donors from BoneMarrowMap^32^ across lifespan (Extended Data Fig. 10b,c), and across 213 genetic clones spanning 13 donors identified by mitochondrial DNA-based lineage tracing^8^ (Extended Data Fig. 10b,c, Supplementary Table 21 and Supplementary Note 5). Overall, these data from experimental and natural settings support the hypothesis that mature lymphomyeloid progeny downstream of HSC-iM inherit an inflammation-associated transcriptional program.

Finally, we leveraged the intermountain risk score (IRS)^58^, which predicts all-cause mortality, to evaluate potential health consequences of the transmission of the HSC-iM state to mature progeny. HSC-iM and HSC-I programs were scored in scRNA-seq of peripheral blood from a cohort of 428 participants in the Ontario Health Study who were stratified by a modified IRS and age (Fig. 5i). In young individuals, the HSC-iM score in B cells, naive T cells and dendritic cells significantly correlated with a high modified IRS, whereas the HSC-I score in natural killer cells, naive T cells, dendritic cells, and CD14 and CD16 monocytes correlated with a low modified IRS (Fig. 5j). By contrast, the effect sizes in aged individuals are small, suggesting that the relationship between previous inflammatory insults and mortality risk may be age dependent. Together, these data suggest that the transmission of the HSC-iM program to progeny may be associated with a higher mortality risk.

## Discussion

Here we have reported the discovery of a new HSC subset, termed HSC-iM, that retains transcriptional and epigenetic memory of previous inflammation. The experimentally identified HSC-iM molecular program is reflective of a lifetime of accumulated inflammatory insults in multiple human physiological and disease settings: severe COVID-19, an inherited blood disorder associated with inflammation, ageing and CH. This finding provides support for the concept of conserved inflammatory memory programs among mouse and human tissue stem cells^41,43,59^. The concordance between T cell memory and HSC-iM programs points to conserved responses among long-lived blood cells after recovery from repeated inflammatory stimuli. Moreover, lymphomyeloid progeny from HSC-iM inherit transcriptional features of HSC-iM including inflammatory signalling. Given that many mature blood cells have a short lifespan, our findings suggest that HSC-iM-derived progeny may continuously and actively influence and/or alter immune responses, with the potential to exacerbate systemic inflammation in a disease setting. Thus, HSC-iM constitutes an important cellular link between infection and inflammatory history, human ageing and clonal disorders of the haematopoietic system.

We propose that the HSC-iM subset emerges as an adaptation to inflammation, where the ‘cost’ of modifying HSC fitness has both beneficial and deleterious consequences^60,61^. Inflammatory insults drive some HSCs into dormancy, possibly as a protective adaptation to safeguard stem cell pool integrity over the lifespan of an individual^60^. In support of this concept, HSC-iM exhibited decreased haematopoietic output in both the xenograft and the CH bone marrow settings, suggesting that HSC-iM accumulation may contribute to age-related haematopoietic dysfunction at an organismal level. This could translate to potential long-acting negative effects on human health, as supported by the association of HSC-iM enrichment in immune progeny with risk scores for all-cause mortality^23,36,51^.

Finally, as HSC-iM enrichment can reflect the systemic level of inflammation in unhealthy individuals, some of the disease correlations attributed to CH status in current literature may potentially reflect survivorship bias of an earlier HSC-iM-driven inflammatory state^1,4^. First, HSC-iM was overrepresented among both mutant and WT cells from CH donors, suggesting that those with detectable CH clones may have had greater exposure to, or are more impacted by, inflammageing. Second, gene expression changes associated with CH mutations occur predominantly within HSC-iM and not HSC-I, and CH mutations appear to alleviate the dormancy/differentiation block inherent to HSC-iM — a conclusion supported by the strong correlation between HSC-iM enrichment and clone size. Third, the consequence of increased haematopoietic output from HSC-iM-derived CH-mutant clones could be greater numbers of intrinsically pro-inflammatory immune cells, which may feed forwards into the inflammatory environment associated with CH. Many open questions remain pertaining to the precise functions, origins and stability of HSC-iM, but these require new markers for prospective isolation^62^ and clonal tracking methods. In summary, a HSC subset that retains memory of previous inflammatory stress through heritable molecular alterations provides a new cellular framework for investigating heterogeneity of health outcomes arising from ageing and age-associated human diseases.

## Methods

### Ethics statement

Human CB samples were obtained with informed consent from Trillium Health, Credit Valley and William Osler Hospitals according to procedures approved by the University Health Network (UHN) Research Ethics Board (REB# 02-0763). The investigation of data from the Ontario Health Study was approved by the University of Toronto Research Ethics Board (protocol #00033112). Consent for use of data and blood samples was previously collected through the Ontario Health Study^63^ and CARTaGENE^64^ regional cohorts within the Canadian Partnership for Tomorrow’s Health^65^. All research using human material was performed in accordance with relevant guidelines and regulations.

### Human CB sample processing

Mononuclear cells from pools of male and female CB (approximately 1–8 donors) were obtained by centrifugation over a density barrier of Lymphoprep (Multicell). After red cell lysis with ammonium chloride (STEMCELL technologies), mononuclear cells were enriched for HSPCs by positive selection with a CD34 Microbead kit (Miltenyi) per the manufacturer’s instructions, or by lineage depletion as previously described^66^. Resulting HSPC-enriched CB cells were cryopreserved in 50% PBS, 40% fetal bovine serum (FBS) and 10% DMSO and stored at −80 °C short term or −150 °C long term.

### CB LT-HSC scRNA-seq

Human immunophenotypic LT-HSCs were purified from pooled CB samples based on a Lin^–^CD34^+^CD38^−^CD45RA^−^CD90^+^CD49f^+^ immunophenotype (see FACS section below). CB LT-HSCs were purified and submitted to the Hospital for Sick Children Genomics Core for scRNA-seq profiling on the BD Rhapsody platform.

### Mice

Animal experiments were done in accordance with institutional guidelines approved by the UHN Animal Care Committee. The following mouse strains (The Jackson Laboratory) were used in this study: NOD.Cg-*Prkdc*^*scid*^ *Il2rg*^*tm1Wjl*^*/SzJ* (NSG; strain 005557), NSG-Tg(CMV-IL3,CSF2,KITLG)1Eav/MloySzJ (NSG-SGM3; strain 013062) and NSG-*Kit*^*em1Mvw*^*/SzJ* (NSGW41). All in vivo experiments were done with 8–12-week-old female or male mice. NSG and NSG-SGM3 were conditioned with 225 cGy of gamma radiation; NSGW41 females were not irradiated. All mice were housed at the animal facility (ARC) at Princess Margaret Cancer Centre in a room designated only for immunocompromised mice with individually ventilated racks equipped with complete sterile microisolator caging (IVC), on corncob bedding and supplied with environmental enrichment in the form of a red house or tube and a cotton nestlet. Rooms were maintained at 21–22 °C and 30–40% humidity with a 12 h–12 h light–dark cycle with sunset and sunrise. Cages were changed at least once a week under a biological safety cabinet. Health status was monitored using a combination of environmental monitoring and evaluation of soiled bedding from sentinel mice.

### FACS and flow cytometry analyses

HSPC-enriched human CB cells were thawed via slow dropwise addition of X-VIVO 10 media (Lonza) with 50% FBS and 100 μg ml^–1^ DNaseI (Roche). Cells were centrifuged at 400*g* for 10 min, then resuspended in PBS + 5% FBS. The populations sorted for individual experiments are indicated; populations are designated based on the full stem and progenitor hierarchy as previously described^37,67^. Cells were resuspended at less than 10^7^ cells per millilitre and stained in one or two subsequent rounds for 15 min at room temperature each. See Supplementary Table 22 for antibodies. Cells were washed following staining and resuspended in PBS + 2% FBS and filtered through a 35-μm nylon mesh for sorting or analyses. To isolate xenografted human cells, bone marrow from three to five mice per experimental group was thawed, pooled, mouse depleted using a MACS-based kit (Miltenyi) and stained for cell sorting. Cells were isolated with BD FACSAria Fusion, BD FACSAria III or BD Symphony S6 cell sorters, or analysed on BD FACSCelesta or BD Symphony A1 coupled to a high-throughput system; instruments were controlled using BD FACSDiva software (v9). Flow cytometry data were evaluated using FlowJo (v10.8–10.10).

### CB xenotransplantation

Following cell sorting, 8,000–10,000 CD34^+^CD38^−^ cells were transplanted by intrafemoral injection into the right femur of appropriately conditioned age-matched and sex-matched mice in a volume of 30 μl PBS. The number of recipients was selected to minimize animal usage (*n* = 3–5 per group) while ensuring statistical comparisons can be made, and cohorts reproduced with independent CB pools to account for donor variability. Mice were randomized. To induce acute inflammatory stress, mice were injected intraperitoneally with 5 μg TNF or 40 μg LPS at the indicated time points. Control groups were injected with the vehicle control PBS. Mice were randomly assigned to experimental groups. Mice were euthanized at the indicated time points by cervical dislocation and the injected femur along with other hindlimb long bones (non-injected femur and both tibias) collected into Iscove’s modified Dulbecco’s medium + 5% FBS. Spleens were also collected in the same medium. Mice were numbered, and technical work was subsequently carried out without knowledge of experimental grouping. For some experiments, blood was collected from the saphenous vein of mice into K_2_EDTA tubes (BD) immediately before euthanasia. Injected and non-injected bones were flushed separately in Iscove’s modified Dulbecco’s medium + 5% FBS using a 23-G needle for some experiments. Alternatively, bones from most mice euthanized at a 20-week post-transplant time point were crushed in PBS + 5% FBS using a mortar and pestle and the resulting cell suspension passed through a 40–70-μm nylon mesh. Spleens were macerated using a syringe plunger, then passed through a 35-μm nylon mesh. Typically, 5–10% of cells were analysed for human chimerism and lineage markers using flow cytometry; antibodies are listed in Supplementary Table 22. Remaining cells were cryopreserved. Plasma was separated from blood and cryopreserved. For serial transplantation, xenografted cells were sorted for human CD45 (Supplementary Table 22) and transplanted into NSG-SGM3 mice at the indicated cell doses. A mouse was considered engrafted if human chimerism was more than 0.05% and LT-HSC frequency by limiting dilution assay was estimated using the ELDA software^68^ (http://bioinf.wehi.edu.au/software/elda/).

### T cell culture and CapTCRseq

Approximately 300,000 CD45^+^CD34^−^CD19^−^CD33^−^ were isolated by FACS as described above from cryopreserved xenograft bone marrow to enrich T cells and deplete B, myeloid and progenitor cells. Cells were seeded in X-VIVO 20 media supplemented with 5% human AB serum and 30 U ml^–1^ hIL-2 and stimulated with anti-CD3/CD28 Dynabeads (0.5 beads per cell)^69^. Live cells were counted by trypan blue exclusion on a haemocytometer and maintained in culture for 8 days before harvesting. Genomic DNA was extracted with a QIAamp DNA blood mini kit (Qiagen), quantified using a NanoDrop ONE instrument and submitted for CapTCRseq^70^ at the Princess Margaret Genomics Centre. Shannon’s diversity index was calculated for the reads obtained for the three TCR genes as previously described^71^.

### Cytokine array

Cryopreserved plasma was submitted for a multiplex human/mouse cytokine assay (HUMU41) to EVE technologies.

### scMultiome and scRNA-seq library preparation

CB was thawed and stained for FACS and sorted for CD19^−^CD34^+^CD38^−^ and CD19^−^CD34^+^CD38^+^ populations. The two populations were pooled back together at known ratios for further processing for scMultiome. Cryopreserved bone marrow from xenografted mice was processed for FACS and sorted for CD19^−^CD34^+^CD38^−^CD45RA^−^ HSPCs for scMultiome and CD19^−^CD34^−^CD33^+^ myeloid cells or CD19^−^CD34^+^CD38^+^ progenitor cells for scRNA-seq. scMultiome processing, including nuclei isolation and library construction, was performed by the Princess Margaret Genome Centre for downstream 10X Genomics scMultiome RNA + ATAC sequencing using their standard or low-input protocol, as appropriate. Specifically, for generation of the xenograft datasets described in Figs. 2a and 5d, we utilized three CB pools derived from a total of 62 individual donors, with approximately equal representation of male and female donors. The three biologically independent pools, each with three treatment conditions, namely, PBS, TNF or LPS, were sorted separately. Ultimately, cells profiled were derived from a total of 13 mice for the PBS and LPS groups, and 15 mice for the TNF group. The HSPCs from each treatment group were pooled after sorting for downstream processing. The CD34^+^CD38^+^ progenitors and CD33^+^ myeloid cells were cryopreserved as separate biological samples and subsequently thawed, and viable cells were pooled into PBS, TNF or LPS. The 10X Genomics 3′ scRNA-seq library preparation and sequencing were performed by the Princess Margaret Genome Centre using their standard protocol.

### CB LT-HSC scRNA-seq QC and preprocessing

Raw sequencing data were aligned using the BD SevenBridges pipeline, with default parameters. CB LT-HSCs were demultiplexed from other samples in the same run based on sample-specific barcodes. Cells passing the following criteria were used for downstream analysis: unique feature counts of more than 500, total number of molecules detected within a cell of more than 1,000, percent mitochondrial genes of less than 5%. Raw counts were normalized using scran (v1.20.1)^72^, followed by variable gene selection, scaling, principal component analysis (PCA) and neighbour graph construction with ten PCA components and *k*-nearest neighbours equal to ten. The neighbour graph was used to construct a force-directed graph for visualization using v1.8.2 Scanpy^73^.

### Consensus NMF

Consensus non-negative matrix factorization (cNMF; cnmf v1.3.4)^74^ was run with 100 iterations on the single cells from components 2 to 15 to derive transcriptional gene programs of variation in an unsupervised manner. Raw counts were fed into the –counts flag, and SCTransform (v2)^75^ corrected counts specifically from the data slot of the SCT assay were fed into the –tpm flag of the cNMF command line function with ten workers. After running cNMF across various components, the optimal number of components was selected by assessing the silhouette score (stability) and Frobenius reconstruction error (error), as implemented in cNMF. The transcriptomic signatures were characterized using an overrepresentation analysis with the function, ‘fora’ from the package fgsea (v1.18.0)^76^ on the top 100 genes from each signature with HSC gene sets and additional pathways from MSigDb.

### CB cNMF program

The gene expression programs of quiescence and inflammation were derived from the CB Multiome and CB LT-HSCs Rhapsody scRNA-seq datasets. These programs were characterized using overrepresentation analysis with hallmark and HSC gene sets (see ‘Data availability’ statement). To closely evaluate the relevance of the inflammation and quiescence programs identified by cNMF within the rhapsody LT-HSC and CB HSPC Multiome datasets, consensus meta-programs were defined by calculating the geometric mean of the feature weights between corresponding programs from each dataset. The genes were ranked by the combined feature weights and the top 200 were used for downstream analysis.

For further validation of meta-programs, additional CB scRNA-seq data from Lehnertz et al.^77^ and Zheng et al.^78^ were used. Specifically, scRNA-seq data from Zheng et al.^78^ were downloaded from GSE97104 and the published pre-processed dataset was used for downstream analysis. Moreover, raw count scRNA-seq data from Lehnertz et al.^77^ were downloaded from GSE153370. Doublets were filtered out using scDblFinder (v1.6.0) and further RNA filtering was performed. Cells passing the following criteria were used for downstream analysis: percentage of mitochondrial genes less than 7% and number of unique RNA transcripts detected of less than 5,000. Single-cell transcriptomes from both datasets were projected onto BoneMarrowMap^32^ with default parameters and HSCs were retained for downstream analysis. The enrichment for each inflammation and quiescence meta-program (based on the top 200 genes) were scored by AUCell.

### scMultiome (RNA and ATAC) preprocessing

Chromium single-cell Multiome ATAC + gene expression sequencing data (scMultiome) was pre-processed with CellRanger-ARC (v2.0.0) and aligned to GRCh38. The RNA feature count matrix for each sample was corrected for ambient RNA contamination using SoupX (v1.6.2)^79^ with default parameters. SoupX-corrected RNA counts were processed using Seurat (v4.3.0)^80^ and ATAC peak counts were processed using Signac (v1.6.0)^81^. RNA and ATAC quality control filtering utilized the following metrics: percentage of mitochondrial genes (pct_mito), nucleosome banding pattern (nucleosome_signal), ATAC transcriptional start site enrichment (TSS_enrichment), number of unique RNA transcripts detected (nFeature_SoupX) and number of fragments in peaks (nCount_ATAC). Thresholds for quality control filtering were adapted to each dataset based on the distribution of quality control metrics. For the CB xenograft scMultiome samples, filtering thresholds were set at: nFeature_SoupX > 1,000, pct_mito < 18%, nCount_ATAC > 1,000, nucleosome_signal < 2 and TSS_enrichment > 1. For the primary CB scMultiome samples, filtering thresholds were set at: nFeature_SoupX > 300, pct_mito < 8%, nCount_ATAC > 1,000, nucleosome_signal < 2 and TSS_enrichment > 1. For the ageing scMultiome samples, filtering thresholds for RNA-seq were set unique to each sample: BM_24M (nFeature_SoupX > 300 and pct_mito < 10%), BM_26F (nFeature_SoupX > 200 and pct_mito < 15%), BM_57M_58F (nFeature_SoupX > 100 and pct_mito < 18%), BM_70F (nFeature_SoupX > 200 and pct_mito < 12%) and BM_77F (nFeature_SoupX > 200 and pct_mito < 14%). For the ageing scMultiome samples, ATAC-seq filtering thresholds were uniform for each sample: nCount_ATAC > 1,000, nucleosome_signal < 2 and TSS_enrichment > 1.

Furthermore, pooled scMultiome samples with multiple donors (CB pools A and B, bone marrow middle-aged donors and CB xenograft) were separated using SoupOrCell^82^ through genotype-based clustering of sequencing reads from scRNA-seq profiles. The optimal number of genotypic clusters was selected using an elbow plot of the total log probability or manually selected when the number of donors was known.

### Donor sex classification

Pools of CB donors were engrafted into NSG mice from which CB xenograft samples were derived. Furthermore, some libraries of primary CB and bone marrow samples consisted of a pool of one male sample and one female sample. To account for variation from donor sex in CB xenograft samples and to demultiplex pools of primary CB and bone marrow samples, we developed an approach to cluster single cells by donor sex.

High-confidence sex classifications were first assigned by ATAC and RNA. For ATAC: among cells with an ATAC read depth of more than 6,000, those with one or more Y chromosome reads were classified as male, and those without were classified as female. For RNA: cells were scored for male-specific (on Y chromosome, excluding the pseudo-autosomal region) genes and female-specific (*XIST* and *TSIX*) genes using AddModuleScore from Seurat (v4.3.0)^80^. These scores were minimum–maximum normalized between 0 and 1, and adaptive thresholding was applied using a function adapted from the AUCell (v1.14.0)^83^ package. Cells surpassing this enrichment threshold for the male-specific gene set with zero female-specific gene expression were classified as male, and those surpassing this enrichment threshold for the female-specific gene set enrichment with zero male-specific gene expression were classified as female. Cells confidently classified as male or female by both RNA and ATAC were used, and differential expression was performed between these male and female cells to identify donor-specific genes within each dataset. Dimensionality reduction and unsupervised clustering were subsequently performed based on these donor-specific genes to cluster cells into male and female donors, and to identify clusters of doublets expressing both male and female genes. This approach was validated on artificially mixed snRNA-seq data from male and female HSPC from CB and bone marrow samples, and applied to multiplexed scMultiome samples from CB xenograft, primary CB and primary bone marrow.

### Doublet identification

Within multiplexed samples, doublet cells with mixed donor genotypes were identified by SoupOrCell^82^. Simultaneously, snRNA-seq-based doublet identification was performed using scDblFinder (v1.6.0)^84^ with default parameters. We confirmed that transcriptomes with co-expression of male-specific and female-specific genes were captured as doublets by SoupOrCell as well as scDblFinder. All identified doublets were removed from downstream analysis.

### CB xenograft processing and classification

After initial quality control, single-cell transcriptomes from the CB xenograft experiment were normalized by scran (v1.20.1), followed by variable feature selection, scaling and PCA reduction. Batch correction with Harmony (v0.1.1)^85^ was performed based on inferred donor sex. Initial dimensionality reduction and clustering revealed two outlier clusters each representing less than 1% of all cells, appearing to be contaminating T cells and pro-B cells. These clusters were excluded, and these data were re-processed, using the top 20 Harmony-corrected principal components, which were used to construct a neighbourhood graph using the top 30 nearest neighbours for each cell. UMAP reduction was performed with min.dist = 0.2 and spread = 1.

For chromatin analyses, peaks were called using MACS2 (v2.2.7.1)^86^ from fragment files corresponding to high-quality cells after quality control filtering. Peaks were called independently from HSPCs in each treatment condition (PBS + recovery, TNF + recovery and LPS + recovery), following the Signac pipeline. Peaks within non-standard chromosomes, genomic blacklisted regions, and those spanning less than 20 bp or more than 10,000 bp were removed from downstream analyses. From the resulting peak matrix, dimensionality reduction with latent semantic indexing (LSI) was performed following the Signac pipeline and LSI components were batch corrected by donor sex using Harmony. Harmony-corrected LSI components 2:30 were used to construct a neighbourhood graph using the top 30 neighbours for each cell. UMAP reduction was performed with min.dist = 0.2 and spread = 1.

For integrative analysis, WNN integration^49^ was performed using Harmony-corrected RNA PCA components 1:20 and Harmony-corrected ATAC LSI components 2:30, considering the top 30 neighbours for each cell. UMAP reduction was performed with a min.dist = 0.2 along the WNN neighbourhood graph.

### CB xenograft cell-state annotations

For cell-state annotations, transcriptomes were projected onto BoneMarrowMap^32^ (https://github.com/andygxzeng/BoneMarrowMap) and filtered at a mapping error threshold of 2 MADs (median absolute deviations) above the median. These cell-state assignments were used to guide clustering from the WNN graph: Leiden clustering was performed at varying resolutions from 0.1 up to 10. Specific Leiden clusters from resolutions of 1, 5 or 10 were selected based on high concordance with cell-state assignments from BoneMarrowMap, and a combination of these clusters was used for cell-state annotation. For a subset of cells (approximately 1%) that were not captured by these clusters, annotation was performed based on the most frequent cell-state annotation of their nearest neighbours.

### Integrated UMAP embeddings by treatment condition

For embeddings generated within each condition, WNN integration was run using the top 30 neighbours from Harmony-corrected RNA PCA components 1:20 and Harmony-corrected ATAC LSI components 2:20. UMAP reduction was performed from neighbourhood graphs for RNA only, ATAC only and integrated WNN with min.dist = 0.2 and spread = 1.2. This was repeated for each individual condition: PBS + recovery, TNF + recovery and LPS + recovery.

### OCAT embedding by CB treatment condition

The conditions PBS, TNF and LPS were each treated as a separate batch. The three batches of scRNA-seq data commonly share 36,601 genes, each with 10,097 cells, 8,069 cells and 8,100 cells, respectively. No genes or cells were filtered in this analysis. OCAT^87^ was used to integrate these three batches of scRNA-seq datasets. OCAT pre-processed the raw gene expression matrices through log transformation and log_2_ normalization and performed dimension reduction on the original data to a *d* = 100 subspace. OCAT then selected 100 ‘ghost’ cells, centres of small cell neighbourhood in the reduced subspace, and connected each individual cells to the global ghost cell set (*m* = 100 × 3) through a bipartite graph. OCAT further made these edge weights sparse by allowing at most 30% of them to be non-zero. These sparse edge weights are treated as the OCAT representation of each cell and were used for downstream visualization with UMAP.

### TooManyCells embedding by CB treatment condition

For analysis with TooManyCells (v2.2.0)^88^, single-cell transcriptomes were filtered to remove cells with less than 250 counts and only include genes present in at least 1 cell to discard low-quality reads. The filtered samples were normalized with term frequency-inverse document frequency and clustered with the TooManyCells divisive hierarchical clustering using matrix-free spectral clustering. The resulting cluster trees were pruned to a minimum of 30 cells for each leaf node to reduce the overall size of the tree and focus visualization on larger sub-populations of cells. Once pruned, the trees were labelled with cell-type annotations, highlighting the clustered differences between HSC and HSC-II populations, including additional annotation for MPP–MyLy (associated with the HSC population) and MPP-II (associated with the HSC-II population).

### TooManyPeaks embedding by CB treatment condition

For analysis with TooManyPeaks^89^, chromatin peak matrices derived from scATAC-seq were filtered to remove cells with less than 1,000 peaks. The filtered samples were processed with latent semantic analysis for dimensionality reduction with 50 components. The peaks for scATAC-seq were not normalized. The filtered samples were clustered with TooManyPeaks, the sister method to TooManyCells. The resulting clustered trees were pruned to a minimum of 200 cells, as scATAC-seq data usually produce an exceptionally large number of clusters (leaf nodes). Once pruned, the trees were labelled in the same manner as the scRNA-seq data analysed with TooManyCells.

### Transcriptional programs from xenograft scMultiome

To find reliable transcriptional markers for each cell state, a composite score was derived from four distinct differential expression statistics. At the single-cell level, the ‘wilcoxauc’ function from presto (v1.0.0)^90^ was applied to obtain the following statistics: (1) log_2_-transformed fold change (log_2_FC) from single cells in a group versus single cells from all other groups; and (2) AUROC metric for distinguishing between cells in a group versus cells from all other groups. Next, pseudobulk profiles were created by combining cells based on cell type, donor sex and treatment condition (excluded pseudobulks with less than five cells), and DESeq2 (ref. ^91^) was used to compare pseudobulks from each cell state against those from all other cell states through a likelihood ratio test. On the basis of this pseudobulk analysis, the following statistics were also incorporated: (3) test statistic from DESeq2; and (4) log_2_FC from DESeq2. The geometric mean from all four statistics was used to prioritize marker genes for each cell state and subsequently called ‘MarkerScore’.

This metric was also used to identify top markers between HSC-I and HSC-II, although the pseudobulk DESeq2 analysis was performed directly contrasting HSC-I and HSC-II, and accounting for treatment (PBS, TNF and LPS) as a covariate. The top 200 genes for each population were used as a program for scoring in external datasets.

### Chromatin accessibility in xenograft scMultiome

For differential chromatin accessibility, pseudobulk profiles were created by combining scATAC-seq cells based on cell type, donor sex and treatment condition, and DESeq2 (ref. ^91^) was used to compare pseudobulks from each cell state against those from all other cell states through a likelihood ratio test, while adjusting for treatment (PBS, TNF and LPS) as a covariate. Significantly differentially enriched peaks were used as signatures for scoring enrichment in external datasets. Among differentially enriched peak sets, transcription factor motif enrichment was determined through the ‘FindMotifs’ function in Signac (v1.6.0)

### GSEA

Gene set scoring was done with AUCell (RNA) and chromVAR (ATAC), as described below. GSEA was performed using the function fgseaMultilevel from fgsea (v1.18.0), with the following parameters: nPermSimple = 1,000,000, eps = 0, minSize = 15 and maxSize = 500. The test statistic from DESeq2 (ref. ^91^) was used as the rank statistic for each gene. For complete biological pathway analysis, GSEA was performed using the April 2023 version of ‘Human_GOBP_AllPathways_no_GO_iea’ from Gary Bader’s laboratory in Toronto (https://download.baderlab.org/EM_Genesets/current_release/Human/symbol/). EnrichmentMap^92^ was used for visualization of biological pathway GSEA results, only retaining signatures and pathways enriched in a given cell type at FDR < 0.01.

For benchmarking of GSEA enrichment results, external gene sets were obtained from the GOBP collection described above as well as the chemical and genetic perturbations (CGP) collection from MSigDB (https://www.gsea-msigdb.org/gsea/msigdb/collections.jsp). After filtering for gene sets with a minimum size of 15 genes and a maximum size of 500 genes, 6,653 gene sets were retained from the GOBP and 2,615 gene sets were retained from the CGP, leading to a total of 9,270 external gene sets. Given that the differential expression rank lists differ in size by comparison, we further filtered for gene sets that had at least three genes in all of the following rank lists: older-aged or middle-aged HSC versus younger-aged HSC, ICU-COVID HSC versus control HSC, ICU-COVID monocyte versus control monocyte, *DNMT3A-*mutant CH HSC versus control HSC, *DNMT3A* WT CH HSC versus control HSC, *TET2*-mutant CH HSC versus control HSC, and *TET2*^*WT*^ CH HSC versus control HSC. This resulted in 8,312 external gene sets, which constituted a universal benchmark for GSEA results from each of these differential expression comparisons. Next, GSEA was rerun on HSC-iM and HSC-I programs together with all 8,312 external gene sets, and multiple testing correction was applied together on the full set of 8,314 gene sets (that is, including the HSC-iM and HSC-I programs). Gene sets were subsequently ranked by significance statistics.

### Augur

Augur (v1.0.3)^93^ was used to determine separability of single cells between HSC-I and HSC-II pertaining to gene expression and chromatin accessibility profiles, applied independently to each treatment condition (PBS, TNF and LPS). For gene expression, Augur was applied on the normalized expression of 2,000 highly variable genes, with default parameters, whereas, for chromatin accessibility, Augur was applied to 50 LSI components, with default parameters. For ATAC, these reduced dimension components were used rather than raw chromatin peak counts due to high sparsity and number of features for the latter. The distribution of Augur classifier performance for each cross-validation split was used to represent the separability between HSC-I and HSC-II within each treatment condition.

### RNA and ATAC signature scoring

Gene set scoring of previously published gene expression signatures was performed using AUCell (v1.14.0)^83^. Enrichment of chromatin regions in scATAC-seq data was evaluated through chromVAR (v1.14.0)^94^. When necessary, hg19 coordinates for chromatin signatures were converted to hg38 using package rtracklayer (v1.52.1)^95^ with the UCSC hg19tohg38 chain file. For both AUCell-based (RNA) and chromVAR-based (ATAC) enrichment scores, standardization was performed across cells before plotting for ease of visualization.

### SCENIC+ eRegulon inference

To infer transcription factor activity from RNA and ATAC profiles by SCENIC+ (v0.1)^39^ within the CB xenograft scMultiome data, the 27,492 single cells from the xenograft scMultiome were first aggregated to the metacell level using the divide and conquer algorithm from the metacell2 (ref. ^96^) package. The highly variable genes used to construct the UMAP were used as input into the algorithm guiding feature gene selection, thus optimizing clustering for metacell partitioning. The approach generated 1,476 metacells with a target metacell size of 75,000 unique molecular identifier. Next, pycisTopic (v1.0.2) was used to identify cell states and *cis*-regulatory topics from the ATAC single cells in an unsupervised manner. The ideal number of topics were evaluated as per the previously reported guidelines^97^. Consequently, pycisTopic optimized at 18 topics. Motif enrichment analysis was subsequently carried out by leveraging pycisTarget (v1.0.2), which utilizes precomputed databases comprising motif scores and rankings for genomic regions, and a motif-to-transcription factor annotation database from the Aerts laboratory resources (https://resources.aertslab.org/cistarget/databases/homo_sapiens/hg38/screen/mc_v10_clust/region_based/). This analysis was conducted on the topics from pycisTopic and differentially accessible regions between each defined cell type.

Following pycisTopic and pycisTarget, a SCENIC+ object was created for downstream steps to create enhancer-driven GRNs using the ATAC and RNA. First, regions and genes absent in more than 0.5% of cells were filtered out. Next, cistromes were generated using the function merge_cistromes, which overlaps targets assigned to a transcription factor from the motif enrichment dictionaries with the regions in the object. Then, enhancer-to-gene relationships were inferred by first defining the search space around each gene of 150 kb upstream or downstream, specifically on the metacells, to limit RAM usage while querying ENSEMBL BioMart (host 98). After, enhancer-to-gene models were generated using gradient-boosting machines with the function calculate_regions_to_genes_relationships. Next, transcription factor-to-gene relationships were inferred using pySCENIC on the RNA metacells as per previously published guidelines^83^ with a candidate list of transcription factors^98^ and loaded into the SCENIC+ object from a saved adjacencies matrix using the function load_TF2G_adj_from_file. Finally, enhancer-driven GRNs were generated using a GSEA recovery approach from SCENIC+ using the function build_grn. The recovered eRegulons were filtered using the function apply_std_filtering_to_eRegulons, restricting analysis to high-confidence transcription factors.

Given that a gene expression-based eRegulon and a chromatin accessibility-based eRegulon were reported for each transcription factor, we performed quality control by Pearson correlation and excluded transcription factors in which correlation between gene expression-based eRegulon activity and chromatin accessibility-based eRegulon activity was below *R* = 0.25. This led to 133 transcription factors in which eRegulon activity passed the correlation threshold and exhibited concordance between RNA and ATAC.

### Differential signature enrichment

Differential enrichment of gene or chromatin signature scores was performed by constructing linear mixed models for each signature using the cell class as an independent variable and accounting for donor sex as a random effect within the data using the function ‘dream’ from package variancePartition (v1.22.0)^99^. The raw and FDR-corrected *P* values from this analysis were used to represent significance. Simultaneously, the AUROC metric from the wilcoxauc function of the presto package (v1.0.0) was used to represent the ability of a signature enrichment score to accurately discern between contrasting cell classes.

Applied to SCENIC eRegulons in which two enrichment scores are used for each transcription factor, representing activity by gene expression as well as chromatin accessibility, these two results were integrated to identify top marker transcription factors for a given cell type or condition. Here the mean AUROC metric and the mean −log_10_(FDR) value were used together to identify top marker transcription factors across RNA and ATAC modalities.

### SCENIC+ transcription factor network construction

Among transcription factors with significant eRegulon activity enrichment in a specific cell state or condition at FDR < 0.05 by both RNA and ATAC modalities, GRNs were constructed. In brief, the strength of transcription factor regulation for a target gene was represented through two metrics: (1) importance of the RNA-based transcription factor-to-gene association, and (2) the importance of the ATAC-based chromatin region-to-gene association among nearby chromatin regions containing the transcription factor motif. The geometric mean of these two metrics was taken to represent the overall strength of regulation of target gene expression by a transcription factor. Finally, this score was subject to adaptive thresholding in line from the AUCell package as described above, and transcription factor-to-transcription factor regulation surpassing this adaptive threshold was retained to construct a transcription factor regulatory network from each condition.

### Analyses of external bulk RNA-seq datasets

For differential expression analysis in bulk RNA-seq datasets, DESeq2 (v1.32.0)^91^ was applied to raw count data. For sample-level scoring of gene set enrichment, GSVA (v1.40.1)^100^ scoring was performed on normalized gene expression data with the ‘kcdf’ parameter set to ‘Gaussian’. Normalized data from the original studies were used when available, otherwise raw count data were subject to vst normalization through DESeq2. For mouse bulk RNA-seq data, mouse genes were converted to human orthologues using babelgene (v22.3) and only conversions supported by a minimum of five databases were retained. These converted gene names were used for downstream processing as described.

For differential accessibility in bulk ATAC-seq datasets, peak count matrices were obtained from the original studies, and differentially accessible peaks were identified through DESeq2. Scoring of chromatin signatures was performed through chromVAR. When comparing gene signature and chromatin signature enrichment between groups of samples, Wilcoxon rank-sum tests were performed.

### Transcriptional signature of BCG vaccination

To derive a signature of mouse LSK^+^CD150^+^ HSCs exposed to the BCG vaccine (BCG-iv)^42^, differential expression analysis was performed through DESeq2 (ref. ^91^) comparing BCG-iv versus control conditions. This resulted in 490 genes upregulated in BCG-exposed mouse HSCs at FDR < 0.05 and log_2_FC > 1, constituting a mouse HSC BCG-iv signature.

To derive a signature of human CD34^+^CD38^−^CD45RA^−^ HSC–MPPs exposed to the BCG vaccine^44^, differential expression analysis was performed through DESeq2 comparing day 90 post-BCG vaccination versus day 0 pre-vaccination conditions. This resulted in 206 genes upregulated in HSC–MPPs at 90 days following BCG vaccination at FDR < 0.05 and log_2_FC > 1, constituting a human HSC–MPP BCG vaccination signature.

### ‘Akondy’ CD8 memory T cell signature

To derive a signature of functionally defined CD8 memory T cells^47^, differential expression was performed between T_M_ cells and naive T cells, and between T_M_ and T_eff_ cells. From 937 genes enriched in T_M_ versus naive T cells at log_2_FC > 1 and FDR < 0.01 and 1,543 genes enriched in T_M_ versus T_eff_ cells at log_2_FC > 1 and FDR < 0.01, we identified 257 overlapping genes significantly enriched in T_M_ cells compared with both naive T and T_eff_ cell subsets. These 257 genes represented the CD8 T cell memory signature. To derive a naive T signature, we utilized the same approach to identify 370 overlapping genes significantly upregulated in both naive T versus T_eff_ cell and naive T versus T_M_ cell comparisons. Owing to large gene set size, the top 200 genes were used for downstream signature scoring in xenograft HSCs.

### Memory T cell signature from CITE-seq data

CITE-seq data of human T cell subsets were obtained^49^. Cell-type annotations and UMAP coordinates were also extracted from the original publication. Differential expression was performed on pseudobulk profiles with DESeq2 (v1.32.0), adjusting for donor as a covariate. To derive CD8 and CD4 T cell memory signatures, DEGs unique to both central memory T and effector memory T cell subsets compared with naive T at log_2_FC > 1 and FDR < 0.05 were retained, and the top 200 enriched genes were used as the signature.

### ICU-COVID analyses and signature derivation

scMultiome of HSPCs in healthy control, ICU-control, and ICU-COVID donors were previously profiled^50^. Cells annotated as HSC–MPPs from this study were projected onto BoneMarrowMap, and transcriptional HSCs were purified in silico by reference map projection^32^. Differential expression analysis with DESeq2 (v1.32.0) was applied to pseudobulk profiles to compare HSCs from ICU-COVID donors against HSCs from ICU-control and healthy control donors, and 20 genes enriched in ICU-COVID at FDR < 0.05 were retained as an ICU-COVID recovery molecular signature. For analysis of monocytes in patients with ICU-COVID compared with healthy controls and ICU-controls, cells classified as CD14 monocytes and CD16 monocytes by reference map projection were retained, and pseudobulk-based differential expression was performed as outlined for the HSCs from these same donors.

### Differential expression between HSC across age

Profiling of primary CD34^+^ bone marrow cells by scMultiome as described above yielded 43,762 cells with high-quality RNA and ATAC profiles spanning 6 donors: 2 young adult donors (20–30 years of age), 2 middle-aged donors (50–60 years of age) and 2 older-aged donors (70–80 years of age). In addition to this data, scRNA-seq data were compiled from 3 additional cohorts comprising bone marrow samples of varying ages: (1) 10X v2 and v3 scRNA-seq from CD34^+^ bone marrow profiled in Ainciburu et al.^53^, spanning 5 young adult donors (20–30 years of age) and 3 older-adult donors (60–80 years of age); quality control filtering as per Jakobsen et al.^52^ yielded a total of 71,805 cells. (2) STRT-seq from CD34^+^ fractions bone marrow profiled in Zhang et al.^54^ spanning 3 young adult donors (20–30 years of age) and 2 older-adult donors (60–90 years of age); quality control from the original study yielded 3,023 cells. (3) 10X v2 scRNA-seq from mixed CD34^+^ and bulk bone marrow profiled in 4 studies within BoneMarrowMap^32^ spanning 13 young adult donors (18–40 years of age) and 9 MA donors (40–60 years of age); quality control filtering as per the BoneMarrowMap publication yielded 194,905 cells.

Single-cell transcriptomes from each cohort were classified by BoneMarrowMap projection, and transcriptional HSCs were purified in silico for downstream analysis. Collectively, this constituted 23,048 transcriptional HSC spanning 23 young adult donors, 11 middle-aged donors and 7 older-aged donors. Pseudobulk profiles were constructed from HSCs from each donor sample, and differential expression was performed between HSCs from middle-aged and older-aged donors and HSCs from young adult donors using DESeq2.

### Aged HSC meta-signature derivation

To derive a meta-signature of aged HSCs spanning all datasets, donor-specific HSC pseudobulk profiles from each dataset were pooled together. Differential expression was performed between HSCs from 18 middle-aged and/or older-aged donors and HSCs from 23 young adult donors, adjusting for the originating dataset as a covariate. Before interpretation of differential expression results, 370 genes that were upregulated in aged HSCs at log_2_FC > 0 within each of the 4 ageing datasets were retained. Among these 370 universally upregulated genes, 37 were significantly upregulated in aged HSCs by pooled differential expression at log_2_FC > 1 and FDR < 0.05. These 37 genes represent a meta-signature that is consistently upregulated across human HSC ageing.

### TARGET-seq+ clustering and cell-type mapping

TARGET-seq+ profiling, scRNA-seq preprocessing and cell-type annotation of human bone marrow HSPCs from CH and control donors are outlined in the original study^52^. Gene identifiers were converted to Ensembl v93 and HSCs were annotated by projecting the dataset onto the bone marrow reference map as described above. The HSC–MPP, LMPP, LMPP cycling and erythroid/megakaryocytic-primed MPPs (EMPP) clusters from our original study^52^ were further subclustered using the self-assembling manifolds (SAMs) algorithm (v1.0.1), using default settings with Harmony-adjusted principal components as input and using the sample identifier as the batch^101^. The resulting SAM-weighted PCA was then used as input to generate a UMAP and for Louvain clustering, which identified seven clusters.

For calculating the HSC2:HSC1 cell ratio, the number of cells in each cluster was calculated on a per-sample basis. Only cells sorted as part of the total Lin^–^CD34^+^ gate were included, to avoid bias introduced by FACS depletion of CD38^–^ cells.

### Differential expression in CH TARGET-seq+ data

Differential expression testing was performed with a linear mixed model to account for sample covariance using the dream pipeline from the variancePartition package^99^ (v1.22.0), which is based on limma-voom^102^. Testing was performed on log-normalized counts, using the scran normalization size factors. Genes were filtered to include only those expressed in at least 10% of cells in either group. A linear mixed model was fitted to each gene using ‘dream’, and differential expression testing was performed using ‘variancePartition::eBayes’ (variancePartition v1.33.0). For comparisons between CH and non-CH controls, the sample type was used as the test variable, and the sample identifier, age, sex and FACS-sorting plate batch effects included as covariates. For comparisons between HSC1 and HSC2, the cell type was used as the test variable, and sex was included as a covariate. For comparisons between genotypes within CH samples, the clone was used as the test variable, and the sample identifier and FACS-sorting plate batch effect included as mixed-effect covariates. For differential expression analysis between HSC1-dominant and HSC2-dominant hierarchies, the dominant HSC group was used as the test variable, and the sample identifier, sex and FACS-sorting plate batch effects included as random effect covariates.

### SCENIC analysis of TARGET-seq+ data

To infer transcription factor regulon activity, regulon analysis was performed using pySCENIC (v0.12.0)^83^, which was run as per previously published workflow guidelines. To identify candidate transcription factor regulons, filtered and pre-processed raw counts were used as the input alongside a list of human transcription factors^98^. Candidate regulons were pruned using the annotations of transcription factor motifs ‘motifs-v10nr_clust-nr.hgnc-m0.001-o0.0.tbl’, and CisTarget was applied using the ‘mc_v10_clust’ databases of known human transcription factor motifs annotated at: (1) 500 bp upstream and 100 bp downstream of the transcription start site (TSS); and (2) 10 kb centred around the TSS. No drop-out masking was applied. Enrichment of refined transcription factor regulons was quantified using AUCell, with default parameters. Tests for differential regulon activity were performed using a linear mixed model, as described above.

### Signature enrichment in TARGET-seq+ data

Differences in gene expression signature or transcription factor regulon scores between conditions were tested by a linear mixed model. For comparisons between CH and non-CH controls, the sample type was used as the fixed effect, and the sample identifier, age, sex and FACS-sorting batch as mixed-effect covariates. For comparisons between genotypes within CH samples, the clone was used as the fixed effect, and the sample identifier as a mixed-effect covariate. *P* values were obtained by a likelihood ratio test of the full model with the fixed effect against the model without the fixed effect.

### Bone marrow xenografts and TARGET-seq+

Bone marrow samples were collected from individuals undergoing elective total hip replacement surgery at the Nuffield Orthopaedic Centre under the Mechanisms of Age-Related Clonal Haematopoiesis (MARCH) Study, as described^52^. Written informed consent was obtained from all participants in accordance with the Declaration of Helsinki with approval by the Yorkshire & The Humber–Bradford Leeds Research Ethics Committee (NHS REC ref: 17/YH/0382). Bone marrow CD34^+^ cells purified using a CD34 MicroBead kit (Miltenyi Biotec) according to the manufacturer’s instructions were transplanted as for CB samples into irradiated NSG-SGM3 animals at 55,000–90,000 CD34-enriched cells per animal by intrafemoral injection. Mice were treated with PBS or 5 μg TNF at 2 weeks and 10 weeks post-transplantation. Human engraftment was assessed at 12 weeks post-transplantation as described above for CB and cells cryopreserved for downstream analysis. After mouse depletion (Miltenyi), samples were stained for FACS (see Supplementary Table 22) to pre-sort for hCD45^+^hCD34^+^hCD19^−^hCD33^−^ HSPCs and hCD45^+^CD33^+^CD34^−^CD19^−^ myeloid cells on a BD Fusion instrument. The NOC153 bone marrow sample was included as a technical control for each sort of a xenografted sample. Pre-sorted subpopulations underwent single-cell index sorting on a Sony MA900 into 384-well plates containing 3 µl lysis buffer for downstream library preparation and computational analysis as previously described with the comparable samples from the original TARGET-seq+ dataset^52^.

### GSEA analysis in human acute myeloid leukaemia

Comparison of BoneMarrowMap-defined HSC–MPP from acute myeloid leukaemia samples versus healthy controls. scRNA-seq data generated from six studies with (1) clinical information on *DNMT3A* and *TET2* status, and (2) internal healthy control samples, were included^32,103,104^. This constituted 91 acute myeloid leukaemia samples and 16 healthy control samples. From these 91 samples, 15 were clinically annotated as DNMT3A-MUT, 18 were clinically annotated as TET2-MUT, 35 were clinically annotated as WT for both DNMT3A and TET2 (double negative), and the remaining were not annotated for DNMT3A or TET2 mutation status. Pseudobulk profiles were obtained at the level of the HSC–MPP populations from each patient sample, and differential expression was performed with DESeq2 between acute myeloid leukaemia HSC–MPP and healthy HSC–MPP while controlling for study as a covariate.

### Myeloid and progenitor scRNA-seq QC and preprocessing

Raw sequencing data were pre-processed with CellRanger-ARC (v2.0.0) and aligned to GRCh38. The RNA feature count matrix for each sample was corrected for ambient RNA contamination using SoupX (v1.6.2)^79^ with default parameters. SoupX-corrected RNA counts were processed using Seurat (v4.3.0) and the following quality control-filtering thresholds were applied to each sample: percentage of mitochondrial genes (pct_mito) < 10% and number of unique RNA transcripts detected (nFeature_SoupX) > 300. After initial quality control, single-cell transcriptomes were normalized by scran (v1.20.1), followed by variable feature selection, scaling and PCA reduction. Batch correction with Harmony (v0.1.1)^85^ was performed with sex as a batch variable. A UMAP reduction was performed using 20 Harmony-corrected principal components, and a neighbourhood graph was constructed using the top 30 neighbours for each cell. Louvain clustering was performed at a resolution of 1.4, and clusters were condensed by concordance for predicted cell-type annotations upon mapping to the BoneMarrowMap^32^. Neutrophil progenitors were validated by evaluating expression for MPO and ELANE.

### Inferring genetic clonal groups using SoupOrCell

To exploit the extensive genetic heterogeneity within the 20-week xenograft data, we utilized SoupOrCell (v2.5), a tool that performs unsupervised clustering of genetic polymorphisms from the raw sequencing data within a single-cell experiment to capture this genetic heterogeneity as barcodes for lineage tracing. To combine the raw sequencing data from the CD34^+^CD38^−^CD45RA^−^ HSPCs with newly generated scRNA-seq data from CD34^+^CD38^−^ progenitors and CD33^+^ mature myeloid cells isolated from these same xenografts, we merged the gene expression bam files from CellRanger-ARC (v2.0.0) using pysam (v0.15.1). Merged bam files were sorted and indexed using samtools (v1.17) with default parameters. SoupOrCell was iteratively run across a parameter search from *k* 1–30, and the optimal number of genetic clonal groups was evaluated using an elbow plot of the total log likelihood and concordance with predicted sex assignments.

Clonal groups were assigned as HSC-I dominant, HSC-iM dominant and nonspecific based on cell composition within the HSC pool. Specifically, clonal groups with more than 95% HSC-iM or HSC-I within the HSC pool were assigned as HSC-iM dominant and HSC-I dominant, respectively. Remaining clonal groups (HSC-iM proportions ranging from 12% to 60% in the HSC pool) were assigned as nonspecific. For differential expression analysis between HSC-I-dominant and HSC-iM-dominant clonal groups, pseudobulk profiles were constructed from each cell type within each clonal group within each treatment condition, and DESeq2 (v1.32.0) was applied to pseudobulk profiles comparing HSC-iM-specific clones against HSC-I-specific clones for each cell type. GSEA was performed using the fgsea (v1.18.0) package. Before differential expression, pseudobulk profiles consisting of less than five cells were filtered out.

### Donor-level associations between HSCs and monocytes

To indirectly infer associations between transcriptional features of monocytes against HSC-iM program enrichment from upstream HSCs, scRNA-seq data from three scRNA-seq datasets of bulk mononuclear cells within BoneMarrowMap^32^ were used^105–107^. First, HSCs and CD14 monocytes from each donor were collapsed into pseudobulk profiles, and only healthy donors with 5 or more HSCs as well as 5 or more monocytes were included, leading to 32 donors used for this analysis. For each donor, the mean enrichment score for the HSC-iM program (top 200 genes scored by AUCell) within the HSC pool was obtained. Next, using the pseudobulk transcriptome for the monocytes from each donor, DESeq2 was applied to regress the monocyte transcriptomes against a continuous variable representing enrichment of the HSC-iM program within the HSC pool. Test statistics and *P* values were obtained for each gene within the monocyte transcriptome, and these statistics were used to construct a rank list for downstream GSEAs. Positive-test statistics translate to higher expression of a given gene in the monocytes of donors with greater HSC-iM enrichment within their upstream HSCs.

### Clone-level associations between HSCs and monocytes

To more directly infer associations between transcriptional features of monocytes against HSC-iM program enrichment from upstream HSCs, we utilized a native lineage-tracing dataset integrating mitochondrial DNA variant-based lineage tracing with concomitant multi-omic profiling of HSCs^8^. Pre-processed single-cell transcriptomes from their dataset were projected onto BoneMarrowMap^32^ to identify HSCs and CD14 monocytes. Next, among mitochondrial DNA-defined clones assigned from their study, we identified clones with at least three HSCs and at least three CD14 monocytes. This resulted in retention of 213 clones spanning 13 donors, which was used for association analysis. For each clone, the mean enrichment score for the HSC-iM program (top 200 genes scored by AUCell) within the HSC pool was obtained. Next, using the pseudobulk transcriptomes for the monocytes from each clone, DESeq2 was applied to regress the monocyte transcriptomes against a continuous variable representing enrichment of the HSC-iM program within the HSC pool. Test statistics and *P* values were obtained for each gene within the monocyte transcriptome, and these statistics were used to construct a rank list for downstream GSEAs. Positive-test statistics transplate to higher expression of a given gene in the monocytes of clones with greater HSC-iM enrichment within their upstream HSCs.

### BM xenograft TARGET-seq+ data processing

Raw sequencing data were preprocessed with the TARGET-seq+ pipeline (https://github.com/asgerjakobsen/TARGET-seq-plus) as previously described^52,108^. In brief, transcriptome data were preprocessed with a custom pipeline (https://github.com/asgerjakobsen/TARGET-seq-plus-RNA) to trim and demultiplex reads into single-cell barcodes using cutadapt (v3.4) and then mapped them to the hg38 reference genome and ERCC92 transcripts with STAR (v2.7.10a) using the GENCODE v38 reference gene annotation. Genotyping amplicon data were demultiplexed and processed to generate tables of allelic counts, which were used to call cell genotypes as previously described^52^ using single-cell data from the NOC153 primary bone marrow sample as WT controls. Finally, FACS indexing, genotyping and transcriptome data were integrated for downstream analysis in Seurat (v5.3.0).

The following quality control filters were applied to the starting dataset of 10,640 cells: minimum of 5,000 RNA reads per cell, minimum of 1,000 genes per cell, maximum percentage of mitochondrial reads = 15% and maximum percentage of ERCC reads = 65%, leaving 8,998 cells remaining. The filtered data were integrated with the 13,247 primary bone marrow cells from the original TARGET-seq+ dataset^52^. Genes expressed in fewer than 10 cells were removed and reads were normalized by scran (v1.36.0) using the pool normalization method with prior clustering, followed by variable feature selection, scaling and PCA reduction. Batch correction with Harmony (v1.2.3) was performed with sample donor and experimental batch as batch variables. A UMAP reduction was performed using 30 Harmony-corrected principal components. Louvain clustering was performed at a resolution of 1. Clusters were annotated and condensed by concordance with predicted cell-type annotations upon mapping to the BoneMarrowMap along with the cell-type annotations from the original primary bone marrow dataset and immunophenotypic profiles.

### Inference of HSC origin in TARGET-seq+ data

To identify HSC-I-derived and HSC-iM-derived cells, we first defined a set of genes that distinguish the differentiation trajectories of HSC-I-dominant and HSC-iM-dominant primary bone marrow samples in the original TARGET-seq+ dataset. Within each cell type, single-cell transcriptomes from each donor were collapsed into pseudobulk profiles, and EdgeR was used to identify genes differentially expressed between HSC-I-dominant (*n* = 6) and HSC-iM-dominant (*n* = 4) samples. We selected genes that were differentially expressed at FDR < 0.10 within 5 or more cell types, generating a list of 702 genes. We then performed dimensionality reduction and clustering of HSPCs and monocytic cell types in the integrated TARGET-seq+ dataset using the SAM algorithm and the 702 HSC-I and HSC-iM trajectory genes. Louvain clustering was performed on the SAM embeddings at a resolution of 1.5, generating 15 clusters. These clusters were then annotated as being of HSC-I or HSC-iM origin based on the composition of primary bone marrow cells from HSC-I-dominant and HSC-iM-dominant samples and expression of the HSC-I and HSC-iM transcriptional programs.

For analysis of monocytic output in bone marrow xenografts, the number of early HSPC cells (HSC–MPP, LMPP, cycling progenitor and early GMP) and monocytic cells (late GMP, promonocytes and monocytes) were calculated separately for each sorted population (CD34^+^ and CD34^–^CD33^+^), and the CD34^–^CD33^+^ counts then multiplied by the ratio of CD34^–^CD33^+^:CD34^+^ cells in FACS-sorting gates to account for the size of each population in the xenograft sample. The frequency of monocytic cells was then divided by the frequency of early HSPCs to obtain a relative abundance.

### scRNA-seq in the OHS to relate HSC-iM to IRS

#### Study population

CanPath is a population cohort of over 330,000 participants across 7 provinces in Canada, and has collected information on health, lifestyle, disease and environmental factors along with biological samples. Requests for access to CanPath data should be made through the data access portal (https://portal.canpath.ca/).

#### Sample selection

A modified version of the IRS (mIRS) was calculated for all participants using the sex-specific 5-year mortality risk coefficients for six values on a complete blood count: haematocrit, white blood cell count, platelet count, mean corpuscular volume, mean corpuscular haemoglobin concentration and red blood cell distribution width. The age component was excluded from the calculation to enable direct comparisons of blood cell profiles among participants of all ages, and the metabolic parameters were also excluded. The complete blood count-based mIRS has been shown to be associated with all-cause mortality in a cohort of approximately 30,000 participants^109^, as well as a randomized trial population of approximately 17,000 (ref. ^110^). Participants with any of the following self-reported diseases were excluded: high blood pressure, myocardial infarction, stroke, asthma, emphysema, chronic bronchitis, chronic obstructive pulmonary disorder, depression, diabetes, liver cirrhosis, chronic hepatitis, Crohn’s disease, ulcerative colitis, irritable bowel syndrome, eczema, systemic lupus erythematosus, psoriasis, multiple sclerosis, osteoporosis, arthritis and cancer. Samples were further filtered to retain only those with European ancestry, and those that were 45 years of age or younger, or 65 years of age or older, classified as young and aged, respectively. For extreme sampling, both the young and the aged groups were ranked according to their age and mIRS, and the top and bottom approximately 100 samples from each age group were retained for molecular phenotyping.

#### scRNA-seq library preparation, sequencing and data preprocessing

Tissue thawing medium (TTM) was prepared by mixing 10% FBS, 25 mM HEPES, 55 μM 2-mercaptoethanol and 1 mM sodium pyruvate, warmed to 37 °C. Buffy coat samples were thawed in a 37 °C water bath for approximately 2 min until a small ice crystal remained. Then, 1 ml TTM at 37 °C supplemented with 25 U ml^−1^ benzonase was added dropwise to the thawed sample. The cells were centrifuged at 600*g* for 8 min at room temperature. The supernatant was removed, and the cells were resuspended in 10 ml of TTM at 37 °C supplemented with 25 U ml^–1^ benzonase. The cells were incubated for 15 min at 37 °C. The cells were then centrifuged at 300*g* for 8 min at room temperature, and resuspended in 1 ml of FBS at 37 °C. The concentration of the cell suspension and viability was assessed using the TC20 Cell Counter (Bio-Rad) with trypan blue staining. The concentration of the cell suspension was adjusted to 1 × 10^6^ cells per millilitre. The cells were left to recover for 30–60 min at 37 °C, after which CD45^+^ cells were isolated using MACS (Miltenyi Biotec) following the manufacturer’s protocol.

scRNA library preparation and sequencing were performed by the Genomics platform at the Ontario Institute for Cancer Research. From the isolated CD45^+^ single-cell suspension, 4 × 10^3^ cells were used to construct libraries with the Chromium Single-Cell 3′ Reagent Kit with v2 chemistry (10X Genomics) following the manufacturer’s protocols. Single-indexed libraries were sequenced on the Illumina Novaseq platform with the following parameters: read 1 for 26 cycles, i7 index for 8 cycles and read 2 for 98 cycles. CellRanger (v3.0.0) --mkfastq was used to demultiplex BCL files and generate a fastq file for each scRNA-seq sample. Then, --count was used to align the fastq files to the hg38 reference, filter out barcodes for empty gel bead-in-emulsion, and generate a cell unique molecular identifier × gene matrix, which was used in downstream data analyses.

#### Single-cell data quality control, filtering and annotation

The following quality control filters were applied to the total starting dataset of 619,925 cells: a minimum of 300 genes per cell, a minimum of 10 cells per gene and maximum percentage of mitochondrial DNA = 20%, leaving 438,986 cells remaining. Samples were then downsampled within each risk group (young low risk, young high risk, aged low risk and aged high risk) such that the number of male and female individuals was equal within each risk group. Cell-type annotation was performed using BoneMarrowMap^32^. Annotations were grouped to form six cell-type categories: naive T cells, B cells, natural killer cells, dendritic cells, CD14 monocytes and CD16 monocytes. We removed memory T cells from analysis due to the potential for confounding effects introduced by T cell-specific memory, given its similarity to the HSC-iM program.

#### Scoring of HSC-I and HSC-iM signatures to predict IRS

Cell-type-specific pseudobulk profiles were created using decoupler (v2.0.2), with the ‘sum’ mode. Only samples with a minimum of 10 cells and 1,000 total counts were retained. The pseudobulked data were log normalized and scaled using default settings with scanpy.pp.normalize_total, scanpy.pp.log1p and scanpy.pp.scale. The top 200 genes in the HSC-I and HSC-iM signatures were scored on each cell-type-pseudobulk profile using scanpy.tl.score_genes. For each cell type, a generalized linear model was constructed to predict high risk or low risk (high or low risk ~ HSC-I or HSC-iM score + batch + sex + age), and regressions were conducted separately among young samples and aged samples. *P* values from each regression were adjusted for multiple testing using the Benjamini–Hochberg procedure, within the young and aged groups, using a significance threshold of FDR < 0.05.

### Statistical testing and data presentation

Graphpad Prism (v9.2-10.6) was used to plot biological data and perform statistical testing as specified in the respective figure legends. Bioinformatic data were plotted and compared using R (v4.1.0-4.5.2 with packages ggplot2 v3.5.1-4.0.0, dplyr v1.1.4 and tidyverse v2.0.0) or Python3 (v3.11 with NumPy v2.2.5) as indicated in the respective sections in the Methods. Figure panels were assembled and most schematics were created using Microsoft Powerpoint (v16).

### Reporting summary

Further information on research design is available in the Nature Portfolio Reporting Summary linked to this article.

## Online content

Any methods, additional references, Nature Portfolio reporting summaries, source data, extended data, supplementary information, acknowledgements, peer review information; details of author contributions and competing interests; and statements of data and code availability are available at 10.1038/s41586-026-10522-7.

## Supplementary information

Supplementary Information Supplementary Notes 1–6, Supplementary Table legends and Supplementary References

Reporting Summary

Supplementary Tables Supplementary Tables 1–22

Peer Review file

## Source data

Source Data Fig. 1

Source Data Fig. 2

Source Data Fig. 3

Source Data Fig. 4

Source Data Fig. 5

Source Data Extended Data Fig. 1

Source Data Extended Data Fig. 2

Source Data Extended Data Fig. 3

Source Data Extended Data Fig. 4

Source Data Extended Data Fig. 5

Source Data Extended Data Fig. 6

Source Data Extended Data Fig. 7

Source Data Extended Data Fig. 8

Source Data Extended Data Fig. 9

Source Data Extended Data Fig. 10

## Data Availability

Processed scMultiome and scRNA-seq datasets generated in this study are available on the Gene Expression Omnibus (GEO): LT-HSC scRNA-seq, CB HSPC scMultiome, CB xenograft HSPC scMultiome and xenograft progenitor and myeloid scRNA-seq, and bone marrow xenograft TARGET-seq+ datasets at GSE249479. BAM files for CB HSPC scMultiome, CB xenograft HSPC scMultiome and xenograft progenitor and myeloid scRNA-seq are available on the European Genome-Phenome Archive (EGA) at EGAS50000001624 with controlled access as required by institutional guidelines for sequencing data from human participants. Access can be requested from the University Health Network’s Data Access Committee via the EGA portal. Other sequencing datasets have been previously published and have been referenced appropriately in the text, with the following accession codes: EGAS50000001623, GSE289435, EGAC00001000135, GSE120221, GSE139369, GSE190067, GSE98600, GSE124220, GSE196990, GSE235646, EGAS00001007358, GSE180298, GSE137864, S-BSST1524 and GSE219015. All data relating to the Ontario Health Study (OHS) are available on request following access approvals from the OHS (https://www.ontariohealthstudy.ca/for-researchers/whats-available/), which are reviewed by an independent Data Access Committee. OHS data can be accessed by approved users, and cannot be disclosed, transmitted or transferred to unauthorized individuals as per the OHS Data and Biosample Access Policy (2017). A filtered compilation of GOBP signatures (https://download.baderlab.org/EM_Genesets/current_release/Human/symbol/) and signatures from the MSigDB (https://www.gsea-msigdb.org/gsea/msigdb/collections.jsp) were used as described in the Methods. Source data for charts are available as Supplementary Information or Supplementary Tables where possible. Source data are provided with this paper.

## Code availability

Custom code and notebooks underlying analyses are available on Zenodo (https://zenodo.org/records/18850871)^111^.
