# Supplementary material for: Human haematopoietic stem cells remember inflammatory stress
Source: Nature. 2026 May 27;655(8122):458–67. doi: 10.1038/s41586-026-10522-7 (PMC13345971; doi:10.1038/s41586-026-10522-7)
Supplement: Supplementary file 1 — Supplementary Notes 1–6, Supplementary Table legends and Supplementary References [file 41586_2026_10522_MOESM1_ESM.pdf]

nature portfolio

https://doi.org/10.1038/s41586-026-10522-7

Supplementary information

Human haematopoietic stem cells remember inflammatory stress

In the format provided by the authors and unedited

Supplementary Information

Human hematopoietic stem cells remember inflammatory stress

The PDF file includes:

Supplemental Note 1: Validation of Inflammation and Quiescence Meta-Programs
Supplemental Note 2: Computational validation of HSC-I and HSC-II as distinct cell states
Supplemental Note 3: Integration of TARGET-seq+ data from primary and xenografted BM samples and inference of HSC origin
Supplemental Note 4: Inference of Genetic Clonal Groups within Xenograft HSPCs
Supplemental Note 5: Validation of HSC-iM program transmission to downstream progeny
Supplemental Note 6: Gating schema for FACS and flow cytometry of fresh CB and xenograft bone marrow

Supplementary Table Legends

Supplemental Note 1: Validation of Inflammation and Quiescence Meta-Programs

Having independently identified gene expression programs corresponding to inflammation as well as quiescence within our CB LT-HSC scRNA-seq and CB HSPC scMultiome datasets, we sought to combine the analogous signatures from each dataset into meta-programs (Supplementary Fig. 1a). Specifically, we defined Inflammation and Quiescence meta-programs by calculating the geometric mean of the feature weights from each dataset, revealing specific genes that drive priming towards inflammation and quiescence within cord blood HSC/MPP (Supplementary Fig. 1b).

In order to validate that these meta-programs truly correspond to quiescence and inflammation, we turned to externally-defined gold standard signatures defining quiescence (Quiescent HSC vs Activated HSC, hereafter qHSC vs aHSC)[1] and inflammation (TNF signaling via NF-kB). Interestingly, when comparing the 200 gene TNF signaling via NF-kB hallmark pathway and the 250 top differentially expressed genes comprising the qHSC vs aHSC signature, we observed 31 overlapping genes. To decouple quiescence and inflammation, we removed these overlapping genes from the signatures to generate TNF-specific and quiescence-specific signatures (Supplementary Fig. 1c). Using these new signatures, we demonstrated that the Inflammation meta-program is more enriched for a TNF-specific signature while the Quiescence meta-program is more enriched for a quiescence-specific signature (Supplementary Fig. 1d).

Moreover, we showed that the Quiescence meta-program exhibited stronger enrichment for quiescence programs from muscle stem cells[2] as well as genes associated with low hematopoietic output from lineage tracing of murine HSCs[3] (Supplementary Fig. 1e). Furthermore we found that the Inflammation meta-program exhibited stronger enrichment for signatures upregulated by acute TNF treatment in mature leukocytes[4] as well as murine HSCs[5] (Supplementary Fig. 1f).

To evaluate the extent to which these signatures varied within and between individual CB donors, we re-analyzed scRNA-seq data from two additional studies with CB HSPCs[6,7]. Single cell transcriptomes from each dataset were projected onto BoneMarrowMap[8] and cells annotated as HSC or MPP were utilized for downstream analysis. We scored HSC/MPP from each dataset for the Inflammation and Quiescence meta-programs using AUCell[9] and evaluated the enrichment of each meta-program by donor (Supplementary Fig. 1g). This revealed a variability in meta-program expression within the HSC/MPP pool of individual donors, alongside further heterogeneity between donors with some exhibiting systematically higher enrichment of Inflammation or Quiescence signatures (Supplementary Fig. 1h). Collectively, these data demonstrate that the Inflammation and Quiescence programs are transcriptionally distinct from each other, and exhibit varying degrees of priming in CB HSC/MPPs both within and across donors.

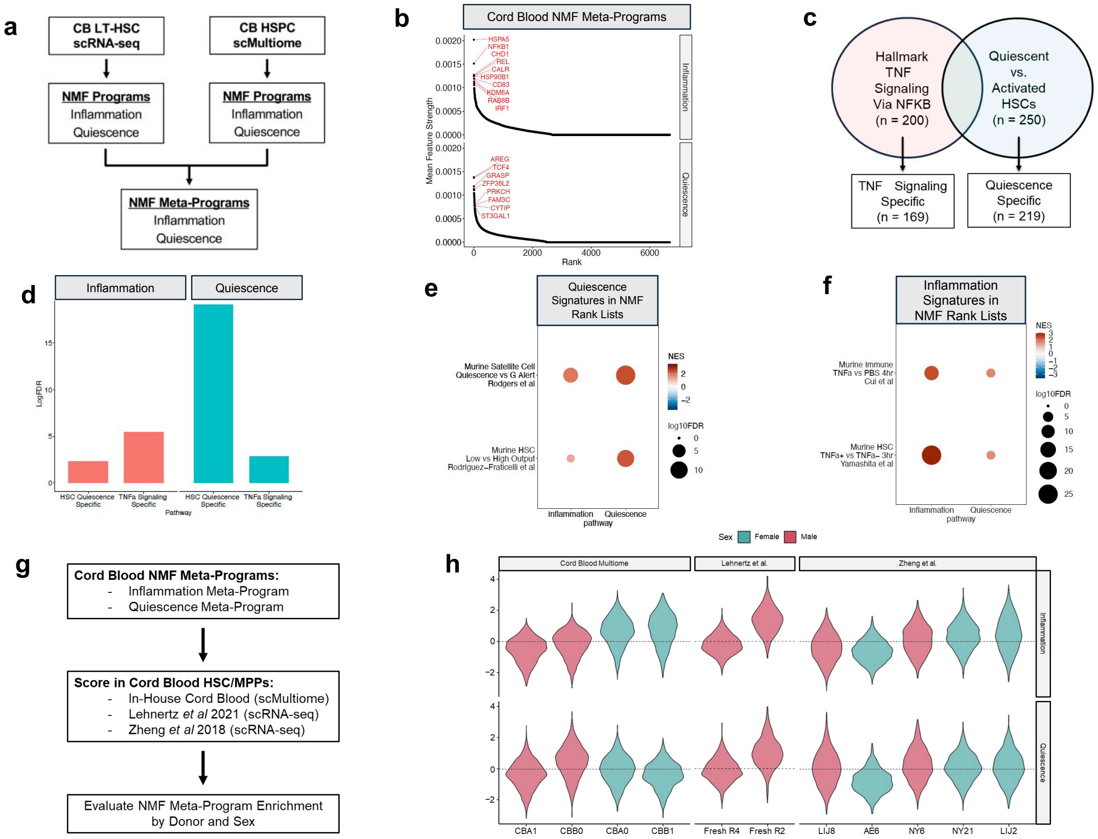

Supplementary Figure 1: Validation of inflammation and quiescence meta-programs. a, Schematic outlining NMF meta-program derivation from the LT-HSC scRNA-seq and CB HSPC scMultiome datasets. **b,** The top genes of a consensus NMF meta-program derived by the geometric mean of feature weights across the quiescence and inflammation signatures from the LT-HSC scRNA-seq and HSPC scMultiome datasets depicted using a scatter plot of mean feature strength on the y-axis and corresponding feature ranking on the x-axis. **c,** Venn diagram depicting the derivation of quiescence specific and TNF signaling specific signatures via the intersection of the qHSC vs aHSC signature and the Hallmark TNF Signaling Via NF-kB signature. **d,** Barplot depicting the LogFDR (-log10(padj)) of an over-representation analysis based on a hypergeometric test using the signatures derived in (C) as a geneset. **e,** GSEA results depicting enrichment of stem cell quiescence signatures in a ranking of the mean feature strengths of the quiescence and inflammation meta-program. **f,** GSEA results depicting enrichment of TNF signatures in a ranking of the mean feature strengths of the quiescence and inflammation meta-program. **g,** Schematic outlining analysis approach. **h,** Distribution of AUCell enrichment score (AUC) for the top 200 genes driving the Inflammation and Quiescence meta-programs in CB HSC/MPPs by donor and sex across three datasets.

Supplemental Note 2: Computational validation of HSC-I and HSC-II as distinct cell states

Multiple other computational approaches were employed to show HSC-I and HSC-II represent distinct transcriptional and epigenetic states in the context of the inflammation-recovery model to complement the weighted nearest neighbor analysis in the main manuscript. Parallel analyses with alternative scRNA and scATAC analysis pipelines including OCAT (Supplementary Fig. 2a-c)[10], TooManyCells (Supplementary Fig. 2d-f)[11], and TooManyPeaks (Supplementary Fig. 2g-i)[12] provided further support that HSC-I and HSC-II represent distinct cellular states. Indeed, differential accessibility (DA) analysis between HSC-I and HSC-II revealed 2,112 differentially accessible regions (DARs) specific to HSC-II (Supplementary Fig. 2j and Supplementary Table 7), as well as global changes in TF binding site accessibility (Supplementary Fig. 2k) and specific enrichment for JUN/FOS motifs from the AP-1 family (Supplementary Fig. 2l,m) in HSC-II. Importantly, HSC-I and HSC-II also separated along defined PCA and latent semantic indexing (LSI) components from dimensionality reduction of gene expression and chromatin accessibility, respectively. Finally, pathway analysis revealed diverse biological pathways significantly enriched in HSC-II, spanning immune signaling, proteostasis and stress responses, and regulation of cell cycle and cell motility, whereas no biological pathways were significantly enriched in differentially expressed genes (DEGs) specific to HSC-I. Thus, we can conclude that HSC-I and HSC-II represent distinct cell states as defined by global gene expression and chromatin accessibility differences.

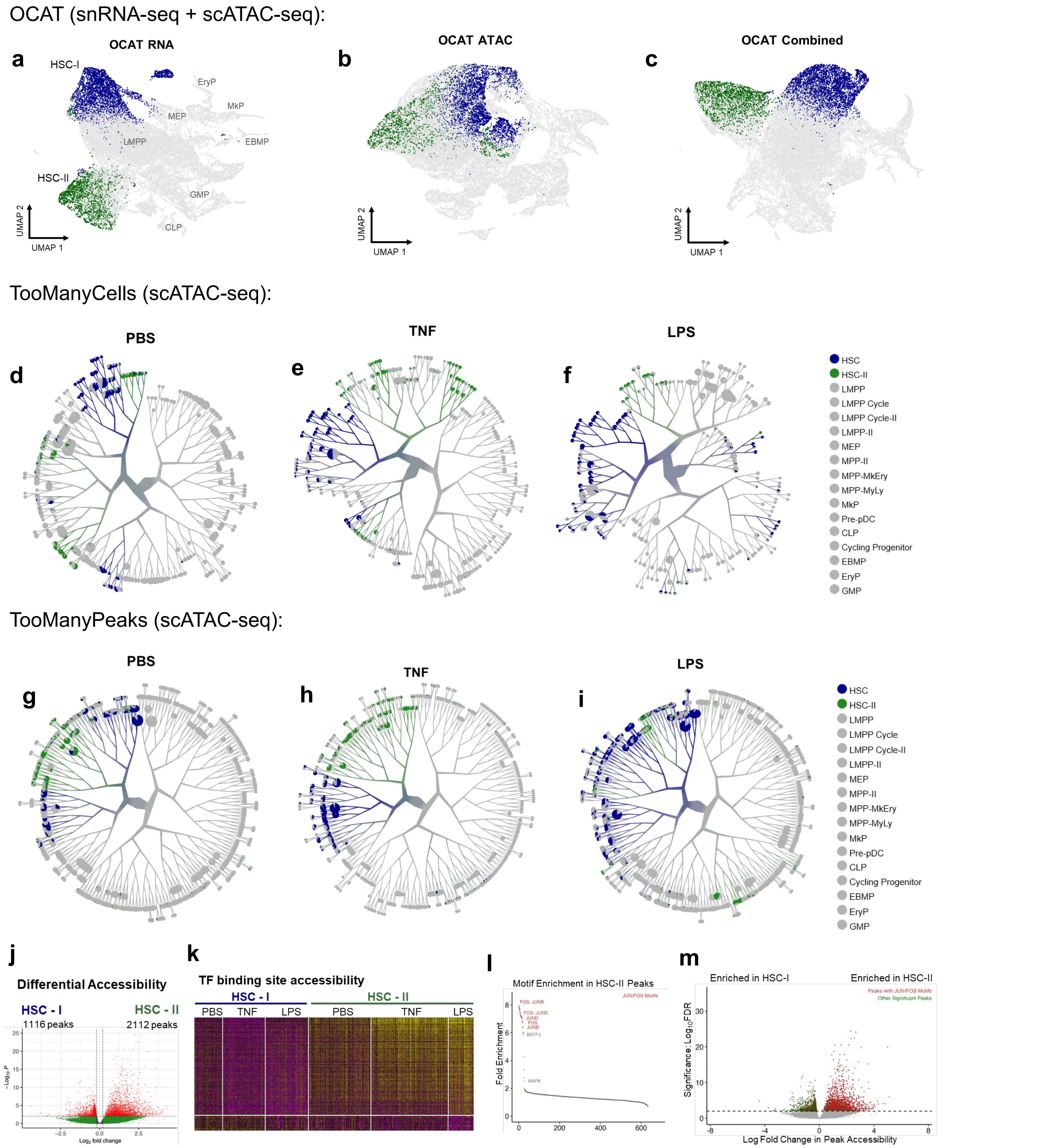

Supplementary Figure 2: Computational validation of HSC-I and HSC-II as distinct cell states. a-c, Alternative reduced dimensional embeddings through One Cell At a Time (OCAT), which constructs "ghost" cells based on neighborhood centroids and generates a sparse encoding for each cell in the dataset based on its proximity to each "ghost" cell, prior to downstream UMAP construction. These embeddings were generated on the basis of gene expression (a), chromatin accessibility (b), and combined gene expression + chromatin accessibility (c). HSC-I and HSC-II are colored in blue and green, respectively, within each embedding. **d-f,** Cell clade visualization from gene expression data by TooManyCells, shown for cells from each treatment condition. Clades containing HSC-I and HSC-II are colored in blue and green, respectively. **g-i,** Cell clade visualization from chromatin peak accessibility by TooManyPeaks, shown for cells from each treatment condition. In this analysis, the input is the chromatin peak matrix called by MACS2 also used as an input for classical scATAC analysis by Signac. Clades containing HSC-I and HSC-II are colored in blue and green, respectively. **j,** Differentially accessible (DA) peaks between HSC-II and HSC-I, performed by creating pseudo-bulks by donor sex and treatment condition. Significant peaks were called at an FDR < 0.05. **k,** Heatmap of DA transcription factor binding sites (TFBS) from ReMap2020; normalized chromVAR enrichment scores are shown. **l,** Fold enrichment of TF motifs, from the JASPAR database, within DA peaks specific to HSC-II. **m,** Presence of JUN-FOS motifs within DA peaks between HSC-II and HSC-I.

Supplemental Note 3: Integration of TARGET-seq+ data from primary and xenografted BM samples and inference of HSC origin

To test the functional characteristics of HSC-I and HSC-iM *in vivo*, we performed xenotransplantation experiments with samples from our adult BM cohort (mix of CH and non-CH) where we had previously characterised the proportion of HSC-I/HSC1 and HSC-iM/HSC2 using TARGET-seq+. We created a modified xenograft challenge model using NSG-SGM3 immunodeficient mice, that allows for better myeloid differentiation output than NSG mice. Mice were treated with PBS or hTNF at 2w and 10w post-transplantation and sacrificed at 12w post-transplantation where human engraftment was assessed and bone marrow cells were viably frozen for downstream analysis (Extended Data Fig. 9a).

CD34+ HSPC and CD33+ progeny were isolated from the xenografts and processed for TARGET-seq+ analysis. In parallel, cells from the NOC153 primary BM sample were sorted onto each plate, serving as a technical control. Pre-processed FACS indexing, genotyping and transcriptome data were integrated for downstream analysis in Seurat (v5.3.0). The filtered dataset of 8,998 cells was integrated with the 13,247 primary BM cells from the original TARGET-seq+ dataset[14]. Batch correction with harmony (v1.2.3) was performed with Sample donor and experimental batch as batch variables, followed by UMAP reduction and Louvain clustering. Clusters were annotated and condensed by concordance with predicted cell type annotations upon mapping to the BoneMarrowMap[15] along with the cell type annotations from the original primary BM dataset and immunophenotypic profiles (Supplementary Figure 3a-b). Effective batch correction between the original and new TARGET-seq+ dataset was confirmed by visualisation of primary cells from the NOC153 control sample which were processed in both batches (Supplementary Figure 3c).

To identify HSC-I and HSC-iM derived cells within the xenografts, we exploited our observation that the HSC transcriptional programs were transmitted to downstream progeny in primary BM samples (Fig. 6a and 6b). We first defined a set of genes that distinguish the differentiation trajectories of HSC-I and HSC-iM dominant primary BM samples in the original TARGET-seq+ dataset. We performed differential expression analysis between HSC-I and HSC-iM dominant samples within each cell type, and selected genes that were differentially expressed at FDR < 0.10 within ≥ 5 cell types (Supplementary Figure 3d). This generated a list of 702 genes that distinguish HSC-I, HSC-iM and their progeny.

We then performed dimensionality reduction and clustering of HSPCs and monocytic cell types in the integrated TARGET-seq+ dataset using the Self-Assembling Manifolds (SAM) algorithm and the 702 HSC-I/HSC-iM trajectory genes (Supplementary Figure 3e-f). Visualisation of the original primary BM cells (where HSC-I vs HSC-iM composition was known) on the resulting UMAP showed effective separation of HSC-I, HSC-iM and their downstream progeny (Supplementary Figure 3g). Louvain clustering was performed on the SAM embeddings at a resolution of 1.5, and the resulting 15 clusters were annotated as being part of the HSC-I or HSC-iM differentiation trajectory based on their composition of primary BM cells from HSC-I and HSC-iM dominant samples and expression of the HSC-I and HSC-iM transcriptional programs (Supplementary Figure 3h). We used this categorization to infer whether xenograft cells were of HSC-I or HSC-iM origin (Supplementary Figure 3i).

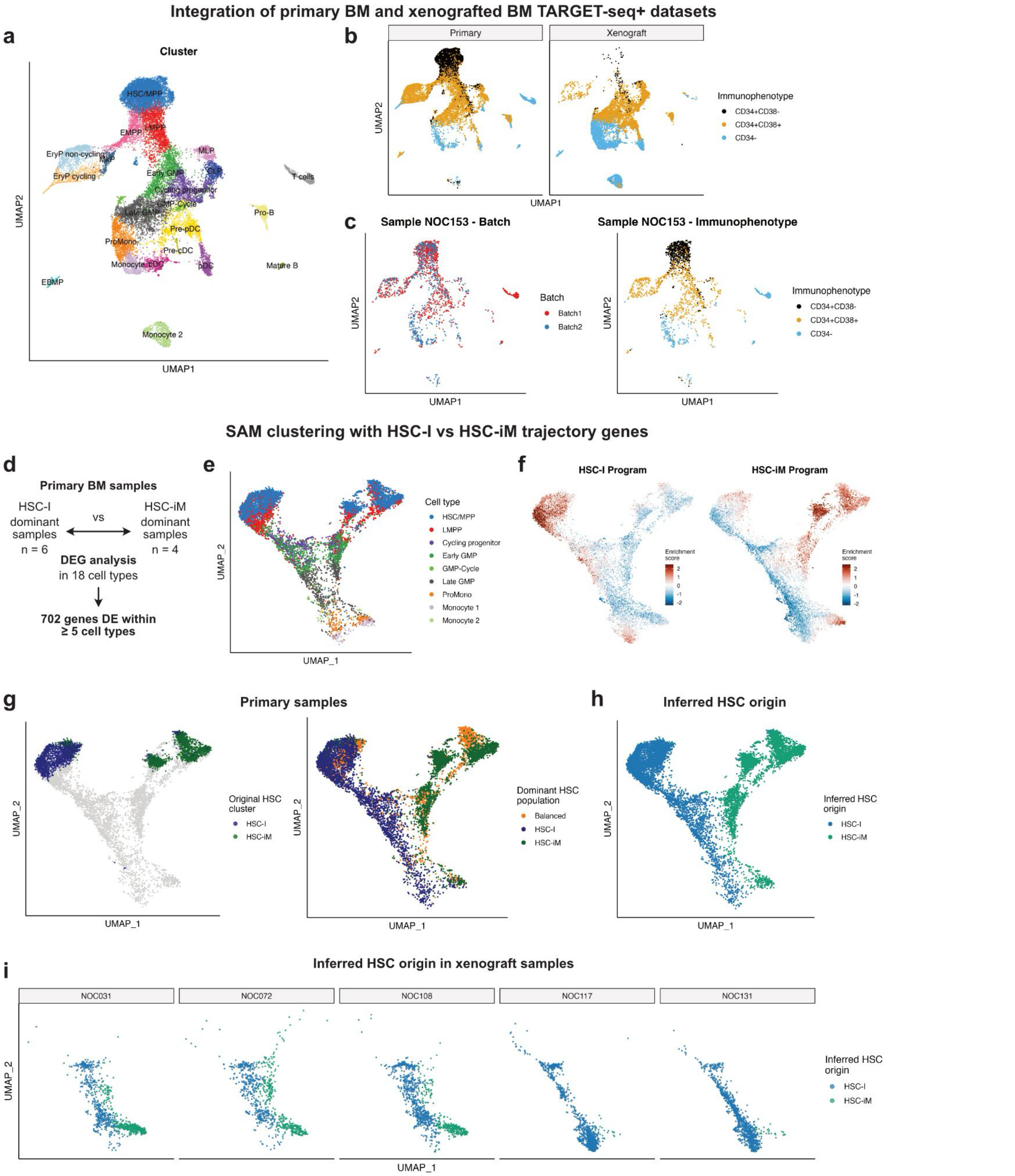

Supplementary Figure 3: Integration of TARGET-seq+ data from primary and xenografted BM samples and inference of HSC origin. a-c, Integration of TARGET-seq+ data from the original primary BM dataset with CD34+ HSPC and CD33+ progeny isolated from xenografted BM samples. UMAP visualisation following Harmony batch correction. **a,** Cell type annotations in the integrated TARGET-seq+ dataset. **b,** Immunophenotype of primary BM and xenografted BM cells in the integrated dataset. **c,** Visualisation of cells from the NOC153 primary BM sample, showing distribution of cells in each experimental batch (left) and their immunophenotype (right). **d-i,** Inference of HSC origin in xenografted samples. 14,546 HSPCs and monocytic cells from the primary and xenografted samples were subclustered with the Self-Assembling Manifolds (SAM) algorithm using genes differentially expressed between HSC-I and HSC-iM dominant primary BM samples. The SAM-weighted principal components were used to generate UMAP projections. **d,** Differential expression analysis between HSC-I and HSC-iM dominant samples in 18 cell types identified 702 genes used for subclustering. **e,** UMAP generated from SAM subclustering, showing cell type annotations from (A). **f,** Enrichment of the HSC-I and HSC-iM programs in the integrated dataset following SAM subclustering using genes

differentially expressed between HSC-I and HSC-iM dominant primary BM samples. **g,** UMAP of 9,295 cells from the primary BM samples, showing HSC-I and HSC-iM cells (left) and whether cells originate from a sample with a dominant HSC-I, dominant HSC-iM or balanced HSC population (right). **h,** Annotation of the inferred HSC origin. **i,** UMAPs showing the 5,251 cells from the xenografted samples, separated according to the sample donor and coloured by the inferred HSC origin.

Supplemental Note 4: Inference of Genetic Clonal Groups within Xenograft HSPCs

We sought to leverage the genetic heterogeneity inherent within our xenograft experiment as genetic barcodes to link individual HSCs to their likely downstream progeny. In terms of genetic heterogeneity, our xenograft-recovery models were established with three separate pools of CB constructed from a total of 62 unique donors. Each pool was transplanted into up to 15 mice, with up to 5 mice assigned to receive repeated challenges with PBS, hTNF, or LPS. Viably frozen samples from each pool were thawed to isolate the CD34+CD38-CD45RA- cells (n=41 xenografted mice; 13 in PBS group, 15 in hTNF group, 13 in LPS group) for downstream 10x scMultiome library generation in a single day. The HSPC scMultiome dataset (27,492 CD34+CD38-CD45RA- cells), the Progenitor scRNAseq dataset (34,755 CD34+CD38+ cells), and the Myeloid scRNAseq dataset (20,280 CD33+ cells) were derived from 62 CB donors spanning 41 xenografted mice across 3 treatment conditions.

We exploited this genetic heterogeneity within the inflammation recovery xenograft model in order to track the lineage output of HSC-iM. To this end, we utilized SouporCell[13], a tool that identifies expressed genetic polymorphisms from the raw sequencing data within a single cell experiment and performs unsupervised clustering of single cells based on their genetic similarity. To link HSCs to downstream progeny based on shared genetic polymorphisms within our xenograft models, we combined raw sequencing data from the CD34+CD38-CD45RA- HSPCs with newly generated scRNA-seq data from 20,280 CD33+ mature myeloid cells and 34,755 CD34+CD38+ progenitor cells isolated from these same 20w xenografts using pysam (v0.15.1). Resulting merged bam files were indexed and sorted using samtools (v1.17) with default parameters. A parameter search identified k = 15 clusters as having the highest log-probability in this dataset, with decreasing log-probability as the number of clusters increased (Supplementary Fig. 4a-b). We also confirmed that k = 15 clusters resulted in the highest concordance with inferred donor sex of each cell (Supplementary Fig. 4c-d).

In unsupervised clustering, more cells are required to confidently cluster at a higher resolution, and it is likely that k = 15 reflects the maximum resolution for polymorphism-based clustering given the number of cells that we have profiled. Thus, we were able to define a total of 15 genotype-based clusters within this new data analysis, each likely reflecting cells derived from groups of 1-5 donors with genetically similar backgrounds. Given that each genotype-based cluster represents a group of distinct clones present within the xenograft, we refer to them as clonal groups for downstream analyses.

Evaluation of cell composition for each of the clonal groups revealed heterogeneity in HSC state preference and thus clonal groups were assigned to the following classes: HSC-I Specific, HSC-iM Specific, and Non-Specific (Extended Data Figure 9a). As each clonal group represents anywhere from a single donor to a small group of genetically similar donors, the identification of multiple clonal groups specific to either HSC-I or HSC-iM demonstrates that these HSC populations are not driven by a single outlier CB donor, but also that these are not derived from every CB donor. Moreover, each clonal group including HSC-I specific and HSC-iM specific groups were identifiable within PBS, hTNF, and LPS treatment conditions (Extended Data Figure 9b), suggesting that (1) each HSC subset exists across multiple mouse transplant recipients, and (2) the formation of HSC-I and HSC-iM populations within the xenograft model may be deterministic and precedes the inflammatory challenges (Extended Data Figure 9b). Last, the identification of HSC-iM and HSC-I specific clonal groups suggests that these can be exploited as barcodes for downstream lineage tracing.

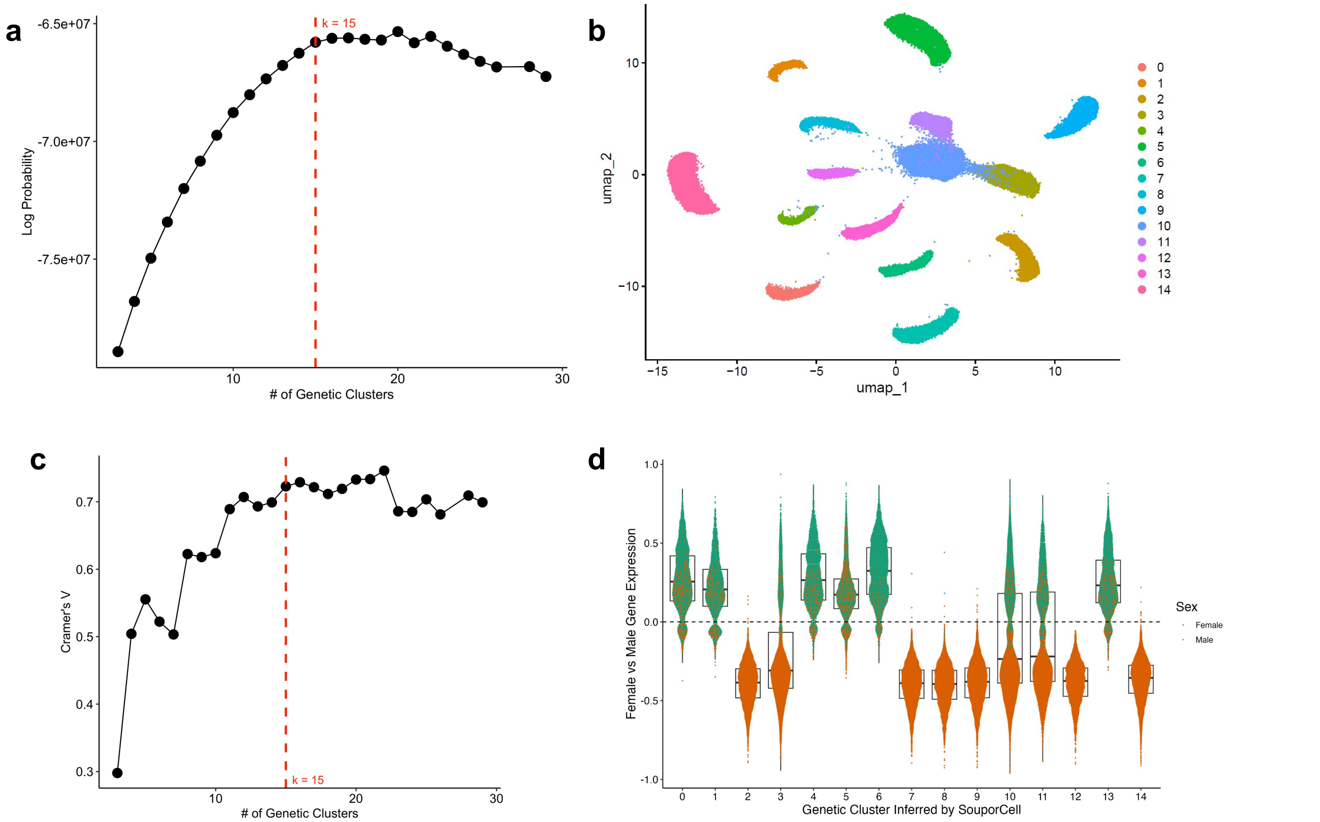

Supplementary Figure 4: Inference of genetic clonal groups within xenograft HSPCs. a, Elbow plot of the total log likelihood versus the number of genetic clusters. **b,** UMAP of the normalized cell-by-cluster log-likelihood matrix from SoupOrCell (n = 82,527). **c,** Scatter plot depicting the Cramer's V metric indicating the concordance with predicted sex annotations across each SoupOrCell run. **d,** Violin plot depicting the Female vs Male Gene expression and predicted sex across each genetic cluster inferred by SoupOrCell for k = 15.

Supplemental Note 5: Validation of HSC-iM program transmission to downstream progeny

To validate our findings that downstream progeny derived from HSC-iM retain inflammatory memory and pro-inflammatory properties, we sought to demonstrate this through orthogonal analysis of three additional datasets. For these analyses, we focused specifically on Monocytes, which are (1) short-lived, and (2) known mediators of peripheral inflammation in CH[16,17].

First, we interrogated the COVID recovery data. Within this dataset, we observe that HSCs from patients who had endured severe COVID infection 2-3 months prior to sample collection (ICU-COVID) were enriched for the HSC-iM program relative to HSCs from healthy controls and HSCs from ICU controls. Therefore, we assessed gene expression within monocytes from the same patients, comparing monocytes from ICU-COVID patients against healthy controls and ICU-recovery controls. Within monocytes from ICU-COVID patients compared to control monocytes, we observed upregulation of the HSC-iM program alongside TGF-Beta signaling and inflammatory programs spanning TNF via NF-kB signaling, IL6 signaling, and IFNγ signaling, with downregulation of the HSC-I program (Extended Data Fig. 10a). This was in striking concordance with the observed changes in ICU-COVID HSCs, thus further supporting the notion of heritability of HSC-iM programs in downstream progeny.

For additional validation, we leveraged data from a native lineage tracing approach within human HSCs utilizing mitochondrial DNA (mtDNA) variants as genetic barcodes which was recently published by the Sankaran Group[18]. Given the transcriptional upregulation that we have observed in HSC-iM derived monocytes based on our CH, xenograft, and COVID datasets, we evaluated how HSC-iM program enrichment within upstream HSCs correlated with transcriptional features of downstream monocytes. To this end, we identified 213 mtDNA-based clones each containing ≥ 3 HSCs as well as ≥ 3 monocytes, spanning 13 donors from their study. For each mtDNA-based clone, we calculated the extent of HSC-iM program enrichment within the HSC compartment and used this score as a continuous variable for differential expression analysis using DESeq2 within the downstream monocytes (Supplementary Fig. 5a). The result was a weight for each gene within the monocyte transcriptome based on their clone-level association with HSC-iM program enrichment in the HSCs from which they descended.

Evaluating association strength from this analysis revealed *NFKB1* to be the top associated gene, meaning that *NFKB1* expression was higher among monocytes linked to HSCs with stronger HSC-iM program enrichment at the level of mtDNA-based clones (Supplementary Fig. 5c). Furthermore, GSEA performed on monocyte transcriptomes revealed that among mtDNA-defined clones with higher HSC-iM program enrichment among their HSCs, the monocytes from those same clones also exhibited enrichment for the HSC-iM program alongside other pro-inflammatory signatures including TNF via NF-kB signaling, known NFKB1/NFKB2 targets[19], and genes upregulated upon inflammatory activation of monocytes with LPS or TREM1 challenge[20] (Supplementary Fig. 5d). For further validation, we performed the same across 3 public datasets in the BoneMarrowMap cohort[8,21–23] by correlating mean HSC-iM program enrichment among HSCs from 32 donors with transcriptional features of monocytes from the same donors. These analyses yielded the same results, with stronger HSC-iM enrichment within the HSC pool correlating with pro-inflammatory properties of downstream monocytes within those same donors (Supplementary Fig. 5b,d).

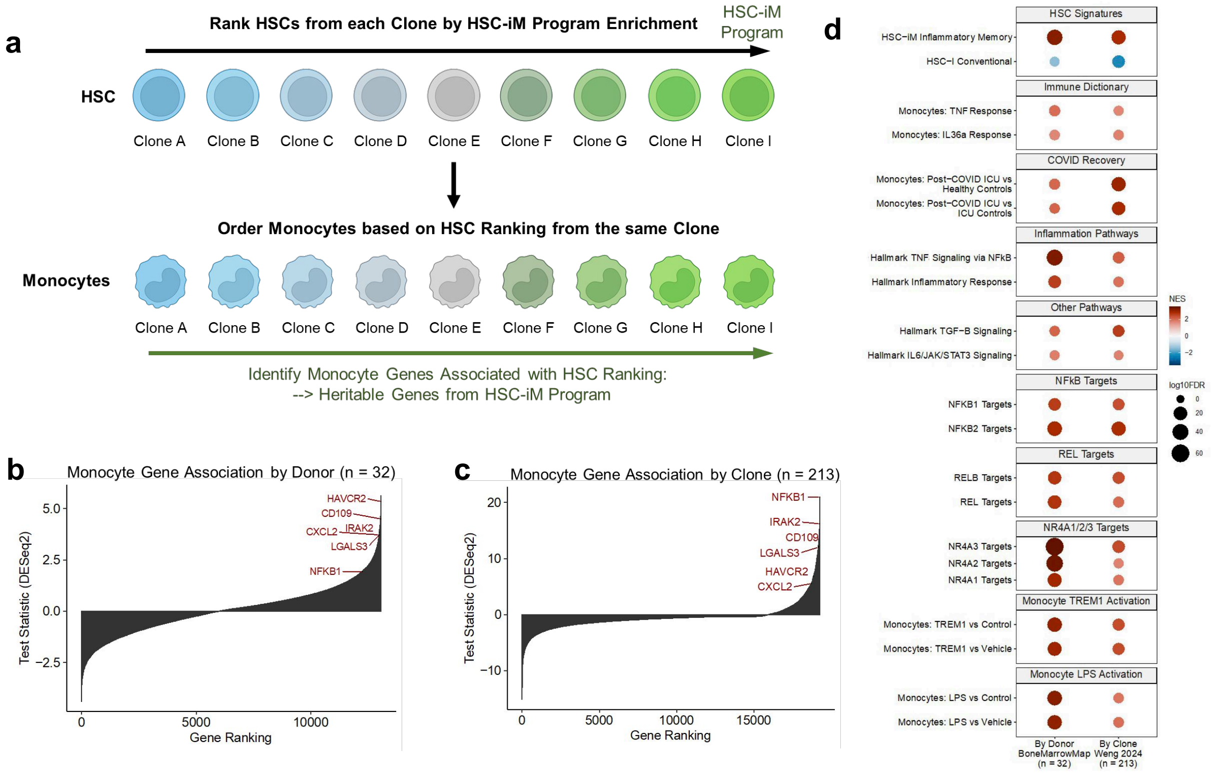

Supplementary Figure 5: Validation of HSC-iM program transmission to downstream progeny. a, Schematic outlining association analysis of HSC-iM program enrichment among HSCs with transcriptional features of downstream monocytes. **b-c,** Association of HSC-iM program strength in HSCs with transcriptional features of downstream monocytes across donors (n = 32; BoneMarrowMap) (b) and across mtDNA-defined clones (n = 213; Weng et al) (c). Genes are ranked by test statistic from differential expression using HSC-iM program strength among HSCs as a continuous variable. **d,** GSEA of signatures and pathways along monocyte transcriptomes from this analysis.

Supplemental Note 6: Gating schema for FACS and flow cytometry of fresh CB and xenograft bone marrow

The following populations were isolated from umbilical cord blood (CB): long-term hematopoietic stem cells (LT-HSC) for single-cell (sc) RNA sequencing (RNAseq), as presented in Figure 1a-e; progenitors for scMultiome (ATAC+RNA sequencing) as presented in Extended Data Figure 1a-h; and CD34+CD38− for xenografting as in Figure 1f,k and Extended Data Figure 2q. These populations are exemplified in the gating schematic in Supplementary Figure 6a.

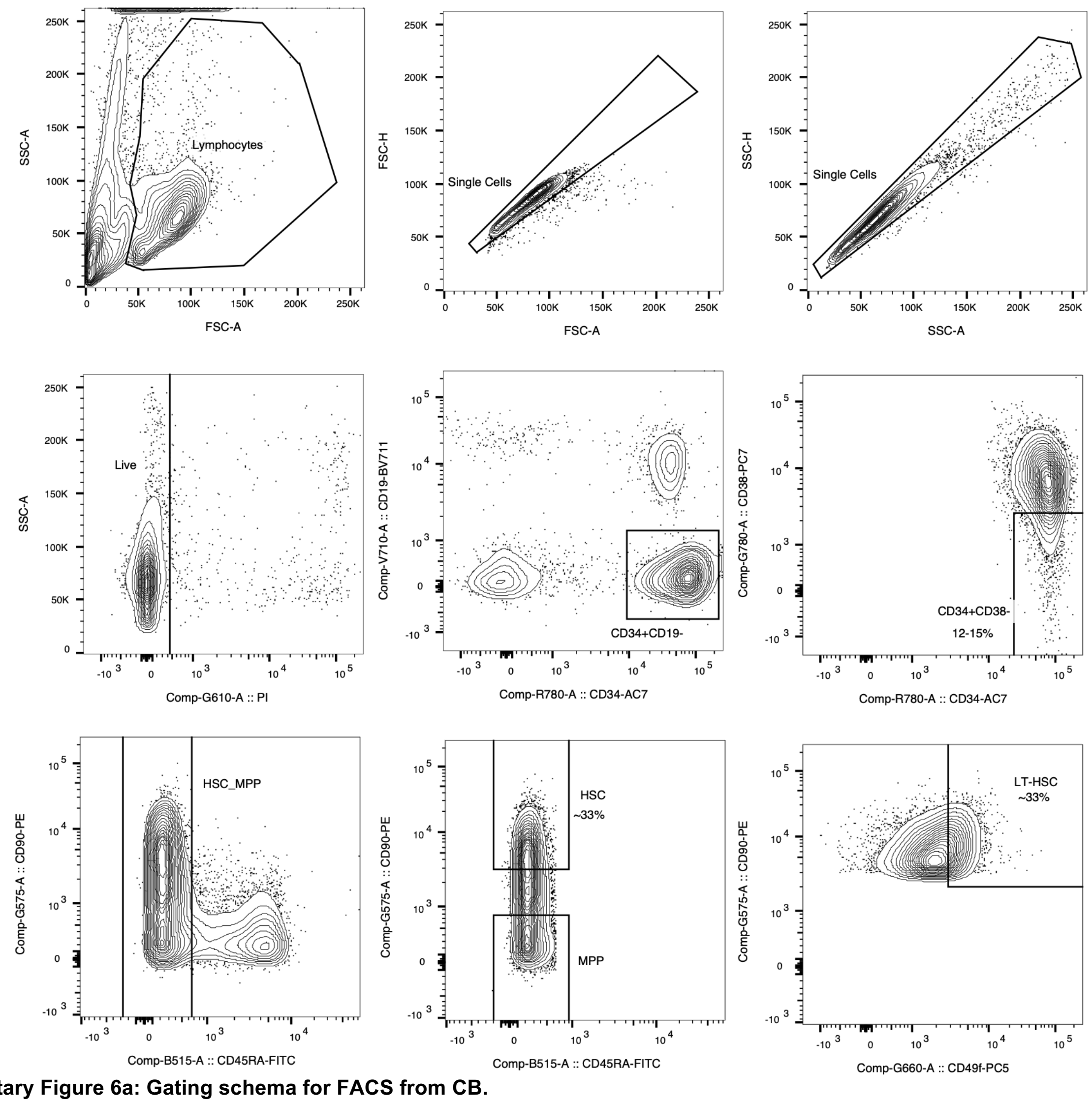

Supplementary Figure 6a: Gating schema for FACS from CB.

Human hematopoietic populations were evaluated in each xenografted mouse in individual experiments as indicated in Figure 1f-s, Extended Data Figure 2, and Extended Data Figure 8b,c per the generalized gating schema shown in Supplementary Figure 6b.

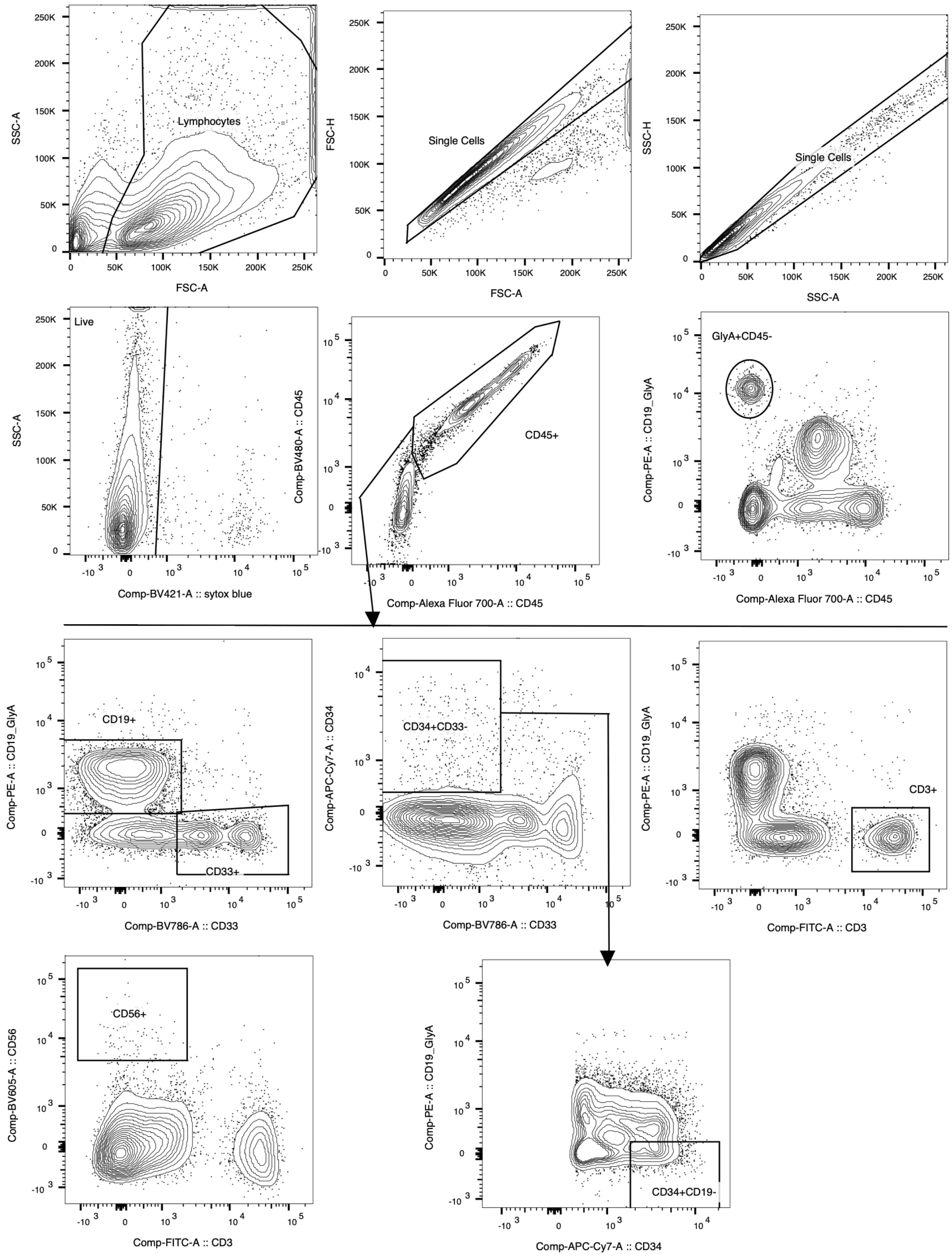

Supplementary Figure 6b: Gating schema for flow cytometry of xenograft-derived hematopoietic progeny; CD33+ cells are denoted as 'Myeloid', CD19+ cells as 'B cell', CD3+ as 'T cell', CD56+ as 'NK cell', and CD34+CD33-CD19- as 'CD34'.

The following human populations were isolated by FACS from pooled CB xenograft-derived bone marrow: HSC/MPP for scMultiome as in Figure 2a; CD33+ Myeloid and CD34+CD38+ Progenitors for scRNAseq as in Figure 5d; CD45+ leukocytes for secondary transplant as in Figure 1p and Extended Data Figure 2i; and CD45+CD19-CD33- 'T cell enriched' for culture as in Extended Data Figure 2n. These populations are exemplified in the gating schematic in Supplementary Figure 6c.

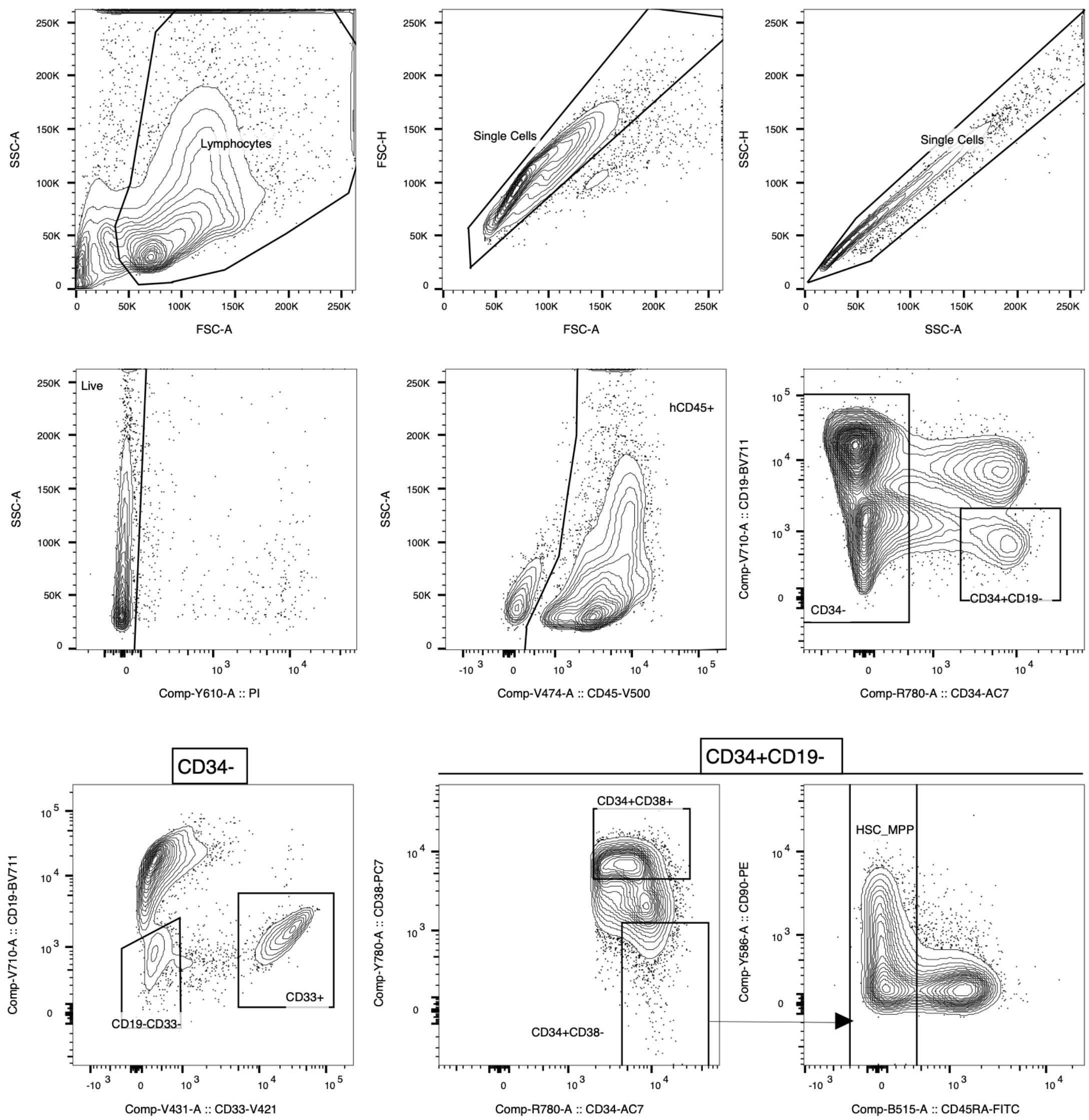

Supplementary Figure 6c: Gating schema for FACS from CB-derived xenograft bone marrow; CD33+ are 'Myeloid' and CD34+CD38+ are 'Progenitors' and CD45+CD19-CD33- are 'T cell enriched'.

Finally, BM-derived xenograft bone marrow was pre-sorted by FACS as exemplified in Supplementary Figure 6d for CD19- CD33+ and/or CD33-CD34+ human populations, which were then index sorted for TARGET-seq+ as for Extended Data Figure 8.

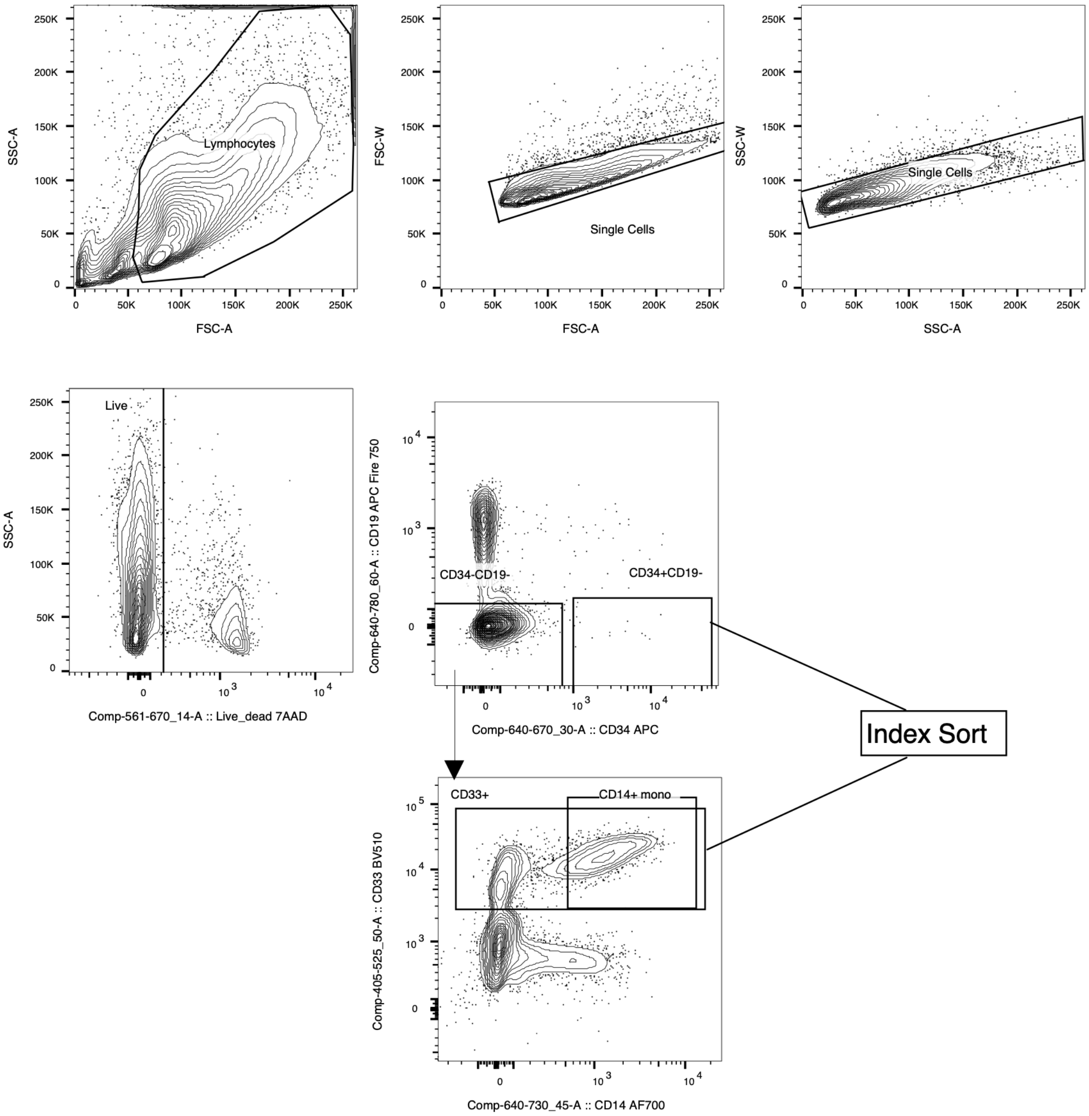

Supplementary Figure 6d: Gating schema for pre-sorting from BM-derived xenograft bone marrow.

Supplementary Table Legends

Supplementary Table 1 | Human HSC signatures
Gene lists of literature-derived normal HSC gene expression signatures generated by differential expression (Log Fold change >= 1, padj <= 0.05) for enrichment analysis. Signatures of quiescent HSC vs activated HSC (qHSC vs aHSC) were derived from Garcia-Prat *et al* 2021. Signatures of LT-HSC vs MPP, LMPP, and Progenitor (defined as MPP, LMPP, MLP, MEP, GMP, CMP) were derived from sorted CB fractions from Xie *et al* 2021. HSC 49f My-Meg-Ery signature was derived from the CD34^{lo}CLEC9A^{hi} fraction within CD34+CD38-CD45RA-CD49f+ HSCs from Belluschi *et al* 2018.

Supplementary Table 2 | LT-HSC cNMF programs
A list of genes ranked by the z-score coefficients from 7 gene expression programs inferred using consensus non-negative matrix factorization (cNMF) of the single cell (sc) RNA-seq of 3,381 Lin-CD34+CD38-CD45RA-CD90+CD49f+ cord blood long-term HSCs (LT-HSCs).

Supplementary Table 3 | Cord blood scMultiome cNMF programs
A list of genes ranked by the z-score coefficients from 6 gene expression programs inferred using cNMF of the scRNA-seq of the cord blood scMultiome HSC/MPPs purified *in silico* by projection on the BoneMarrowMap.

Supplementary Table 4 | NMF Meta-programs
Meta-programs of Inflammation and Quiescence constructed from integration of NMF signatures derived from CB LTHSC scRNAs-seq and CB HSC/MPP scMultiome data in Supplementary Tables 2 and 3. Integrated feature weights for each of the Quiescence and Inflammation meta-signatures are represented as the geometric mean of the feature weights from each component dataset.

Supplementary Table 5 | Multiplex Cytokine profiling at 6 Weeks Xenotransplantation
Blood plasma from NSG mice transplanted with human CB-derived blood progenitors was sampled 6w post-transplant, then challenged with intraperitoneal injection of PBS or LPS, and blood plasma sampled again 16hrs later. Human (23) and mouse cytokines (18) were measured using a multiplex assay (EVE technologies). Paired data for individual mice is shown along with lower limits of detection for each cytokine.

Supplementary Table 6 | LDA
Summary of limiting dilution analysis (LDA) of xenograft bone marrow-derived hCD45+ leukocytes in secondary transplant recipients to measure stem cell frequency retained at 20 weeks post-graft after single or repeated inflammatory challenge.

Supplementary Table 7 | Cord blood xenograft marker genes
Marker genes for each cell type in the cord blood xenograft scMultiome with marker score representing the geometric mean of sc-AUROC, sc-logFC, DESeq-stat, DESeq-logFC.

Supplementary Table 8 | Xenograft HSC-II vs HSC-I differentially accessible regions
Differentially accessible chromatin peaks between HSC-II and HSC-I in the cord blood xenograft scMultiome.

Supplementary Table 9 | Xenograft HSC-II vs HSC-I differentially expressed genes

Differentially expressed genes between HSC-II and HSC-I in the cord blood xenograft scMultiome.

Supplementary Table 10 | SCENIC+ HSC-II vs HSC-I
Differentially enriched TF eRegulons between HSC-II and HSC-I, stratified by treatment condition. Only TFs where both RNA and ATAC eRegulons were significantly enriched in the same direction at a significance threshold of FDR < 0.05 are shown.

Supplementary Table 11 | Multiplex Cytokine profiling of plasma from 20 Weeks Xenotransplantation after repeat challenge and recovery
Blood plasma from NSG mice transplanted with human CB-derived blood progenitors and challenged twice with PBS, TNF or LPS was sampled 20w post-transplant. Human (21) and mouse cytokines (18) were measured using a multiplex assay (EVE technologies). Data for individual mice is shown along with lower limits of detection for each cytokine.

Supplementary Table 12 | SCENIC+ by condition
Differentially enriched TF eRegulons between PBS, TNF, and LPS conditions stratified by HSC subtype. Only TFs where both RNA and ATAC eRegulons were significantly enriched in the same direction at a significance threshold of FDR < 0.05 are shown.

Supplementary Table 13 | Immunological signatures
Genes comprising signatures of immune memory derived by evaluating differential expression within murine/human hematopoietic stem cells (HSC) following BCG vaccination, CD4 and CD8 memory T cells compared to effector/naive, and between ICU-COVID HSC vs HSC from Healthy and ICU Controls.

Supplementary Table 14 | Aging signatures
Gene lists of expression signatures of aging derived by differential expression between older/middle adult HSCs vs young adult HSCs from Ainciburu et al 2023, Zhang et al 2022, BoneMarrowMap, and our in-house Bone Marrow scMultiome. The human HSC datasets were also integrated and differential expression was performed between middle age/older adult vs young adult HSC adjusting for dataset as a covariate, resulting in a 37-gene aged HSC meta-signature with genes as listed in this file that passed the following criteria: LFC > 1 and FDR < 0.05.

Supplementary Table 15 | CH signatures
Gene lists of expression signatures from TARGET-seq+ CH dataset from 9 donors with *DNMT3A* or *TET2* mutant CH and 4 age-matched controls without CH. Top genes enriched in HSC2 vs HSC1 after adjusting for sex as a covariate are included, alongside genes enriched in CH wild-type HSC compared to wild-type HSC from non-CH controls, filtered at LFC > 0.5 and FDR < 0.05.

Supplementary Table 16 | HSC2/HSC-iM shared differentially expressed genes
List of concordant differentially expressed genes between HSC-iM (vs HSC-I) from the xenograft scMultiome dataset and HSC2 (vs HSC1) from the CH TARGET-seq+ dataset, including 1456 mutually significantly upregulated genes in both HSC-iM and HSC2.

Supplementary Table 17 | CH mutant vs WT in HSC-I and HSC-II
Differential expression results between CH Mutant vs CH Wild-type HSC within the CH TARGET-seq+ dataset, shown for each HSC subset and for each mutation (*DNMT3A* and *TET2*). Results were generated from a linear mixed model adjusting for sample identifier and FACS sorting plate batch as covariates

using the dream pipeline.

Supplementary Table 18 | HSC-iM vs HSC-I along myeloid differentiation within CH TARGET-seq+ dataset
Differential expression results between HSC-iM-Dominant vs HSC-I-Dominant hierarchies within the CH TARGET-seq+ dataset, shown for each cell type along myeloid differentiation. Results were generated from a linear mixed model adjusting for sample identifier, donor sex, and FACS sorting plate batch as covariates using the dream pipeline.

Supplementary Table 19 | HSC-iM vs HSC-I among progenitors within xenograft dataset
Differential expression results between HSC-iM-specific and HSC-I-specific clonal groups within the xenograft dataset, shown for progenitor cell types. Results were generated by pseudo-bulk based differential expression with DESeq2 wherein cells from each cell type within each clonal group within each treatment condition were combined.

Supplementary Table 20 | HSC-iM vs HSC-I along myeloid differentiation with xenograft dataset
Differential expression results between HSC-iM-specific and HSC-I-specific clonal groups within the xenograft dataset, shown for each cell type along myeloid differentiation. Results were generated by pseudo-bulk based differential expression with DESeq2 wherein cells from each cell type within each clonal group within each treatment condition were combined.

Supplementary Table 21 | HSC-iM correlation with monocyte genes
Differential expression results depicting association between genes within the monocyte transcriptome with HSC-iM program enrichment strength in upstream HSCs using DESeq2. Associations were made across pseudo-bulk profiles of HSCs and Monocytes from 32 donors from BoneMarrowMap as well as 235 mitochondrial DNA-defined clones spanning 13 donors from Weng *et al* 2024.

Supplementary Table 22 | List of antibodies used in this study

Supplementary References

1. García-Prat, L. *et al.* TFEB-mediated endolysosomal activity controls human hematopoietic stem cell fate. *Cell Stem Cell* (2021) doi:10.1016/j.stem.2021.07.003.
2. Rodgers, J. T. *et al.* mTORC1 controls the adaptive transition of quiescent stem cells from G0 to G(Alert). *Nature* **510**, 393–396 (2014).
3. Rodriguez-Fraticelli, A. E. *et al.* Single-cell lineage tracing unveils a role for TCF15 in haematopoiesis. *Nature* **583**, 585–589 (2020).
4. Cui, A. *et al.* Dictionary of immune responses to cytokines at single-cell resolution. *Nature* **625**, 377–384 (2024).
5. Yamashita, M. & Passegué, E. TNF-α coordinates hematopoietic stem cell survival and myeloid regeneration. *Cell Stem Cell* **25**, 357–372.e7 (2019).
6. Lehnertz, B. *et al.* HLF expression defines the human hematopoietic stem cell state. *Blood* **138**, 2642–2654 (2021).
7. Zheng, S., Papalexi, E., Butler, A., Stephenson, W. & Satija, R. Molecular transitions in early progenitors during human cord blood hematopoiesis. *Mol. Syst. Biol.* **14**, e8041 (2018).
8. Zeng, A. G. X. *et al.* Precise single-cell transcriptomic mapping of normal and leukemic cell states reveals unconventional lineage priming in acute myeloid leukemia. *bioRxiv* (2023) doi:10.1101/2023.12.26.573390.
9. Aibar, S. *et al.* SCENIC: single-cell regulatory network inference and clustering. *Nat. Methods* **14**, 1083–1086 (2017).
10. Wang, C. X., Zhang, L. & Wang, B. One Cell At a Time (OCAT): a unified framework to integrate and analyze single-cell RNA-seq data. *Genome Biol.* **23**, 102 (2022).
11. Schwartz, G. W. *et al.* TooManyCells identifies and visualizes relationships of single-cell clades. *Nat. Methods* **17**, 405–413 (2020).
12. Schwartz, G. W., Zhou, Y., Petrovic, J., Pear, W. S. & Faryabi, R. B. TooManyPeaks identifies drug-resistant-specific regulatory elements from single-cell leukemic epigenomes. *Cell Rep.* **36**, 109575 (2021).
13. Heaton, H. *et al.* Souporcell: robust clustering of single-cell RNA-seq data by genotype without reference genotypes. *Nat. Methods* **17**, 615–620 (2020).
14. Jakobsen, N. A. *et al.* Selective advantage of mutant stem cells in human clonal hematopoiesis is associated with attenuated response to inflammation and aging. *Cell Stem Cell* **31**, 1127–1144.e17 (2024).
15. Zeng, A. G. X. *et al.* Single-cell transcriptional atlas of human hematopoiesis reveals genetic and hierarchy-based determinants of aberrant AML differentiation. *Blood Cancer Discov.* **6**, 307–324 (2025).
16. Jaiswal, S. *et al.* Clonal hematopoiesis and risk of atherosclerotic cardiovascular disease. *N. Engl. J. Med.* **377**, 111–121 (2017).
17. Jaiswal, S. & Ebert, B. L. Clonal hematopoiesis in human aging and disease. *Science* **366**, (2019).
18. Weng, C. *et al.* Deciphering cell states and genealogies of human haematopoiesis. *Nature* **627**, 389–398 (2024).
19. Keenan, A. B. *et al.* ChEA3: transcription factor enrichment analysis by orthogonal omics integration. *Nucleic Acids Res.* **47**, W212–W224 (2019).
20. Dower, K., Ellis, D. K., Saraf, K., Jelinsky, S. A. & Lin, L.-L. Innate immune responses to TREM-1 activation: overlap, divergence, and positive and negative cross-talk with bacterial lipopolysaccharide. *J. Immunol.* **180**, 3520–3534 (2008).
21. Oetjen, K. A. *et al.* Human bone marrow assessment by single-cell RNA sequencing, mass cytometry, and flow cytometry. *JCI Insight* **3**, e124928 (2018).

22. Hay, S. B., Ferchen, K., Chetal, K., Grimes, H. L. & Salomonis, N. The Human Cell Atlas bone marrow single-cell interactive web portal. *Exp. Hematol.* **68**, 51–61 (2018).
23. Granja, J. M. *et al.* Single-cell multiomic analysis identifies regulatory programs in mixed-phenotype acute leukemia. *Nat. Biotechnol.* **37**, 1458–1465 (2019).
