# Supplementary material for: Human haematopoietic stem cells remember inflammatory stress
Source: Nature. 2026 May 27;655(8122):458–67. doi: 10.1038/s41586-026-10522-7 (PMC13345971; doi:10.1038/s41586-026-10522-7)
Supplement: Supplementary file 2 — Reporting Summary [file 41586_2026_10522_MOESM2_ESM.pdf]

nature portfolio

Corresponding author(s): Stephanie Z Xie

Last updated by author(s): Mar 2, 2026

Reporting Summary

Nature Portfolio wishes to improve the reproducibility of the work that we publish. This form provides structure for consistency and transparency in reporting. For further information on Nature Portfolio policies, see our Editorial Policies and the Editorial Policy Checklist.

Statistics

For all statistical analyses, confirm that the following items are present in the figure legend, table legend, main text, or Methods section.

n/a	Confirmed	
☐	☒	The exact sample size (*n*) for each experimental group/condition, given as a discrete number and unit of measurement
☐	☒	A statement on whether measurements were taken from distinct samples or whether the same sample was measured repeatedly
☐	☒	The statistical test(s) used AND whether they are one- or two-sided<br>*Only common tests should be described solely by name; describe more complex techniques in the Methods section.*
☐	☒	A description of all covariates tested
☐	☒	A description of any assumptions or corrections, such as tests of normality and adjustment for multiple comparisons
☐	☒	A full description of the statistical parameters including central tendency (e.g. means) or other basic estimates (e.g. regression coefficient) AND variation (e.g. standard deviation) or associated estimates of uncertainty (e.g. confidence intervals)
☐	☒	For null hypothesis testing, the test statistic (e.g. *F*, *t*, *r*) with confidence intervals, effect sizes, degrees of freedom and *P* value noted<br>*Give P values as exact values whenever suitable.*
☒	☐	For Bayesian analysis, information on the choice of priors and Markov chain Monte Carlo settings
☐	☒	For hierarchical and complex designs, identification of the appropriate level for tests and full reporting of outcomes
☐	☒	Estimates of effect sizes (e.g. Cohen's *d*, Pearson's *r*), indicating how they were calculated

Our web collection on statistics for biologists contains articles on many of the points above.

Software and code

Policy information about availability of computer code

Data collection	FACSDiva v9
Data analysis	FlowJo v10.8 to 10.10 (analysis of flow cytometry data)<br>Prism GraphPad v9.2 to 10.6 (statistical testing and plotting for biological data)<br>Microsoft powerpoint (v16) to assemble figure panels and create schematics<br><br>Data analysis:<br>ELDA (https://bioinf.wehi.edu.au/software/elda/)<br>scran v1.20.1<br>scanpy v1.8.2-v1.11.1<br>cnmf v1.3.4<br>SCTransform v2<br>fgsea v1.18.0<br>scDblFinder v1.6.0<br>BoneMarrowMap<br>AUCell v1.14.0<br>CellRanger-ARC v2.0.0<br>SoupX v1.6.2<br>SoupOrCell v2.5<br>Seurat v4.3.0-5.3.0<br>Signac v1.6.0

Python3 v3.11
harmony v0.1.1
MACS2 v2.2.7.1
OCAT (dev)
TooManyCells v2.2.0
presto v1.0.0
DESeq2 1.32.0 (A.V. 1.44.0)
chromVAR v1.14.0
Augur v1.0.3
rtracklayer v1.52.1
SCENIC+ v1.0
Metacell2
pycisTopic v1.0.1
pycisTarget v1.0.1
pySCENIC v0.12.0
variancePartition v1.22.0
GSVA v1.40.1
babelgene v22.3
SAM v1.0.1
pysam v0.15.1
samtools v1.17
cutadapt v3.4
STAR v2.7.10a
CellRanger v3.0.0
decoupler v2.0.2
NumPy v2.2.5
ggplot2 v3.5.1-4.0.0
dplyr v1.1.4
tidyverse v2.0.0
R v4.1.0-4.5.2
variancePartition v1.33.0

No new software or code was created. The code underlying key analyses in the main figures are available through https://figshare.com/s/a5555b5ed37e8208b88a - key methodology is described in detail in the Methods, and all of the analyses utilizes published/common packages

For manuscripts utilizing custom algorithms or software that are central to the research but not yet described in published literature, software must be made available to editors and reviewers. We strongly encourage code deposition in a community repository (e.g. GitHub). See the Nature Portfolio guidelines for submitting code & software for further information.

Data

Policy information about availability of data

All manuscripts must include a data availability statement. This statement should provide the following information, where applicable:

- Accession codes, unique identifiers, or web links for publicly available datasets
- A description of any restrictions on data availability
- For clinical datasets or third party data, please ensure that the statement adheres to our policy

Processed scMultiome and scRNA-seq datasets generated in this study as well as a gmt file with new gene expression signatures generated in this study are available through Figshare: https://figshare.com/s/107af8fb585a9a244235. These datasets are also available on GEO: LT-HSC scRNA-seq, CB HSPC scMultiome, CB Xenograft HSPC scMultiome and Xenograft Progenitor and Myeloid scRNA-seq, and BM Xenograft TARGET-seq+ datasets at GSE249479. BAM files for CB HSPC scMultiome, CB Xenograft HSPC scMultiome, and Xenograft Progenitor and Myeloid scRNA-seq are available on EGA at EGAS50000001624 with controlled access as required by institutional guidelines for sequencing data from human subjects. Other sequencing datasets have been previously published and have been referenced appropriately in the text, with the following accession codes: EGAS50000001623, GSE289435, EGAC00001000135, GSE120221, GSE139369, GSE190067, GSE98600, GSE124220, GSE196990, GSE235646, EGAS00001007358, GSE180298, GSE137864, S-BSST1524, and GSE219015. All data relating to the Ontario Health Study (OHS) are available upon request following Access approvals from the OHS (https://www.ontariohealthstudy.ca/for-researchers/whats-available/), which are reviewed by an independent Data Access Committee. OHS data can be accessed by Approved Users, and cannot be disclosed, transmitted, or transferred to unauthorized individuals as per the OHS Data and Biosample Access Policy (2017). A filtered compilation of GOBP signatures (https://download.baderlab.org/EM_Genesets/current_release/Human/symbol/) and signatures from MSigDB (https://www.gsea-msigdb.org/gsea/msigdb/collections.jsp) were used as described in the Methods. Source data for charts is available as supplementary information or tables where possible.

Research involving human participants, their data, or biological material

Policy information about studies with human participants or human data. See also policy information about sex, gender (identity/presentation), and sexual orientation and race, ethnicity and racism.

Reporting on sex and gender	Biological sex is referred using the correct terminology. Representation within our experiments from both common sexes is planned, unless not possible. Sex as a variant that affects transcriptional and epigenetic properties of cells is adjusted using methodology described in the Methods section. Sex is not considered an experimental variant in this study. Gender is not disclosed on the anonymized CB samples that we used. To the best of our knowledge, any of the data we have generated or used in this study is obtained from cells isolated from cis-gendered individuals.
Reporting on race, ethnicity, or other socially relevant	No information besides date/time of birth and biological sex is provided to us for umbilical cord blood samples used in this study. Details regarding donors within the published studies we have used can be found in the respective manuscripts, which have been referenced in full. Only age and sex of adult samples used in this study have been reported.

groupings	Age groupings were determined arbitrarily to match anecdotal reports of incidence of age-associated syndromes, as well as reported incidence of clonal hematopoiesis. Care is taken when naming of these groups to refer to the age bin and not assume ability of the donating individuals/patients.
Population characteristics	Age groupings were determined arbitrarily to match anecdotal reports of incidence of age-associated syndromes, as well as reported incidence of clonal hematopoiesis. Age of cord blood is implied. Age of adult samples ranges from 18 to 90 years of age and age ranges are clearly indicated on figures where relevant. Because age is a variant in some analyses, it was not adjusted for. Where adjusted, an appropriate indication in the text is provided.
Recruitment	There are no recruitment criteria for cord blood donors other than live healthy birth with no known inborn/genetic disorders at the time of birth. Bone marrow samples were collected in a unbiased manner from material discarded during hip replacement surgeries after obtaining consent from the donors.
Ethics oversight	Study approvals for published data can be found in their respective manuscripts.<br>Human CB samples were obtained with informed consent from Trillium Health, Credit Valley and William Osler Hospitals (Toronto, ON) according to procedures approved by the University Health Network (UHN) Research Ethics Board (REB# 02-0763). The investigation of data from the Ontario Health Study was approved by the University of Toronto Research Ethics Board (Protocol #00033112). Consent for use of data and blood samples was previously collected through the Ontario Health Study63 and CARTaGENE64 regional cohorts within the Canadian Partnership for Tomorrow's Health65. All research using human material was performed in accordance with relevant guidelines and regulations.

Note that full information on the approval of the study protocol must also be provided in the manuscript.

Field-specific reporting

Please select the one below that is the best fit for your research. If you are not sure, read the appropriate sections before making your selection.

☒ Life sciences ☐ Behavioural & social sciences ☐ Ecological, evolutionary & environmental sciences

For a reference copy of the document with all sections, see nature.com/documents/nr-reporting-summary-flat.pdf

Life sciences study design

All studies must disclose on these points even when the disclosure is negative.

Sample size	Statistical test to determine sample size was not conducted. Typical xenograft experiments can yield sufficient data with 3 to 4 mice, provided environment and other factors including recipient age and sex are controlled; and since mice can often fall sick and have early mortality - a feature of their immunocompromised nature - we increased the group size to 5 recipients. Variation in effect size was observed between animals engrafted with a single cord blood pool, so experiments were repeated with 2-3 cord blood pools as indicated in the text/figure legends.<br>For bone marrow transplantation, numbers of mice used per treatment group were limited by how much sample from each donor we had access to; a minimum number of 3 mice per group were transplanted to ensure potential for statistical comparisons, and if cells were available 4 mice were used.<br>For single cell sequencing experiments, cell numbers were input based on whatever was available, or with maximum input. No downsizing was performed.
Data exclusions	Engraftment data from some mice that were extremely sick/moribund prior to pre-determined experimental endpoint were excluded, with the rationale that a true measurement of the effect of inflammatory stress cannot be obtained with confounding sickness.<br>Data for sequencing experiments are all processed with quality control steps, described in the Methods, which results in appropriate exclusion of a minority of data points.
Replication	As indicated for selection of sample size, experiments were repeated with 2-3 independently processed and engrafted cord blood pools, each engrafted in 5 mice per experimental group. Replication showed consistent trends between treatment groups.<br>Engraftment with bone marrow samples was performed across multiple donors but not replicated in a separate cohort of mice due to low sample availability.<br>Computational conclusions were only made when the same/similar information could be extracted from multiple datasets. Comments to this effect are made throughout the text, and exceptions are indicated as limitations.
Randomization	Mice that received engrafted cells were randomized prior to irradiation, again during injection of cells, and cages with 4-5 mice were randomly selected to receive treatments. When the sex of the mice was male, mice were not randomized for irradiation to prevent confounding effects of fighting between non-littermates in cages.
Blinding	Investigators euthanizing and collecting tissue from mice were blinded to experimental groups - mice were assigned numbers. Blinding was not entirely possible during analysis, although care was taken to create homogeneous gating/sorting schemes where appropriate. Because blinding was not possible, data were analyzed or validated independently by at least two investigators.

Reporting for specific materials, systems and methods

We require information from authors about some types of materials, experimental systems and methods used in many studies. Here, indicate whether each material, system or method listed is relevant to your study. If you are not sure if a list item applies to your research, read the appropriate section before selecting a response.

Materials & experimental systems

n/a	Involved in the study	
☐	☒	Antibodies
☒	☐	Eukaryotic cell lines
☒	☐	Palaeontology and archaeology
☐	☒	Animals and other organisms
☒	☐	Clinical data
☒	☐	Dual use research of concern
☒	☐	Plants

Methods

n/a	Involved in the study	
☒	☐	ChIP-seq
☐	☒	Flow cytometry
☒	☐	MRI-based neuroimaging

Antibodies

Antibodies used

Antigen,Fluorophore,Company,Cat. No.,Species,Isotype,Clone
CD10,BV421,BD,562902,Mouse,IgG1κ,HI10a
CD10,Alexa Fluor 700,BD,563509,Mouse,IgG1κ,HI10a
CD14,BV605,BD,564054,Mouse,IgG2aκ,M5E2
CD14,PE-Cy5,beckman coulter,IM2640U,Mouse,IgG2a,RMO52
CD19,BV711,BD,563036,Mouse,IgG1κ,SJ25C1
CD19,PE,BD,349209,Mouse,IgG1κ,4G7
CD19,PE-Cy5,beckman coulter,IM2643U,Mouse,IgG1,J3-119
CD235a,PE,beckman coulter,IM2211U,Mouse,IgG1,11E4B-7-6
CD235a,PE-Cy5,BD,559944,Mouse,IgG2bκ,GA-R2
CD3,FITC,BD,349201,Mouse,IgG1κ,SK7
CD33,BV786,Biolegend,303428,Mouse,IgG1κ,WM53
CD33,BV421,Biolegend,303416,Mouse,IgG1κ,WM53
CD34,APC-Cy7,BD,624072 (Custom),Mouse,IgG1κ,581
CD38,PE-Cy7,BD,335790,Mouse,IgG1κ,HB7
CD45,Alexa Fluor 700,BD,560566,Mouse,IgG1κ,HI30
CD45,V500,BD,560777,Mouse,IgG1κ,HI30
CD45,APC,BD,340943,Mouse,IgG1κ,2D1
CD45RA,FITC,BD,555488,Mouse,IgG2bκ,HI100
CD49f,PE-Cy5,BD,551129,Rat,IgG2aκ,GoH3
CD56,BV605,BD,562780,Mouse,IgG2bκ,NCAM16.2
CD66b,Alexa Fluor 647,BD,561645,Mouse,IgMκ,G10F5
CD7,Alexa Fluor 700,BD,561603,Mouse,IgG1κ,M-T701
CD71,FITC,BD,347513,Mouse,IgG2a,L01.1
CD90,APC,BD,559869,Mouse,IgG1κ,5E10
CD90,PE,BD,555596,Mouse,IgG1κ,5E10

These are reported in Supplementary Table 22 along with the dilutions used, available in the supplemental information

Validation

All primary antibodies used in this study were validated for specificity of the human surface protein and application suitability in accordance with Nature Research antibody validation guidelines. Antibodies and dilutions used for flow cytometry analysis and fluorescence activated cell sorting (FACS) of primary and xenografted human samples (listed in Sup Table 22) were validated using one or more of the following strategies:

-shown to have human specificity and did not bind mouse analogs, consistent with published xenograft studies (Notta 2011, Garcia-Prat 2021, Kaufmann 2022, Xie 2021 and others)
-orthogonal validation of FACS purified populations from cord blood sources and verification of cell identity with in vitro clonogenic assays, gene expression and chromatin accessibility studies,
-in most cases, manufacturer provided validation data combined with peer reviewed literature was used

All antibody mediated phenotypes and population frequencies were reproducible across independent donors, multiple xenografts, and biological replicates, supporting antibody specificity and application validity.

Animals and other research organisms

Policy information about studies involving animals; ARRIVE guidelines recommended for reporting animal research, and Sex and Gender in Research

Laboratory animals

All animals in this study were bred under license and guidance from Jackson laboratories (JAX). NOD.Cg-Prkdcscid Il2rgtm1Wjl/SzJ (NSG; strain 005557), NSG-Tg(CMV-IL3,CSF2,KITLG)1Eav/MloySzJ (NSG-SGM3; strain 013062), and NSG-Kitem1Mvw/SzJ (NSGW41). All in vivo experiments were done with 8- to 12-week-old female or male mice. All mice were housed at the animal facility (ARC) at Princess Margaret Cancer Centre in a room designated only for immunocompromised mice with individually ventilated racks equipped with complete sterile microisolator caging (IVC), on corn-cob bedding and supplied with environmental enrichment in the form of a red house/tube and a cotton nestlet. Rooms were maintained at 21-22°C and 30-40% humidity with a 12:12 light-dark cycle with sunset and sunrise. Cages were changed at least once a week under a biological safety cabinet. Health status was monitored

using a combination of environmental monitoring and evaluation of soiled bedding from sentinel mice.

Wild animals

This study did not involve wild animals

Reporting on sex

Most experiments were conducted with a single recipient sex. Typically, female recipients were used, especially for longer-term xenografts, as better engraftment is reported in females. At least one CB group from each described experiment was engrafted in male recipients to ensure the effect of inflammatory stress was not restricted to female recipients. Mixed sex of NSG-SGM3 mice were used for secondary transplantation, but mice with different sex were divided equally amongst dosage groups to minimize sex-related bias. This is reported in a supplementary table in the manuscript. Female NSG-W41 mice were used as the strain available to us only engrafts without irradiation in female recipients.

Field-collected samples

samples were not collected from the field.

Ethics oversight

Animal experiments were done in accordance with institutional guidelines approved by the UHN Animal Care Committee.

Note that full information on the approval of the study protocol must also be provided in the manuscript.

Plants

Seed stocks

Report on the source of all seed stocks or other plant material used. If applicable, state the seed stock centre and catalogue number. If plant specimens were collected from the field, describe the collection location, date and sampling procedures.

Novel plant genotypes

Describe the methods by which all novel plant genotypes were produced. This includes those generated by transgenic approaches, gene editing, chemical/radiation-based mutagenesis and hybridization. For transgenic lines, describe the transformation method, the number of independent lines analyzed and the generation upon which experiments were performed. For gene-edited lines, describe the editor used, the endogenous sequence targeted for editing, the targeting guide RNA sequence (if applicable) and how the editor was applied.

Authentication

Describe any authentication procedures for each seed stock used or novel genotype generated. Describe any experiments used to assess the effect of a mutation and, where applicable, how potential secondary effects (e.g. second site T-DNA insertions, mosiacism, off-target gene editing) were examined.

Flow Cytometry

Plots

Confirm that:

☒ The axis labels state the marker and fluorochrome used (e.g. CD4-FITC).

☒ The axis scales are clearly visible. Include numbers along axes only for bottom left plot of group (a 'group' is an analysis of identical markers).

☒ All plots are contour plots with outliers or pseudocolor plots.

☒ A numerical value for number of cells or percentage (with statistics) is provided.

Methodology

Sample preparation

Samples were prepared for staining as described previously in Garcia-Prat et al 2021 and briefy described in the Methods section: Cells were centrifuged at 400g for 10min, then resuspended in PBS+5% FBS. Cells were resuspended at <10E6 cells/mL and stained in one or two subsequent rounds for 15min at room temperature each. Cells were washed following staining and resuspended in PBS+5%FBS and filtered through 40µm nylon mesh for sorting or analyses.

Instrument

BD FACSCelesta or BD Symphony A1 were used for analysis or BD FACSAria Fusion, BD FACSAria III or BD Symphony S6 for cell sorting

Software

FACSDiva v9 was used to collect data and or cell sorters, and FlowJo v10+ was used to analyze data

Cell population abundance

Typically, CD34+CD38- cells were isolated for engraftment, which were sorted from CD34+ cells enriched by physical (magnetic enrichment or depletion) methods - these are typically 10-15% of CD34+CD19- cells. Purity was typically not determined unless an excess of cells was isolated.
CD45+ cells were sorted from xenografts after physical mouse cell depletion, so these were also typically >85% positive for human CD45.
HSC/MPP fractions were also sorted from mouse depleted fractions. Their abundance was in the range of 0.15-0.3% of total human CD45 cells. Progenitor abundance (CD34+CD38+) was typically 40-50% of CD34+ cells. Myeloid cells (CD33+) varied in size ranging from 20-50% of human cells.
LT-HSC from cord blood are <0.01% of total CD34+ cells per the gating scheme described in Notta et al 2011

Gating strategy	Gating strategies are replicated from previously published studies, including Notta 2011, Garcia-Prat 2021, Kaufmann 2022, Xie 2019/2021. Generalized gating strategies are shown in supplementary note/figure 6. No flow plots are presented in the main or extended data figures, only summarized data.

☒ Tick this box to confirm that a figure exemplifying the gating strategy is provided in the Supplementary Information.
