# Supplementary material for: Human haematopoietic stem cells remember inflammatory stress
Source: Nature. 2026 May 27;655(8122):458–67. doi: 10.1038/s41586-026-10522-7 (PMC13345971; doi:10.1038/s41586-026-10522-7)
Supplement: Supplementary file 4 — Peer Review file [file 41586_2026_10522_MOESM4_ESM.pdf]

nature portfolio

Peer Review File

Human hematopoietic stem cells remember inflammatory stress

Corresponding Author: Dr Stephanie Xie

Any redactions in this file are there to maintain patient confidentiality, the confidentiality of unpublished data, or to remove third-party material.

This file contains all reviewer reports in order by version, followed by all author rebuttals in order by version.

Version 0:

Reviewer comments:

Referee #1

(Remarks to the Author)
In their manuscript, Zeng et al. use single-cell multi-ome analysis, as well as functional assays and re-analysis of published data to characterize a hematopoietic stem cell subset (termed "HSC-iM") that retains memory of inflammatory stress.

This study is relevant, as inflammation is a key driver of hematopoietic ageing and clonal selection. The identification of a, molecularly quite distinct, subset of HSCs that reacts to inflammation and achieves a lower cellular output is therefore important, and so is the finding that clonal hematopoiesis (CH) mutations overcome this inflammation-induced quiescence. The illustration of HSC-iMs in CoVID and ageing, as well as in xenotransplant models of inflammation, make this a very round and complete report of the prevalence of this population, although further insights on its functional consequences (e.g. role in leukemic transformation or immune dysfunction) are lacking.

I do not consider the findings described in this study fully novel. The authors identified the HSC-iM population as "HSC-2" in a prior study on clonal hematopoiesis (DOI: 10.1016/j.stem.2024.05.010). They also already characterized the effect of CH mutations on HSC-2 and their enrichment in individuals affected by CH (Figure 6 from Jakobsen et al). The authors handle this prior report transparently but it does compromise the novelty of this study. Specifically there is overlap between figure 5a-d here and Figure 6a-e from Jakobsen, as well as Figure 5j here and figure 6i from Jakobsen, and several supplemental items here and Jakobsen. Beyond this report, other studies have also observed sub-populations of HSCs with a similar molecular signature (e.g. population HSC-6 from 10.1038/s41586-024-07066-z). The merit of this (Zeng et al) manuscript is rather to demonstrate the widespread prevalence of this population in many relevant conditions.

So overall, I think this is a relevant, comprehensive and well-done study (specific points below). I would not hesitate to recommend its publication in any leading journal of the hematology/stem cell field, but it lacks the exceptional novelty and strong implications I would expect from a publication in Nature.

Conceptual points

1. Functional consequences of HSC-iM accumulations: This study shows that HSC-iMs exist, accumulate in many conditions, and are affected by CH mutations. The authors discuss potential relevance, but further *data* on the implications of this population for human health could really boost novelty and relevance of this manuscript.

2. Stability of HSC-iMs. The name "inflammatory memory" is suggestive, but in many scenarios that the authors report (old age, CHIP, potentially even the post-CoVID scenario) inflammation is still present. So these might also just be HSCs that happen to currently respond to inflammation, without keeping a long term stable memory.
The lineage tracing experiment suggests that many "clades" have both HSC-I and HSC-iM. This is consistent with the mitochondrial lineage tracing data from 10.1038/s41586-024-07066-z , where HSC-6 (likely corresponding to HSC-iM) and other HSC subpopulations co-exist in most clonal groups. Doesn't this suggest that HSC-I and HSC-iM frequently inter-convert?
But then again, these lineage tracing approaches have weaknesses. In the xenotransplant setting, would it be possible to

prospectively (e.g. with an AP1 reporter lentivirus) isolate HSC-iM and re-transplant to confirm that this is a stable population?

3. The findings on non-injected BM are interesting. To summarise, there is a treatment effect after an acute challenge 6 weeks after transplant (Figure S3b,c), but not after 20 weeks (S3d,e). How do the authors interpret this result? Does it mean that long-term HSCs that go into circulation cannot become HSC-iM? Might pre-existing clonal differences between HSCs determine the ability of an HSC to become an HSC-iM or go into circulation?

4. The xenotransplants were done with cord blood HSCs, is the ability to become HSC-iM different in adult BM HSCs?

Technical points

5. Documentation of computational methods: I was trying to understand how the y-axis from Figure 4b/c (HSC inflammatory memory program score) was calculated but couldn't find this information. The methods section "ICU-COVID analyses and signature derivation" appears to describe something else. I appreciate the effort of the authors to make code underlying key analyses public, but when I looked at the 8_postCOVID_analyses.nb.html the HSCII_inflammatory_memory_top200_AUC value just got read in from somewhere and I couldn't find how it was generated. Generally these Rmd files should minimally rely on reading in pre-computed information and rather make relevant computations transparent.

6. Lineage tracing analysis: The analysis in lines 500-523 has the goal to check if the inflammatory memory program is inherited from stem cells in a cell autonomous manner. For this, the best experimental design would probably be to uniquely barcode each cell prior to transplant (e.g. with a lentivirus), and check if, for HSCs that acquire the iM phenotype, their progeny is also iM. The authors try to circumvent this by working with a pool of 62 donors, and use genotype as a surrogate for cell of origin.
Question: 8,000-10,000 CD34+CD38- cells were transplanted (Methods). How many stem cells engrafted? The authors probably have limiting dilution data from a similar setup that allows to estimate a rough order of magnitude.
Comment 1: If more than 15 human HSCs engrafted, the bioinformatic algorithm should identify more than 15 "clades". Otherwise they are really comparing all cells that come from donors with, say, African ancestry, to cells that come from donors with, say, Asian ancestry. This is quite detached from what this experiment is trying to address.
Comment 2: If more than ~30-40 human HSCs engrafted, it would be better to repeat this experiment with lentiviral lineage tracing, since otherwise this experiment is not well suited to check if the clonal progeny of HSC-iM is also iM.

Manuscript readability

7. The manuscript would benefit from shortening and removal of supplementary items, in particular at the level of omic data description. E.g. in lines 218-294, if RNA-seq, ATAC-seq and GRN analyses all point towards a common set of pathways differing between HSC-I and HSC-II, I don't see a need to spread this out over six paragraphs and tens of figure panels.

Minor points

In figure S22e,f why are only 14 out of 15 clades shown? Also, the information here is not consistent with what is written in the text (6 were HSC-I specific in the text, and 8 in the figure)

Single cell analysis: In the uMAP in Figure S1K, donors separate. It is worrisome that different cell types (Figure S1J) differ in abundance across donors (comparing S1K and S1J), this is unlikely to be real biology? Should a different data integration/batch correction method be used here?

(Remarks on code availability)
See my main report.

Referee #2

(Remarks to the Author)

This manuscript addresses an interesting and very timely topic of inflammation and its effects on human HSCs and the longevity and integrity of the hematopoietic system. The effects of inflammation on HSCs have been quite extensively studied in mice, as it has important implications for human disease. Experimental in vivo models to study inflammation in human HSCs are lacking for obvious reasons, and primary samples are not easy to get access to, and have many confounding factors. To address this gap, this paper creates an experimental model to study inflammation in human HSCs, and compares their cord blood HSCs exposed to inflammatory stimuli after transplantation to immunodeficient mice to various data sets where inflammatory HSCs are likely to be present.

The manuscript first utilizes scRNAseq and Multiome (scRNA/ATACseq) to identify two distinct subsets of human cord blood HSCs, and show that one of the HSC subsets displays an inflammatory signature. After establishing this inflammatory HSC population in cord blood, they document the presence of a similar population in an inflammation stimulus immunodeficient mouse model that they create by exposing mice transplanted with cord blood HSCs to inflammatory stimuli, and finally correlates the findings to previously published data sets from patients recovered from COVID as well as patients with clonal

hematopoiesis (CH) caused by distinct mutations. Distinct from the previous studies published by the same team of senior authors (CH) and others (COVID), the current manuscript creates an inflammation stimulus mouse model for human HSCs where these concepts can be experimentally addressed.

Apart from the generation of the inflammatory stimulus mouse model after cord blood HSC transplantation and comparing the scRNAseq data from the mice to various data sets where HSCs have been exposed to diverse inflammatory stimuli, the manuscript is largely relying on bioinformatics analysis of various data sets. While the manuscript contains a lot of sophisticated bioinformatics analysis, such analysis are not always easy to translate to practice to make the conceptual advance easily accessible for readers. The authors could take the analysis and validation of the identity of inflammatory HSCs further and improve data presentation to make it easy for others to pinpoint inflammatory HSCs in diverse human samples and data sets, and thereby make the findings more accessible to the broader readership of general interest journals.

1) Can the authors identify a surface marker or other cellular features that can be assayed that would enable prospective isolation of the inflammatory HSCs? It would be ideal for future follow up studies if these cells could be enriched and tested for their epigenetic memory in experimental models. Were there any surface markers enriched in the inflammatory HSC subset?

2) What is the significance of inflammatory HSCs present in newborn cord blood?
- The manuscript begins with identification of distinct population of inflammatory HSCs in newborn cord blood samples. Since these cells are obtained at birth from healthy pregnancies, it is not clear what these distinct subsets mean at this stage of ontogeny. In theory, all HSCs should have been exposed to the same in the same “protected” utero environment, unless they derived or resided in specific niches. At the same time, inflammatory signaling is required for HSC development and physiologically important, so the exposure to inflammatory signals is not unexpected.

3) Can the authors identify a distinct single cell signature for inflammatory HSCs that is broadly applicable across different data sets, and could be used for easy identification of the presence of such cells?

4) How similar are the inflammatory HSCs obtained from different sources or interventions, or do they each have unique features? The manuscript is focusing on identifying similarities in HSCs that have been exposed to various inflammatory signals, as well as aged HSCs. Given that inflammatory signaling is also part of physiological HSC development, is there a way to distinguish inflammatory HSCs exposed to “physiological inflammatory signals”, vs those that have had dangerously high exposure to inflammatory signaling, or have already been compromised during ageing process?

5) How long is inflammatory memory retained, and does it depend on the type or duration of stimulus that cause it?

6) Does the age /developmental stage of HSCs influence how they respond to inflammatory stress in the experimental models?

7) How well does the immunodeficient mouse BM mimic the inflammatory environment in human? Some of the inflammatory cytokines are secreted from the stroma, are they species specific? Does the fact that the mouse is immunodeficient and lacks many critical immune cells compromise the inflammatory signals that the transplanted cells are exposed to after stimuli?

8) The manuscript is somewhat tedious to follow, as it is almost entirely graphs and figures illustrating bioinformatics analysis, and it does not render the data and findings easily accessible for the broader readership. Below are some suggestions summarized that could be added to make the findings more accessible and easy to apply also in other settings.

a. Can the authors identify surface marker candidates to isolate inflammatory HSCs?

b. Can the authors create molecular signatures from the single cell data that help distinguish the two different HSC subtypes, and help identify iM-HSCs from various data sets using dot plots or other features that are easy to visualize and recapitulate without having to do complex bioinformatics analysis?

c. The Multiome/ATAC seq data is presented as UMAPs, and there is little “visual” data to help the reader grasp what the epigenetic changes are. It would be helpful to see examples of altered accessibility of TSS or enhancers in genes that change upon inflammatory stimuli, or in CH or COVID patients, and are representative of the epigenetic memory.

d. The authors show that the ATAC seq data correlates well with gene expression. In addition, it would be interesting to identify accessible or closed regulatory sites that serve as evidence past exposure to inflammatory signals, or predict predisposition for expression in the future, without necessarily being actively expressed?

Specific questions:

Fig. 2 The authors should assess and document if the epigenetic remodeling triggered by inflammatory stimuli and the resulting epigenetic memory occurs primarily in enhancers or promoters or both?

Fig 3. The sizes of the two HSPC populations don’t look very different between PBS or TNF, but LPS treatment makes the inflammatory HSC population smaller rather than larger. What does this mean? They are all analyzed independently. If

analyzed in the same UMAP, with or without Harmony reduction, do they cluster together , or separately? I.e. how similar are inflammatory HSCs caused by different stimuli?

Comparison of various iM HSC data sets. Can the Multiome data sets be analyzed together, rather than simply projecting signatures from one data sets to anotherr? Can the inflammatory HSCs from COVID or CH be compared directly to Multiome of the experimental model to identify similarities and unique differences based on exposure type or time of exposure?

Fig. 4 i. What is the HSC aging meta signature of 37 genes?

Referee #3

(Remarks to the Author)
Zeng and colleagues present a very interesting and provocative study that identifies a subset of human hematopoietic stem cells (HSCs) that retain a transcriptional memory of prior inflammatory challenge. It has been well described in mouse models that some HSCs undergo epigenetic and transcriptional changes in response to inflammatory stimuli that permanently alter their function and allow them to respond differently upon repeated inflammatory challenge. To this reviewers knowledge, the current manuscript presents the first evidence of such populations existing in humans, identified through rigorous single cell transcriptional and multi-omic profiling across a variety of experimental and physiological human systems.
The authors start by exerting various inflammatory stressors (LPS, TNFa) to mice xenografted with cord blood-derived HSCs. The functional consequences of prior inflammatory exposure on human HSCs in these models was relatively modest. However, scMultiome profiling of the human cells after the experimental treatments revealed two distinct clusters of HSCs (both defined by expression of many traditional HSC marker gene) – a more “classical” HSC cluster and a very distinct cluster of HSCs with upregulation of NFkB, AP-1, and other inflammation-activated signaling pathways. These are termed the inflammatory memory HSCs (HSC-iM). The single cell profiling is quite rigorous and convincing. The authors then extend these experimental findings into physiological human settings and are able to identify analogous HSC-iM populations during human aging, recovery from severe COVID-19 infection, and clonal hematopoiesis (CH).
Overall the manuscript is quite compelling and uncovers regulation of human HSC dynamics at unprecedented single cell resolution. The statistical analyses and data representations are appropriate. However, the major conclusions are somewhat over-reliant on the sequencing data interpretation and in some instances are not supported by orthogonal functional analyses. In this reviewers mind, there are two major questions presented this data that need additional clarification. First, it is unclear how the authors disentangle their HSC-iM signature from that of normal human aging HSCs. From mouse studies, we know that HSCs accrue inflammatory damage with aging and that inflammation may in fact play a large role in the functional decline in HSC potential with age. All the physiological conditions where the authors identify HSC-iM in this study (aging, CH, COVID-19 recovery) are associated with the elderly (I could not find ages for COVID-19 patients?). So the existence of this cell population in these conditions might just reflect a consequence of human aging rather than a history of inflammatory challenge (although the two conditions may be hard to deconvolute in real world settings). Secondly, what are the consequences of this HSC-iM signature on functional output of the HSCs? The consequences of the HSC-iM signature are interpreted from transmission of this transcriptional program to downstream progenitor cells and monocytes. There is a lack of direct functional examination of the impact of this gene expression program on the HSC-iM HSCs in vivo. If these points could be clarified this would represent a very impactful study.

MAJOR POINTS:

• All the conditions where the HSC-iM signature is identified in human cell populations are associated with aging (elderly people, CH, COVID-19 recovery). To truly prove that this HSC-iM signature and these cells exist as a consequence of prior inflammatory stress, the authors should examine some pediatric populations to see if such HSC-iM exists in direct response to inflammatory challenge. There are several potential pediatric populations that could be studied – recovery from severe infectious disease (as done for the adult COVID-19 patients), autoimmune disease, or after vaccinations. If blood/BM could be sampled from these children and compared to “healthy” age-matched control samples to show enrichment of the HSC-IM signature and existence of this distinct HSC population in younger patients it would almost completely alleviate the concern that this signature overlaps strongly with the HSC aging signature.

• The authors have very convincingly described the existence of this distinct HSC-iM population of HSCs by scMultiome profiling. But what is the functional and physiological significance of this molecular signature in terms of the output of these HSCs and their response to repeated inflammatory challenge? The authors need to complement their molecular profiling with some in vivo functional assays. This would help clarify why this population exists in real-world human settings. This would probably be best examined using xenograft models for a more controlled experiment. Can the authors show there is a different lineage output from HSC-iM HSCs versus traditional HSCs in response to repeated inflammatory challenge? Is there a difference in self-renewal of the HSC-iM cells during infection? Do they respond better/faster than normal HSCs during repeated inflammatory stimulation? Can the authors use their scMultiome data to identify cell surface markers to separate HSC-iM HSCs versus other HSCs after inflammatory challenge via LPS, TNFa etc.. and then perform serial transplant to observe engraftment and self-renewal kinetics? If not a cell surface marker can a barcoding approach be used? This would be preferred but even in vitro CFU assays would be a helpful start. Is there a difference in cytokine output of the HSC-iM HSCs?

• Figure 1g-j – this seems like a very significant drop in human cell engraftment in response to an acute (16-hour) inflammatory challenge. What is the fate of the missing cells? Are they undergoing apoptosis? Mobilization from the BM?

The sharp drop in human cell engraftment over a short period is inconsistent with the statement on line 160-161 "these findings demonstrate a negative impact of acute inflammatory stimuli on human hematopoietic output in a xenograft model". It is likely not a defect of the stem cell differentiation output during this 16-hour period, but rather a negative impact on survival of the mature BM cells. Please clarify. Also, it would be better to represent these data as percentage change in engraftment for each mouse pre- and post-exposure. Due to variability in engraftment between mice, perhaps the drop comes from selection of mice that were unknowingly at a lower starting BM engraftment. Without knowing the pre-exposure engraftment level for each mouse it is hard to justify this claim.

MINOR POINTS:

• Figure 1: Some of the xenograft data is hard to interpret (e.g %CD34 of CD45). Please show overall frequency and absolute numbers of these cell populations per mouse for more transparency. And it would be better to have quantification of more pure HSC populations (as indicated on line 206) rather than just overall CD34+.

• Line 398-399: "Taken together, our results demonstrate the molecular programs underlying the HSC-iM state accumulate in human HSCs with age". The alternative explanation is that aging HSCs are just reflective of a lifetime accumulation of inflammatory insults.

• The section "CH mutations preferentially impact HSC-iM to promote differentiation" is not really supportive of the overall message of the manuscript. Because it seems like different platforms were used for the analysis there is no direct readout of HSC-iM in this data. Line 483 – "predominantly within the HSC2 state (which is equivalent to HSC-iM). And again, the impact on differentiation is inferred rather than a direct functional readout.

Referee #4

(Remarks to the Author)
In the manuscript entitled "Identification of Human Hematopoietic Stem Cell Subset That Retains Memory of Inflammatory Stress," Zeng and colleagues investigate the impact of inflammatory stimuli, specifically TNF-α and LPS, on hematopoietic stem cells (HSCs) and their functional outcomes. Utilizing a preclinical xenograft inflammatory recovery model, as well as analyzing HSCs from individuals with severe COVID-19, aged individuals, and those with clonal hematopoiesis, the authors identify a subset of HSCs with inflammatory memory (HSC-iM). These cells retain inflammatory reprogramming, which can be transmitted to their progeny—particularly innate immune cells—in a cell-autonomous manner. Notably, the authors demonstrate that the programming of HSC-iM shares similarities with the mechanisms underlying memory T cell formation. While this study is significant and employs elegant experimental approaches to address key hypotheses, there are several conceptual gaps that require further scrutiny.

1. It is unclear how intraperitoneal administration of LPS or TNF leads to the reprogramming of HSCs and the generation of HSC-iM. Is this a direct effect of LPS or TNF on HSCs in the bone marrow (BM), or is it mediated indirectly through systemic factors? Can the authors measure the levels of TNF and LPS in the BM of the injected CD34+ cells to clarify this mechanism? Previous studies have shown that systemic type I IFN or IL-1 signaling is required for HSC reprogramming following intraperitoneal administration of β-glucan. Additionally, the memory programming of HSCs in response to BCG versus Mtb infection has been attributed to type II IFN and type I IFN, respectively. Does the generation of HSC-iM depend on TLR4 or TNFR signaling pathways? Addressing these points could provide critical insights into the different mechanisms underlying HSC reprogramming.

2. The data from the COVID-19 study are intriguing and further support the central hypothesis of the current work. However, it is important to clearly define the ICU-control group. Were there any septic patients included among the ICU controls? If so, it would be expected that the HSC-iM profile induced by LPS or TNF would be more similar in these septic patients compared to the COVID-19 patients. Since SARS-CoV-2 is a viral infection that primarily engages type I IFN responses, these data suggest that type I IFN in the bone marrow might play a crucial role in the LPS or TNF inflammatory xenograft model for generating HSC-iM. Additionally, in the original study by Cheong et al., the sample sizes were COVID-19 = 19, ICU-control = 11, and healthy = 19. However, in the current study, the sample sizes have been reduced to COVID-19 = 4, ICU-control = 6, and healthy = 7. Can the authors clarify the reasons for this reduction in sample size?

3. The authors demonstrated that the memory program of HSC-iM is more similar to memory T cells. Could this observation be influenced by the xenograft model system? Notably, the model was established using fetal CD34+ cells from cord blood, which are known to have distinct transcriptional and epigenetic profiles that promote rapid growth and differentiation, particularly through the upregulation of genes associated with proliferation and lymphoid differentiation. Can the authors compare these findings to an adult CD34+ xenograft model to determine whether the observed memory program is unique to fetal HSCs or shared across different HSC sources?

4. Interestingly, the authors indicate that they have identified a memory program in HSCs that differs from those reported in BCG studies, both in mice and humans. This is a critical observation, as humans are exposed to a variety of pathogens that cause acute or chronic infections, each with distinct characteristics, such as structural differences (e.g., BCG lacks LPS), replication rates, and pathogenicity. For instance, a recent study (PMID: 33125891) comparing the BCG vaccine to a virulent strain of Mycobacterium tuberculosis (Mtb, the causative agent of TB) showed that, unlike BCG, Mtb-induced HSC memory responses result in a distinct program characterized by reduced engraftment and the generation of monocytes/macrophages

that are more susceptible to subsequent Mtb infection. Can the authors compare their identified HSC memory program to the existing Mtb data to highlight potential similarities or differences? Furthermore, in real-world conditions, humans are rarely exposed to isolated factors like LPS or TNF-α. How would cumulative exposure to multiple pathogens or inflammatory stimuli influence the development and functional outcomes of HSC-iM? Addressing these questions could significantly enhance our understanding of how diverse environmental and pathogenic exposures shape HSC memory.

Version 1:

Reviewer comments:

Referee #1

(Remarks to the Author)
I appreciate the data on human health (SCD, AML, all-cause mortality) that the authors have added, and their study is now a very comprehensive survey of a molecularly distinct HSC subset that accumulates in inflammaging and as a long-term consequence of inflammatory insults. This study is important, because it highlights the transversal importance of inflammation (and not clonal hematopoiesis mutations) in shaping health outcomes, and because this hitherto not well described HSC subset might be of large relevance also in other contexts such as trained immunity.

I recommend its publication, with a few final recommendations:

- The manuscript requires editing and shortening. I struggled both during my initial review and my re-review to understand all messages and the experimental evidence that they are based on. Key messages should be distilled more clearly. Description of experimental setups and analyses should be simplified. and unnecessary detail removed.
- Good sections to shorten drastically would be “transmission of inflammatory memory programs from HSC-iM to downstream progeny” and the section on extended data figure 8. These sections are still not fully conclusive, as discussed also in the author's reply to my original points 2 and 6. This regards the “lineage tracing” setup employed, but also analysis from figure EV8: I drew the bar chart of figure EV8h as a scatter plot (% HSC-iM in primary vs. xenotransplant) and there was no correlation. I think these sections are not required to be included prominently, as the manuscript has been strengthened elsewhere, particularly regarding health relevance. I follow the argument of the authors that clonal tracing in human HSCs might be beyond the scope of this study. I believe that good tools for this task now exist (10.1038/s41586-025-09041-8) and could be cited in the discussion.
- I liked the wording employed in the rebuttal letter, "These data raise a novel hypothesis that HSC-iM enrichment reflects the systemic level of inflammation in unhealthy individuals, and that some of the health correlations attributed to CH status in current literature could be survivorship bias of an earlier HSC-iM-driven inflammatory state." This could be included in the main text.
- I would suggest to discuss the extent to which the HSC-iM signature might capture more general inflammation marks and not always be related to HSC-iM. In particular, in the context of all cause mortality / intermountain risk score prediction, any inflammation signature would probably work as well? See for example 10.1016/j.ajhg.2025.06.009 . Also they could cite 10.1038/s41467-025-58321-4 in the context of HSC-iMs and SCD.

Referee #2

(Remarks to the Author)
Xie and colleagues have considered reviewers comments thoroughly and performed extensive new experiments and analyses, and prepared a revised manuscript that clarifies and expands their findings on human HSCs with epigenetic inflammatory memory. They extend their findings further to disease settings by showing increased HSC-iM in children with sickle cell disease, and in patients with myeloid malignancies. They show that HSC-iM inflammatory state is reflected in HSC functional properties upon transplantation and transmits to both myeloid and lymphoid progeny. They document altered cytokine secretion in mice transplanted with HSC-iM. They also include analysis of a large transcriptome data set of 400 individuals and show that HSC-iM signature correlates with a high risk score for 5 year all cause mortality, strengthening the link between HSC-iM and clinical impact. The authors made extensive attempts to identify a surface marker for HSC-iM, including performing Cite-seq, and I applaud their efforts, even though they were not able to identify a suitable surface marker for prospectively isolating HSC-iM. The authors have responded to reviewer criticisms carefully and made revisions that make the manuscript and figures more accessible for broader audiences. I am convinced that publishing their novel findings will have an impact on the field, and allow future studies built on it. I would be happy to recommend publication of the revised manuscript in Nature.

Referee #3

(Remarks to the Author)
The authors have added a plethora of new data which further supports their observation of a human HSC subset that retains molecular and functional memory of prior inflammatory stress. This significantly strengthens the manuscript. There are many novel and interesting concepts generated from this work. For example, were any differences in the HSC-iM population observed between CH with DNMT3A versus TET2 mutations? The authors seem to have compiled them together for their figures. But I would not dare ask any further experimentation or analysis from the authors. They have gone above and beyond in the revised manuscript. I credit the group for their diligence, and this study will set the stage for multiple

opportunities for investigation in the coming years. The originality and significance is high as it identifies a new human HSC signature across multiple age ranges and disease stages. They have generated a huge amount of data to support this which will be a valuable resource for the community. The conclusions are robustly supported by the data. I have no further concerns.

- GAC

Referee #4

(Remarks to the Author)
The authors experimentally addressed all my concerns.

Open Access This Peer Review File is licensed under a Creative Commons Attribution 4.0 International License, which permits use, sharing, adaptation, distribution and reproduction in any medium or format, as long as you give appropriate credit to the original author(s) and the source, provide a link to the Creative Commons license, and indicate if changes were made.
In cases where reviewers are anonymous, credit should be given to 'Anonymous Referee' and the source.
The images or other third party material in this Peer Review File are included in the article's Creative Commons license, unless indicated otherwise in a credit line to the material. If material is not included in the article's Creative Commons license and your intended use is not permitted by statutory regulation or exceeds the permitted use, you will need to obtain permission directly from the copyright holder.
To view a copy of this license, visit https://creativecommons.org/licenses/by/4.0/

We thank the Referees for their thorough and constructive assessment of our manuscript. We are pleased to report that we have generated a significant amount of new experimental data that significantly strengthens the main message of our paper. To our knowledge, this is the first experimental study to demonstrate inflammatory memory at both the chromatin and transcriptional level in stem cells of any human tissue, and to demonstrate its relevance towards a breadth of health conditions across the human population. As highlighted by Dr. Gebala, the key concern identified by Referees #1-3 pertained to the functional relevance of the HSC-I and HSC-iM subsets to human health. Below, we summarize the new findings that support the functional importance of HSC-iM as a cellular state to predict heterogeneous human health outcomes:

1. We show that HSC-iM is elevated in pediatric samples from patients with sickle cell disease, an inherited blood disorder known to be associated with inflammation (see Referee Fig. 1). This suggests that HSC-iM not only arises during physiological aging, but also in pediatric patients exposed to an inflammatory environment.
2. We find that HSC-iM is elevated in specific subsets of acute myeloid leukemia (AML) (see Referee Fig. 2), providing the first link between HSC-iM, CH and AML and pointing to the possibility that the HSC-iM program is relevant in other hematological malignancies.
3. To validate the transmission of the HSC-iM inflammatory state to downstream progeny in human adult samples, we xenotransplanted adult bone marrow stem and progenitor cells from 5 individuals in our clonal hematopoiesis (CH) cohort - where the HSC-iM and HSC-I composition was known - into immunodeficient mice. We found that HSC-I and HSC-iM transmit their state to myeloid progeny after xenotransplantation. Importantly, fewer progeny were derived from HSC-iM than HSC-I, independent of CH status of the donor (see Referee Fig. 6, 8 & 23). Indeed, HSC-iM origin was a stronger determinant of inflammatory state than CH status. These data raise a novel hypothesis that HSC-iM enrichment reflects the systemic level of inflammation in unhealthy individuals, and that some of the health correlations attributed to CH status in current literature could be survivorship bias of an earlier HSC-iM-driven inflammatory state.
4. We extend the concept of transmission of the HSC-iM program to downstream progeny to include circulating B cells, T cells, and dendritic cells. We evaluated HSC-iM enrichment in a new single cell transcriptome dataset of peripheral blood samples from ~400 individuals within a large population cohort (Ontario Health Study) with our collaborator Dr. P. Awadalla (Oxford). Remarkably, we were able to associate this transmitted HSC-iM signature to a well-documented risk score for 5 year all-cause mortality in young individuals (see Referee Fig. 3). This represents the strongest finding we have made regarding the clinical impact of HSC-iM.

Another set of concerns from the Referees was regarding the relevance of our xenograft model. We summarize new findings that support the functional importance of our model:

1. We generated new single cell transcriptomic profiles of CD34+CD38+ progenitors from our xenograft-recovery model to show that HSC-iM transmits inflammatory properties to BOTH the B and myeloid lineages via their progenitors (see Referee Fig. 21 & 22), akin to what we see in the human CH cohort (point 3 above) and Ontario Health Study data (point 4 above).
2. We profiled 23 human and 18 mouse cytokines in peripheral blood plasma from our xenografts at 6w (acute response) and 20w (steady state post-recovery) and show that the human graft itself contributes to a complex cytokine milieu (see Referee Fig. 15, 25, 27 & 28). Such comprehensive cytokine profiling in the xenograft setting has never been reported and demonstrates another aspect of human hematopoiesis that can be recapitulated when humanizing the blood system of immunodeficient mice.

The relevant new pieces of data are presented in the fulsome responses to the specific concerns from each Referee below. Additionally, we provide a detailed Supplementary Information section to more fully described the detailed informatic analyses we have undertaken. The detailed point-by-point response to all comments from the Referees have helped improve the manuscript, and for that we are deeply grateful.

Referee comments:

Referee #1 (Remarks to the Author):

In their manuscript, Zeng et al. use single-cell multi-ome analysis, as well as functional assays and re-analysis of published data to characterize a hematopoietic stem cell subset (termed "HSC-iM") that retains memory of inflammatory stress.

This study is **relevant**, as inflammation is a key driver of hematopoietic ageing and clonal selection. The identification of a, molecularly quite distinct, subset of HSCs that reacts to inflammation and achieves a lower cellular output is therefore **important**, and so is the finding that clonal hematopoiesis (CH) mutations overcome this inflammation-induced quiescence. The illustration of HSC-iMs in CoVID and ageing, as well as in xenotransplant models of inflammation, make this **a very round and complete report of the prevalence of this population**, although further insights on its functional consequences (e.g. role in leukemic transformation or immune dysfunction) are lacking.

I do not consider the findings described in this study fully novel. The authors identified the HSC-iM population as "HSC-2" in a prior study on clonal hematopoiesis (DOI: 10.1016/j.stem.2024.05.010). They also already characterized the effect of CH mutations on HSC-2 and their enrichment in individuals affected by CH (Figure 6 from Jakobsen et al). The authors handle this prior report transparently but it does compromise the novelty of this study. Specifically there is overlap between figure 5a-d here and Figure 6a-e from Jakobsen, as well as Figure 5j here and figure 6i from Jakobsen, and several supplemental items here and Jakobsen. Beyond this report, other studies have also observed sub-populations of HSCs with a similar molecular signature (e.g. population HSC-6 from 10.1038/s41586-024-07066-z). The merit of this (Zeng et al) manuscript is rather to demonstrate the widespread prevalence of this population in many relevant conditions.
So overall, I think this is a relevant, comprehensive and well-done study (specific points below). I would not hesitate to recommend its publication in any leading journal of the hematology/stem cell field, but it lacks the exceptional novelty and strong implications I would expect from a publication in Nature.
We thank the Referee for finding our study to be relevant and important. First, we would like to highlight the distinction between our study, as compared to the Jakobsen et al study. HSC2 was identified in that study using a computational algorithm called "Self-Assembly Manifold" (SAM) to cluster HSCs. It was noted in that paper that HSC2 was enriched for an "inflammation" gene set by GSEA. However, we did NOT describe the HSC2 subset in the Jakobsen et al study as one with inflammatory memory; **there is no mention of "memory" in that study**. It is worthwhile clarifying that the concept of a specific human HSC subset with memory of prior inflammatory stress did not exist at the time of publication of the Jakobsen et al

manuscript. We used data from Jakobsen et al as one of several real-world, clinically relevant scenarios for validating the findings from the experimental xenograft models we first established in this manuscript.

The distinct messages of the current paper are: (i) the development of an experimental model that led to the identification of two human HSC subsets, where one possesses a memory of prior inflammatory stress; and (ii) demonstrating that the new HSC subset is relevant to human aging and all-cause mortality, infection, clonal hematopoiesis, AML and sickle cell disease.

Thus, these are not extensions of any findings in Jakobsen et al, but a fully distinct set of messages. We establish that the HSC-iM subset exhibits inflammatory memory at both the chromatin accessibility and transcriptional level. Our current study also establishes precedence for xenograft models to faithfully mimic the effect of aging/inflammation on human HSCs and their progeny, as well as laying the foundation for future mechanistic studies of how environmental drivers of inflammatory response can lead to pathology.

Conceptual points

1. Functional consequences of HSC-iM accumulations: This study shows that HSC-iMs exist, accumulate in many conditions, and are affected by CH mutations. The authors discuss potential relevance, but further *data* on the implications of this population for human health could really boost novelty and relevance of this manuscript.

We have heeded this excellent prompt for further exploration of the relevance of HSC-iM to human health. Beyond the role in COVID and CH shown in the original submission, we now report four additional settings of relevance:

<u>HSC-iM in sickle cell disease (SCD)</u>. We leveraged a new study describing single cell transcriptome profiling of CD34+ bone marrow samples from patients with SCD and age-matched healthy donors (HD) between 12-20 years of age[1]. We found HSC-iM enrichment in SCD HSCs compared to HD HSCs (Referee Fig. 1a). SCD pathophysiology manifests in a variable manner. We find that HSC-iM enrichment in SCD patients is variable, with some HSC

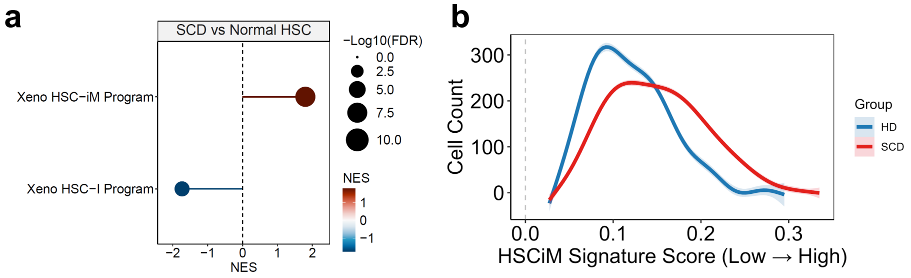

Referee Fig. 1. HSC-iM is enriched in sickle cell disease. **a**, GSEA results depicting HSC-iM and HSC-I gene expression program enrichment in HSCs comparing HSCs from SCD patients vs healthy controls from Sobrino, et al. **b**, HSC-iM signature score is variable in HSCs from individuals with SCD.

displaying similar HSC-iM enrichment to HD HSCs, while others are higher (Referee Fig. 1b). The functional relevance of this variability is unknown, but it would be interesting to explore how it is connected to the known waxing and waning of crises within SCD in future studies, as well as connecting gene or other therapy outcomes to the inflammatory memory status of SCD recipients. These data have been added to Fig. 4 and Extended Data Fig. 6 of the revised manuscript (MS).

HSC-iM and AML. We originally reported that the HSC-iM state accumulates with age and CH. We[2] and others[3] showed that CH is linked to increased risk of acute myeloid leukemia (AML). This raised the question of whether HSC-iM was directly associated with AML. To explore the transcriptional enrichment of HSC-iM in AML, we compared the transcriptional profiles of HSC/MPP-like cells in AML patients to those from healthy controls. Specifically, we focused on scRNAseq data from six studies with 1) clinical information on *DNMT3A* and *TET2* mutational status and 2) those with internal healthy control samples[4–9]. In total, we analyzed 91 AML samples and 16 healthy control samples. By GSEA, we found significant HSC-iM enrichment in AML compared to the healthy controls (FDR = 9.1e-08). This is most pronounced in AML samples with mutations in *TET2* (n=18, NES = 2.44, FDR = 2.7e-18) or *DNMT3A* (n=15, NES = 2.33, FDR = 5.2e-15) compared to those that did not (n=35, NES = 1.38, FDR = 3.2e-02). These data suggest a complex relationship between inflammatory memory in HSC and clonal expansion and evolution to malignancy that is mutation-dependent (see Referee Fig. 2). These data have been added to Extended Data Fig. 7 in the revised MS. Though these data are interesting, future studies with larger AML cohorts and specific experimental modeling with inflammatory challenges would be necessary to understand whether this enrichment of HSC-iM in malignancy is a consequence of evolution of HSC-iM to CH to AML.

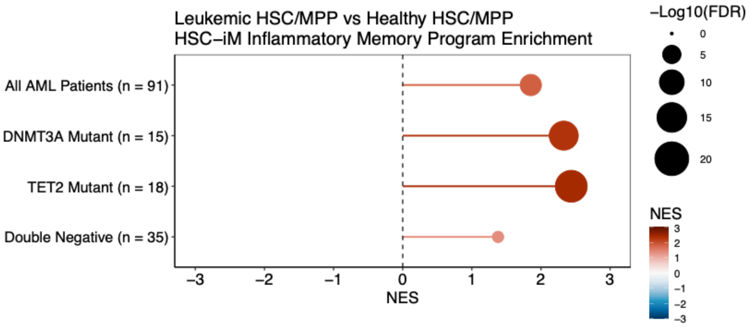

Referee Fig. 2. GSEA of the HSC-iM signature in primary acute myeloid leukemia (stratified by subtype) as compared to healthy controls.

Inflammatory programs in HSC-iM-derived Monocytes following xenotransplantation.
The basis of the epidemiologic associations of CH with all-cause mortality as well as multiple inflammation-associated diseases has been attributed primarily to potentiation of inflammatory programs within CH^{MUT} immune cells compared to CH^{WT} immune cells, particularly in the context of monocytes and macrophages. In our new xenotransplantation experiment of bone marrow HSPCs from adult CH donors, we confirm that HSC-iM derived monocytes are enriched for inflammatory programs independent of CH status (see Referee Fig. 6 and 8 in response to

points 2 and 4). Critically, CH^{WT} monocytes derived from HSC-iM have significantly higher levels of TNFα signaling compared to CH^{MUT} monocytes derived from HSC-I (see Referee Fig. 8c), linking HSC-iM origin with a more potent inflammatory phenotype compared to CH mutation status in monocytes. We recognize these data are from only 4 CH donors. Nonetheless, these provocative data suggests that inference of HSC-iM origin at larger scale has the potential to reveal even stronger epidemiological associations with diseases of inflammation and all-cause mortality than what has been demonstrated for CH.

<u>HSC-iM program and all-cause mortality.</u>
As a proof of principle, we evaluated the relevance of the transmission of pro-inflammatory transcriptional features of HSC-iM to their downstream progeny in relation to the well-known Intermountain Risk Score (IRS). The IRS is a clinical decision tool that uses common lab results, age, and sex to predict a patient's risk of mortality and was trained and validated using the health records of 119,379 patients enrolled in the Intermountain HMO (comprising 22 hospitals and many clinics and employed physician offices with the state of Utah in the United States)[10]. The IRS is associated with 5-year all-cause mortality and has been validated as a prognostic tool in settings of heart failure[11], cardiogenic shock[12], and COVID-19[13]. We harnessed new single-cell (sc)RNAseq data from peripheral blood of ~400 individuals in the Ontario Health Study (OHS), for whom age, sex, and a modified version of the IRS - that uses six parameters from a complete blood count - was known (Referee Fig. 3a,b). All participants in this scRNAseq cohort were free of any of 21 common chronic health conditions (eg. high blood pressure, diabetes) at the time of sampling. We first demonstrate broad transmission of HSC-iM associated programs across multiple immune effector cell types in an expanded single-cell dataset from our xenograft model (Referee Fig. 21). Within the OHS cohort, we thus scored mature immune cell populations (CD14 and CD16 monocytes, dendritic cells, NK cells, naive T cells, and B cells) for HSC-I and HSC-iM program enrichment and used regressions to predict IRS with these scores within the young samples separately from the aged samples. HSC-I program enrichment in natural killer (NK) cells, naive T cells, dendritic cells (DC), CD14 monocytes, and CD16 monocytes predicts low IRS, while HSC-iM program enrichment in B cells, naive T cells, and DC significantly predicts high IRS in young samples (Referee Fig. 3c). In contrast, the effect size in aged individuals is small suggesting that the impact of prior inflammatory insults on mortality risk is age-dependent. These data have been added to Fig. 6 in the revised MS. This remarkable finding substantially addresses the request from the Referee to provide more examples of relevance for HSC-iM in human health, and points to the HSC-iM program as reflective of prior inflammatory insults that is distinct from aging.

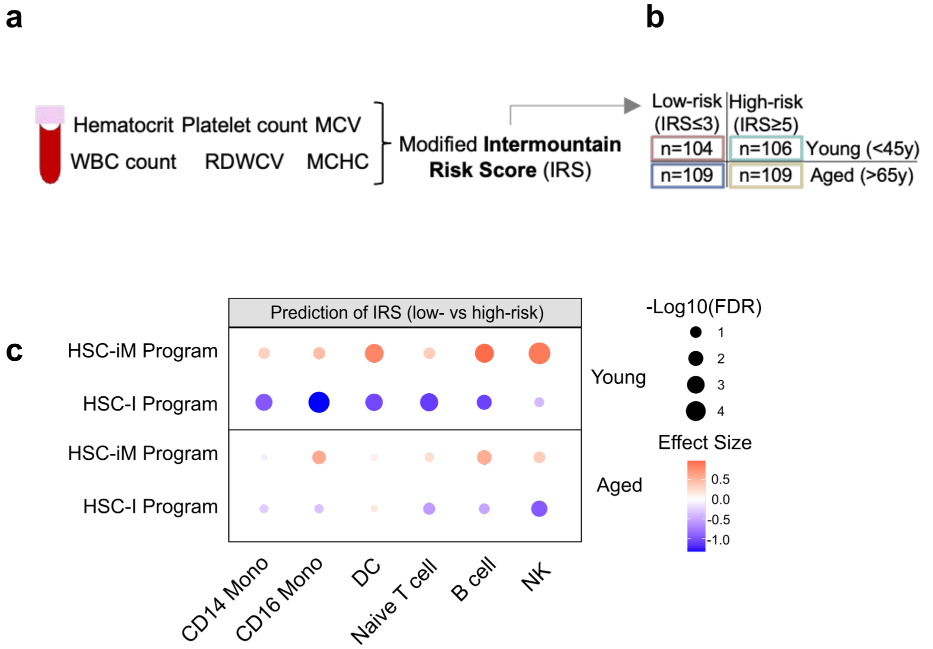

Referee Fig. 3. HSC-iM signature enrichment in mature cells predicts increased Intermountain Risk Score (IRS) within the Ontario Health Study cohort. a, Schematic depicting the calculation of the modified IRS using six widely measured complete blood count values. b, Schematic depicting the stratification scheme for the Ontario Health Study scRNAseq cohort by age and IRS. c, Prediction of low or high IRS using HSC-I and HSC-iM program scores in mature cell pseudobulks within young or aged samples using generalized linear models, corrected for batch and sex.

2. Stability of HSC-iMs. The name “inflammatory memory” is suggestive, but in many scenarios that the authors report (old age, CHIP, potentially even the post-CoVID scenario) inflammation is still present. So these might also just be HSCs that happen to currently respond to inflammation, without keeping a long term stable memory.

We concur that the stability of HSC-iMs is of great interest. However, at least in the severe COVID recovery scenario, we were accessing circulating HSCs 2-4 months post-infection and observed more HSC-iM program enrichment not just compared to healthy controls, but also to individuals that had previously been in the ICU for reasons unrelated to COVID infection (see Referee Table 1, Referee 4, point 2; clinical information is listed in Table S1 of Cheong, et al.[14]). This strongly suggests that inflammatory memory is stable at least for 2-4 months in peripheral blood HSCs after an infection.

In Referee Fig. 4 we also provide cytokine profiling data from the post-COVID and control donors included in our original analysis. As shown in Fig. 4, HSCs from these samples were enriched for transcriptional and epigenetic inflammatory memory programs compared to Healthy Controls and ICU Controls. There are no key inflammatory cytokines which are specifically enriched in post-COVID samples compared to both Healthy and ICU controls. In fact, the measured pro-inflammatory cytokines tend to be higher in the ICU controls, which decouples our inflammatory memory program from ongoing inflammation.

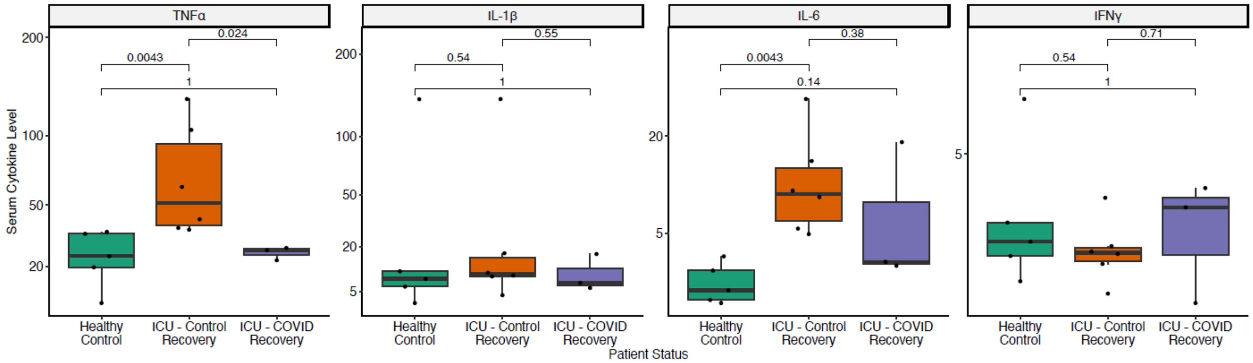

Referee Fig. 4. Plasma cytokine profiles from samples for which scMultiome was done from individuals following ICU-COVID recovery compared to ICU-Control and Healthy Controls. Luminex profiling for the indicated cytokines for each donor is from Cheong, et al.

Redacted

The lineage tracing experiment suggests that many "clades" have both HSC-I and HSC-iM. This is consistent with the mitochondrial lineage tracing data from 10.1038/s41586-024-07066-z , where HSC-6 (likely corresponding to HSC-iM) and other HSC subpopulations co-exist in most c lonal groups. Doesn't this suggest that HSC-I and HSC-iM frequently inter-convert?

We recognize this is a fundamental question. To definitively address this requires samples where the HSC-I/HSC-iM state of HSCs is known and there is an ability to clonally trace these states over time. Indeed, given the complexity of the question and the current absence of reliable tools for clonal tracing in primary human HSCs, a concerted effort from many groups will be needed for future investigations of such clonal relationships. Much progress has been made in the mouse system in this regard, where more tools are available; yet, many groups are required to continue this ongoing work. However, as a surrogate, we present the comparison of the frequency of HSC-I/HSC-iM states between primary samples and their paired xenografts. In the last 9 months we have made a strong effort to address this important question with this new set of challenging transplant experiments.

We created a modified xenograft challenge model using NSG-SGM3 immunodeficient mice, which allows for better myeloid differentiation than NSG (Referee Fig. 6a). We then asked the question: do HSCs that are labelled as HSC-iM and HSC-I before transplant, remain as such after transplantation and inflammation? A prerequisite for such an experiment would be to know, *a priori*, the HSC-I vs HSC-iM composition of a donor. We were able to do this using the original human bone marrow samples (mix of CH and non-CH) where we had previously identified the proportion of HSC-I/HSC1 and HSC-iM/HSC2 using TARGETseq+, as shown in Fig. 5k. We performed xenotransplantation with 1 CB sample and 5 BM samples and assessed engraftment at 12 weeks (Referee Fig. 6b-c).

CD34+ HSPC and CD33+ progeny were isolated from the xenografts for TARGET-seq+ analysis, and integrated with the original scRNAseq from primary BM HSPCs from the same donors (see Supplementary Note 4). To identify HSC-I and HSC-iM derived cells within the xenografts, we first defined a set of genes that distinguish the differentiation trajectories of HSC-I and HSC-iM dominant primary BM samples in the original TARGET-seq+ dataset. We performed differential expression analysis between HSC-I and HSC-iM dominant samples within each cell type, and selected genes that were differentially expressed at FDR < 0.10 within ≥ 5 cell types (Referee Fig. 6d). This generated a list of 702 genes that distinguish HSC-I, HSC-iM and their progeny.

We then performed dimensionality reduction and clustering of HSPCs and monocytic cell types in the integrated TARGET-seq+ dataset using the Self-Assembling Manifolds (SAM) algorithm and the 702 HSC-I/HSC-iM trajectory genes (Referee Fig. 6e-f). Visualization of the original primary BM cells (where HSC-I vs HSC-iM composition was known) on the resulting UMAP showed effective separation of HSC-I, HSC-iM and their downstream progeny (Referee Fig. 6g). Louvain clustering was performed on the SAM embeddings at a resolution of 1.5, and the

resulting 15 clusters were annotated as being part of the HSC-I or HSC-iM differentiation trajectory based on their composition of primary BM cells from HSC-I and HSC-iM dominant samples and expression of the HSC-I and HSC-iM transcriptional programs (Referee Fig. 6h). We used this categorization to infer whether xenograft cells were of HSC-I or HSC-iM origin.

We then compared the frequency of HSC-I and HSC-iM-derived HSPCs in the xenografts with the frequency of HSC-I and HSC-iM in the primary BM samples. Overall, HSC-I and HSC-iM-derived HSPCs were detected in the xenografts, although the proportion of HSC-iM was generally lower than in the original samples (Referee Fig. 6i). The decrease in HSC-iM derived cells was most significant in NOC117 where xenograft HSPCs are primarily HSC-I-derived despite the original engrafted HSCs being HSC-iM dominant. This can either be attributed to reduced engraftment of HSC-iM compared to HSC-I or interconversion of HSC-I to HSC-iM. As we cannot currently perform lineage tracing robustly (see Referee Fig. 9 and response to point 6), we cannot rule out interconversion for NOC117, but a simpler explanation would be stochastic engraftment from a limited number of HSC-I clones. The latter is supported by the observation that the proportion of *TET2*-mutant cells in the primary BM sample was lower in HSC-I than HSC-iM, and a decrease in *TET2*-mutant cells was observed in xenograft HSPCs (Referee Fig. 6j). These new data are in Extended Data Fig. 8 of the revised manuscript.

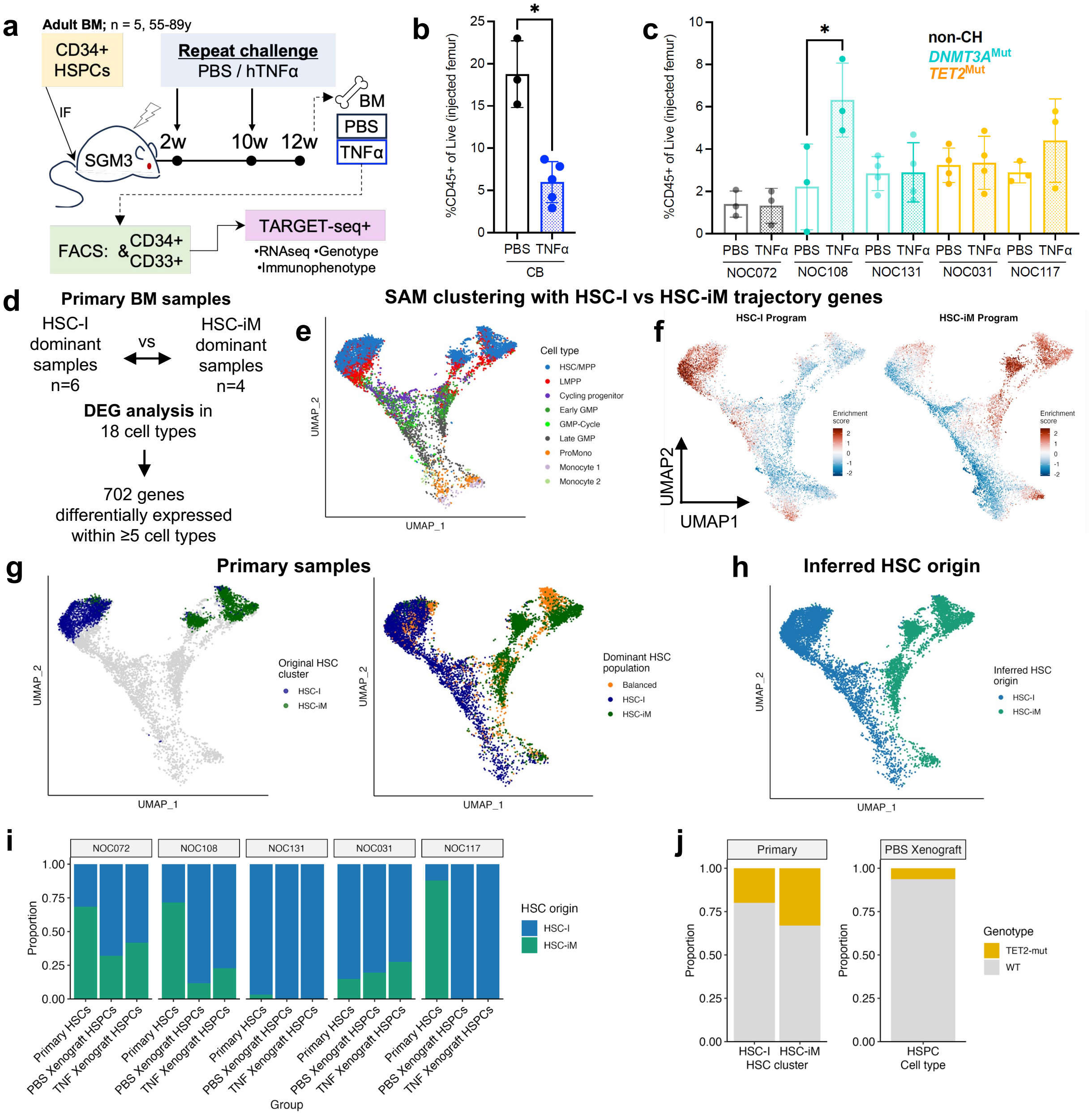

Referee Fig. 6: Repopulation of human bone marrow donors in an inflammation recovery model. a, Schematic for modeling inflammatory challenge with hTNFα in the NSG-SGM3 mouse model. b, Human engraftment from a single CB donor (80,000 cells injected/mouse) is decreased following TNFα repeated challenge compared to PBS controls at 12w post-transplant. c, Human engraftment of CD34+ HSPCs from bone marrow of 5 individuals with and without CH mutations at 12w post-transplant (NOC072: 77,500 cells/mouse; NOC108: 55,800 cells/mouse; NOC131: 90,600 cells/mouse; NOC031: 80.600 cells/mouse; NOC117: 55,800 cells/mouse). d-h, Inference of HSC origin in xenografted samples. Comparing HSC-I- and HSC-iM-dominant samples in 18 cell types identified 702 genes used for subclustering (d). 14,546 HSPCs and monocytic cells from the primary and xenografted samples were subclustered with SAM using the DEGs. The SAM-weighted PCs were used to generate UMAPs showing cell type annotations (e), enrichment of the HSC-I and HSC-iM programs (f), HSC-I and HSC-iM cells and whether cells originate from a sample with a dominant HSC-I, dominant HSC-iM or balanced HSC population (g) and the annotation of the inferred HSC origin derived from SAM clustering (h). i, Proportion of HSC-I vs HSC-iM within the HSCs at homeostasis and within the CD34+ HSPCs isolated from xenograft recipients treated with PBS or TNFα. j, Comparison of the TET2-mutant clone size between primary HSCs (left) and HSPCs isolated from the xenograft (right) for the NOC117 sample.

But then again, these lineage tracing approaches have weaknesses. In the xenotransplant setting, would it be possible to prospectively (e.g. with an AP-1 reporter lentivirus) isolate HSC-iM and re-transplant to confirm that this is a stable population?

Published and preprint studies point to AP-1 as an evolutionarily conserved mechanism for encoding inflammatory memory in tissue stem cells[15] and therapy resistance in the context of cancer[16,17] and we understand the potential value of the suggested experiment. However, human HSCs would have to be transduced, a process that requires several days of *ex vivo* culture, viral exposure and genomic integration. All of these will affect the HSC state, and we do not yet know the effect on the HSC-iM state. Moreover, we have longstanding evidence from studies in the Dick lab that cell culture/lentivector transduction alters/impairs repopulating HSC[18]. Additionally, we present specific new empirical evidence showing that HSPC from a human gene therapy trial for Fanconi Anemia[19] are changed in the long-term in their enrichment of the HSC-iM program as a consequence of lentivector transduction (Referee Fig. 7). Thus, while we agree that usage of reporters would be important in future studies, we first need to experimentally determine exactly what molecular effects and functional consequences are exerted by the transduction itself before we could deconvolute experimental artifacts away from true biology associated with HSC-iM.

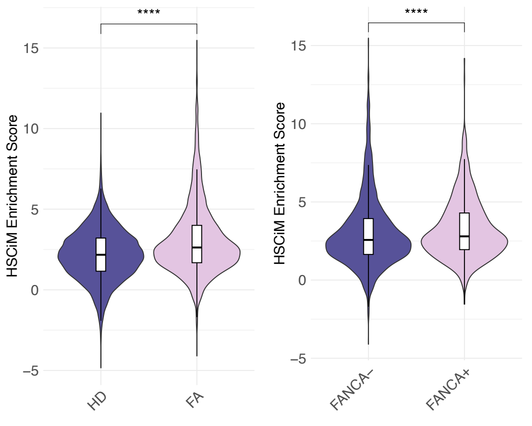

Referee Fig. 7: HSC-iM enrichment analysis following lentiviral gene therapy. a, HSC-iM enrichment in Healthy Donor (HD) and Fanconi Anemia (FA) HSC b, HSC-iM enrichment in uncorrected (FANCA-) and corrected (FANCA+) cells.

3. The findings on non-injected BM are interesting. To summarise, there is a treatment effect after an acute challenge 6 weeks after transplant (Figure S3b,c), but not after 20 weeks (S3d,e). How do the authors interpret this result? Does it mean that long-term HSCs that go into circulation cannot become HSC-iM? Might pre-existing clonal differences between HSCs determine the ability of an HSC to become an HSC-iM or go into circulation?

We would like to clarify that Fig. S3b-e described acute challenge experiments where grafts were analyzed 16 hours after treatment. We cannot explain the reason for the differences between injected and non-injected BM at this time. However, we want to highlight that there is no connection between the acute challenge experiment highlighted by the Referee and the setting with repeated challenge at 2wks and 10 wks followed by identification of HSC-iM at 20w (in Fig. 1n and Extended Data Fig. 2l for NSG, and Extended Data Fig. 2q-t for NSGW41). In the latter experiments, both injected and non-injected BM showed similar changes in engraftment across treatments, though the effect sizes were slightly different. We also note that the scMultiome where HSC-iM was observed was performed on cells sorted from pooled BM cells collected from all long bones. Finally, we draw attention to our results extracted from the severe

COVID recovery dataset, which is derived from circulating HSPCs, providing evidence that HSC-iM can be found in circulation.

4. The xenotransplants were done with cord blood HSCs, is the ability to become HSC-iM different in adult BM HSCs?

As we have demonstrated, the HSC-iM program accumulates in HSCs from adult/aged individuals in several settings. However, to directly address this highly relevant question requires prior knowledge of the HSC-I and HSC-iM status in such adult HSPC samples. Currently, this is only available to us in the CH cohort where we had determined the proportion of HSC-I and HSC-iM with each BM sample (Fig. 5). The Referee may note that no sample is 100% HSC-I or HSC-iM. To answer this question with the highest level of experimental rigor, we would need to identify samples with just one HSC subtype, or have a method for prospective separation of HSC-I from HSC-iM and then transplant in our xenograft models to determine if they can acquire the other HSC state. Such studies are clearly beyond the scope of this first paper describing the discovery of HSC-iM and would be the subject of important future studies.

Nonetheless, as noted above, we are sharing our most recent analysis with the Referee. We were able to transplant CD34+ progenitors isolated from a 79yo non-CH donor (NOC072) and performed a 2x PBS or TNFα challenge (see Referee Fig. 6). The original cells were profiled by TARGETseq+ in our Jakobsen et al study and this donor was found to have a mixed composition of HSC-I and HSC-iM. By co-clustering the cells profiled from these xenografts with the original data, we were able to show balanced distribution of HSC1/HSC-I vs HSC2/HSC-iM HSPC and their monocyte progeny, even with the TNFα challenge (Referee Fig. 8a). We recognize the difficulty to fully attribute the proportions of HSC-I- vs HSC-iM-derived cells observed after xenotransplantation to either stability or conversion between HSC-I and HSC-iM, but these new data support a model for adult HSCs to have a more stable state than neonatal sources.

Finally, we compared transcriptional profiles between monocytes within HSC-I-derived or HSC-iM-derived progeny within the entire cohort of xenografted progeny from 5 BM donors (see Referee Fig. 6). Our analysis is preliminary, because we detected only a limited number of monocytes bearing CH mutations in the xenografts. However, we evaluated for the Referee the enrichment of the HSC-iM and the Hallmark TNFa via NFkB signatures in CH^{WT}, CH^{MUT} and non-CH monocytes (Referee Fig. 8b, c). The presence of the CH mutation results in a modest increase in the Hallmark TNFa via NFkB signature within HSC-I (Referee Fig, 8c). Remarkably, HSC-iM-derived progeny, even in the absence of CH, exhibited stronger enrichment of TNFa via NFkB than HSC-I with CH. Thus, we speculate that most murine models of CH are examining effects of CH on HSC-I. As predicted from the data reported in Fig. 6 and our previously published CH TARGETseq+ study[20], we also observed a trend towards reduced TNFα signaling

in BM HSC-iM-derived monocytes when they possess the CH mutation. These data are preliminary and not included in the revised manuscript.

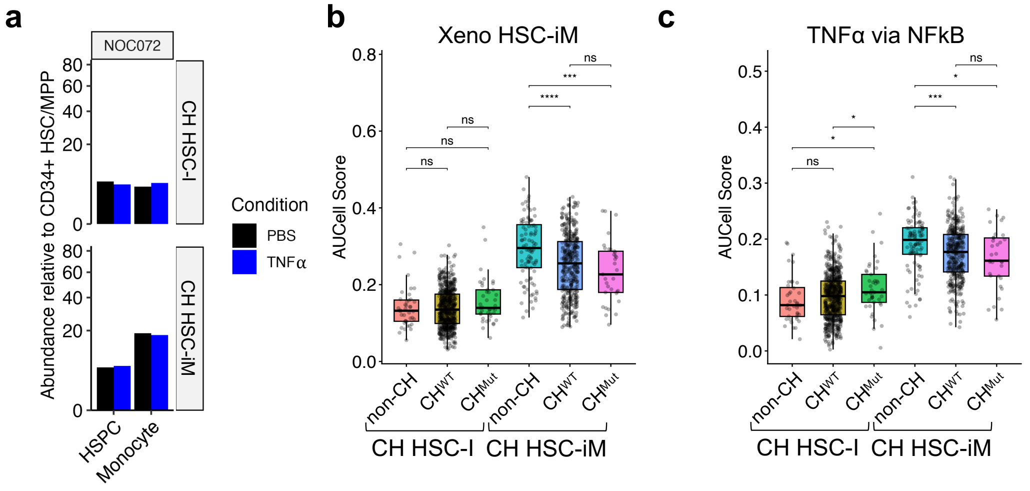

Referee Fig. 8: Assessing HSC-iM enrichment in adult bone marrow progeny after xenotransplantation. a, relative abundance of CD34+ HSPC and CD33+ myeloid cells at 12w post-transplant from a non-CH bone marrow with mixed HSC-I/HSC-iM composition following xenotransplantation (See Referee Fig 6c for engraftment) as assessed by flow cytometry normalized to CD34+HSC/MPP cells transplanted. b, Xeno HSC-iM signature enrichment (AUCell score) and c, Hallmark TNFα via NFkB signature enrichment (AUCell score) in CH HSC-I- and CH HSC-iM-derived monocytes profiled by TARGET-seq+ derived from a non-CH control or CH-mutated samples recovered post-xenotransplantation.

Technical points

5. Documentation of computational methods: I was trying to understand how the y-axis from Figure 4b/c (HSC inflammatory memory program score) was calculated but couldn't find this information. The methods section "ICU-COVID analyses and signature derivation" appears to describe something else. I appreciate the effort of the authors to make code underlying key analyses public, but when I looked at the 8_postCOVID_analyses.nb.html the HSCII_inflammatory_memory_top200_AUC value just got read in from somewhere and I couldn't find how it was generated. Generally these Rmd files should minimally rely on reading in pre-computed information and rather make relevant computations transparent.

We thank the Referee for making us aware of the potential discrepancy in our organization of the methods section. We note that the section refers to where the COVID-related data was obtained and how we identified HSC/MPP from the scRNA profiles, and how we defined the 20 gene COVID-related signature.

The signatures that are labelled in the y-axes were described in the prior section in the Results entitled "HSC-iM share core molecular programs with memory T cells", and the signature is listed in Supplementary tables 8 and 9/12. The derivation of these signatures is outlined in the methods in the sections "Marker gene derivation within CB Xenograft scMultiome data" and "Differential chromatin accessibility within CB Xenograft scMultiome data". The enrichment of

these signatures in the various groups was determined using AUCell or chromvar for transcriptional or ATAC data, respectively, as described in "RNA and ATAC signature scoring". We have also now updated the Figshare directory with the appropriate scripts and have added in additional source data as needed.

6. Lineage tracing analysis: The analysis in lines 500-523 has the goal to check if the inflammatory memory program is inherited from stem cells in a cell autonomous manner. For this, the best experimental design would probably be to uniquely barcode each cell prior to transplant (e.g. with a lentivirus), and check if, for HSCs that acquire the iM phenotype, their progeny is also iM. The authors try to circumvent this by working with a pool of 62 donors, and use genotype as a surrogate for cell of origin.
Question: 8,000-10,000 CD34+CD38- cells were transplanted (Methods). How many stem cells engrafted? The authors probably have limiting dilution data from a similar setup that allows to estimate a rough order of magnitude.
Comment 1: If more than 15 human HSCs engrafted, the bioinformatic algorithm should identify more than 15 "clades". Otherwise they are really comparing all cells that come from donors with, say, African ancestry, to cells that come from donors with, say, Asian ancestry. This is quite detached from what this experiment is trying to address.
Comment 2: If more than ~30-40 human HSCs engrafted, it would be better to repeat this experiment with lentiviral lineage tracing, since otherwise this experiment is not well suited to check if the clonal progeny of HSC-iM is also iM.
We would be expecting 60-75 HSCs from the initial dose we used. Note that clonal selection is a known consequence of transplantation[21]. Also note that these 60-75 HSCs would have stochastically been derived from any of the 62 donors in any given mouse. The analysis to identify genetic clades was performed iteratively, and we determined the number of clades using an elbow plot of the total log probability as well as concordance with predicted sex assignments. We reasoned that being able to reliably identify the 15 clades was a sufficiently representative model of donor-derived heterogeneity. The Referee has concerns that we may simply be sampling donors of related ancestry; while we are not privy to any identifying information from the donors, including race/ancestry, we believe 15 clades is substantially more than that which can broadly be assigned to ancestry. Finally, we agree with the Referee that comparing the molecular profiles of HSC across ancestry is not the point of our study; we would note that we do not entirely rely on direct comparisons between any two single clades, and importantly we are unlikely to be capturing ancestral variation in all of the datasets we have interrogated, which provide key ancillary evidence to our findings from the xenografts.

Although we had prepared to attempt a lineage tracing experiment and have obtained a lentiviral library, we learned of work from our colleague Dr. Elisa Laurenti (University of Cambridge) who is a leading expert in human HSC studies. Her group has made extensive efforts over several years to make lentiviral barcoding-mediated clonal tracing work *in vivo* with

xenotransplantation, but to little avail. In her latest attempts, although 20,000 GFP+ CD34+ HSPCs subjected to LARRY lentiviral barcoding were transplanted and ~12,000 clones/barcodes were expected 10 weeks after xenotransplantation, a clonal diversity of only 23 clones was found. She has kindly shared her unpublished data with us, shown in Referee Fig. 9 for the Referee. We speculate that lentiviral transduction coupled to transplantation likely creates massive compression of clonal diversity. In addition, we have shown the Referee the potential effect of lentiviral transduction on HSC-iM enrichment in Referee Fig. 7. Thus, the approach of lentiviral barcoding to map cell fates is not currently technically informative for our experimental models.

Redacted

Manuscript readability

7. The manuscript would benefit from shortening and removal of supplementary items, in particular at the level of omic data description. E.g. in lines 218-294, if RNA-seq, ATAC-seq and

GRN analyses all point towards a common set of pathways differing between HSC-I and HSC-II, I don't see a need to spread this out over six paragraphs and tens of figure panels.
We have made considerable efforts to streamline the data presentation and the results. Original and newly generated supporting data deemed necessary have been organized into 10 Extended Data Figures. This specific computational analysis has been moved into Supplementary Note 2 of the supplementary materials.

Minor points

In figure S22e,f why are only 14 out of 15 clades shown? Also, the information here is not consistent with what is written in the text (6 were HSC-I specific in the text, and 8 in the figure)
The number of HSC-I-specific clades was 8 in the original submission. Furthermore, we originally conducted our analyses with only 14 of the 15 clades detected, as one was composed of too few HSPCs to make meaningful conclusions. In the revised manuscript, we have performed scRNAseq analysis of the CD34+CD38+ progenitors isolated at the same time as the other xenografted populations and have re-derived the genetic clades. We still find 15 clades, but now have enough cells in each to include all 15. Specifically, 9 are now HSC-I dominant, 3 are HSC-iM dominant and 3 are mixed composition.

Single cell analysis: In the uMAP in Figure S1K, donors separate. It is worrisome that different cell types (Figure S1J) differ in abundance across donors (comparing S1K and S1J), this is unlikely to be real biology? Should a different data integration/batch correction method be used here?
We have included a plot to indicate the proportion of the relevant cell types within each donor (Referee Fig. 10a); the Referee will note that there is no meaningful difference in abundance across donors.

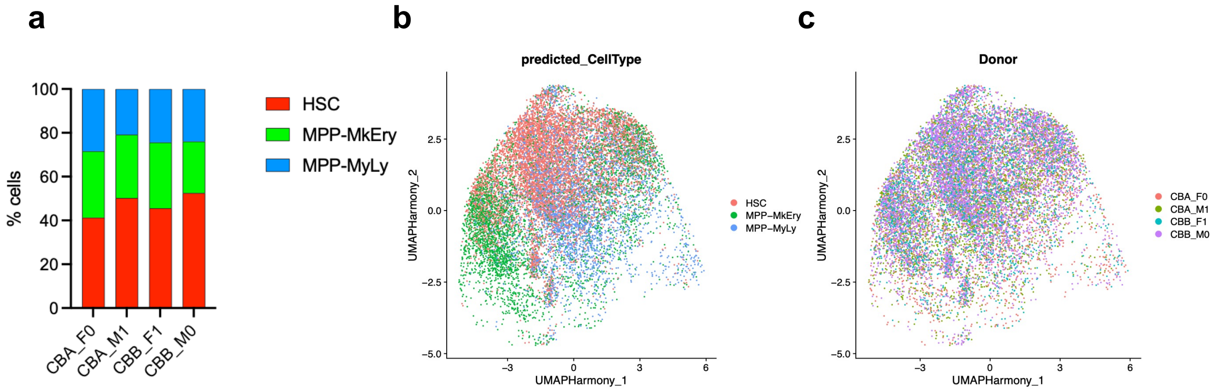

Referee Fig. 10: QC of CB scMultiome. a, Proportion of HSC, MPP-MkEry, and MPP-MyLy from each CB donor. b-c, UMAP with batch correction by cell type (b) and donor (c).

We also appreciate the Referee's concern regarding batch correction. We note that, for the sake of transparency, we did not correct for donor or sex when presenting this specific data, so that the donor-driven differential enrichment of inflammation priming would be transparent.

However, as shown in Supplementary Fig. 1h (part of Supplemental Note 1 in the revised MS), there is also substantive intra-donor heterogeneity in inflammation priming. To ensure a fulsome response, we have included UMAPs with batch correction (Referee Fig. 10b-c) to show that relevant cell types cluster together and that cells from different donors can be evenly distributed.

Referee #1 (Remarks on code availability):
See my main report.

Referee #2 (Remarks to the Author):

This manuscript addresses an interesting and very timely topic of inflammation and its effects on human HSCs and the longevity and integrity of the hematopoietic system. The effects of inflammation on HSCs have been quite extensively studied in mice, as it has important implications for human disease. Experimental in vivo models to study inflammation in human HSCs are lacking for obvious reasons, and primary samples are not easy to get access to, and have many confounding factors. To address this gap, this paper creates an experimental model to study inflammation in human HSCs, and compares their cord blood HSCs exposed to inflammatory stimuli after transplantation to immunodeficient mice to various data sets where inflammatory HSCs are likely to be present.

The manuscript first utilizes scRNAseq and Multiome (scRNA/ATACseq) to identify two distinct subsets of human cord blood HSCs, and show that one of the HSC subsets displays an inflammatory signature. After establishing this inflammatory HSC population in cord blood, they document the presence of a similar population in an inflammation stimulus immunodeficient mouse model that they create by exposing mice transplanted with cord blood HSCs to inflammatory stimuli, and finally correlates the findings to previously published data sets from patients recovered from COVID as well as patients with clonal hematopoiesis (CH) caused by distinct mutations. Distinct from the previous studies published by the same team of senior authors (CH) and others (COVID), the current manuscript creates an inflammation stimulus mouse model for human HSCs where these concepts can be experimentally addressed.

Apart from the generation of the inflammatory stimulus mouse model after cord blood HSC transplantation and comparing the scRNAseq data from the mice to various data sets where HSCs have been exposed to diverse inflammatory stimuli, the manuscript is largely relying on bioinformatics analysis of various data sets. While the manuscript contains a lot of sophisticated bioinformatics analysis, such analysis are not always easy to translate to practice to make the conceptual advance easily accessible for readers. The authors could take the analysis and validation of the identity of inflammatory HSCs further and improve data presentation to make it easy for others to pinpoint inflammatory HSCs in diverse human samples and data sets, and thereby make the findings more accessible to the broader readership of general interest journals.

1) Can the authors identify a surface marker or other cellular features that can be assayed that would enable prospective isolation of the inflammatory HSCs? It would be ideal for future follow up studies if these cells could be enriched and tested for their epigenetic memory in experimental models. Were there any surface markers enriched in the inflammatory HSC subset?
We agree that prospective isolation of HSC-iM represents a logical next step beyond our current study. Prospective isolation of HSC-I and HSC-iM has been a goal we have been tackling since we first identified these subsets, but with all our best efforts, including using a state-of-the-art

Cellular Indexing of Transcriptomes and Epitopes by Sequencing (CITE-seq) panel validated on young human bone marrow HSPCs[22], we have not found a set of reagents that are adequate for prospective isolation of HSC-iM from HSC-I. Our efforts are summarized below.

First, we took a single candidate approach. We share with the Referee the set of differentially expressed transcripts between HSC-I and HSC-iM encoding cell surface markers across both the CH bone marrow TARGETseq+ dataset (y-axis) and the xenograft scMultiome (x-axis; Referee Fig. 11). We surveyed available reagents to stain for these cell surface markers and have evaluated 15 candidates by flow cytometry of CD34+ cells from adult bone marrow samples, where we expect heterogenous presence of HSC-iM. Some candidates are expressed on all immunophenotypic HSCs and primitive cells while others were not found on any primitive cells at baseline. This endeavor did yield two very interesting targets, namely ATP2B1 and CD83, resulting in novel human HSC biology. However, these do not segregate HSC-I from HSC-iM. Instead, surface expression of the calcium transporter ATP2B1 (Varesi, Nagree, et al, in revision; https://www.biorxiv.org/content/10.1101/2025.06.11.659017v1) marks more quiescent, self-renewing LT-HSC, as opposed to directly being associated with an inflammatory state. CD83 is an exquisitely sensitive biomarker for human TNFα response on human LT-HSC from both neonatal and adult HSC sources. These data indicate CD83 is only primed for expression at the mRNA level at homeostasis, but upon exposure to TNFα gains protein surface expression as summarized in previous conference publications [see https://doi.org/10.1182/blood-2023-187737 and https://doi.org/10.1016/j.exphem.2024.104524].

Redacted

Referee Fig. 11: Identification of candidate surface markers for HSC-iM by correlation of differentially expressed genes in the CH bone marrow and the xenograft data sets.

We turned to an unbiased approach, and we performed a new experiment in collaboration with Dr. Lee Grimes - a leader in this area - to identify cell surface markers distinguishing HSC-iM from HSC-I using a validated CITEseq panel[22]. We leveraged BM from our inflammation-recovery xenotransplantation models with either a single or repeated challenge of PBS, TNFα, or LPS. We present the results in Referee Fig. 12; these are not included in the revised manuscript.

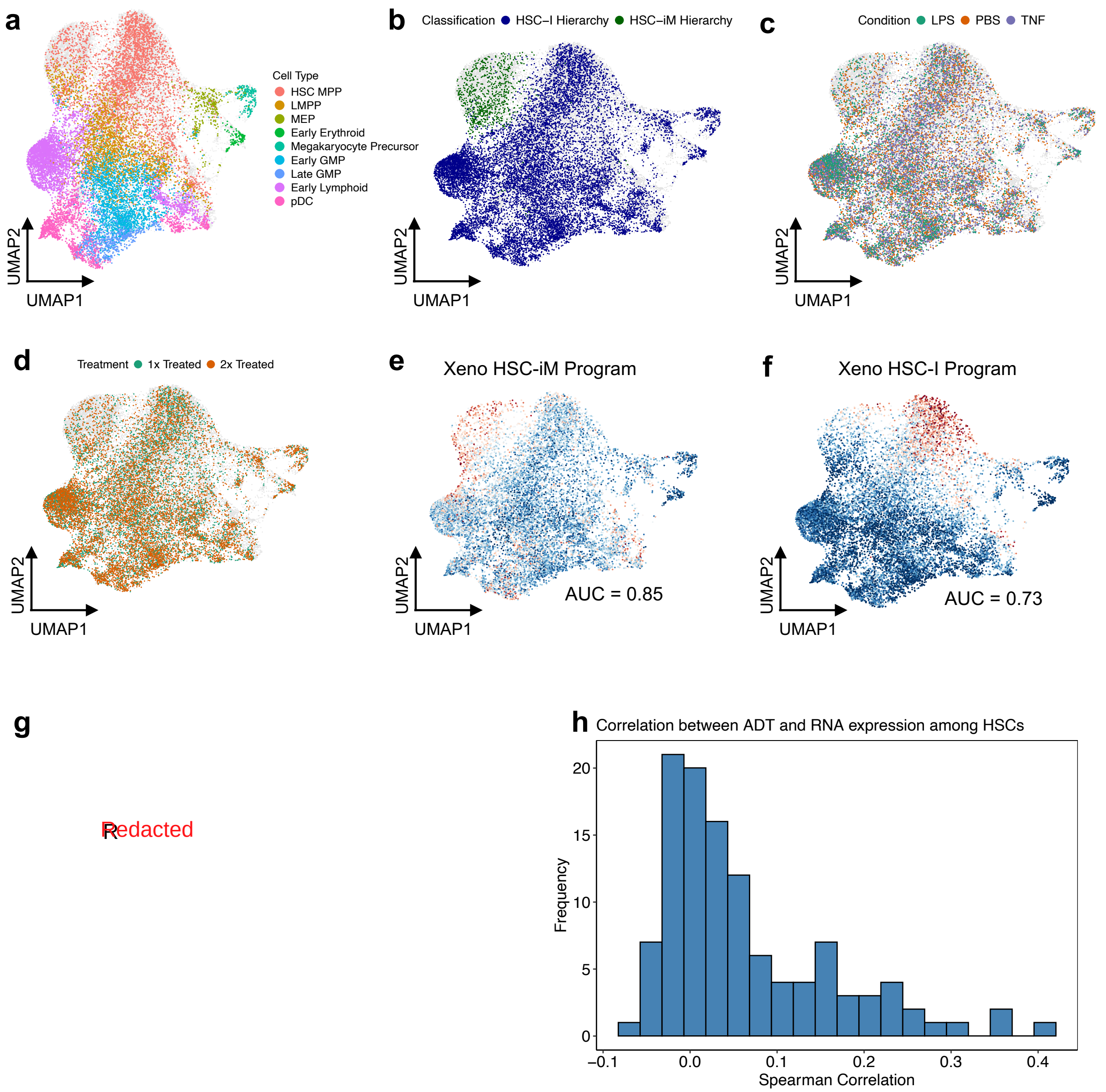

Referee Fig. 12: CITE-seq profiling of inflammation-recovery xenografts. a-d, UMAP of joint RNA integration of CITE-seq and RNAseq from the original xenograft scMultiome. The 30,000+ CITE-seq cells are colored broadly by their predicted cell type (a), hierarchy classification (b), treatment condition (c), and treatment frequency (d). The cells from the original xenograft are in gray. e-f, Normalized signature enrichment scores (AUCell) of the Xeno HSC-iM or HSC-I program overlaid on the CITE-seq HSPCs, subset from the joint RNA integration with the original xenograft scMultiome. g, Differential ADT signal between the HSC, MPP, and LMPP populations from the HSC-iM and HSC-I hierarchy. The highlighted surface proteins have a significance at FDR < 0.05. AUC closer to 1 would indicate ability to discriminate between the two populations. h, Histogram depicting the spearman correlations between the surface protein antibody signal and the corresponding RNA gene expression, among the HSCs in the CITE-seq dataset.

Details regarding the CITE-seq experiment: We profiled both transcriptome and cell surface proteins in the same cells, focusing on CD34+CD19- HSPCs spiked with CD34-CD19-CD33+ myeloid cells at a 10:1 ratio. To classify cells as HSC-iM or HSC-I, we integrated HSPCs from the new CITE-seq dataset with those from the original xenograft dataset, leveraging the shared xenograft experimental background to justify co-clustering based on transcriptional similarity.

Specifically, clustering was guided using the 2000 highest variable genes from the original xenograft, and batch correction was applied for dataset and sex. Notably, across all treatment conditions and frequencies, we identified cells that co-clustered with either the HSC-iM or HSC-I populations from the original xenograft, each showing enrichment for their respective transcriptional programs (Referee Fig. 12a-f). To optimize reliable nomination of cell surface markers capable of distinguishing relevant populations, we ran TotalVI to denoise the antibody-background signal after utilizing the validated panel of 134 antibody-derived tags (ADTs). Next, to nominate potential cell surface markers that distinguish the HSC-iM and HSC-I hierarchies, we performed differential enrichment analysis using a linear mixed model to account for cell type, restricting the analysis to HSC, MPP, and LMPP populations (Referee Fig. 12g). However, no cell surface markers demonstrated a consistent ability to reliably distinguish the two hierarchies. Nominating cell surface markers has proven challenging, partly explained by the weak correlation between RNA expression and signal related to cell surface protein abundance among the HSCs (Referee Fig. 12h). This aligns with a recent report that found a large discordance between RNA and protein composition when mapping human HSPC differentiation with single cell proteomics and transcriptomics[23].

We suspect that the most reliable method for prospective isolation of HSC-iM will be a functional reporter, as also highlighted by Referee #1. However, introduction of such a reporter would require a genetic perturbation, which is likely inflammatory and could potentially confound results (see Referee #1, point 2). Future studies will require experimental identification of confounding transcriptional and epigenetic changes associated with genomic perturbations so they can be dissociated from HSC-iM.

2) What is the significance of inflammatory HSCs present in newborn cord blood?
- The manuscript begins with identification of distinct population of inflammatory HSCs in newborn cord blood samples. Since these cells are obtained at birth from healthy pregnancies, it is not clear what these distinct subsets mean at this stage of ontogeny. In theory, all HSCs should have been exposed to the same in the same "protected" utero environment, unless they derived or resided in specific niches. At the same time, inflammatory signaling is required for HSC development and physiologically important, so the exposure to inflammatory signals is not unexpected.

We are not claiming to find HSC-iM directly in CB. We would like to clarify that the transcriptional heterogeneity we report in Fig. 1 was identified using a machine learning-based method, cNMF, which detects gene expression programs in an unbiased manner. Furthermore, LT-HSC were sorted from >15 cord blood units and then processed as a pool for scRNAseq to generate the data in Fig. 1a-e. Therefore, it is difficult to know which CB units contributed to the single-cell transcriptomes that passed QC. Further, these LT-HSC at 'homeostasis' did not separate into easily distinguishable clusters as we observed in our xenograft model. Thus, in a formal sense inflammatory "memory" - the essential definition of HSC-iM - was not identified in

CB; this required the experimental xenograft system. Finally, our REB requires fully anonymized sampling, so we do not have information about the health status of the mother.

Although we cannot easily speculate on the significance of “NMF2-Inflammation”, we would also like to clarify that the 200 leading edge genes that represent this cNMF-derived program (supplementary table 2) only have 5% overlap (19 genes; NFKB1, RANBP2, MCL1, KLF6, PSME4, TPM4, DUSP2, KMT2E, USP9X, CHD1, TM9SF3, PPP1R15A, REL, SPAG9, GNAS, FOSB, PIM3, KDM6B, H3F3B) with the 200-gene HSC-iM signature we established from our xenograft model (supplementary table 13: “HSCII_inflammatory_memory_top200”). Thus, the NMF-derived gene program that we hypothesize to be an inflammation-primed state of CB LT-HSC at homeostasis is distinct from the experimentally derived HSC-iM signature. At present, we have no direct evidence that HSC-iM exists as a cellular entity in CB. However, upon engraftment in xenografts and upon inflammatory challenge, we see them emerge. A major question for the field - that will require both prospective isolation and lineage tracing capacity - will be whether CB contains a select subset of HSC that is primed to respond to inflammation and from which HSC-iM form upon inflammatory exposure and/or regenerative stress. Alternatively, all the CB HSC may equally but stochastically be able to form HSC-iM.

3) Can the authors identify a distinct single cell signature for inflammatory HSCs that is broadly applicable across different data sets, and could be used for easy identification of the presence of such cells?

We agree with the Referee that the identification of a conserved HSC-iM signature that is applicable to cells exposed to different inflammatory environments would bolster the strength of our claims. To this end, we turned to scRNAseq datasets of HSPC obtained from varying contexts of inflammation, some evaluated during this revision process. In order to identify HSC-specific gene expression signatures from these datasets, HSC/MPP were isolated via BMRefMap projection and consensus non-negative matrix factorization (cNMF) was used for *de novo* identification of gene expression modules in an unsupervised manner (Referee Fig. 13a).

Within HSC/MPP, we identified NMF programs corresponding to HSC-iM within each dataset. Each of these NMF-derived programs were highly enriched in the experimentally-derived HSC-iM program (relative to HSC-I) by GSEA (Referee Fig. 13b). The HSC-iM program was, in turn, highly enriched along the top ranked genes from each NMF program (Referee Fig. 13c). We developed a consensus program based on the top ranked genes from each NMF signature, and validated that it successfully separates HSC-iM from HSC-I within the xenograft data (Referee Fig. 13d).

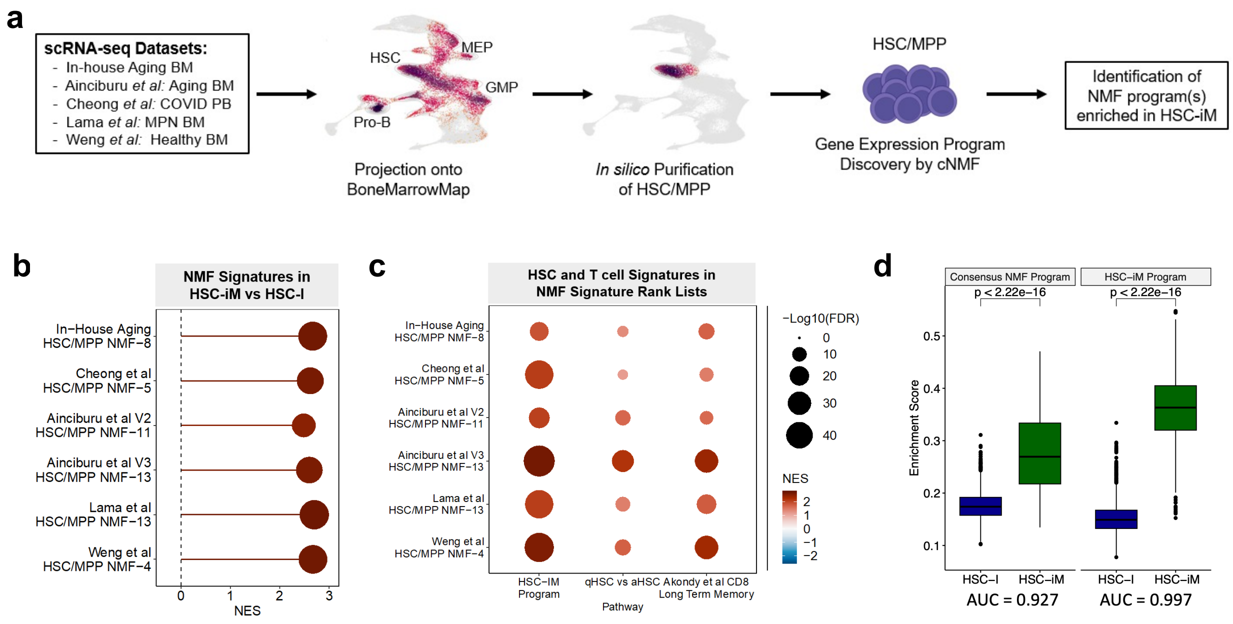

Referee Fig. 13. Evaluation of a conserved inflammatory memory signature across inflammatory datasets. a, HSC/MPPs from each component dataset (in-house Aging Bone Marrow, Cheong *et al* COVID Recovery, Ainciburu *et al*, Lama *et al*, Weng *et al*) were purified *in silico* using BoneMarrowMap, and cNMF was performed to identify gene expression modules in an unbiased manner. NMF modules were then evaluated for similarity to the HSC-iM program. Ainciburu *et al* was split into two batches based on 10x sequencing technology. b, Enrichment of HSC-iM-like NMF signatures within the HSC-iM vs HSC-I rank list by GSEA. c, Enrichment of HSC signatures within each NMF signature by GSEA. d, Boxplot depicting the enrichment of a consensus meta-program derived through integration of NMF signatures from all six datasets within xenograft-derived HSC-iM and HSC-I.

Thus, we consistently identify HSC-iM-like programs in multiple datasets across varying inflammatory contexts, and we can combine these into a single meta-program with strong concordance to our xenograft-derived HSC-iM Program (Referee Fig. 13d). However, our emphasis in this present study surrounds the distinct HSC-iM state which we first defined within our xenograft dataset, and these same data show that our previously identified HSC-iM program is superior for discerning cellular state in our dataset while remaining concordant with *de novo* NMF programs in other datasets.

Thus, our original HSC-iM program consisting of the top 200 HSC-iM-defining genes from our xenograft experiment remains best for validation in other datasets.

4) How similar are the inflammatory HSCs obtained from different sources or interventions, or do they each have unique features? The manuscript is focusing on identifying similarities in HSCs that have been exposed to various inflammatory signals, as well as aged HSCs. Given that inflammatory signaling is also part of physiological HSC development, is there a way to distinguish inflammatory HSCs exposed to "physiological inflammatory signals", vs those that have had dangerously high exposure to inflammatory signaling, or have already been compromised during ageing process?

In our response to the previous question from the reviewer, we have demonstrated that HSC-iM programs can be identified *de novo* from different datasets and biological contexts, and that there is a high degree of similarity between these programs. Whether other inflammation programs exist in HSCs, the distinct physiologic or pathologic associations of these additional programs is out of scope of this story, but we can share results from a more comprehensive cNMF analysis that will be published in the future.

In Referee Fig. 14, we combined 500+ cNMF-derived gene expression programs from 500,000+ HSC transcriptomes across 38 studies to discover 16 transcriptional meta-programs reflective of various facets of HSC biology, including five distinct meta-programs related to inflammation, of which four align with the experimentally determined HSC-iM. The existence of these distinct programs further support that memory of inflammation are separate hallmark transcriptional components of HSC biology found in primary human HSC. These distinct inflammation-related meta-programs exhibit different patterns of enrichment across different biological contexts. For example, in the context of CH which can be considered more akin to "dangerously high" exposure to inflammation, mutant cells exhibit significant upregulation of some meta-programs (DNMT3A: MP5, MP10; TET2: MP5, MP10, MP14) and downregulation of others (DNMT3A: MP2, MP15; TET2: MP15).

While the biological significance of these specific patterns is out of scope for this study, these data serve as proof of principle for the diversity of distinct transcriptional programs in HSCs related to inflammation and other processes. Unbiased derivation of HSC transcriptional programs could provide additional insight into health conditions. Our group has made significant progress towards generating such a framework for human HSC transcriptomic heterogeneity, and have presented these data at international meetings (please see 10.1182/blood-2024-212079 and 10.1016/j.exphem.2023.06.282).

These 16 HSC meta-programs are in the process of being interrogated in the Ontario Health Study (see relevant description in response to Referee #1 point 1 and Referee Fig 3). This will allow various facets of inflammation to be linked to all-cause mortality and aging, independently, via the IRS score, as well as more specific clinical outcome data for various diseases. The ultimate goal of this separate study is to identify new biology and new biomarkers that can be used in a similar fashion to the risk score for AML[24], to predict disease-related outcomes.

Redacted

5) How long is inflammatory memory retained, and does it depend on the type or duration of stimulus that cause it?

Here is what we have learned from the various datasets we have examined:

1. In Xenografts: at least 10 weeks (time from the second challenge) in the bone marrow.
2. In peripheral blood HSCs isolated from severe COVID19 recovery patients: up to 4 months. We did not observe HSC-iM enrichment in the 1 year follow-up data from Cheong, et al.[14], but cannot rule out potential differences in the ability of HSC-iM cells to migrate into the blood.
3. In Bone Marrow from aging/CH cohorts: while we do not have start/end dates for underlying inflammatory events, we know that there is an accumulation with age.

In summary, at this point we can only say up to 2-4 months from an inflammatory stimulus in patients. We would need additional studies with longitudinal samples to specifically assess retention of inflammatory memory.

There is a gap in the literature in fundamental work that investigates the impact of individual inflammatory stimuli on early human hematopoiesis that is required to address the Referee's question. As humans are subjected to inflammatory challenges over their lifetime, longitudinal sampling from an early age would be required alongside clear documentation of the nature and occurrence of various inflammatory events including infection, injury and age-related diseases. The easiest tissue to access would be blood. The obvious implication is that this would be best done in a prospective cohort in a study designed to answer this question, which is obviously out of scope for this manuscript. We have, however, begun to look at data from OHS, as described in Referee#1 point 1, Referee Fig. 3.

One caveat to such work is that blood may not fully mirror bone marrow[25]. To begin investigating such concepts experimentally we have evaluated the impact of specific stimuli - including LPS, TNFα, IL1β, IFNγ, IFNβ and IL6 - directly on human LT-HSC across the first 70 hours in *ex vivo* culture as part of an investigation of CD83 and its role in HSC inflammation (see Referee Fig. 30). TNFα is the strongest inducer of CD83 surface expression, but there are significant changes for other cell-phenotypic measurements amongst the various stimuli. We have also undertaken a study to assess transcriptional changes by TNFα (versus PBS control) in purified LT-HSC, but these changes are quite distinct from the HSC-iM state (Referee Fig. 5).

6) Does the age /developmental stage of HSCs influence how they respond to inflammatory stress in the experimental models?

To begin addressing this question, we xenografted progenitors isolated from some of the adult bone marrow donors with and without CH included in this study for whom the HSC1 vs HSC2 ratio was known (see Referee Fig. 6). Unlike experiments with cord blood, we did not observe a decrease in engraftment with the adult samples. In one case, we even observed increased engraftment following TNFα challenge when a carrier of a *DNMT3A* mutation (NOC108) was

used. Results from this one sample align with a murine study of TNFα challenge in *Dnmt3a*-driven CH[26]. These preliminary data indicate that developmental stage and CH status/age influence how human HSCs respond to inflammatory stress in xenograft models. However, more adult samples from various physiological scenarios would need to be evaluated as part of a separate study for a fulsome answer.

7) How well does the immunodeficient mouse BM mimic the inflammatory environment in human? Some of the inflammatory cytokines are secreted from the stroma, are they species specific? Does the fact that the mouse is immunodeficient and lacks many critical immune cells compromise the inflammatory signals that the transplanted cells are exposed to after stimuli?
As far as we are aware, a broad survey of human and mouse cytokines has not been reported in xenografts. We undertook a multiplex assay to measure human (n=23) and mouse (n=18) cytokines in the plasma of xenografted mice 6w post-engraftment before and after an inflammatory challenge with LPS (Referee Fig. 15). All human cytokines were detected above the detection limit in at least one mouse. We observe that many human cytokines are being produced even at baseline and in the PBS treatment group, explaining why xenografting is such a valuable model. We also found that 19 of 23 cytokines significantly changed upon LPS challenge, indicating that a broad human inflammatory response occurs in xenografts. We have added these data into Extended Data Fig. 2 of the revised manuscript and Supplementary Table 5. We believe our new data will be a valuable reference to the entire field that uses xenografting.

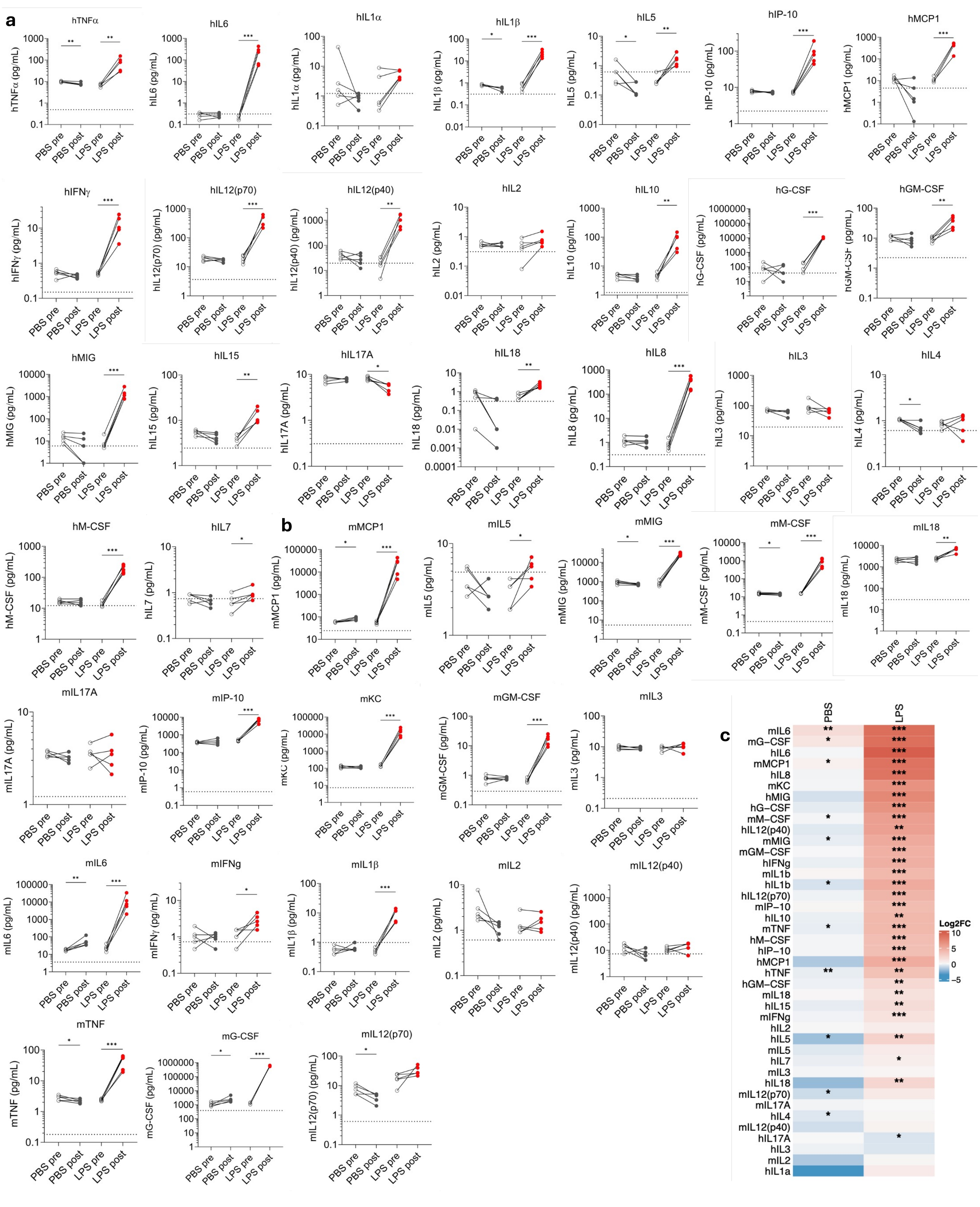

Referee Fig. 15: Multiplex cytokine profiling of blood plasma at 6 weeks post-xenotransplantation. Blood plasma from xenografted mice (n=5 per group) was sampled before and after an acute challenge with PBS control or LPS (n=5 per group) for a, human (n=23) and b, mouse (n=18) cytokines. The dashed horizontal line line shows the lower detection limit. Lines relate matched samples. c, Heatmap of all data. paired-ratio t tests. *** p<0.001, ** p<0.01, * p<0.05

8) The manuscript is somewhat tedious to follow, as it is almost entirely graphs and figures illustrating bioinformatics analysis, and it does not render the data and findings easily accessible for the broader readership. Below are some suggestions summarized that could be added to make the findings more accessible and easy to apply also in other settings.
We thank the Referee for this suggestion which echoes that from Referee #1. Consequently, we have considerably streamlined the manuscript. Supporting computational analyses are provided in Supplementary Notes 1 and 2 of the Supplementary Materials. Specific questions are addressed below.

a. Can the authors identify surface marker candidates to isolate inflammatory HSCs?
We have not found a surface marker that can prospectively isolate HSC-iM, despite significant effort and cost. See response to point 1 and Referee Fig. 11 and 12.

b. Can the authors create molecular signatures from the single cell data that help distinguish the two different HSC subtypes, and help identify iM-HSCs from various data sets using dot plots or other features that are easy to visualize and recapitulate without having to do complex bioinformatics analysis?

We recognize the value of what the Referee is proposing and have made considerable attempts to undertake an analysis that would simplify classification of HSC-iM and HSC-I. We took a few approaches. First, we attempted to create a Symphony Reference Map (akin to BoneMarrowMap) using a combination of the original xenograft and CH datasets. Data from a query dataset would be 'projected' onto this reference map to classify HSCs. Second, we attempted to co-cluster HSCs from query datasets with the original xenograft; this requires batch correction and prioritization of features that specifically define the HSC-iM cluster. Despite a lot of effort to optimize these methods, both have technical shortcomings and do not eliminate batch effects, such that the interpretation of biological meaning of the output is affected.

Finally, to exemplify the significant effort we made with various approaches to capture the molecular features of HSC-iM, we describe in detail our workflow with starCAT[27]. This tool uses a new module scoring approach that uses the weights of thousands of genes that define the HSC-iM/HSC2 state in the xenograft and CH datasets. Utilizing this new weighted scoring approach in combination with our current Top 200-gene HSC-iM AUCell enrichment score enables clear separation of the HSC-iM and HSC-I state in both the xenograft and CH datasets, exhibiting a bimodal distribution (Referee Fig. 16a,b). However, in some datasets, such as the COVID dataset, the distribution of scores is unimodal, making it difficult to define clear thresholds for HSC classification (Referee Fig. 16c). The module scores show variation across datasets that may reflect underlying biological differences but also could result from technical batch effects, such as differing library preparation technologies. Consequently, it is challenging

to compare the composite gene expression scores between datasets with confidence, and thus we are unable to define universal parameters and thresholds for HSC classification.

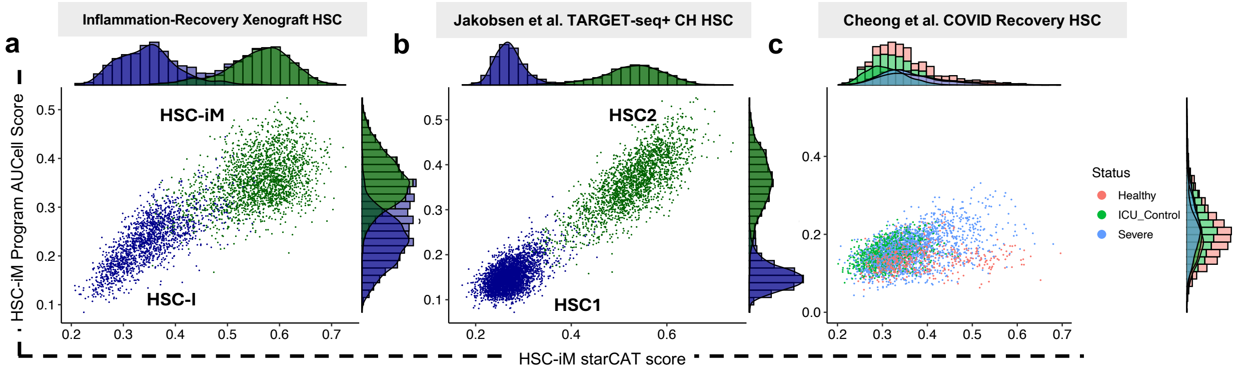

Referee Fig. 16: Classification of HSC using molecular signatures of HSC-iM. a-c, Scatter plots depicting the correlation between the HSC-iM AUCell program enrichment scores derived using the top 200-gene program (y-axis) and weighted scores derived using starCAT (x-axis). Density distributions of each score are shown along the respective axes. (a), Module enrichment scores in the Inflammatory Recovery Model Xenograft HSCs. (b), Module enrichment scores in the Jakobsen et al. TARGET-seq+ CH HSCs. Dark green points and distributions represent HSC2 cells, and dark blue represent HSC1 cells in (a & b). (c), Module enrichment sores in the Cheong et al COVID Recovery HSC. Points are colored by condition.

c. The Multiome/ATAC seq data is presented as UMAPs, and there is little “visual” data to help the reader grasp what the epigenetic changes are. It would be helpful to see examples of altered accessibility of TSS or enhancers in genes that change upon inflammatory stimuli, or in CH or COVID patients, and are representative of the epigenetic memory.

In our original manuscript we illustrated the number of peaks that are specific to HSC-iM and highlighted how these peaks were significantly enriched in binding motifs for the AP-1 transcription factor family (Fig. 2 and Extended Data Fig. 4f,g in the revised manuscript). As suggested by the Referee, we have provided some examples of specific epigenetic changes (see Referee Fig. 17) for the readers which have been added to Extended Data Fig. 4i in the revised manuscript. We show two examples of loci that are accessible only within HSC-iM populations. The AP-1 motifs within these loci are designated as cis-regulatory elements based on statistical linkages made between chromatin accessibility and gene expression from the scMultiome data. In concordance with their increased accessibility in HSC-iM, we observed increased expression of their associated genes (*LMNA* and *SEMA4A* in the first example; *EZR* and *SYTL3* in the second example). This new representation will help provide readers with a stronger visual understanding of how the epigenetic and transcriptional changes fit together to establish the HSC-iM state.

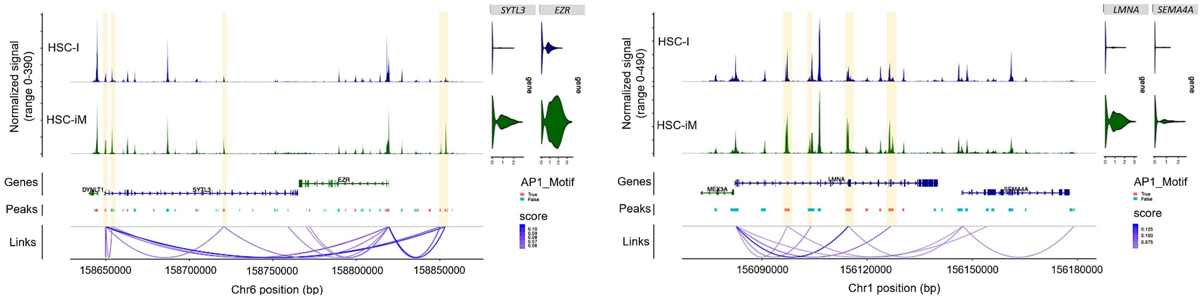

Referee Fig. 17: Cis-regulatory linkage between HSC-iM marker genes and proximal accessible chromatin regions. Examples of chromatin regions along select HSC-iM marker genes for which *cis*-regulatory relationships could be inferred. The expression of these genes between HSC-I and HSC-iM are shown as violin plots on the right-hand side. Chromatin accessibility in the vicinity of each gene is visualized for the HSC-I and HSC-iM populations. Differentially accessible peaks at FDR < 0.01 are highlighted with yellow bands. Peaks containing AP-1 motifs are also indicated in each plot. Finally, Peak-to-Gene linkages from Signac are shown, which infer *cis*-regulatory relationships between chromatin peaks and target gene expression.

d. The authors show that the ATAC seq data correlates well with gene expression. In addition, it would be interesting to identify accessible or closed regulatory sites that serve as evidence past exposure to inflammatory signals, or predict predisposition for expression in the future, without necessarily being actively expressed?

We have focused on differentially accessible chromatin regions (DAR) with concordant gene expression changes when comparing HSC-iM to HSC-I in order to simplify our biological message. However, it is certainly interesting to consider DARs which were not associated with gene expression changes at the time of sampling. To distinguish between these two types of DARs we will term them 'active' and 'inactive'. For each DAR we identified the most proximal gene and asked whether those genes happened to also be differentially expressed (DEGs) between HSC-iM and HSC-I (Referee Fig. 18).

We identified 353 DEGs linked to DARs, of which 276 were upregulated in HSC-iM (Referee Fig. 18a). We considered these DARs as 'active'. We compared the characteristics of active DARs to 'inactive' DARs not linked to any DEGs. HSC-iM-specific DARs showed similar expression patterns in our UMAP regardless of whether they were active or inactive (Referee Fig. 18b-c). We asked whether any differences in TF binding motifs may exist between active and inactive DARs. We found high concordance between both classes in motif enrichment (R^2 = 0.97) with AP-1 family member binding sites being most abundant (Referee Fig. 18d). In our interpretation, this is in keeping with the known role of the AP-1 family as pioneering transcription factors capable of reshaping the chromatin landscape, which appears to explain a sizable proportion of the chromatin changes within our inflammatory memory context. The preponderance of inactive DARs may provide future utility for developing chromatin-based biomarkers of inflammatory memory in HSC and their progeny.

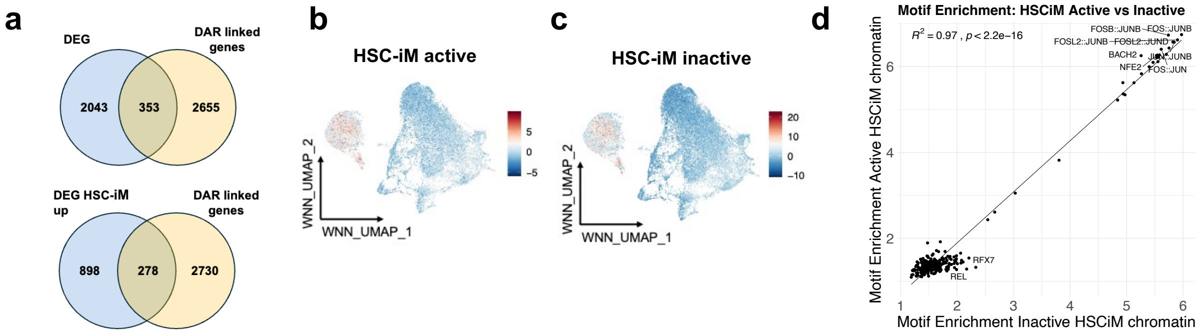

Referee Fig. 18: Inference of accessible and transcriptionally active chromatin sites in scATACseq. a, Venn diagram of genes linked to differentially accessible chromatin regions in HSC-iM and all transcripts differentially expressed (top) or that are upregulated (bottom), in HSC-iM vs HSC-I. Thresholds: padj <0.05 and log2FC >1 or <-1. b-c, Normalized enrichment score (ZScore) of (b) active and (c) inactive chromatin signature specific to HSC-iM scored by chromVAR in the Xenograft model. d, Pearson correlation of the TF motif enrichment among active and inactive chromatin regions specific to HSC-iM. Only significantly enriched TF motifs (padj <0.05) were considered.

Specific questions:

9) Fig. 2 The authors should assess and document if the epigenetic remodeling triggered by inflammatory stimuli and the resulting epigenetic memory occurs primarily in enhancers or promoters or both?

We performed this annotation for each HSC-iM-specific DAR and compared the results against non-specific but accessible chromatin regions, that is, those that were not significantly enriched in either HSC-iM or HSC-I (Referee Fig. 19). We observed a subtle, but statistically significant enrichment of HSC-iM DARs within promoter regions (29.43% versus 26.34%, p=0.02), but no significant differences across other regions - introns or exons, 5’ or 3’ UTRs, or intergenic regions - were found.

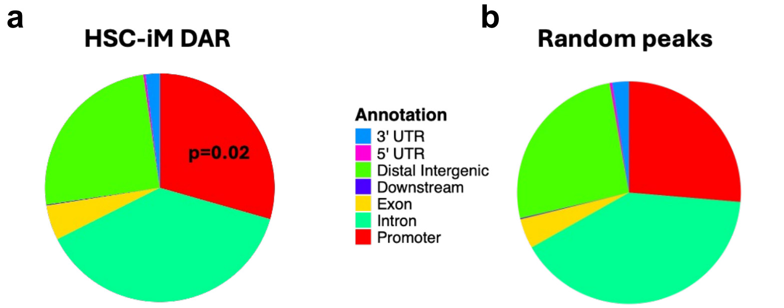

Referee Fig. 19: Genomic landscape of differentially accessible chromatin regions in HSC-iM. a-b, pie charts depicting the genomic annotation of (a) differentially accessible chromatin regions in HSC-iM and (b) set of random peaks (same number as in (a)) sampled form the total peaks in the xenograft model. An FDR-adjusted two-sample proportions test was used for overall comparison of the different populations.

Additionally, out of 187,329 chromatin peaks within our scMultiome dataset, we identified only 990 peaks which had statistically linkage to gene expression and can thus be considered cis-regulatory elements (CREs). These comprised 3.1% of HSC-iM-specific chromatin regions and 0.9% of non-specific chromatin regions (FDR-adjusted two sample proportions test, p=0.02).

Thus, epigenetic remodeling related to inflammatory memory shows a very mild, but statistically significant, preference for both promoters and enhancers.

10) Fig. 3. The sizes of the two HSPC populations don't look very different between PBS or TNF, but LPS treatment makes the inflammatory HSC population smaller rather than larger. What does this mean? They are all analyzed independently. If analyzed in the same UMAP, with or without Harmony reduction, do they cluster together , or separately? I.e. how similar are inflammatory HSCs caused by different stimuli?

When each condition is visualized on the same UMAP (i.e. the combined UMAP from Fig. 2b), the respective HSC-I and HSC-iM populations (as shown in Fig. 3) indeed overlap in the same clusters, demonstrating a large degree of similarity in the HSC-iM state across treatments (Referee Fig. 20a). This is further supported by examining the top HSC-iM-specific genes and eRegulons, which show consistent discriminatory power between HSC-iM and HSC-I within each treatment condition (Referee Fig. 20b-c). Together, these data show that the molecular features that underlie the HSC-iM state are relatively stable and similar amongst the treatment conditions/stimuli we have investigated.

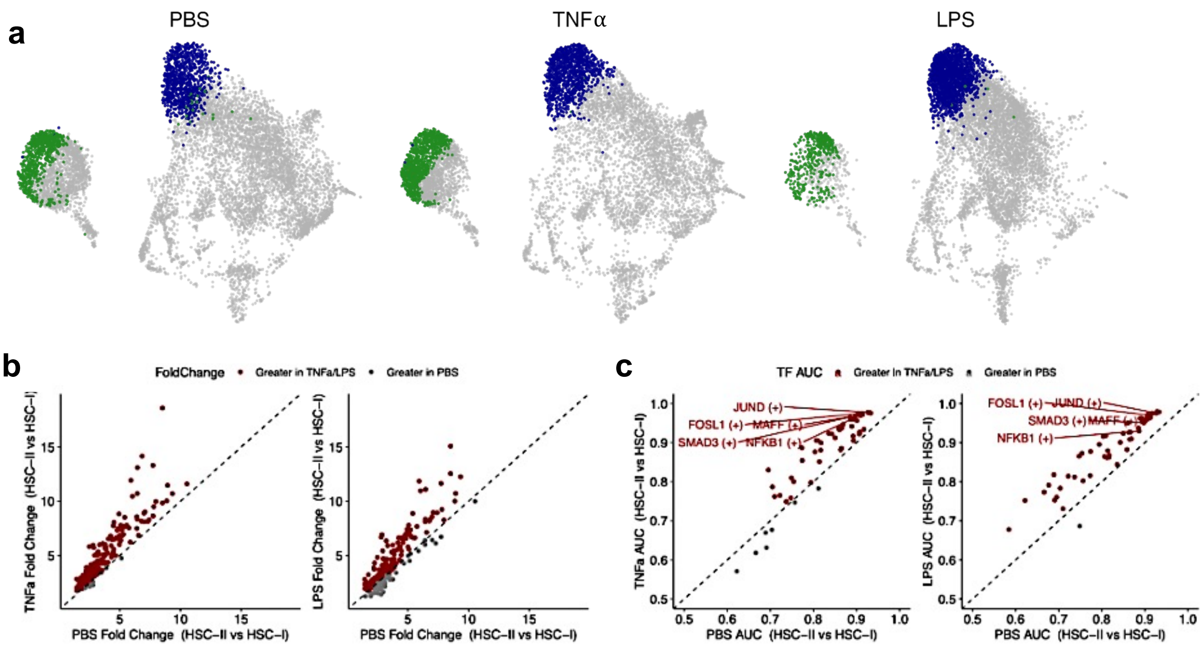

Referee Fig. 20: Conservation of the HSC-iM state across treatment conditions. a, Projection of HSPCs isolated from each of the PBS, TNFα and LPS conditions projected onto the combined scMultiome UMAP. b, Fold-change of top 200 HSC-II vs HSC-I marker genes, based on differential expression within PBS, hTNFα, and LPS conditions. Each point represents a gene, and points are colored based on whether the HSC-II vs HSC-I fold-change is greater in the hTNFα/LPS conditions (red) or greater in the PBS condition (grey). c, AUROC of HSC-II specific TF eRegulons (SCENIC+), evaluated within PBS, hTNFα, and LPS conditions. AUROC value depicted is the average of the chromatin-based AUROC and expression-based AUROC for each TF eRegulon.

With that said, there is indeed a lower number of HSC-iM relative to HSC-I within the LPS condition (18.6%) compared to the PBS (43.7%) and TNFα (47.3%) conditions. We do not know the reason for this and can only speculate based on our available data, and it may even include technical reasons. Furthermore, we showed in Fig. 3 of our main manuscript that there are indeed

differences in gene expression and in eRegulons, that is, transcription factor activity, *within* the HSC-iM population between PBS, TNFα, and LPS treatment conditions. Thus, some features exist in HSC-iM that are unique to different inflammatory stimuli.

11) Comparison of various iM HSC data sets. Can the Multiome data sets be analyzed together, rather than simply projecting signatures from one data sets to anotherr? Can the inflammatory HSCs from COVID or CH be compared directly to Multiome of the experimental model to identify similarities and unique differences based on exposure type or time of exposure?

We recognize the value of what the reviewer is proposing and have made considerable attempts to undertake an analysis that would integrate the COVID (peripheral HSPC), aging BM, fresh cord blood, and cells from the inflammation-recovery xenograft model. We attempted 2 approaches: creating a Symphony Reference Map; and co-clustering the HSCs from the various datasets by batch correcting using harmony and using a refined set of variable genes from HSC-iM vs HSC-I differential expression (DE) analysis. Please see our response to point 8(b). Unfortunately, it has not been possible to combine and directly compare the HSC-iM cells from each of the datasets together.

12) Fig. 4 i. What is the HSC aging meta signature of 37 genes?
As indicated in the methods/Fig. 4h, the aging metasignature was derived by intersecting the common differentially expressed genes across all the datasets presented in Fig. 4h and Extended Data Fig. 6h. The specific signature of 37 genes is listed in supplementary table 14, as indicated in the text at line 381. The list is also pasted below for convenience:

> NR4A2, NR4A1, PLK3, FOSB, ETV3, EHD4, JUNB, ANKMY1, PHLDB1, SERTAD1, ARL5B, ICAM1, NFATC1, ZFP36, ID1, PLAUR, FIGN, PTGS2, DUSP2, KLF4, MCL1, NR4A3, EGR1, PTPRM, AEN, NCEH1, MIDN, CD83, FOS, MLF1, AR, SPAG6, TNFAIP3, BHLHE40, CSRNP1, EMP1, JAG1

Referee #3 (Remarks to the Author):

Zeng and colleagues present a very interesting and provocative study that identifies a subset of human hematopoietic stem cells (HSCs) that retain a transcriptional memory of prior inflammatory challenge. It has been well described in mouse models that some HSCs undergo epigenetic and transcriptional changes in response to inflammatory stimuli that permanently alter their function and allow them to respond differently upon repeated inflammatory challenge. To this reviewers knowledge, the current manuscript presents the first evidence of such populations existing in humans, identified through rigorous single cell transcriptional and multi-omic profiling across a variety of experimental and physiological human systems.

The authors start by exerting various inflammatory stressors (LPS, TNFα) to mice xenografted with cord blood-derived HSCs. The functional consequences of prior inflammatory exposure on human HSCs in these models was relatively modest. However, scMultiome profiling of the human cells after the experimental treatments revealed two distinct clusters of HSCs (both defined by expression of many traditional HSC marker gene) – a more "classical" HSC cluster and a very distinct cluster of HSCs with upregulation of NFkB, AP-1, and other inflammation-activated signaling pathways. These are termed the inflammatory memory HSCs (HSC-iM). The single cell profiling is quite rigorous and convincing. The authors then extend these experimental findings into physiological human settings and are able to identify analogous HSC-iM populations during human aging, recovery from severe COVID-19 infection, and clonal hematopoiesis (CH).

Overall the manuscript is quite compelling and uncovers regulation of human HSC dynamics at unprecedented single cell resolution. The statistical analyses and data representations are appropriate. However, the major conclusions are somewhat over-reliant on the sequencing data interpretation and in some instances are not supported by orthogonal functional analyses. In this reviewers mind, there are two major questions presented this data that need additional clarification. First, it is unclear how the authors disentangle their HSC-iM signature from that of normal human aging HSCs. From mouse studies, we know that HSCs accrue inflammatory damage with aging and that inflammation may in fact play a large role in the functional decline in HSC potential with age. All the physiological conditions where the authors identify HSC-iM in this study (aging, CH, COVID-19 recovery) are associated with the elderly (I could not find ages for COVID-19 patients?). So the existence of this cell population in these conditions might just reflect a consequence of human aging rather than a history of inflammatory challenge (although the two conditions may be hard to deconvolute in real world settings). Secondly, what are the consequences of this **HSC-iM signature on functional output of the HSCs**? The consequences of the HSC-iM signature are interpreted from transmission of this transcriptional program to downstream progenitor cells and monocytes. There is a lack of direct functional

examination of the impact of this gene expression program on the HSC-iM HSCs in vivo. If these points could be clarified this would represent a very impactful study.

We thank the Referee for finding our study to be rigorous, compelling, and provocative as the first known description of epigenetic and transcriptional memory of inflammation in human HSCs. We agree that the additional interrogation of HSC-iM in physiological conditions beyond those primarily associated with the elderly was warranted. We have included new analyses of HSCs from pediatric patients with sickle cell disease (New Fig. 4i and Extended Data Fig. 6k, l). We have also generated new data showing HSC-iM signature strength predicts all-cause mortality risk in peripheral blood cell single-cell transcriptomes from individuals between 30 to 75 years of age (New Fig. 6i-k). See response to Referee #1, point 1 and Referee Fig. 1 & 3.

To extend the evaluation of the functional outcomes of HSC-I and HSC-iM in the xenograft setting from our original submission, we have now performed scRNAseq of CD34+CD38+ progenitors, to accompany the existing CD34+CD38-CD45RA- HSC/MPP-enriched scMultiome and CD33+ myeloid scRNAseq (Referee Fig. 21a). We classified cell types from this CD34+CD38+ dataset by mapping onto BoneMarrowMap (Referee Fig. 21b). We inferred clonal relationships between all cells subjected to scRNAseq/Multiome across the three datasets (similar to how we had done in our submitted manuscript). We identified 15 genetic clades as before (Referee Fig. 21c), and each clade had cells from all three treatment conditions (PBS, TNFα or LPS; Referee Fig. 21d). Next, we evaluated progeny from HSC-I or HSC-iM-biased genetic clades across all identified cell types from the three datasets, and found enrichment of the HSC-iM signature in B-lymphoid cell types as well as myeloid lineage cells derived from HSC-iM dominant clades (Referee Fig. 21e, f).

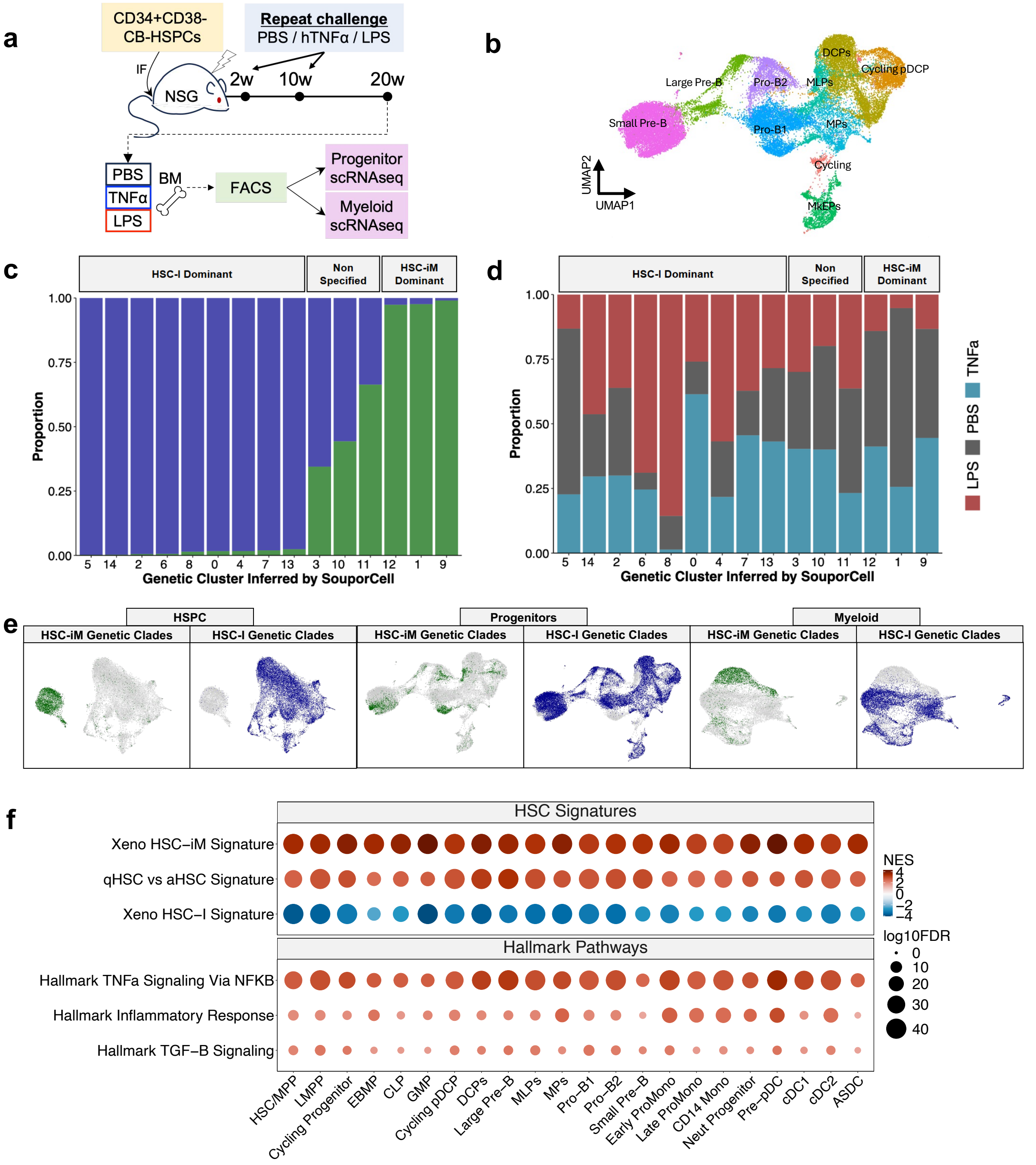

Referee Fig. 21. a, Schematic depicting sorting of committed progenitor and myeloid compartments from the xenograft model for scRNA-seq. b, UMAP of CD34+CD38+ progenitor cells. c, Bar plot depicting the proportion of HSC-IM and HSC-I in each clonal group. d, Bar plot depicting the proportion of cells derived from PBS-, TNFα-, or LPS-treated mice in each clonal group. e, UMAPs of HSPC, progenitor, and myeloid cells depicting distribution of HSC-iM-specific and HSC-I-specific genetic clades. f, GSEA results depicting enrichment of HSC and hallmark signatures in differential expression results between HSC-iM-specific genetic clades vs HSC-I-specific genetic clades within HSPC, downstream progenitors, and myeloid populations. Positive enrichment scores (red) indicate enrichment in HSC-iM-specific genetic clades.

We also evaluated the proportion of cell types and differentiation trajectories from HSC-I-dominant vs HSC-iM-dominant clades from the xenograft model. We found a significantly increased abundance of HSC and CD14 Monocytes within HSC-iM-biased clades (Referee Fig.

22a) as presented in the projections of HSC-I (downsized to match HSC-iM cell numbers) and HSC-iM derived progeny onto BoneMarrowMap as one illustration of this finding for the Referee. By computing pseudotime, we noticed different trajectories of differentiation between HSC-I- and HSC-iM-biased clades (Referee Fig. 22b). The relative proportions and statistical comparisons for each cell type across the three datasets are shown (Referee Fig. 22c-f). These data further confirm that HSC-iM shows a limited differentiation output with relative monocytic skewing in the CD33+ immunophenotypic progeny. However, all lympho-myeloid progeny from HSC-iM show enrichment of an inflammatory state. Data from Referee Fig. 21 and 22 have been added into Fig. 6 and Extended Data Fig. 9 of the revised manuscript.

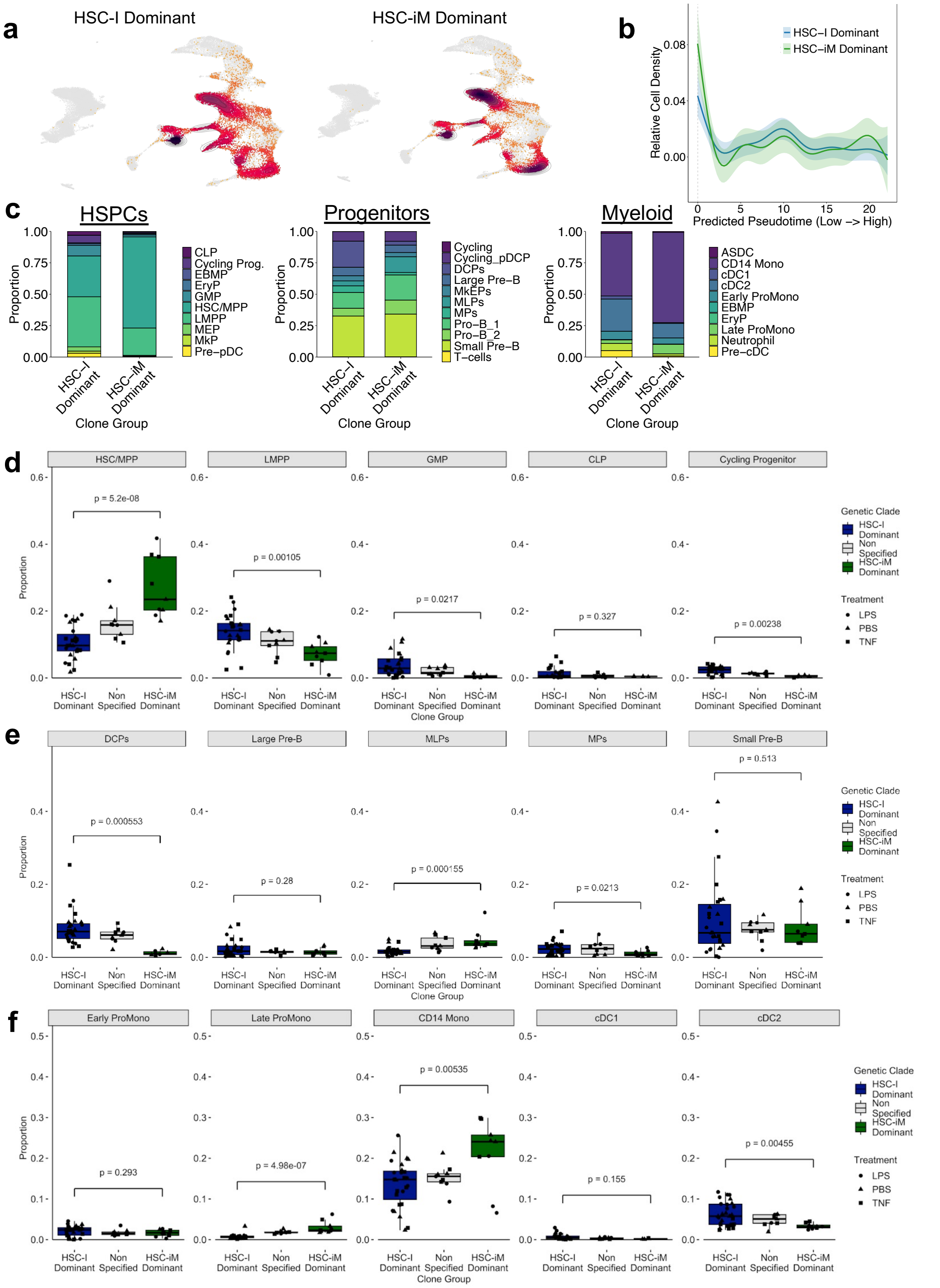

Referee Fig. 22: a, Density plot of HSC-I and HSC-iM derived progeny mapped onto BoneMarrowMap. HSC-I has been downsized to match HSC-iM cell numbers (n=10,719 single cells). b, Predicted pseudotime within HSC-I and HSC-iM derived progeny shown in (a). c, Proportions of cell types for HSC-iM-dominant, HSC-I-dominant, and non-specific genetic clades within the CD34+CD38-CD45RA- HSPC, CD34+CD38+ Progenitor, and CD33+ Myeloid datasets. e-f, Proportion of each indicated cell type in HSC-I-dominant, non-specific or HSC-iM-dominant clades as derived from the HSPC (d), progenitor (e) or (f) myeloid datasets isolated from 20w xenograft inflammation-recovery recipients.

MAJOR POINTS:

• All the conditions where the HSC-iM signature is identified in human cell populations are associated with aging (elderly people, CH, COVID-19 recovery). To truly prove that this HSC-iM signature and these cells exist as a consequence of prior inflammatory stress, the authors should examine some pediatric populations to see if such HSC-iM exists in direct response to inflammatory challenge. There are several potential pediatric populations that could be studied – recovery from severe infectious disease (as done for the adult COVID-19 patients), autoimmune disease, or after vaccinations. If blood/BM could be sampled from these children and compared to "healthy" age-matched control samples to show enrichment of the HSC-IM signature and existence of this distinct HSC population in younger patients it would almost completely alleviate the concern that this signature overlaps strongly with the HSC aging signature.
As requested by the Referee, we have examined HSC-iM in sickle cell disease patients and shown enrichment in this blood disorder relative to age-matched controls (see Referee Fig. 1, Referee #1, point 1). Additionally, to further emphasize that we can dissociate age from consequences of prior inflammatory stress, we note to the Referee that the severe COVID recovery analysis was age-matched. Finally, enrichment of HSC-iM within aged vs young adult-derived HSCs is ranked 47th in >7000 gene sets as shown in Extended Fig. 6g, unlike in COVID-recovery or CH, suggesting HSC-iM is not merely a reflection of the aging process in HSCs.

• The authors have very convincingly described the existence of this distinct HSC-iM population of HSCs by scMultiome profiling. But what is the functional and physiological significance of this molecular signature in terms of the output of these HSCs and their response to repeated inflammatory challenge? The authors need to complement their molecular profiling with some in vivo functional assays.
We recognize that definitive functional studies require prospective isolation and/or lineage tracing; something that is currently not possible as described in response to Referee #1, point 6 and Referee #2, point 1. However, we have strengthened our data on the functional and physiological significance of the HSC-iM state by additional investigations into lineage output and connecting it to health consequences. Specifically, we have extended our analysis of differentiation trajectories in the xenograft setting to show that HSC-iM have a bias towards monocytic lineage output (Referee Fig. 21/22, Extended Data Fig. 9 of the revised MS). We have already shown that the HSC-iM state exists in many real-world settings associated with inflammation including COVID19 (Fig. 4a-e) and SCD (Referee Fig. 1, Fig. 4i of revised MS). We now show in an analysis of a large population cohort that enrichment of the HSC-iM signature in circulating lympho-myeloid cells is highly predictive of all-cause mortality (Referee Fig. 3 and response to Referee#1, point 1, Fig. 6i-k of revised MS). Indeed, absent a formal clinical trial, we would argue that the latter study is the ultimate *in vivo* functional study done in humans where the read out of the assay is being dead or alive. Finally, we also find that the

transmission of the HSC-iM program to myeloid progeny is not dependent on whether an individual has clonal hematopoiesis, at least when examining the xenotransplanted subset of adult donors where we knew the proportion of HSC1/HSC-I vs HSC2/HSC-iM and had enough material to perform the experiment (see Referee Fig. 6 and 8 and response to Referee #1 points 2 and 4).

Can the authors show there is a different lineage output from HSC-iM HSCs versus traditional HSCs in response to repeated inflammatory challenge?

We created a modified xenograft challenge model employing NSG-SGM3 immunodeficient mice and performed xenotransplantation of five adult BM samples, where the HSC-I/HSC-iM composition was known to address this question (see Referee Fig. 6). We assessed engraftment outcome with a single CB donor in this mouse model to bridge our findings from inflammatory challenge in the NSG and NSG-W41 models (Referee Fig. 23a). Proliferative output from adult samples are known to be reduced relative to CB, which we see here (Referee Fig. 23b). Interestingly, TNFα treatment significantly increased total graft size in NOC108, a *DNMT3A*-mutant sample that is mixed for HSC-I/HSC-iM, but not in any of the other samples (Referee Fig. 23b). NOC108 is the first example that we are aware of an increased graft from a primary *DNMT3A* mutation-containing sample following inflammatory treatment. TNFα treatment reduced the monocytic output of HSC-I-derived progeny in the two HSC-I-dominant xenografts, whereas it increased the monocytic output of HSC-iM-derived progeny in the other three xenografts (Referee Fig. 23c). Unfortunately, too few mutant cells were detected to enable an analysis of clone size in these xenograft models. Collectively, these data, shown in Extended Data Fig. 8, validate our observation that HSC-iM generate fewer myeloid progeny than HSC-I, and suggest the two HSC subsets respond differently to inflammation.

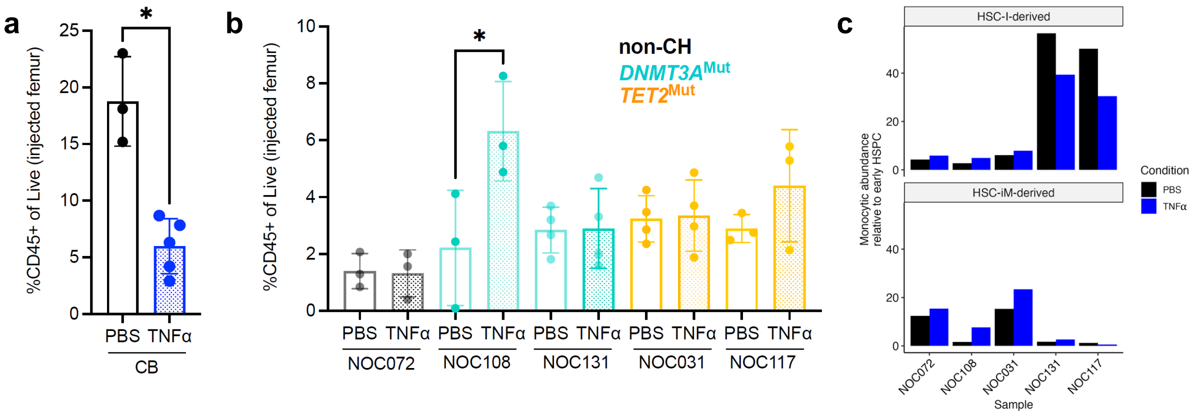

Referee Fig. 23: Hematopoietic output from human bone marrow HSPCs in an inflammation-recovery model. a, Human engraftment from a single CB donor (80,000 CD34+ cells injected/mouse) is decreased following TNFα repeated challenge compared to PBS controls at 12w post-transplant. b, Human engraftment at 12w post-transplant of CD34+ HSPCs from bone marrow of 5 individuals with or without CH mutations where the HSC-I and HSC-iM composition is known (NOC072: 77,500 cells/mouse; NOC108: 55,800 cells/mouse; NOC131: 90,600 cells/mouse; NOC031: 80,600 cells/mouse; NOC117: 55,800 cells/mouse). c, Abundance of monocytic cells (Late GMP, ProMono and Monocytes) relative to early HSPC populations among HSC-I vs HSC-iM-derived cells in the xenograft BM samples treated with either PBS or TNFα.

Is there a difference in self-renewal of the HSC-iM cells during infection? Do they respond better/faster than normal HSCs during repeated inflammatory stimulation? Can the authors use their scMultiome data to identify cell surface markers to separate HSC-iM HSCs versus other HSCs after inflammatory challenge via LPS, TNFα etc.. and then perform serial transplant to observe engraftment and self-renewal kinetics?

Fig. 1 of the original submission shows secondary limiting dilution transplantation assay data on CB following TNFα or LPS treatment. However, we cannot formally determine self-renewal of HSC-I and HSC-iM separately as we do not have surface markers to prospectively isolate the two subsets, despite extensive forays to find immunophenotypic markers (see Referee Fig. 11 and 12 and response to Referee #2 point 1). We will continue to evaluate novel technologies and additional markers to continue to strive towards this goal, but hope the Referee can appreciate the difficulty of such a task and why this aim is outside the scope of this study.

If not a cell surface marker can a barcoding approach be used? This would be preferred but even in vitro CFU assays would be a helpful start. Is there a difference in cytokine output of the HSC-iM HSCs?

Unfortunately, lentiviral barcoding is currently not an option (see Referee Fig. 7 & 9 and response to Referee #1 point 6).

As the Referee suggested, we have begun to interrogate whether HSC-iM impacts functional output with *in vitro* colony assays. We isolated a limited number of LT-HSC from the bone marrow of the five individuals from our CH cohort that we xenografted in Referee Fig. 6 where we had prior knowledge of the HSC1/HSC-I vs HSC2/HSC-iM composition (Referee Fig. 24a). CB samples have increased cloning efficiency with TNFα treatment compared to controls (Referee Fig. 24b). Adult samples have overall decreased cloning efficiency compared to the CB samples. We recognize this is a limited sample set with multiple confounding elements including CH and HSC status. But, we observed that those samples classified as HSC-iM (NOC117) or mixed (NOC072 and NOC108) show decreased clonogenic output relative to those classified as HSC-I (NOC031 and NOC131).

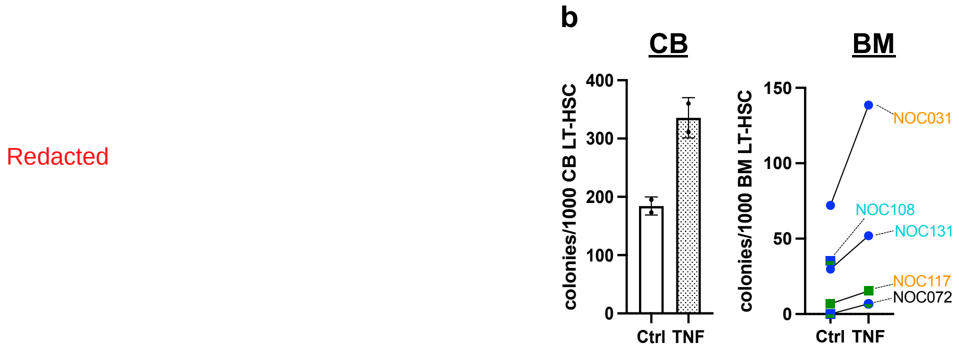

Referee Fig. 24: Colony forming cell assays of LT-HSC from human cord blood and bone marrow donors following TNFα treatment. a, Experimental schematic. b, Cloning efficiency is increased in CB LT-HSC following TNFα treatment (n=2) c, Cloning efficiency of 5 bone marrow donors with HSC1/HSC-I (blue, NOC031 and NOC131), HSC2/HSC-iM (green, NOC117) or mixed (NOC072 and NOC108) composition. Note: there were too few LT-HSC for NOC108 for the TNFα-treated condition.

In the absence of prospective isolation it is not definitively possible to link cytokine output with HSC subsets. One way to approach this in a future study would be to acquire additional samples such as those used in Referee Fig. 6 and perform transplantation studies with more HSPCs to generate larger human grafts, then perform cytokine profiling. In lieu, we have evaluated levels of 23 human and 18 mouse cytokines in plasma of mice challenged with LPS to establish the cytokine landscape in our xenograft model in response to acute inflammation (see Referee Fig. 15 and response to Referee #2 point 7). We have also evaluated the levels of these same cytokines in banked plasma from the mice that had recovered from repeat challenge, i.e. a subset of the same mice from which the HSC-iM population was originally identified (Referee Fig. 25, Extended data Fig. 4h in the revised MS). However, we note that the scMultiome was obtained from HSPC isolated from many recipients that were pooled. Hence, it is impossible to determine HSC-I vs HSC-iM classification on a per mouse basis and subsequently link it to cytokine output. Nonetheless, we found sustained and significantly elevated levels of numerous cytokines, including IL8, TNFα and IL6, in mice that had recovered from repeated TNFα challenge, while more variable levels were found in LPS challenged mice.

Of note, we found reduced IL3 levels after inflammatory challenge that correlated with the reduction in engraftment levels. IL3 is a cytokine that helps maintain hematopoietic output; this finding therefore provides one avenue for further exploration to determine the mechanism for the sustained reduction in graft size but with an unaffected stem cell compartment (as determined by secondary transplant shown in Fig. 1p-s).

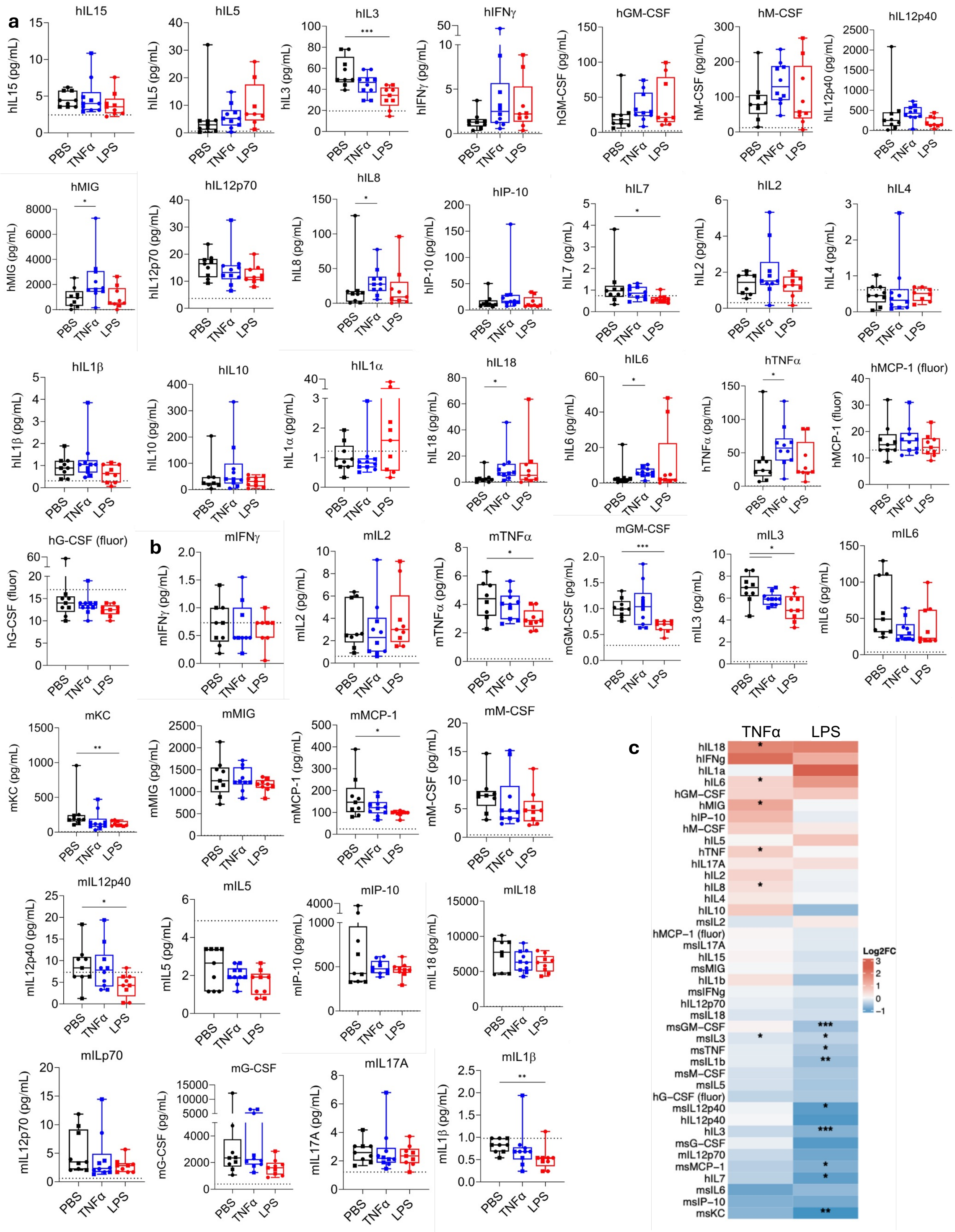

Referee Fig. 25: Multiplex cytokine profiling of blood plasma at 20 weeks xenotransplantation. Blood plasma from xenografted mice (n=2 CB pools, 4-5 mice/group) was sampled after repeated challenge with PBS, TNFα or LPS for human (n=23) and b, mouse (18) cytokines. The dashed horizontal line shows the lower detection limit. c, Heatmap of all data relative to PBS control. Pairwise Mann-Whitney tests *** p<0.001, ** p<0.01, * p<0.05

• Figure 1g-j – this seems like a very significant drop in human cell engraftment in response to an acute (16-hour) inflammatory challenge. What is the fate of the missing cells? Are they undergoing apoptosis? Mobilization from the BM?

We have looked in the peritoneum, spleen, and cranium, where we consistently observe a depletion (as opposed to specific enrichment). We have also evaluated peripheral blood where we did not observe many human cells in our model regardless of LPS treatment. We always include propidium iodide or sytox in our flow cytometry to assess viability, and as shown in Referee Fig. 26, we do not see major changes in the proportion of live cells (a,e), although reduced cellularity is seen upon treatment (b,f). Moreover, we also included an anti-mouse CD45 antibody in this experiment and showed concomitant depletion of mouse cells, as expected from such treatment (d,h). It is possible we are sampling the bone marrow at a time point too late to observe direct effects of the treatment. Thus, at the present time, we do not know the fate of the human cells.

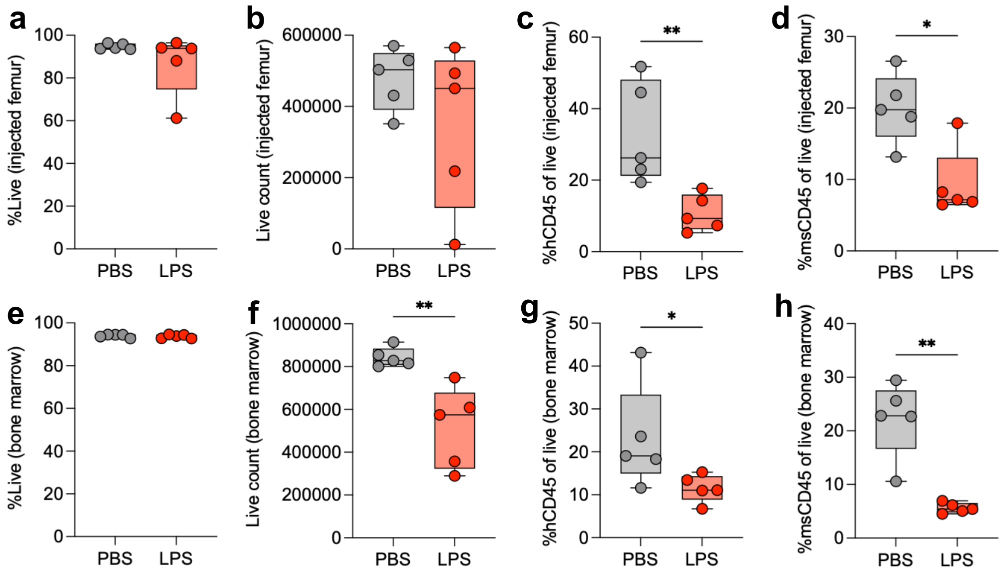

Referee Fig. 26: Human and mouse CD45 composition after acute inflammatory challenge. NSG mice transplanted with CB CD34+CD38- cells (n=5 per group) were treated with PBS control or LPS at 6 weeks post-transplantation and analyzed for a, %live cells, b, live cell count c, %human CD45 or d, %mouse CD45 in the injected femur and e, %live cells, f, live cell count g, %human CD45 and h, %mouse CD45 in the non-injected bones (bone marrow). The cytokine profiling of blood plasma and bone marrow from these mice are shown in Referee Fig. 15 and 25.

The sharp drop in human cell engraftment over a short period is inconsistent with the statement on line 160-161 “these findings demonstrate a negative impact of acute inflammatory stimuli on human hematopoietic output in a xenograft model”. It is likely not a defect of the stem cell differentiation output during this 16-hour period, but rather a negative impact on survival of the mature BM cells. Please clarify.

We agree with the Referee that our statement was misleading, and that the observed reduction of graft size is likely a result of depletion of mature BM cells. We have revised the text at line 183-184.

Also, it would be better to represent these data as percentage change in engraftment for each mouse pre- and post-exposure. Due to variability in engraftment between mice, perhaps the drop comes from selection of mice that were unknowingly at a lower starting BM engraftment. Without knowing the pre-exposure engraftment level for each mouse it is hard to justify this claim.

While it is theoretically possible to measure the baseline engraftment level in mice pre-challenge, this intervention would require a bone marrow aspirate and would likely introduce a confounding variable to our experimental model. Engraftment is typically not observed at a significant level in peripheral blood of xenografted NSG transplanted by intrafemoral injection; we confirmed this in our model, including in some of the mice presented in this study (graft size <0.01% hCD45/GlyA). Further, the Dick lab established the original version of this xenograft model to study human hematopoiesis, and many prior studies have used the same transplantation method. It results in consistent graft sizes from a known injected cell dose. Note that the engraftment data has been presented for each mouse and has not been normalized across experiments where different CB pools were used as denoted by use of different symbols. The variability is therefore transparently shown, but is not restricted by group. In addition, we have performed numerous follow-up experiments while investigating interventions/new markers, including an experiment specifically for the revision of this paper, where we found a similar effect of LPS (see Referee Fig. 26). As such, we maintain our claim that the graft has reduced in size due to the inflammatory challenge.

MINOR POINTS:

• Figure 1: Some of the xenograft data is hard to interpret (e.g %CD34 of CD45). Please show overall frequency and absolute numbers of these cell populations per mouse for more transparency. And it would be better to have quantification of more pure HSC populations (as indicated on line 206) rather than just overall CD34+.

We are uncertain as to what aspect of which figure is hard to interpret. We are also mindful of the restrictions that *Nature* imposes on us in terms of figure panels and size, as for example we cannot add extra panels to Extended Data Fig. 2 which shows the engraftment data. We will share files that will include all raw engraftment data upon acceptance.

The proportion of CD34+ cells within engrafted mice is limited. Thus, we are not able to undertake analysis of pure LT-HSC (Lin-CD34+CD38-CD45RA-CD90+CD49f+) at an individual mouse level, only in pools.

• Line 398-399: "Taken together, our results demonstrate the molecular programs underlying the HSC-iM state accumulate in human HSCs with age". The alternative explanation is that aging HSCs are just reflective of a lifetime accumulation of inflammatory insults.

We agree. As we also observed enrichment of HSC-iM in sickle cell disease, which has been associated with premature hematopoietic aging due to chronic inflammation from the disease manifestation, we have revised the summary sentence in that section to "Taken together, our results demonstrate that a lifetime of inflammatory insults expected to occur with aging as well as chronic inflammation arising from SCD result in accumulation of HSC-iM."

• The section "CH mutations preferentially impact HSC-iM to promote differentiation" is not really supportive of the overall message of the manuscript. Because it seems like different platforms were used for the analysis there is no direct readout of HSC-iM in this data. Line 483 – "predominantly within the HSC2 state (which is equivalent to HSC-iM). And again, the impact on differentiation is inferred rather than a direct functional readout.

We have rewritten this section and clarified the wording. We present unequivocal evidence that the populations formerly identified as HSC1 and HSC2[20] are phenocopies of HSC-I and HSC-iM which we first identified experimentally using our xenograft challenge-recovery model. Thus, we can make the key claim that humans possess HSC-iM and that this population is not simply an artifact of xenografting. TARGETseq+ is a high performing scRNA-seq platform that captures an even higher number of expressed genes per cell than a standard 10x scMultiome or scRNA-seq with the added benefit of also capturing immunophenotypic and genotyping information. The fact that these two platforms arrive at the same conclusion is an advantage, not a liability.

Also, we disagree about the inference comment. The HSC-I vs HSC-iM annotation is directly determined from the respective signature expression on a per HSC cell basis and not inferred. Thus, both in our original data and also with the new data on xenografts derived from the CH samples, we can directly examine the functional cellular output of either HSC-I or HSC-iM and present strong evidence that there is reduced myeloid maturation of HSC-iM compared to to HSC-I, that the HSC-iM signatures are transmitted to the entirety of the myelo-lymphoid progenitor and mature cell compartments we examined, and that the presence of the CH mutation relieves this maturation block. This new evidence raises an important hypothesis for the field in terms of how CH mutations might become selected for. These are all discussed in our revised manuscript.

Referee #4 (Remarks to the Author):

In the manuscript entitled "Identification of Human Hematopoietic Stem Cell Subset That Retains Memory of Inflammatory Stress," Zeng and colleagues investigate the impact of inflammatory stimuli, specifically TNF-α and LPS, on hematopoietic stem cells (HSCs) and their functional outcomes. Utilizing a preclinical xenograft inflammatory recovery model, as well as analyzing HSCs from individuals with severe COVID-19, aged individuals, and those with clonal hematopoiesis, the authors identify a subset of HSCs with inflammatory memory (HSC-iM). These cells retain inflammatory reprogramming, which can be transmitted to their progeny—particularly innate immune cells—in a cell-autonomous manner. Notably, the authors demonstrate that the programming of HSC-iM shares similarities with the mechanisms underlying memory T cell formation. While this study is significant and employs elegant experimental approaches to address key hypotheses, there are several conceptual gaps that require further scrutiny.

1. It is unclear how intraperitoneal administration of LPS or TNF leads to the reprogramming of HSCs and the generation of HSC-iM. Is this a direct effect of LPS or TNF on HSCs in the bone marrow (BM), or is it mediated indirectly through systemic factors? Can the authors measure the levels of TNF and LPS in the BM of the injected CD34+ cells to clarify this mechanism? Previous studies have shown that systemic type I IFN or IL-1 signaling is required for HSC reprogramming following intraperitoneal administration of β-glucan. Additionally, the memory programming of HSCs in response to BCG versus Mtb infection has been attributed to type II IFN and type I IFN, respectively. Does the generation of HSC-iM depend on TLR4 or TNFR signaling pathways? Addressing these points could provide critical insights into the different mechanisms underlying HSC reprogramming.
To investigate what systemic effects would be elicited by an acute LPS challenge via intraperitoneal injection, we performed a new xenograft experiment and sampled blood plasma before and after acute LPS challenge at 6w post-transplantation (see response to Referee#2 point 7 and data in Referee Fig. 15). We also sampled BM supernatant in the same experiment; BM was flushed from injected or non-injected bones, and cells were removed by centrifugation to obtain diluted BM plasma (see results in Referee Fig. 27 & 28).

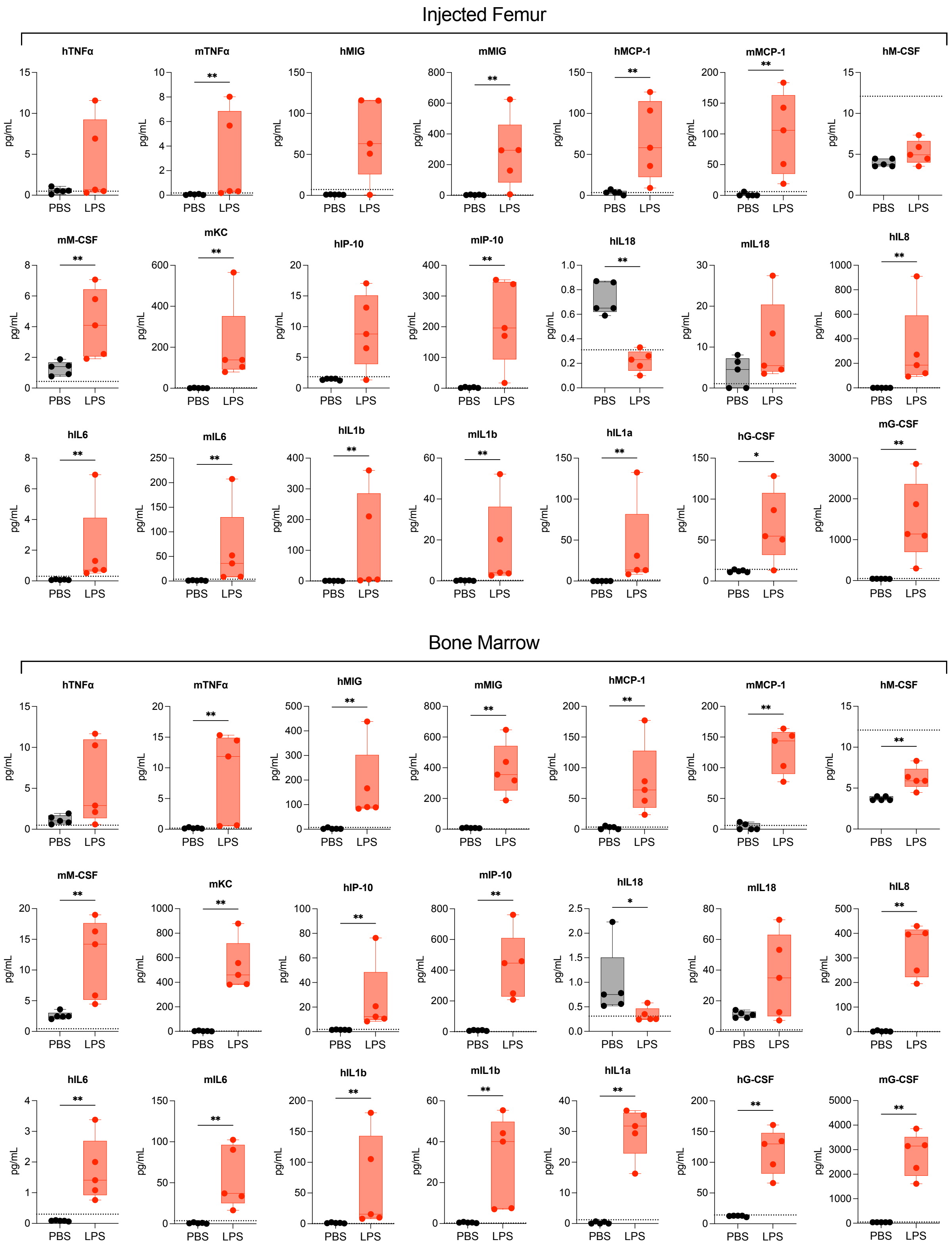

Referee Fig. 27: Multiplex cytokine profiling in the inject femur and non-injected bone marrow at 6 weeks post-transplantation. The human and mouse cytokines detected above baseline are shown here and are complementary to the blood plasma cytokine profiling in Referee Fig. 15. Mann-Whitney tests. ** p<0.01, * p<0.05

An elevated level of hIL1β in plasma was found. We were unable to measure type I IFN, but did find elevated levels of IFNγ. In bone marrow plasma we also found variable but significantly elevated hIL1β and hIL1α; IFNγ was below detection level. Thus, as expected in a human, our model has the potential to elicit a multifaceted inflammatory response that probably results in a complex array of signaling cascades that ultimately result in HSC memory. We and others are actively working to resolve the exact mechanism by which HSC memory forms in ongoing studies that will form part of future manuscripts.

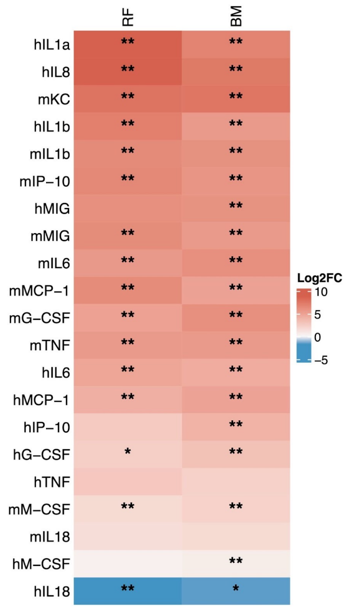

Referee Fig. 28: Heatmap depicting the relative levels of human and mouse cytokines detected in the injected femur (RF) and bone marrow (BM) at 6 weeks post-transplantation after LPS treatment when compared to PBS controls. paired-ratio t tests. *** p<0.001, ** p<0.01, * p<0.05

To effectively evaluate whether TLR4 and/or TNFR within the human graft and specifically in the HSC compartment is required for the observed effects, we would need to genetically manipulate the graft, and this would introduce a confounding factor with respect to prior inflammatory exposure of the HSC; and the conditions for genetic manipulation require *in vitro* culture, another confounder. Similarly, we would need to determine the correct dosage and delivery methods for any chemical or antibody-mediated interventions and ensure they don't affect our experimental model.

Finally, in our unpublished work, we have evidence that human LT-HSC are exquisitely sensitive to TNFα. Specifically, we cultured purified LT-HSC in the presence of TNFα and found that a response marker we have discovered, CD83, is most upregulated at 16h in LT-HSC when compared to ST-HSC or mature progenitors. scRNAseq of TNFα-treated LT-HSC shows profound changes in transcriptional profiles at 16h after challenge, many of which were validated at the protein level by low-input mass spectrometry. The response to TNFα treatment includes expression of IL1β and EBI3 by the LT-HSC themselves. We also sorted CD34+CD38-CD45RA- stem-enriched cells from xenografts challenged with acute LPS and performed bulk RNAseq. Here we found an elevated level of a murine-derived HSC-specific signature of TNFα response after LPS challenge. Similarly, we find enrichment of unbiased NMF-derived gene expression signatures from *in vitro* TNFα-challenged LT-HSC in LPS-challenged xenografted mice. This data suggests that TNFα can have a direct effect on primitive human HSCs and is likely involved, at least in part, in the formation of HSC-iM. This data is being prepared for publication but has been presented at numerous conferences [see https://doi.org/10.1182/blood-2023-187737 and https://doi.org/10.1016/j.exphem.2024.104524].

2. The data from the COVID-19 study are intriguing and further support the central hypothesis of the current work. However, it is important to clearly define the ICU-control group.

Were there any septic patients included among the ICU controls? If so, it would be expected that the HSC-iM profile induced by LPS or TNF would be more similar in these septic patients compared to the COVID-19 patients.

We provide Referee Table 1 that summarizes the demographic information for this group; the table is derived from Table S1 of the original Cheong et al (2023) manuscript[14]. As can be seen in the table, all of the 4 COVID patients had sepsis as did 3 of 6 ICU control cases. We therefore split the ICU control cohort to evaluate whether sepsis would independently result in enrichment of the HSC-iM signature. As seen in Referee Fig. 29, the minor but statistically significant HSC-iM enrichment related to sepsis within the ICU control group is far outweighed by the enrichment related to COVID19, shown here comparing only the 4 COVID19 patients with the 3 ICU controls with sepsis. These data do not support the notion that HSC-iM enrichment is stronger following sepsis compared to severe COVID19 infection, and further research is required to evaluate and compare cytokine responses in severe COVID19 versus sepsis[28,29].

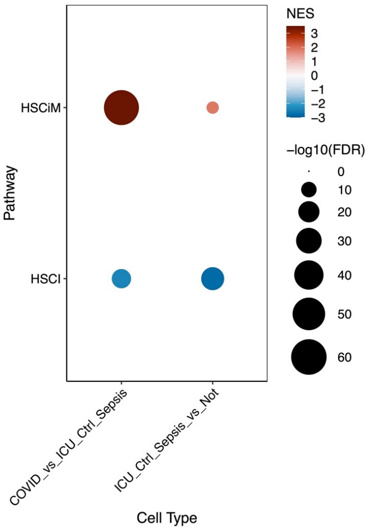

Referee Fig. 29: Dot plot summarizing GSEA enrichment of the HSC-iM or HSC-I transcriptional signatures in the ICU_control group from Cheong et al (2023) when septic vs non-septic patients are compared, and in patients recovering from COVID19 compared to only ICU-admitted patients with sepsis.

No.	Sample Name	Status	Age	Sex	BMI	Admission	Comorbidities	Days post admission	Sepsis (Y/N)	Clinical Notes
1	HDa	Healthy	36	F	19.5		none			
2	HDj	Healthy	40	M	29.7		intermittent asthma, environmental allergies			
3	HDv	Healthy	65	F	24.5		none			
4	HD_AD	Healthy	68	M	27.7		HTN, CAD (s/p stent), vitiligo			
5	Hdu	Healthy	69	M	33.1		Asthma, DM			
6	lgtd17	Healthy	40	M	21.4		None			
7	lgtd18	Healthy	40	M	21.4		None			
9	jcov115	nonCoV	64	F	26.32	Chronic liver disease and cirrhosis; encephalopathy and hydrothorax	HTN, HLD, hypothyroidism, alcoholic cirrhosis	32	Y	admitted with chronic liver disease and cirrhosis. Hospital course was complicated by encephalopathy thought due to liver failure and hepatic hydrothorax requiring chest tube placement. The patient had likely sepsis due to fungemia (*c. parapsilosis*) and a bacterial urinary tract infection. Total length of hospital stay = 48 days.
10	jcov119	nonCoV	65	F	25.24	Respiratory failure (cardiovascular-related)	Dermatomyositis, Afib	32	N	admitted with acute hypoxic respiratory failure due to pulmonary edema. Found to have severe mitral regurgitation and underwent surgical valve replacement. Hospital course was complicated by recurrent pleural effusion and postpericardiotomy syndrome. The patient did not have sepsis. Total hospital length of stay = 55 days.
11	JCov114***	Other	32	M	24.39	Hematemesis	Alcoholic Hepatitis, Portal HTN, Esophageal varices	3	N	admitted with hematemesis in the setting of acute alcoholic hepatitis. The patient did not have sepsis. Total length of hospital stay = 7 days.
12	jcov122	nonCoV	68	M	34.72	Renal mass and failure	HTN, HLD, CKD, renal mass s/p nephrectomy	27	N	admitted with renal mass and renal failure. Hospital course was complicated by hypotension in setting of postoperative bleeding and persistent atrial fibrillation with rapid ventricular response. The patient did not have sepsis. Total hospital length of stay = 29 days.
13	jcov117	nonCoV	73	F	33.1	Cholangitis	Hypothyroidism, obesity, breast cancer s/p chemo, RT	5	Y	admitted with cholangitis. Hospital course was complicated by septic shock from polymicrobial bacteremia secondary to ascending cholangitis, pulmonary edema, renal injury, and mild pancreatitis. Total length of hospital stay = 6 days.
14	jcov124	nonCoV	68	F	20.25	Sepsis (muscle)	HTN	6	Y	admitted with sepsis due to Iliopsoas muscle *S. intermedius* abscess and septic arthritis. The patient's hospital course was complicated by volume overload and atrial fibrillation. Total hospital length of stay = 11 days.
21	jcov52	Early	68	F	21.7		HTN	50	Y	admitted for acute hypoxic respiratory failure due to COVID-19 pneumonia. The patient was septic during the hospital stay due to multiple episodes of pneumonia with identified organisms by respiratory culture.
22	jcov21	Early	65	M	23.7		HTN, HLD, DM2	36	Y	admitted for acute hypoxic respiratory failure secondary to COVID-19 pneumonia. There was evidence of sepsis early in the patient's hospital course as evidenced by multiorgan dysfunction (altered mental status, renal failure, respiratory failure).
24	jcov42	Early	37	M	29		none	26	Y	admitted for acute hypoxic respiratory failure due to COVID-19 pneumonia. There was evidence of sepsis during the patients ICU course due to multiple episodes of bacterial pneumonia with identified organisms by respiratory culture as well as bacteremia thought to be due to blood stream infection from indwelling central catheters and/or pneumonia.
25	jcov69-8	Early	43	M			None	111	Y	admitted for acute hypoxic respiratory failure due to COVID-19 pneumonia. There was evidence of sepsis during the patients ICU course due to multiple episodes of bacterial pneumonia with identified organisms by respiratory culture as well as bacteremia thought to be due to blood stream infection from indwelling central catheters and/or pneumonia.

Referee Table 1. Summary of donor information from Cheong et al.

Since SARS-CoV-2 is a viral infection that primarily engages type I IFN responses, these data suggest that type I IFN in the bone marrow might play a crucial role in the LPS or TNF inflammatory xenograft model for generating HSC-iM.

LPS elicits a broad human inflammatory response in xenografts with an upregulation of 19 of the 23 human cytokines we measured (Referee Fig. 15). We cannot comment on IFNα and IFNβ levels in the context of the xenograft as they were not among the human cytokines in the

multiplex cytokine panel. We present for the Referee data from human LT-HSC treated *ex vivo* with two IFNs alongside TNFα and IL1β for a separate study investigating CD83 in human LT-HSC (Referee Fig. 30). We have found CD83 to be an exquisitely sensitive surface biomarker on human LT-HSC for acute inflammatory stress *in vitro* and *in vivo*, especially in response to TNFα, as summarized in point 1.

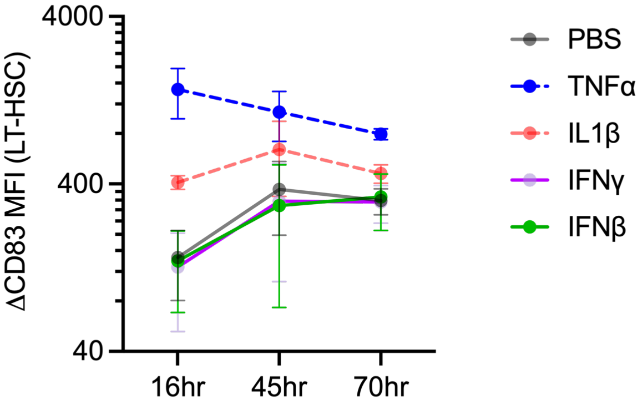

Referee Fig. 30: CD83 surface expression for human LT-HSC by flow cytometry after culture with indicated cytokines.

Redacted . Thus, while we have not specifically investigated interferons in the context of HSC-iM formation, the effect of other inflammatory cytokines known to be elevated in COVID19 is stronger than type I IFNs on purified HSCs in our experiments.

Additionally, in the original study by Cheong et al., the sample sizes were COVID-19 = 19, ICU-control = 11, and healthy = 19. However, in the current study, the sample sizes have been reduced to COVID-19 = 4, ICU-control = 6, and healthy = 7. Can the authors clarify the reasons for this reduction in sample size?

We clarify that not all samples were subjected to scMultiome. The samples profiled by scMultiome and used in our study are a subset of those described by the Referee. This is indicated in the original Cheong et al manuscript in Table S1 and summarized in Referee Table 1. We confirm that there were only 4 samples from early convalescence patients after recovery from severe COVID, 6 non-COVID ICU-admitted controls and 7 healthy donors for which scMultiome was performed and analyzed.

3. The authors demonstrated that the memory program of HSC-iM is more similar to memory T cells. Could this observation be influenced by the xenograft model system? Notably, the model was established using fetal CD34+ cells from cord blood, which are known to have distinct transcriptional and epigenetic profiles that promote rapid growth and differentiation, particularly through the upregulation of genes associated with proliferation and lymphoid differentiation.

We do not make claims wherein we compare the inflammatory memory in HSCs to memory across different cell types. To clarify, we assume the Referee means to point out that HSC-iM are more similar to memory T cells compared to effector or naive T cells. We thank the Referee for noting the potential similarity in activation and differentiation properties between fetal HSCs and T cells. We note that we used umbilical cord blood (CB)-derived HSCs, distinct from fetal liver-

derived HSCs. Notably, we do not believe the xenograft system influences this characteristic because we find the HSC2 population - the correlate of HSC-iM - in HSCs isolated from human donor bone marrow from our CH cohort[20]. To ensure completeness of our analyses, we also evaluated enrichment of a memory T cell RNA signature (as described in Extended Data Fig. 5 of the revised manuscript) in the adult BM-derived HSC2 vs HSC1. As expected, HSC2 were also enriched in the memory T cell signature (T-mem) (Referee Fig. 31). Thus, neither the xenograft setting nor the neonatal source of CB is required for establishing the enrichment of memory T cell-like properties in HSC-iM.

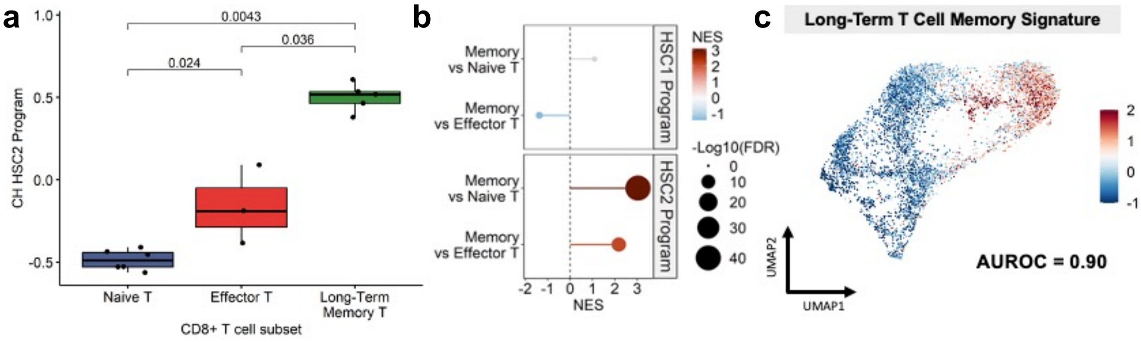

Referee Fig. 31: Conservation of gene expression between CH HSC2/HSC-iM and functional T cell memory. A, Enrichment of a gene expression program composed of the top 200 differentially expressed genes specific to CH BM HSC2 in human T cell subsets from Akondy et al. b, GSEA results depicting the enrichment of the CH BM HSC1/HSC-I and HSC2/HSC-iM programs between human T cell subsets from Akondy et al. c, AUCell enrichment of the CD8 long-term T cell memory signature in the CH BM HSPC data

Can the authors compare these findings to an adult CD34+ xenograft model to determine whether the observed memory program is unique to fetal HSCs or shared across different HSC sources?

We undertook a new set of transplant experiments using adult bone marrow samples from our CH cohort (see response to Referee#1 point 2 and Referee Fig. 6 and 8) to address this important concern. In summary, the HSC-iM state is retained through transplantation.

4. Interestingly, the authors indicate that they have identified a memory program in HSCs that differs from those reported in BCG studies, both in mice and humans. This is a critical observation, as humans are exposed to a variety of pathogens that cause acute or chronic infections, each with distinct characteristics, such as structural differences (e.g., BCG lacks LPS), replication rates, and pathogenicity.

For instance, a recent study (PMID: 33125891) comparing the BCG vaccine to a virulent strain of Mycobacterium tuberculosis (Mtb, the causative agent of TB) showed that, unlike BCG, Mtb-induced HSC memory responses result in a distinct program characterized by reduced engraftment and the generation of monocytes/macrophages that are more susceptible to subsequent Mtb infection. Can the authors compare their identified HSC memory program to the existing Mtb data to highlight potential similarities or differences?

We have investigated the HSC-iM transcriptional program in the context of murine HSCs 28 days following exposure to Mtb vs control. The DEGs reported in the supplement of Khan et al

were retrieved and 'babel gene' was used to convert the gene names from mouse to human. GSEA was performed with the HSC-iM program, which was found to be negatively enriched in Mtb vs Ctl (Referee Fig. 32a). To investigate further, we isolated significant DEGs (FDR<0.05 and log2FC>0.585 or <-0.585) from murine HSCs after Mtb or BCG exposure, and compared to significant DEGs in HSC-iM (vs HSC-I; reported in Supplementary Table 9). A very limited subset of these genes overlap (Referee Fig. 32b,c), and their expression/rank vary dramatically between the two datasets. Whether this is simply because of species-related differences or due to fundamentally different composition of transcriptional memory after Mtb/BCG vs single inflammatory agents remains to be explored.

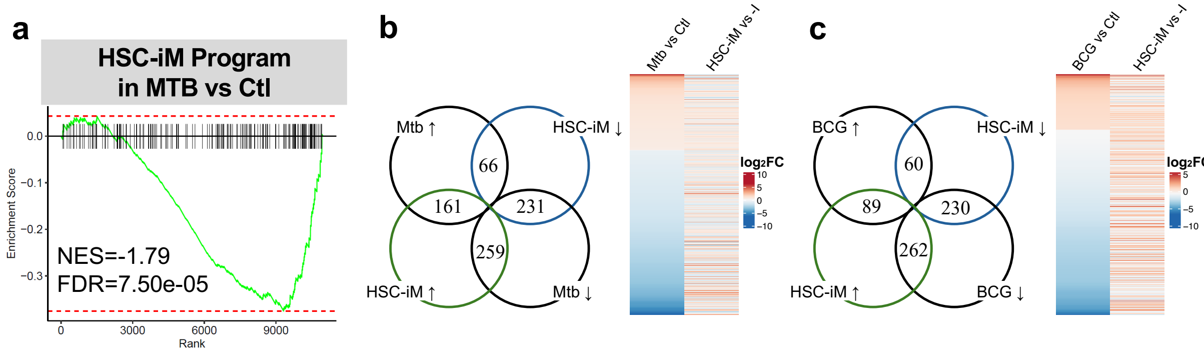

Referee Fig. 32: Comparison between transcriptional profiles of xenograft-derived HSC-iM and murine HSCs after *Mycobacterium tuberculosis* (Mtb) or Bacille Calmette-Guérin vaccine (BCG) exposure, from Khan et al. (2020). a, GSEA results showing enrichment of the HSC-iM program in DEGs derived from murine HSCs after their exposure to Mtb. b-c, Significant DEGs in murine HSCs after either Mtb (b) or BCG (c) exposure were translated to human homologs and compared with DEGs in HSC-iM (FDR<0.05, log2FC>0.585 or <-0.585). Overlapping number of genes (<3.5% of total set for each comparison) are indicated in Venn diagrams, and a heatmap shows their differential expression in murine Mtb/BCG vs HSC-iM datasets.

Furthermore, in real-world conditions, humans are rarely exposed to isolated factors like LPS or TNF-α. How would cumulative exposure to multiple pathogens or inflammatory stimuli influence the development and functional outcomes of HSC-iM? Addressing these questions could significantly enhance our understanding of how diverse environmental and pathogenic exposures shape HSC memory.

We agree. The best strategy would be to perform a large epidemiological study where we have a complete clinical history of the study participant's infectious and environmental exposures - such as air pollution - to extend our new findings from the Ontario Health Study (Referee Fig. 3 and response to Referee #1 point 1).

REFERENCES

1. Sobrino, S. *et al.* Severe inflammation and lineage skewing are associated with poor engraftment of engineered hematopoietic stem cells in patients with sickle cell disease. *Nat. Commun.* **16**, 3137 (2025).
2. Abelson, S. *et al.* Prediction of acute myeloid leukaemia risk in healthy individuals. *Nature* **559**, 400–404 (2018).
3. Jaiswal, S. *et al.* Age-related clonal hematopoiesis associated with adverse outcomes. *N. Engl. J. Med.* **371**, 2488–2498 (2014).
4. van Galen, P. *et al.* Single-cell RNA-seq reveals AML hierarchies relevant to disease progression and immunity. *Cell* **176**, 1265–1281.e24 (2019).
5. Abbas, H. A. *et al.* Single cell T cell landscape and T cell receptor repertoire profiling of AML in context of PD-1 blockade therapy. *Nat. Commun.* **12**, 6071 (2021).
6. Wang, B. *et al.* Comprehensive characterization of IFNγ signaling in acute myeloid leukemia reveals prognostic and therapeutic strategies. *Nat. Commun.* **15**, 1821 (2024).
7. Lasry, A. *et al.* An inflammatory state remodels the immune microenvironment and improves risk stratification in acute myeloid leukemia. *Nat. Cancer* **4**, 27–42 (2023).
8. Beneyto-Calabuig, S. *et al.* Clonally resolved single-cell multi-omics identifies routes of cellular differentiation in acute myeloid leukemia. *Cell Stem Cell* **30**, 706–721.e8 (2023).
9. Zeng, A. G. X. *et al.* Single-cell transcriptional atlas of human hematopoiesis reveals genetic and hierarchy-based determinants of aberrant AML differentiation. *Blood Cancer Discov.* **6**, 307–324 (2025).
10. Horne, B. D. *et al.* Exceptional mortality prediction by risk scores from common laboratory tests. *Am. J. Med.* **122**, 550–558 (2009).
11. Engelsgjerd, E. K., Benziger, C. P. & Horne, B. D. Validation of the Intermountain Risk Score and Get with the Guidelines-Heart Failure Score in predicting mortality. *Open Heart* **8**, e001722 (2021).
12. Mert İlker, H., Faysal, S., Ahmet Çağdaş, Y., Murat, S. & Tufan, Ç. Prognostic value of Intermountain Risk Score for short- and long-term mortality in patients with cardiogenic shock. *Coron. Artery Dis.* **34**, 154–159 (2023).
13. Horne, A. W. & Missmer, S. A. Pathophysiology, diagnosis, and management of endometriosis. *BMJ* **379**, e070750 (2022).
14. Cheong, J.-G. *et al.* Epigenetic memory of coronavirus infection in innate immune cells and their progenitors. *Cell* **186**, 3882–3902.e24 (2023).
15. Naik, S. *et al.* Inflammatory memory sensitizes skin epithelial stem cells to tissue damage. *Nature* **550**, 475–480 (2017).
16. Li, J. *et al.* AP-1 mediates cellular adaptation and memory formation during therapy resistance. *bioRxivorg* (2024) doi:10.1101/2024.07.25.604999.
17. Nagaraja, S. *et al.* Clonal memory of colitis accumulates and promotes tumor growth. *bioRxivorg* (2025) doi:10.1101/2025.02.13.638099.
18. Mazurier, F., Gan, O. I., McKenzie, J. L., Doedens, M. & Dick, J. E. Lentivector-mediated clonal tracking reveals intrinsic heterogeneity in the human hematopoietic stem cell compartment and culture-induced stem cell impairment. *Blood* **103**, 545–552 (2004).
19. Lasaga, M. *et al.* Gene therapy restores the transcriptional program of hematopoietic stem cells in Fanconi anemia. *Haematologica* **108**, 2652–2663 (2023).
20. Jakobsen, N. A. *et al.* Selective advantage of mutant stem cells in human clonal

hematopoiesis is associated with attenuated response to inflammation and aging. *Cell Stem Cell* **31**, 1127–1144.e17 (2024).
21. Biasco, L. *et al.* In Vivo Tracking of Human Hematopoiesis Reveals Patterns of Clonal Dynamics during Early and Steady-State Reconstitution Phases. *Cell Stem Cell* **19**, 107–119 (2016).
22. Zhang, X. *et al.* An immunophenotype-coupled transcriptomic atlas of human hematopoietic progenitors. *Nat. Immunol.* **25**, 703–715 (2024).
23. Furtwängler, B. *et al.* Mapping early human blood cell differentiation using single-cell proteomics and transcriptomics. *Science* eadr8785 (2025).
24. Ng, S. W. K. *et al.* A 17-gene stemness score for rapid determination of risk in acute leukaemia. *Nature* **540**, 433–437 (2016).
25. Furer, N. *et al.* A reference model of circulating hematopoietic stem cells across the lifespan with applications to diagnostics. *Nat. Med.* **31**, 2442–2451 (2025).
26. SanMiguel, J. M. *et al.* Distinct Tumor Necrosis Factor Alpha Receptors Dictate Stem Cell Fitness versus Lineage Output in Dnmt3a-Mutant Clonal Hematopoiesis. *Cancer Discov.* **12**, 2763–2773 (2022).
27. Kotliar, D. *et al.* Reproducible single-cell annotation of programs underlying T cell subsets, activation states and functions. *Nat. Methods* **22**, 1964–1980 (2025).
28. Herminghaus, A. & Osuchowski, M. F. How sepsis parallels and differs from COVID-19. *EBioMedicine* **86**, 104355 (2022).
29. Ghaffarpour, S. *et al.* Cytokine profiles dynamics in COVID-19 patients: a longitudinal analysis of disease severity and outcomes. *Sci. Rep.* **15**, 14209 (2025).

Referees' comments:
Responses in blue

Referee #1 (Remarks to the Author):

I appreciate the data on human health (SCD, AML, all-cause mortality) that the authors have added, and their study is now a very comprehensive survey of a molecularly distinct HSC subset that accumulates in inflammaging and as a long-term consequence of inflammatory insults. This study is important, because it highlights the transversal importance of inflammation (and not clonal hematopoiesis mutations) in shaping health outcomes, and because this hitherto not well described HSC subset might be of large relevance also in other contexts such as trained immunity.
We thank the referee for finding the revised study to be important and comprehensive.

I recommend its publication, with a few final recommendations:

- The manuscript requires **editing and shortening**. I struggled both during my initial review and my re-review to understand all messages and the experimental evidence that they are based on. Key messages should be distilled more clearly. Description of experimental setups and analyses should be simplified. and unnecessary detail removed.
We have edited extensively throughout the manuscript including reducing the main text from >7000 words to <4500.

- **Good sections to shorten drastically** would be "transmission of inflammatory memory programs from HSC-iM to downstream progeny" and the section on extended data figure 8. These sections are still not fully conclusive, as discussed also in the author's reply to my original points 2 and 6. This regards the "lineage tracing" setup employed, but also analysis from figure EV8: I drew the bar chart of figure EV8h as a scatter plot (% HSC-iM in primary vs. xenotransplant) and there was no correlation. I think these sections are not required to be included prominently, as the manuscript has been strengthened elsewhere, particularly regarding health relevance. I follow the argument of the authors that clonal tracing in human HSCs might be beyond the scope of this study. I believe that good tools for this task now exist (10.1038/s41586-025-09041-8) and could be cited in the discussion.
We have refined the text for this section and altered the data presentation within the figure to make this section more accessible and clear.

- I liked the wording employed in the rebuttal letter, "These data raise a novel hypothesis that HSC-iM enrichment reflects the systemic level of inflammation in unhealthy individuals, and that some of the health correlations attributed to CH status in current literature could be survivorship bias of an earlier HSC-iM-driven inflammatory state." This could be included in the main text.
We have added a statement from our Referee response letter on HSC-iM and CH survivorship bias as requested into the Discussion as requested.

- I would suggest to discuss the extent to which the HSC-iM signature might capture more general inflammation marks and not always be related to HSC-iM. In particular, in the context of all cause mortality / intermountain risk score prediction, any inflammation signature would probably work as well? See for example 10.1016/j.ajhg.2025.06.009 . Also they could cite 10.1038/s41467-025-58321-4 in the context of HSC-iMs and SCD.

The referee provides an insightful suggestion. We have included the following statement in our discussion: “The experimentally identified HSC-iM molecular program is reflective of a lifetime of accumulated inflammatory insults in multiple human physiological and disease settings: severe COVID-19, congenital blood disorders, aging, and CH.”

Regarding the Sobrino et al study, we utilized the SCD data set and it is cited in the revised manuscript.

Referee #2 (Remarks to the Author):

Xie and colleagues have considered reviewers comments thoroughly and performed extensive new experiments and analyses, and prepared a revised manuscript that clarifies and expands their findings on human HSCs with epigenetic inflammatory memory. They extend their findings further to disease settings by showing increased HSC-iM in children with sickle cell disease, and in patients with myeloid malignancies. They show that HSC-iM inflammatory state is reflected in HSC functional properties upon transplantation and transmits to both myeloid and lymphoid progeny. They document altered cytokine secretion in mice transplanted with HSC-iM. They also include analysis of a large transcriptome data set of 400 individuals and show that HSC-iM signature correlates with a high risk score for 5 year all cause mortality, strengthening the link between HSC-iM and clinical impact. The authors made extensive attempts to identify a surface marker for HSC-iM, including performing Cite-seq, and I applaud their efforts, even though they were not able to identify a suitable surface marker for prospectively isolating HSC-iM. The authors have responded to reviewer criticisms carefully and made revisions that make the manuscript and figures more accessible for broader audiences. I am convinced that publishing their novel findings will have an impact on the field, and allow future studies built on it. I would be happy to recommend publication of the revised manuscript in Nature.
We are grateful for the Reviewer's efforts in providing meaningful feedback and finding our revised study to be thorough and important for a broad audience.

Referee #3 (Remarks to the Author):

The authors have added a plethora of new data which further supports their observation of a human HSC subset that retains molecular and functional memory of prior inflammatory stress. This significantly strengthens the manuscript. There are many novel and interesting concepts generated from this work. For example, were any differences in the HSC-iM population observed between CH with DNMT3A versus TET2 mutations? The authors seem to have compiled them together for their figures. But I would not dare ask any further experimentation or analysis from the authors. They have gone above and beyond in the revised manuscript. I credit the group for their diligence, and this study will set the stage for multiple opportunities for investigation in the coming years. The originality and significance is high as it identifies a new human HSC signature across multiple age ranges and disease stages. They have generated a huge amount of data to support this which will be a valuable resource for the community. The conclusions are robustly supported by the data. I have no further concerns.

- GAC
We greatly appreciate the Referee''s time and recommendations which have played an important role in helping to improve our manuscript.
With respect to the one question the reviewer raises, we would like to note the following:
-in Figure 4k we denote which donors have TET2 vs DNMT3A mutations, highlighting any potential differences in the proportion of HSC-iM for each. While the number of donors is low, we did not observe any clear difference.

-in Figure 4f-h the mutation status is noted when evaluating enrichment of HSC-iM; again, no clear differences.
-In Figure 4j and Extended Figure 7h, we show how many specific genes are different within HSC-iM when CH^{Mut} vs CH^{WT} cells are compared in DNMT3A or TET2 mutated HSCs. Regulon activity is shown in Extended Data Fig 7d and mutation status is denoted, with HSC-iM specific Regulon activity reported in Extended Data Fig 7h. Here, there do appear to be some differences; specific results can be found in Supplementary Table 17.

Referee #4 (Remarks to the Author):

The authors experimentally addressed all my concerns.
We thank the Referee for their time and are pleased we have addressed all concerns.
